# Unsuccessful Coupling of Weakly Coordinating Anion–Based Mg Salts and Alkoxyalkylamine Solvents for Rechargeable Mg Battery Application: Trace Water‐Driven Interfacial Failure

**DOI:** 10.1002/advs.76897

**Published:** 2026-07-29

**Authors:** Toshihiko Mandai, Antoine Barthélemy, Hendrik Koger, Harald Scherer, Ingo Krossing

**Affiliations:** ^1^ Research Center for Energy and Environmental Materials (GREEN) National Institute for Materials Science (NIMS) Tsukuba Ibaraki Japan; ^2^ Institute For Inorganic Chemistry and Analytic Chemistry as Well as Freiburg Materials Research Center FMF University of Freiburg Freiburg Germany

**Keywords:** anion, electrolyte, interface, magnesium battery, solvation

## Abstract

Rechargeable magnesium metal batteries (RMMBs) are promising candidates for large‐scale energy storage owing to the advantageous properties of magnesium metal negative electrodes. Recent advances, particularly in electrolyte chemistry, have accelerated progress toward practical RMMBs. Here, we combined two emerging concepts—weakly coordinating anion (WCA)‐based Mg salts and alkoxyalkylamine solvents—in electrolyte design. Unexpectedly, the combination of a representative WCA‐based Mg salt, Mg[Al(Ohfip)_4_]_2_, and a conventional alkoxyalkylamine, 2‐methoxyethylamine (MOEA), resulted in inferior electrochemical performance, whereas MOEA favored strongly coordinating anion‐based Mg salts such as Mg[TfO]_2_. Comprehensive analyses revealed distinct anion‐dependent solvation structures and interfacial chemistries. In Mg[Al(Ohfip)_4_]_2_ systems, the anion and the solvent complexes are only minimally involved in initial Mg‐interface formation. Yet, the presence of trace water in MOEA induces the MOEA‐mediated partial hydroxylation of the anion [Al(Ohfip)_4_]^−^, which leads to the formation of unstable oxygen‐rich interfaces upon cycling. On the contrary, Mg^2+^, [TfO]^−^, and MOEA form associative ion‐paired complexes that jointly contribute to the formation of fluorine‐ and nitrogen‐rich hybrid interfaces that suppress parasitic reactions and enhance performance. Despite these benefits, the associative Mg[TfO]_2_–MOEA electrolyte still exhibits limitations for practical RMMB application. The complex dilemma in developing rational electrolyte materials for RMMB applications will also be discussed.

## Graphical abstract

Combining recently established two ground‐breaking concepts, the adoption of WCAs and the use of alkoxyalkylamine‐based solvents in electrolyte formulations for rechargeable magnesium metal batteries, has led to unexpectedly unsuccessful electrochemical Mg plating/stripping performance. Comprehensive experimental and computational analyses revealed the amine‐mediated peculiar failure mechanism caused by unavoidable water impurity.

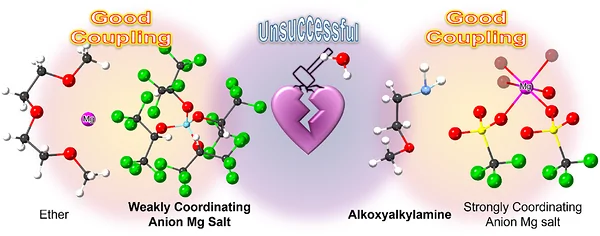

## 1 Introduction

The global demand for electricity continues to rise exponentially with advancements in science and technology. In recent years, the rapid expansion of artificial intelligence has further accelerated electricity consumption worldwide. This trend necessitates continuous innovation in energy storage technologies that harness renewable power sources such as wind and solar energy. According to market forecasts, global battery demand is expected to exceed 5000 GWh by 2040—far surpassing the available lithium capacity [1]. Therefore, the development of rechargeable batteries based on non‐lithium chemistries has become increasingly imperative. Among these, rechargeable magnesium metal batteries (RMMBs) are particularly promising for large‐scale energy storage applications, owing to the high 2.33% natural abundance of magnesium in the earth crust (cf. Li: 0.006%) [2, 3]. The theoretical energy density of RMMBs can rival that of current Lithium Ion Batteries (LIBs), benefiting from the two‐electron chemistry enabled by the transport of divalent Mg^2+^ ions. Moreover, the electrochemical and chemical properties of Mg metal—its low tendency to form dendrites during electroplating and the inherent stability of bulk Mg against ambient conditions—make it an attractive choice for developing safe, practical batteries.

The development and successful application of weakly coordinating anion (WCA)‐based electrolytes have driven significant recent progress in the field of RMMBs [4, 5]. Certain WCA‐based electrolytes have demonstrated reversible Mg plating and stripping behavior with excellent cycling efficiency, even in the absence of conventional double‐edged reagents such as [BH_4_]^−^ and chloride species [6, 7, 8]. A wide range of WCAs, primarily composed of group XIII element complexes, have been investigated to date, including [HCB_11_H_11_]^−^ [6, 9], [HCB_11_H_10_F]^−^ [10], [B(Otfe)_4_]^−^(Otfe = OCH_2_CF_3_) [11, 12], [B(Ohfip)_4_)]^−^(Ohfip = OCH(CF_3_)_2_) [8, 13, 14, 15, 16], [B(Otfe)_3_(TfO)]^−^ (TfO = OSO_2_CF_3_) [17], and [Al(Ohfip)_4_]^−^ [7, 14, 18]. A recent comprehensive review by Riedel et al. systematically compared various WCA‐based RMMB electrolytes in terms of their electrochemical performance, stability, and synthetic feasibility [19]. Among them, [B(Ohfip)_4_]^−^ and [Al(Ohfip)_4_]^−^‐based electrolytes are regarded as state‐of‐the‐art systems owing to their well‐balanced electrochemical and practical characteristics.

Concurrently, extensive efforts have been devoted to exploring solvents suitable for high‐energy RMMBs. Ethereal solvents are generally considered the first choice because of their excellent compatibility with Mg electrodes. To further enhance the performance of ethereal electrolyte solutions, several strategies have been investigated, including structural modification of ether molecules to tune their physicochemical properties [20, 21], incorporation of additives and co‐solvents in dual‐solvent systems [22, 23, 24, 25, 26, 27, 28], and substitution of ethers with alternative solvent classes [29]. However, due to the limited compatibility of non‐ethereal solvents with Mg electrodes [30], the co‐solvent approach remains a feasible and promising route to meet diverse design requirements without compromising the inherent advantages of ethereal electrolytes. Among non‐ethereal candidates, amines and their derivatives exhibit both high reductive stability and strong solvating ability. Although the beneficial effects of incorporating amine‐based solvents into conventional ethereal RMMB electrolytes were first reported in the 1930s [31], this strategy has attracted renewed and growing attention in recent years [22, 25, 32, 33, 34]. Enhanced electrochemical performance, particularly at the Mg electrode interface, has been achieved by incorporating certain alkoxyalkylamines and alkyldiamines into conventional ethereal electrolytes. Comprehensive experimental and computational studies have revealed the critical influence of reactive nitrogen‐bound hydrogen atoms within the amine molecular structure [35, 36, 37]. The formation of MgH_2_‐containing species, originating from parasitic reactions between primary or secondary amines and the Mg metal surface, promotes the development of compositionally graded inorganic–organic interphases accompanied by anion decomposition. These interphases effectively suppress further electrolyte decomposition while facilitating interfacial charge transfer, functioning analogously to conventional solid–electrolyte interphases (SEIs) [22, 25, 34]. Furthermore, Mg^2+^ coordination by amine molecules in solution markedly affects the rate‐determining steps of Mg plating and stripping, including charge‐transfer kinetics and the desolvation process [35].

Given the synergy demonstrated by recent advances in WCAs and amine co‐solvents for RMMB electrolytes, the development of promising new electrolyte systems can be anticipated by combining these two groundbreaking concepts. In this work, we systematically examined the electrochemical characteristics of WCA‐based ether–amine dual‐solvent electrolytes and comprehensively investigated the influence of the paired anion species on their performance. Diglyme (G2) and 2‐methoxyethylamine (MOEA) were adopted as benchmark solvents owing to their proven applicability in RMMB electrolytes and their favorable solubility for the studied salts (ESI; §3) [14, 18, 25, 34]. Two anions with distinctly different coordinating abilities—[Al(Ohfip)_4_]^−^ and [TfO]^−^—representing weakly coordinating and strongly coordinating counterions, respectively, were selected to elucidate the role of anions in electrochemical and interfacial behaviors.

Unexpectedly, no beneficial synergistic effect was observed for the WCA–amine combination; rather, the system exhibited inferior electrochemical performance. Note that, due to its chemical nature as a base and a primary amine, MOEA typically contains higher amounts of hydrogen‐bonded trace water that is difficult to remove or even quantify. In addition, this trace water may act as an acid toward the MOEA base and hence may form hydroxide ions and protonated MOEA in equilibrium. Both may possibly influence the electrolyte performance, as indicated by comprehensive interfacial analyses, combined with detailed insights into the coordination states of Mg^2+^ in solution. Our investigations revealed an MOEA‐mediated trace‐water‐driven hydroxylation‐based failure mechanism within the WCA–amine system, which is particularly critical for regulating the Mg electrode–electrolyte interface. Finally, the complex dilemma associated with developing next‐generation electrolyte materials for RMMB applications is discussed with new facets.

## 2 Results and Discussion

### 2.1 Mg Plating/Stripping Performance and Morphological Evolution

Based on the preliminary compositional survey with respect to the salt solubility and primary electrochemical performance, a 1:1 (v/v) mixture of G2 and MOEA was employed as a benchmark solvent system. The chemistry behind the salt solubility and basic electrochemical properties is detailed in the electronic supporting information (ESI; §3 and Figures S1–S4). The electrochemical Mg plating/stripping performance of the electrolytes containing Mg[Al(Ohfip)_4_]_2_ and Mg[TfO]_2_ salts were evaluated by systematic galvanostatic cycling and impedance measurements. The galvanostatic cycling results revealed a pronounced dependence of electrochemical performance on the nature of the anion. Unexpectedly, an electrolyte incorporating [Al(Ohfip)_4_]^−^ exhibited unstable Mg plating/stripping behavior, irrespective of the cell configuration, and these cells experienced short‐circuiting at relatively early stages of cycling (Figure 1a). A similarly poor electrochemical performance was also observed for the electrolyte containing the structurally analogous [B(Ohfip)_4_]^−^ anion (Figure S5), indicating the limited compatibility of dual‐solvent electrolyte systems with G2–MOEA and fluorinated alkoxyaluminate and alkoxyborate WCA‐salts. In contrast, the coordinating‐anion‐based Mg[TfO]_2_/G2‐MOEA electrolyte displayed excellent Mg plating/stripping performance in both symmetric and asymmetric cell configurations. These cells exhibited highly stable cycling with minimal polarization for more than 600 h, even at a practical current density of 1 mA cm^−2^ (Figure 1b). The galvanostatic cycling‐hold measurements further corroborate the greater static stability of the Mg[TfO]_2_‐based against the Mg[Al(Ohfip)_4_]_2_‐based electrolyte (Figure S6).

**FIGURE 1:**
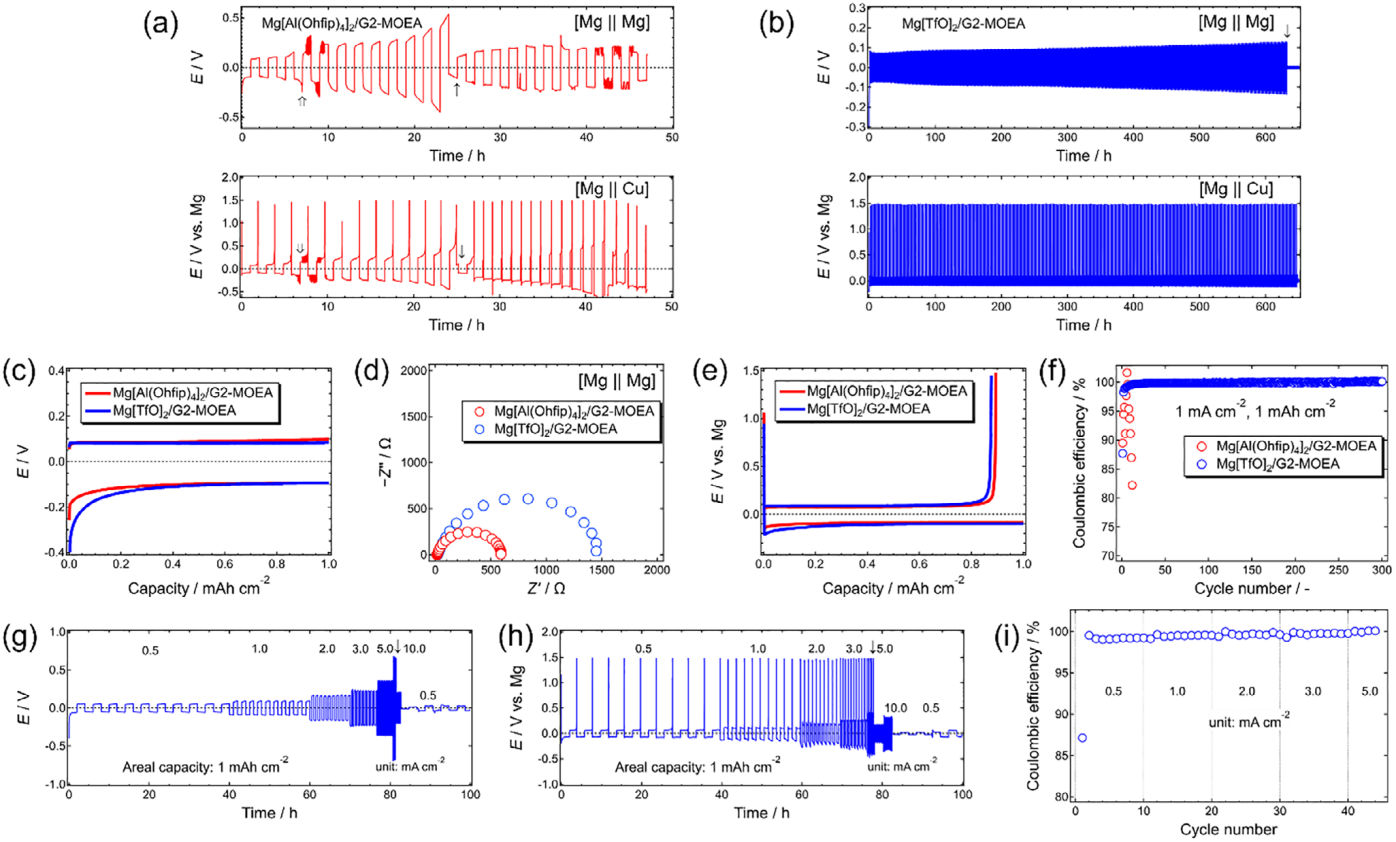
(a, b) Galvanostatic Mg plating/stripping cycling profiles of symmetric [Mg || Mg] and asymmetric [Mg || Cu] cells using G2–MOEA‐based electrolytes. Polarization curves of (c) symmetric and (e) asymmetric cells. (d) Electrochemical impedance spectra of symmetric cells before cycling. (f) Coulombic efficiencies. Rate capabilities of Mg[TfO]_2_/G2‐MOEA electrolytes with (g) symmetric and (h) asymmetric cells and (i) corresponding Coulombic efficiencies. Double arrows and single arrows in the cycling profiles indicate microshorts and definite shorts, respectively.

A closer examination of the polarization behavior revealed that the electrolytes containing weakly coordinating [Al(Ohfip)_4_]^−^ and strongly coordinating [TfO]^−^ anions exhibited similar characteristics, regardless of the cell configuration. The overpotential for Mg plating and stripping stabilized at approximately 80 mV after the initial nucleation stage. The difference in the depth of the nucleation overpotential between the Mg[Al(Ohfip)_4_]_2_‐ and Mg[TfO]_2_‐based electrolytes—more pronounced in the symmetric cell configuration (Figure 1c)—is most likely attributable to their distinctly different interfacial impedances, as shown in Figure 1d. The smaller interfacial charge‐transfer resistance observed for the Mg[Al(Ohfip)_4_]_2_‐based electrolyte corresponds to the shallowest nucleation overpotential. The Coulombic efficiency for Mg plating/stripping during the first cycle was also nearly identical between these two electrolytes (around 90%); however, their long‐term cycling stability differed significantly. The Coulombic efficiency of the Mg[Al(Ohfip)_4_]_2_/G2‐MOEA electrolyte fluctuated throughout the first 13 cycles (Figure 1f), and micro‐short‐circuiting events, possibly arising from inhomogeneous Mg plating/stripping reactions (vide infra), were observed. In contrast, the efficiency of the Mg[TfO]_2_/G2‐MOEA electrolyte stabilized rapidly after the initial formation and activation processes, approaching 99.9% in subsequent cycles. This electrolyte also demonstrated outstanding Mg plating/stripping performance even at higher current densities, up to 5 mA cm^−2^, regardless of the cell configuration examined (Figure 1g,i).

The morphological characteristics of the deposits reflect the differences in electrochemical performance among the electrolytes. As shown in Figure S7, crystalline granular deposits with an average size of less than 10 µm were formed on the substrates regardless of the electrolyte type; however, their surface morphology differed significantly. The magnified scanning electron microscopy (SEM) images revealed that, in the Mg[Al(Ohfip)_4_]_2_‐based electrolyte, highly crystalline angular primary particles coalesced (Figure S7b), whereas in the Mg[TfO]_2_‐based, smaller primary crystals fused together to form rounded secondary particles (Figure S7d). Subsequent powder X‐ray diffraction (pXRD) analysis confirmed successful plating of hcp‐Mg with high crystallinity and almost identical crystal orientation on the substrates in both electrolytes (Figure S7e). Despite exhibiting similar electrochemical Mg plating behavior, the weakly coordinating Mg[Al(Ohfip)_4_]_2_ and the strongly coordinating Mg[TfO]_2_ electrolytes showed a pronounced difference in their long‐term plating/stripping performance (Figure 1a,b). These observations strongly suggest that interfacial stability largely depends on the coordinating characteristics of the anions in the electrolyte solution and potentially also the capacities of the system to interact with the trace water present, e.g., by deprotonation and hydroxide formation (vide infra.).

Repeated stripping and plating cycles inevitably induce successive morphological changes in the metal electrodes. The characteristic plating behavior of Mg on substrates containing pits is widely recognized as a critical issue in the field of RMMBs, because such localized plating results in a markedly rough surface [13, 38, 39, 40, 41]. This roughness, in turn, severely limits the active surface utilization and increases the risk of short‐circuiting through the intrusion of metallic Mg into porous separators. The surface morphology of Mg after a single stripping–plating cycle in the Mg[Al(Ohfip)_4_]_2_‐based electrolyte clearly exhibited this characteristic feature, as shown in Figure 2a,b. The deposits preferentially accumulated around the pits formed during the previous stripping process, while granular deposits were also observed both inside the pits and on the unreacted surface areas. As Mg tends to deposit preferentially on less resistive areas [42], this observation suggests spatially inhomogeneous passivation. The surface image obtained after a single stripping–plating cycle also indicated partial detachment of the deposits from the substrate (Figure 2b). A similar low‐adhesive behavior has also been reported for Mg deposited after a single plating process—without a preceding stripping step—in the MOEA‐free ethereal Mg[Al(Ohfip)_4_]_2_/G2 electrolyte [40]. The adhesion of the deposits to the substrate is particularly important for long‐term cycling stability, as weakly adherent deposits can easily lose physical contact with the substrate, forming electrically isolated “dead” Mg, which in turn leads to poor Coulombic efficiency and an increased risk of short‐circuiting upon percolation. Fractured “dead” Mg was directly observed in the [Mg || Mg] symmetric cells with the Mg[Al(Ohfip)_4_]_2_/G2‐MOEA electrolyte during repeated plating and stripping cycles by in situ electrochemical confocal microscopy (vide infra). The cross‐sectional view of the deposits on the substrate provided additional insights into the stripping–plating behavior. Figure 2c shows the cross‐sectional SEM image of the Mg electrode after a single stripping–plating cycle, obtained using a focused ion beam technique. Semicircular, granular Mg particles were deposited on the underlying Mg substrate. The corresponding high‐resolution scanning transmission electron microscopy (STEM) image, together with position‐resolved electron diffraction, energy‐dispersive X‐ray spectroscopy (EDX), and electron energy loss spectroscopy (EELS), clearly revealed the formation of an oxygen‐ and fluorine‐containing amorphous phase at the interface between the deposits and the substrate (Figure 2d–g).

**FIGURE 2:**
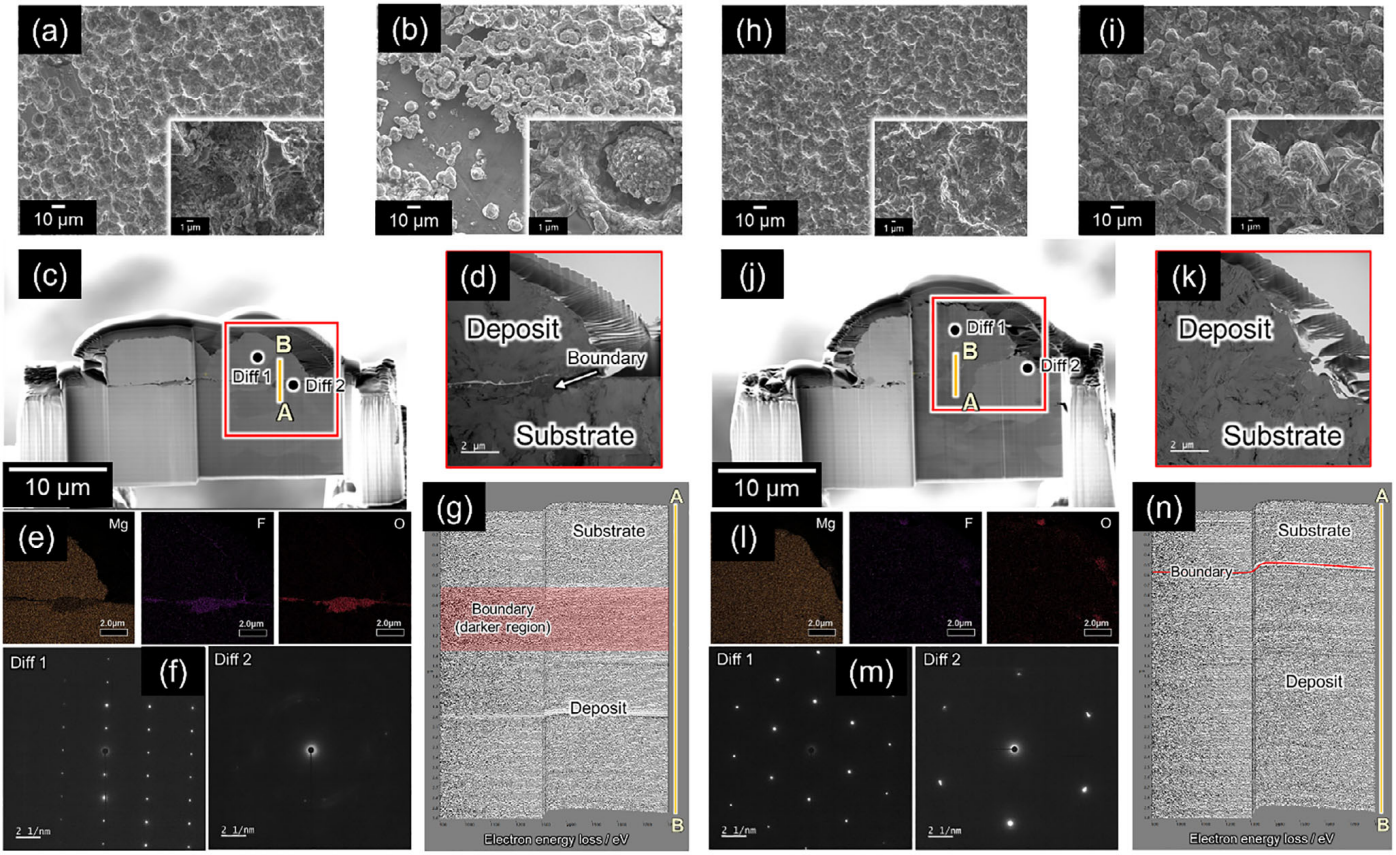
SEM and STEM analyses of Mg metals after electrochemical stripping–plating reactions conducted in (a–g) Mg[Al(Ohfip)_4_]_2_/G2‐MOEA and (h–n) Mg[TfO]_2_/G2‐MOEA electrolytes. Surface SEM images of Mg metals after (a, h) a single stripping process and (b, i) one stripping–plating cycle at a current density of 1 mA cm^−2^ and a geometrically fixed areal capacity of 1 mAh cm^−2^. Cross‐sectional (c, j) SEM images and the corresponding (d, k) STEM images, (e, l) EDX mappings, (f, m) position‐resolved electron diffraction patterns, and (g, n) EELS spectra of the deposits and substrates are shown. The positions of STEM observations, diffraction measurements, and EELS analyses are indicated in (c) and (j).

In the case of the Mg[TfO]_2_‐based electrolyte, granular Mg deposits were observed uniformly across the substrate after a single stripping–plating cycle (Figure 2i). Such distinct deposition behavior suggests the distinct interfacial chemistries between the Mg[Al(Ohfip)_4_]_2_‐ and Mg[TfO]_2_‐based electrolytes. The detailed cross‐sectional STEM image, combined with subsequent comprehensive analyses, demonstrated favorable Mg plating behavior in the Mg[TfO]_2_‐based electrolyte. Only a very limited amorphous phase was detected at the interface between the deposits and the substrate, as confirmed by EDX, position‐resolved electron diffraction, and EELS analyses (Figure 2l–n). These observations strongly suggest the formation of a stable interfacial layer in the Mg[TfO]_2_‐based electrolyte.

Visual observation is a powerful learning tool, especially in the field of battery research. Directly monitoring the dynamic plating and stripping processes provides crucial insights into interfacial behavior and morphological evolution across different electrolytes. To visualize the Mg plating/stripping behavior under operating conditions, a specially designed electrochemical cell was fabricated in a symmetric [Mg || Mg] configuration, incorporating a conventional glass fiber separator. Galvanostatic cycling measurements were then performed under a confocal microscope. As shown in Videos 1 and 2 deposited with the supporting information, the dynamic behavior varied significantly depending on the electrolyte used. In the case of the Mg[Al(Ohfip)_4_]_2_‐based electrolyte (Video 1), the electrode surfaces were already damaged by the electrolyte prior to electrochemical measurements, and fractured Mg particles had penetrated into the porous separator. The exposed electrode surface gradually responded to the applied current, and localized plating and subsequent stripping of Mg metal were observed at the facing Mg electrodes, accompanied by minimal gas evolution. The snapshots captured at specific reaction intervals further supported these dynamic observations (Figure S8). The cell experienced an undesired short circuit during the third plating/stripping cycle, as evidenced by irregular voltage fluctuations, although this event was unfortunately not visible within the observed field. This observation is in good agreement with the unstable electrochemical performance shown in Figure 1a. The percolation of isolated Mg particles through localized, uneven plating/stripping likely accelerated the short‐circuit event. In contrast, surface reactions accompanied by significant gas evolution (bubbling) initiated immediately after the current was applied in the cell employing the Mg[TfO]_2_‐based electrolyte (Video 2). These observations suggest that surface reactions are likely accelerated by freshly exposed, thus highly active Mg^0^ in this electrolyte. Spatially homogeneous plating and stripping reactions were also evident from the operando observations. Notably, plating and stripping in the Mg[TfO]_2_‐based electrolyte were always accompanied by continuous bubbling. This finding implies a strong correlation between gas evolution and the uniformity of Mg plating and stripping, because the cells employing the Mg[Al(Ohfip)_4_]_2_‐based electrolytes with and without MOEA—where little or no bubbling was observed during repeated plating/stripping cycles—exhibited uneven utilization of the Mg electrodes (Video 1 and Video S1). The critical role of reactive amine protons in alkylamines in influencing Mg plating and stripping has been experimentally supported in previous studies [22, 23, 36]. The observed results indicate that either the amine protons of MOEA or the potentially present Mg‐coordinated trace water exhibit greater reactivity in the Mg[TfO]_2_‐based electrolyte, and this enhanced reactivity promotes the formation of a functional interfacial layer. In contrast, such reactivity appears to be suppressed in the Mg[Al(Ohfip)_4_]_2_‐based electrolyte.

It has been reported that replacing the moderately coordinating [TFSA]^−^ into strongly coordinating [TfO]^−^ anions in amine‐based electrolytes can weaken the H_2_ evolution through ‐NH_2_ reduction, and this effectively enhances the stability of the Mg‐electrolyte interface [25]. The recent combined theoretical and spectroscopic studies on the Mg salt‐ether‐amine systems, however, suggested the dominant role of free or loosely‐bound amine solvents for inducing H_2_ gas evolution and associated MgH_2_ formation to stabilize the interface [34, 37]. A competition in coordination to Mg^2+^ among amine, ether, and counterions seems important to stabilize the interface by amine decomposition, as the single amine solvent systems, where the dominant species in electrolyte solutions should be Mg‐amine solvates, exhibited substantially poor performance compared to the corresponding dual amine‐ether solvent systems. The present study also points out that the combined presence of water and amine with the strongly coordinating [TfO]^−^ anions would drive gas evolution (vide infra). As the present and preceding works draw somewhat different conclusions with respect to the mechanisms of H_2_ gas evolution and stabilization of the Mg‐electrolyte interface, and more importantly, water contents of respective electrolyte solutions are unclear, further detailed compositional and mechanistic studies with an aid of computational approaches combined with advanced operando characterization techniques are necessary to fully understand the interfacial chemistry in Mg‐amine systems.

The importance of reactive amine protons or the trace‐water‐based reactivity in the presence of amines—rather than merely the interfacial composition—toward electrochemical Mg plating and stripping was also supported experimentally. Indeed, no significant improvement in plating/stripping performance was observed for the MOEA‐free Mg[Al(Ohfip)_4_]_2_/G2 electrolyte, even when Mg electrodes previously cycled in the Mg[TfO]_2_‐based electrolyte were used for the subsequent measurements (Figure S9). As the amine‐trace water proton‐driven surface reaction consumes active amine molecules in electrolyte solutions [43], the cycle life of the cells is dominated by the amount of electrolyte. To achieve the sufficiently stable cycling of Mg electrodes, a statically stable interface is necessary to be developed.

### 2.2 Solvation State and Interfacial Chemistry

The distinct solvation environments of G2 and MOEA in Mg[Al(Ohfip)_4_]_2_/G2‐MOEA and Mg[TfO]_2_/G2‐MOEA electrolytes were investigated by ^1^H NMR spectroscopy (Figure S10). The amine proton (─NH_2_) resonance at 1.2 ppm showed only slight upfield shift upon mixing with G2, indicating weak G2–MOEA interactions. Downfield shifts of the ─NH_2_, methoxide, and methylene groups confirm MOEA–Mg^2+^ interactions that differ by salt. In the Mg[Al(Ohfip)_4_]_2_‐based system, ether oxygen (methoxide) atoms of MOEA bind stronger to Mg^2+^ (─NH_2_ downfield shift: 0.6 ppm), while in Mg[TfO]_2_, the larger 1.3 ppm ─NH_2_ shift indicates ─N(H)‐*H*⋯X H‐bonding presumably involving [TfO]^−^ oxygen atoms, confirming MOEA participation in Mg^2+^ solvation via anion interaction, consistent with earlier MD simulations [34]. Single‐solvent MOEA spectra (Figure S11) show analogous trends. The small G2 chemical shift changes in the Mg[Al(Ohfip)_4_]_2_‐based electrolyte indicate minor G2 participation in Mg^2+^ complexation within fast exchange equilibria, consistent with the absence of non‐coalescent solvent signals on the time scale of NMR measurements.

In the WCA‐based electrolytes, Mg^2+^ is preferentially solvated by solvent molecules due to the low associativity of WCAs. Small (C─)H chemical shift changes for both G2 and MOEA confirm dual‐solvent participation in Mg^2+^ solvation. Without strong H‐bonding to [Al(Ohfip)_4_]^−^, the 0.6 ppm ─NH_2_ low‐field shift identifies MOEA as the preferred Mg^2+^ ligand via NH_2_ coordination, but still being in rapid equilibrium with G2. By contrast, strong Mg^2+^–[TfO]^−^ association (confirmed by Walden plots from conductivity and viscosity measurements, Figure S12) shields Mg^2+^ and likely excludes G2 coordination in the Mg[TfO]_2_‐based electrolyte, consistent with unchanged ^1^H NMR signals of G2. As a known H‐bond acceptor (HBA) [44], [TfO]^−^ attracts the positively polarized amine protons of Mg^2+^‐coordinated MOEA through H‐bonding, as further investigated by Raman spectroscopy.

Raman analysis of the Mg[TfO]_2_/G2‐MOEA electrolyte (Figure S13) focused on the CF_3_ symmetric bending mode δ_s_(CF_3_), sensitive to ion‐pair formation [45, 46, 47]. Voigt deconvolution yielded two modes at 767 and 761 cm^−1^, with no signal in the 757–740 cm^−1^ range, confirming the absence of any “free”, non‐coordinated [TfO]^−^ (reference: 752 cm^−1^ for [N(Bu)_4_]^+^ salt [48]). The blueshifted 767 cm^−1^ mode corresponds to strongly associated [TfO]^−^, and the 761 cm^−1^ mode—consistent with hydrogen‐bonded [TfO]^−^ near 762 cm^−1^ in dialkylimidazolium systems [47]—supports HBA action of [TfO]^−^ and corroborates (H)N‐H⋯O‐S H‐bonding between [TfO]^−^ and the NH_2_ group of MOEA in the [Mg(MOEA)*_n_*]^2+^ complex.

HF‐DFT calculations at the RI‐B3LYP(D3BJ)/def2‐TZVPP level with COSMO implicit solvation (*ε*_r_ = 7.23 for G2) [49] and extra explicit solvent molecules were performed to support the spectroscopic findings. Thermodynamic values are reported in solution (Δ_r_ *G*° at 298 K; all deposited in §2.1 ESI). For the Mg[Al(Ohfip)_4_]_2_/G2‐MOEA system (evaluated to always exhibit coordination number 6), the global minimum is [Mg(MOEA)_3_]^2+^; [Mg(G2)_2_]^2+^ lies +11 kJ mol^−1^ higher. With Δ_r_ *G*°(solution) = –*RT* ln K, the equilibrium constant K is 10^−1.93^, implying only 1 out of 85 Mg^2+^ ions takes up G2 at equilibrium. For the Mg[TfO]_2_/G2‐MOEA system, the solvation structures of which were previously elucidated [25, 34], our calculations agree with prior work: the tight ion pair [Mg(MOEA)_2_(TfO)_2_] is the dominant species, and all G2‐containing structures are much higher in energy (Table S2). Conversion to the free [Mg(MOEA)_3_]^2+^ ion (which dominates in the Mg[Al(Ohfip)_4_]_2_/G2‐MOEA electrolyte) costs ca. 170 kJ mol^−1^ in Gibbs energy, confirming that [TfO]^−^ ions persist in the Mg^2+^ environment, primarily as the tight ion pair [Mg(MOEA)_2_(TfO)_2_] and, as a very minor component, H‐bonded [Mg(MOEA)_3_(TfO)_2_], that is ca. 40 kJ mol^−1^ higher in energy.

To elucidate the impact of the solvation state on the interfacial characteristics of the Mg[Al(Ohfip)_4_]_2_ and Mg[TfO]_2_‐based electrolytes, comparative studies were conducted. Figure 3 presents SEM images of Mg metal soaked in these two electrolytes for 48 h. Evidently, the Mg surface was damaged due to reactions with the alkoxyalkylamine‐integrated electrolyte solutions, whereas no significant change in surface morphology was observed for Mg metal soaked in the MOEA‐free Mg[Al(Ohfip)_4_]_2_/G2 electrolyte (Figure 3 and Figure S14). Subsequent X‐ray photoelectron spectroscopy (XPS) analysis of the soaked Mg metals, however, revealed distinct reaction mechanisms between the Mg[Al(Ohfip)_4_]_2_‐ and Mg[TfO]_2_‐based electrolytes. For the Mg[Al(Ohfip)_4_]_2_‐based electrolyte, the metallic Mg surface is well exposed, and the residual surface composition was dominated by oxygen‐based compounds such as MgO and Mg(OH)_2_ [50]. The larger relative content of Mg(0) against Mg‐oxygen compounds and the negligible contribution of nitrogen‐ and fluorine‐containing species to the surface composition strongly suggests that the native oxygen‐based surface layer was removed through an etching effect, while the MOEA solvent and the [Al(Ohfip)_4_]^−^ anion do not likely decompose during this etching process (vide infra for its mechanism). The etching behavior of the Mg[Al(Ohfip)_4_]_2_‐based electrolyte is further supported by the exceptionally small interfacial resistance (Figure 1d). In contrast, for the Mg[TfO]_2_‐based system, various elements including fluorine, nitrogen, and oxygen significantly contributed to the formation of a more complex surface layer. Notably, this layer consisted of both organic (C‐NH_2_ and CF_3_) and inorganic (MgF_2_, Mg(NO*_x_*)_2_, and Mg(OH)_2_) species [13, 50]. These observations clearly indicate that both the MOEA solvent and the [TfO]^−^ anion participated in the surface reactions. The formation of a hybrid organic–inorganic interface through preferential decomposition of the MOEA‐coordinated Mg^2+^ complex, accompanied by [TfO]^−^ decomposition, has also been reported in previous studies [25, 34]. The resulting hybrid interface, enriched with relatively insulating components, is responsible for the substantially larger interfacial resistance observed for Mg metal in the Mg[TfO]_2_/G2‐MOEA electrolyte (Figure 1d).

**FIGURE 3:**
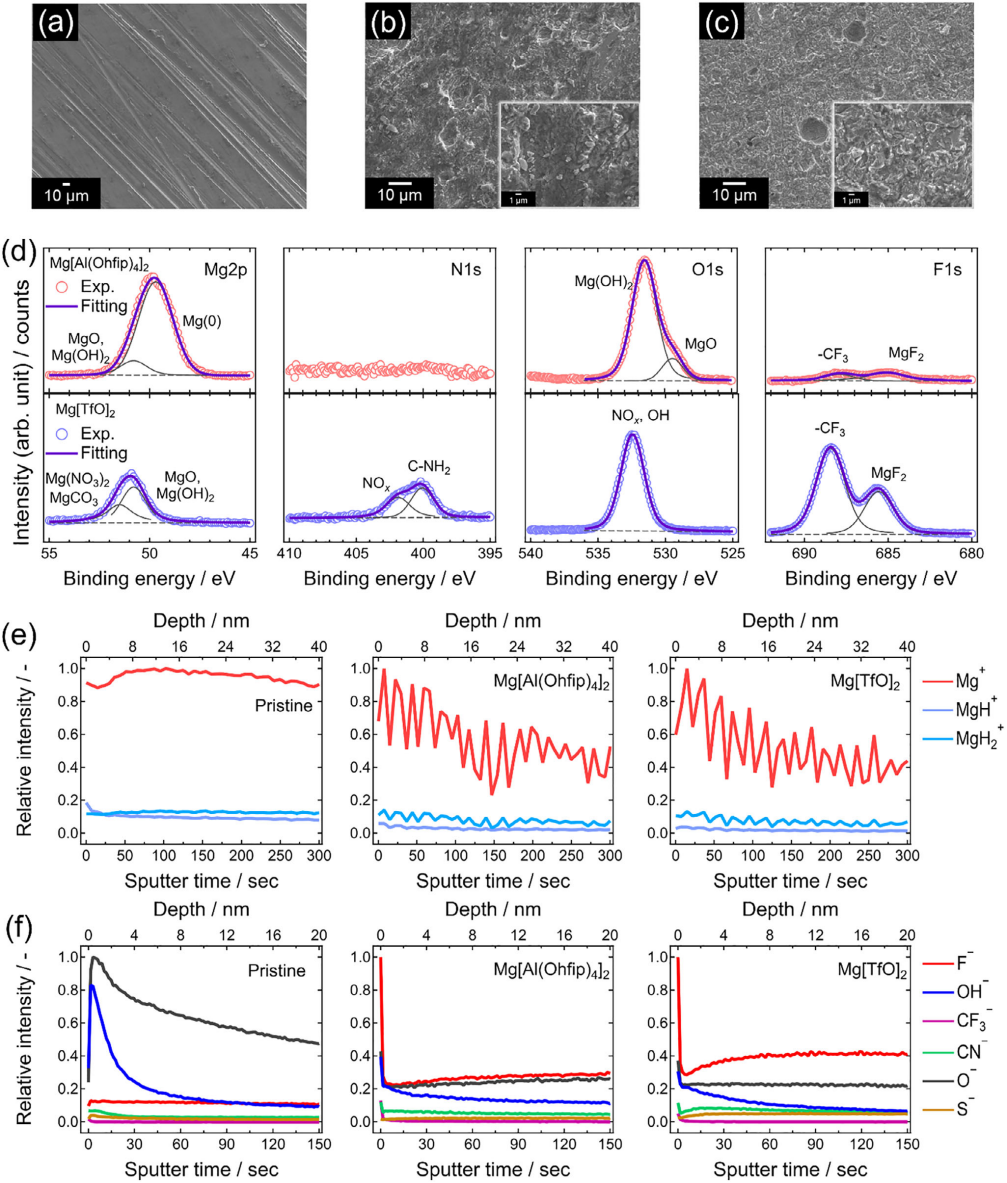
Surface SEM images of (a) pristine Mg and Mg metals soaked in (b) Mg[Al(Ohfip)_4_]_2_/G2‐MOEA and (c) Mg[TfO]_2_/G2‐MOEA. (d) Mg 2p, N 1s, O 1s, and F 1s (left to right) XPS spectra of Mg electrodes soaked in (upper) Mg[Al(Ohfip)_4_]_2_/G2‐MOEA and (lower) Mg[TfO]_2_/G2‐MOEA. Depth‐profiled SIMS spectra of pristine Mg and Mg metals soaked in the two electrolytes were acquired in (e) positive and (f) negative ion modes. The samples used for these characterizations were prepared by soaking mechanically polished Mg metals in the respective electrolytes for 48 h at 30°C.

In addition to these conventional interfacial components, metal hydrides originating from parasitic interfacial reactions have recently been identified as functional agents in Mg plating and stripping processes [22, 23, 36, 37]. Because hydrogen ions are difficult to detect by XPS, time‐of‐flight secondary ion mass spectrometry (TOF‐SIMS) analysis was conducted on the same Mg metal samples used for the XPS measurements. Figure 3e,f presents the depth profiles of TOF‐SIMS spectra obtained in the positive and negative ion modes, respectively. The positive‐mode profiles indicate a minimal contribution of metal hydrides to the surface layer composition. Hydride species were detected even in pristine Mg metal, and their relative intensities remained almost unchanged across the Mg samples soaked in different electrolytes. Taking a closer look at the SIMS profiles, particularly at the outermost surface (sputter time = 0 s), the mass signals assignable to MgH^+^ appear slightly higher for the sample soaked in Mg[Al(Ohfip)_4_]_2_/G2‐MOEA than for those soaked in Mg[TfO]_2_/G2‐MOEA. However, such a minor difference is insufficient to account for the substantially larger disparity in the interfacial characteristics of these two electrolytes. In contrast to the positive‐ion mode, the negative‐ion mode profiles clearly revealed compositionally distinct interlayer formations on the Mg surfaces soaked in the Mg[Al(Ohfip)_4_]_2_‐ and Mg[TfO]_2_‐based electrolytes. The surface of the Mg metal soaked in the Mg[Al(Ohfip)_4_]_2_‐based electrolyte was enriched with oxygen‐containing compounds, whereas significant contributions from fluorine‐ and nitrogen‐containing species were observed in the sample derived from the Mg[TfO]_2_‐based electrolyte. Notably, substantially larger contributions of CN^−^ and S^−^ were discernible for the Mg[TfO]_2_/G2‐MOEA electrolyte (Figure 3f). These findings are in good agreement with the results of the XPS analysis (Figure 3d). Considering the electrochemical and analytical results together, it can be concluded that the nitrogen‐ and fluorine‐containing interfacial components derived from MOEA and [TfO]^−^ play a crucial role in stabilizing the Mg/electrolyte interface by suppressing further parasitic reactions during Mg plating and stripping cycles.

The distinct compositions of the Mg–electrolyte interfaces in Mg[Al(Ohfip)_4_]_2_/G2‐MOEA and Mg[TfO]_2_/G2‐MOEA systems can thus be rationalized in terms of their respective solution states. It is well recognized, particularly in RMMB research, that species coordinated to Mg^2+^ become highly susceptible to reduction because of the strong electric field exerted by Mg^2+^ and the resulting large shift in the electron affinity of the coordinating species [20, 51, 52]. In Mg[Al(Ohfip)_4_]_2_/G2‐MOEA, the theoretical calculations suggest [Mg(MOEA)_3_]^2+^ to be the dominant species. Upon electronation, the Löwdin population analysis of the vertical radical cation emphasizes that the [Mg(MOEA)_3_]^·+^ ion has a coefficient directly at the Mg atom (see §2.2 ESI). As the higher spin density on Mg and the NH_2_ groups stabilize the electron density through effective spin‐delocalization – similar to the high stability of even free solvated electrons in solutions of alkaline or alkaline earth metals in ethylene diamine H_2_N‐CH_2_CH_2_‐NH_2_ [53, 54], a [Mg(MOEA)_3_]^·+^ radical cation yields Mg‐metal presumably directly plated on the interface with almost no decomposition products upon reduction at the Mg interface. This agrees with the absence of any nitrogen signature in the XPS analysis (Figure 3d). Because [Al(Ohfip)_4_]^−^ remains outside the primary solvation sheath of Mg^2+^, it retains its intrinsic reductive stability in solution. Yet, if the highly polarized coordinated G2 molecules in the [Mg(G2)_2_]^2+^ ion available in equilibrium accept electrons from the reductive Mg metal surface at the interface, the electrons taken up by the system are much higher in energy, thus more reactive and likely induce ligand‐degradation. Consequently, the surface of Mg metal in contact with Mg[Al(Ohfip)_4_]_2_/G2‐MOEA slowly and over time becomes enriched with G2‐derived oxygen‐containing compounds, as evidenced by the XPS spectra (Figure 3d). In contrast, [TfO]^−^ participates strongly in the solvation of Mg^2+^ and the ‐NH_2_ group of MOEA is further bound to [TfO]^−^ via H‐bonding in the Mg[TfO]_2_‐based electrolyte. Such a multi‐compositional solvation structure promotes the decomposition of both [TfO]^−^ and MOEA at the Mg metal surface, leading to the formation of the observed fluorine‐ and nitrogen‐rich functional interface.

### 2.3 MOEA‐Mediated Partial Hydroxylation of [Al(Ohfip)_4_]^−^ and Understanding of Dynamic Failure Mechanism in Mg[Al(Ohfip)_4_]_2_/G2‐MOEA

During the NMR investigations, a peculiar water‐induced hydroxylation process of the [Al(Ohfip)_4_]^−^ ion was detected. Note that, despite adopting an intensive dehydration protocol to MOEA for this study – i.e., by distillation over CaH_2_ and storage with activated molecular sieves for several days – the water content in our MOEA solvent batches was indirectly determined to spread between several hundred to several thousand ppm based on the NMR analysis, where all products formed were assumed to originate from the reaction of [Al(Ohfip)_4_]^−^ with water remnants in MOEA. To further support this point, we have investigated the water content in the MOEA batch used by a titration protocol in the presence of benzoic acid, that is reported bind all the basic MOEA amine, and only leaves the less basic water unaffected and to be determined [55]. This protocol gave a water content of 156 ppm in MOEA. As the MOEA was mixed with G2 in 1:1 volume ratio in our electrolyte formulations, the water content in Mg[Al(Ohfip)_4_]_2_/G2‐MOEA is assumed to be less than 150 ppm. However, these values are not very trustworthy in our opinion, as they depend much on the titration criteria applied (details of the titration protocol and criteria are given in the ESI). By contrast, water contamination of the Mg[Al(Ohfip)_4_]_2_/G2 electrolyte does not significantly affect Mg plating/stripping performance and the [Al(Ohfip)_4_]^−^ anion tolerates even 1000 ppm water content with no visible byproduct as shown by NMR [18]. Since this work showed the somewhat moist Mg[Al(Ohfip)_4_]_2_/G2‐MOEA electrolyte to support Mg plating and stripping (Figures 1 and 2), its water content seems to lie still below the direct hydrolysis threshold of the [Al(Ohfip)_4_]^−^ anion [18, 27]. Therefore, MOEA apparently plays a crucial role in enabling the hydroxylation of [Al(Ohfip)_4_]^−^.

With this background, the stability of the [Al(Ohfip)_4_]^−^ ion in the presence of water and amine was investigated in solution. As shown in Figure 4a, Mg[Al(Ohfip)_4_]_2_ in G2 exhibits a characteristic septet ^1^H resonance at 4.5 ppm, which is assignable to the α‐proton of the O‐C(*H*)(CF_3_)_2_‐group. However, an additional downfield resonance appears in the G2–MOEA solution that is assigned to a reaction product with [Al(Ohfip)_4_]^−^. The corresponding ^19^F NMR spectrum confirms the formation of another Ohfip‐compound and the sharp ^27^Al resonance arising from the tetracoordinated environment (Al_CN4_) in Mg[Al(Ohfip)_4_]_2_/G2 is now flanked by an additional broader signal at approximately 30 ppm in the presence of MOEA/trace water. The shift position at 30 ppm agrees with a penta‐coordinated structure [56]. For example, the trigonal bipyramidal aluminate anion [F_2_Al(Ohfip)_3_]^2−^, has a distinct ^27^Al NMR signal at 33.7 ppm [57] (replace fluoride by isosteric hydroxide). Also a dimeric [(hfipO)_3_Al‐(µ‐F)_2_‐Al(Ohfip)_3_]^2−^ is known [57], unfortunately without NMR shift. If one again replaces F for OH, this could suggest dimerization of an initial tetracoordinate [Al(Ohfip)_3_(OH)]^−^ (cf. the known [58], bulkier [Al(OC(CF_3_)_3_)_3_(OH)]^−^) to give the pentacoordinate (Al_CN5_) [(hfipO)_3_Al‐(µ‐OH)_2_‐Al(Ohfip)_3_]^2−^ dianion. From diffusion‐ordered NMR spectroscopy measurements (data not shown), the formation of a large complex anion, diffusing slower than monomeric [Al(Ohfip)_4_]^−^, agrees with the assignment of dimeric [(hfipO)_3_Al‐(µ‐OH)_2_‐Al(Ohfip)_3_]^2−^, (as its Mg^2+^ complex?), as the pentacoordinate aluminate in solution. Also partial hydrolysis of the parent Mg[Al(Ohfip)_4_]_2_∙3G1 adducts is known to yield a binuclear aluminate anion [59]. In addition, solvent coordination may induce formation of penta‐coordinated Al species [60].

**FIGURE 4:**
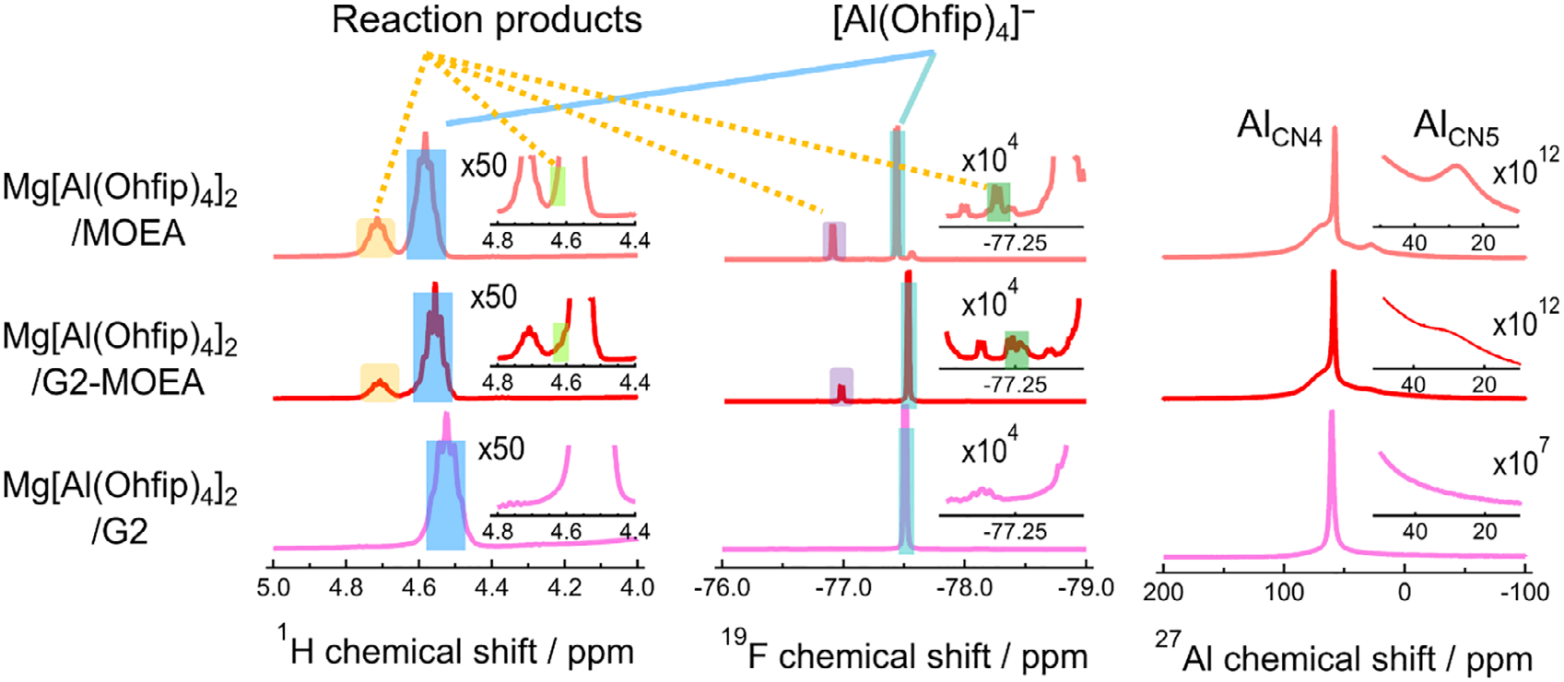
^1^H, ^19^F, and ^27^Al NMR (25°C; either 400.17, 376.54, and 104.27 MHz or 300.18, 282.45, and 78.22 MHz, respectively) spectra of Mg[Al(Ohfip)_4_]_2_‐based electrolytes. Selected spectral regions corresponding to [Al(Ohfip)_4_]^−^ are highlighted. Al_CN4_ and Al_CN5_ denote Al species with a coordination number of 4 and 5, respectively. The intensive broad resonance between 0 and 100 ppm in the ^27^Al‐NMR spectrum is a background signal from the probehead, whereas the small broad shoulder at 30 ppm is a signal from the sample.

The relative intensities of the reaction products – the Ohfip‐compound and the penta‐coordinate Al species – are higher in the single MOEA‐solvent system than in the mixed G2–MOEA solvent system (Figure 4), confirming that the reaction with [Al(Ohfip)_4_]^−^ is significantly promoted by MOEA. Nevertheless, quantitative analysis of the relevant signal areas indicates that only about 20% of [Al(Ohfip)_4_]^−^ undergoes reaction. Even an intentionally added far excess of water (ca. 0.5 mol dm^−3^!), which would have been enough to react with all [Al(Ohfip)_4_]^−^ in the electrolyte, shows that only about 20% of [Al(Ohfip)_4_]^−^ undergoes reaction (Figure S15) in the Mg[Al(Ohfip)_4_]_2_/G2‐MOEA solutions. Moreover, the reaction reaches completion immediately after sample preparation, as aging has no discernible effect on the relative composition of [Al(Ohfip)_4_]^−^ and its reaction products (cf. identical NMR spectra of as‐prepared and aged samples; Figure S16). These observations suggest a peculiar reaction mechanism driven by the water‐contaminated MOEA and [Al(Ohfip)_4_]^−^.

Further Fourier transform attenuated total reflection infrared (FT‐ATR‐IR) spectroscopy measurements of the electrolyte shown in Figure S17 verify a collective large redshift of the N‐H vibrational modes in the Mg[Al(Ohfip)_4_]_2_/G2‐MOEA system. This strongly suggests protonation of MOEA resulting in strong H‐bonding visible by IR. Hence, also protonated MOEA ([H‐MOEA]^+^) appears to be relevant as part of this reaction.

To identify the underlying cause of the undesired water‐induced reaction of [Al(Ohfip)_4_]^−^, the Gibbs free reaction energies (Δ_r_ *G*°) of possible reaction products were DFT calculated in G2 solution as described above [49]. Since MOEA is chemically a base (cf. p*K*_a_ of its protonated form in water: 9.40 [61]), it may react in an acid‐base equilibrium with water to yield hydroxide (reaction (1), with hydration of the cation being considered). The nucleophilic hydroxide ion formed in (1) could then attack the electrophilic Al‐atom of the [Al(Ohfip)_4_]^−^ anion (2):

(1) $$\begin{array}{ccc} {\text{MOEA}}_{\left(\mathrm{solv}.\right)}+2H_{2}O_{\left(\mathrm{solv}.\right)} & ⇄ & {\left[H_{2}O\cdots H-\mathrm{MOEA}\right]}_{\left(\mathrm{solv}.\right)}^{+}\\ & & +\;{{\mathrm{HO}}^{-}}_{\left(\mathrm{solv}.\right)}\;\Delta_{r}G^{\circ }\left(1\right)\\ & = & +6\;{\mathrm{kJ}\;\mathrm{mol}}^{-1} \end{array}$$

(2) $$\begin{array}{ccc} {{\mathrm{HO}}^{-}}_{\left(\mathrm{solv}.\right)}+{{\left[\mathrm{Al}{\left(\mathrm{Ohfip}\right)}_{4}\right]}^{-}}_{\left(\mathrm{solv}.\right)} & \to & {{\left[\mathrm{Al}{\left(\mathrm{Ohfip}\right)}_{4}\mathrm{OH}\right]}^{2-}}_{\left(\mathrm{solv}.\right)}\\ \Delta_{r}G^{\circ }\left(2\right) & = & -62\;{\mathrm{kJ}\;\mathrm{mol}}^{-1} \end{array}$$

Δ_r_ *G*°(1) implies equilibrium, and at standard conditions only one out of 10 molecules on the left reacts to the right. The solvated hydroxide ions thereby formed, immediately react with the [Al(Ohfip)_4_]^−^ anion according to (2) and give the dianion [Al(Ohfip)_4_OH]^2−^. Yet, this structure is not stable and easily loses solvated [Ohfip]^−^ according to (3) as the more stable anion (cf. p*K*_a_ values: HO‐hfip is the stronger acid than water [62]).

(3) $$\begin{array}{ccc} {{\left[\mathrm{Al}{\left(\mathrm{Ohfip}\right)}_{4}\mathrm{OH}\right]}^{2-}}_{\left(\mathrm{solv}.\right)}\; & \to & \;{{[\mathrm{Al}{\left(\mathrm{Ohfip}\right)}_{3}\mathrm{OH}]}^{-}}_{\left(\mathrm{solv}.\right)}\;+\;{{\left[\mathrm{Ohfip}\right]}^{-}}_{\left(\mathrm{solv}.\right)}\\ \Delta_{r}G^{\circ }(3)\; & = & -46\;\mathrm{kJ}\;\;\mathrm{mo}l^{-1} \end{array}$$

With 0.3 m solvated Mg^2+^ ions in the liquid electrolyte close by, the solvated [Ohfip]^−^ anion will immediately undergo ion‐pairing as in (4) to give the known [63] compound Mg(Ohfip)_2_:

(4)

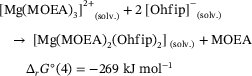




The solvated [Al(Ohfip)_3_OH]^−^ ion formed in Equation (3) will likely be in equilibrium with dimeric forms (5), e.g., mediated by ion‐pairing with the Mg^2+^ ion in (6):

(5) $$\begin{array}{ccc} & & {{\left[\mathrm{Al}{\left(\mathrm{Ohfip}\right)}_{3}\mathrm{OH}\right]}^{-}}_{\left(\mathrm{solv}.\right)}\;\\ & & \;⇄\;{{\left[{(\mathrm{hfipO})}_{3}\mathrm{Al}{\left(\mu -\mathrm{OH}\right)}_{2}\mathrm{Al}{\left(\mathrm{Ohfip}\right)}_{3}\right]}^{2-}}_{\left(\mathrm{solv}.\right)}\\ & & \;\Delta_{r}G^{\circ }\left(5\right)\;=\;+44\;\mathrm{kJ}\;\mathrm{mo}l^{-1} \end{array}$$

(6) $$\begin{array}{ccc} & & {{\left[{\left(\mathrm{hfipO}\right)}_{3}\mathrm{Al}{\left(\mu -\mathrm{OH}\right)}_{2}\mathrm{Al}{\left(\mathrm{Ohfip}\right)}_{3}\right]}^{2-}}_{(\mathrm{solv}.)\;}\\ & & +{{\left[\mathrm{Mg}{\left(\mathrm{MOEA}\right)}_{3}\right]}^{2+}}_{\left(\mathrm{solv}.\right)}\;\to \;\;{\left[{\left(\mathrm{hfipO}\right)}_{6}Al_{2}{\left(\mathrm{OH}\right)}_{2}\mathrm{Mg}\left(\mathrm{MOEA}\right)\right]}_{(\mathrm{solv}.)\;}\\ & & +\;2\mathrm{MOE}A_{\left(\mathrm{solv}.\right)}\;\Delta_{r}G^{\circ }\left(6\right)=-127\;\mathrm{kJ}\;\mathrm{mo}l^{-1} \end{array}$$

Thermodynamically, the very exergonic reaction (6) will remove the dianion from the left side of the endergonic reaction (5). Overall, the sequence (1) to (6) implies that the pentacoordinate aluminate (Al_CN5_) observed by NMR‐spectroscopy is the Mg^2+^ complex of the dianion [(hfipO)_3_Al(µ‐OH)_2_Al(Ohfip)_3_]^2−^ and the second Ohfip‐containing compound is the long‐known Mg(Ohfip)_2_.

Now we need to address the question as to why the hydroxylation stops at about 20% conversion of [Al(Ohfip)_4_]^−^ to the Mg^2+^ complex of [(hfipO)_3_Al(µ‐OH)_2_Al(Ohfip)_3_]^2−^. Hence, if we start in (1) with 2280 ppm water (=0.12 mol dm^−3^), this should generate with *K*(1) = 0.1 only a small amount of hydroxide (0.006 mol dm^−3^). In a 0.3 mol dm^−3^ electrolyte, the [Al(Ohfip)_4_]^−^ concentration is 0.6 mol dm^−3^. Experimentally, we have seen the hydroxylation of 20% of [Al(Ohfip)_4_]^−^, that would correspond to 0.12 mol dm^−3^. With the exergonic reactions (2) (−62 kJ mol^−1^) and the [Ohfip]^−^ scavenging by Mg^2+^ in (4) (−269 kJ mol^−1^), the solvated HO^−^_(solv.)_ is withdrawn from (1) and yields the hydroxylated aluminate dianion. Apparently, and from the experiments described above, the formation of the hydroxylated aluminate dianion levels off at 0.12 mol dm^−3^ concentration. This may occur, when either all water is consumed or the reaction levels off due to the strong increase of the concentration of the cation [H_2_O∙∙∙H‐MEOA]^+^ that has to form concomitantly with hydroxide in (1) and which increases the chemical potential in the system and stops further hydroxylation.

The analysis of the hydroxylation of the [Al(Ohfip)_4_]^−^ anion in the G2‐MOEA system revealed that hydroxide ions are present in the electrolyte system. A broad evaluation of the structure and energetics of solvated [Mg(OH)]^+^ or [Mg(OH)_2_] species was performed, that also did include water complexes of Mg compounds. Out of the investigation, the four ions shown on the top of Scheme 1 below were found to be relevant in the Mg[Al(Ohfip)_4_]_2_/MOEA solution; all others were too high in energy and hence not present in equilibrium. In the subsequent reactions, shown below at the Mg oxide‐hydroxide interphase, now a surface Mg(OH)_2_ may be solubilized by one reactive [Mg(MOEA)_3_(OH_2_)]^2+^ ion in the form of two solvated [Mg(OH)]^+^ complexes via the ammonium‐assisted solubilization mechanism [64] – one emerging from the solution and one emerging from the surface‐species. Hence, the high available concentration of [Mg(MOEA)_3_]^2+^ ions quickly removes the surface Mg(OH)_2_ interphase as observed in the experiment. A substantial stability of amines against an electron also renders absence of MOEA decomposition during etching process very likely (cf. Scheme S5) [54, 65, 66].

**SCHEME 1:**
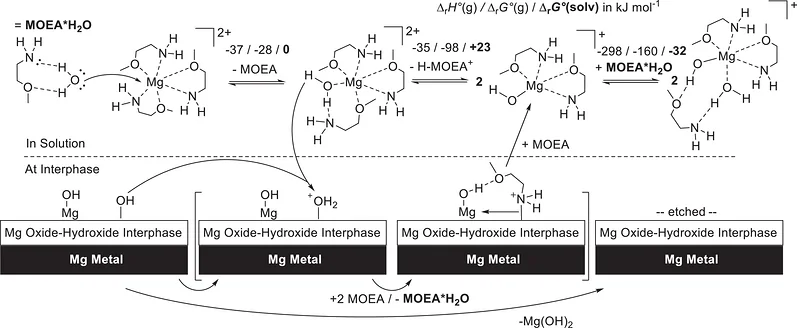
Top, solution part: the four ions shown to be relevant for the solution chemistry; all others were too high in energy and hence not present in equilibrium. Bottom: Reaction at the Mg oxide‐hydroxide interphase. The rather reactive [Mg(MOEA)_3_(OH_2_)]^2+^ ion contains an (acidified, positively polarized) water molecule that may protonate a surface Mg‐OH and thereby induce dissolution and etching of surface Mg(OH)_2_ in the subsequent reaction sequence. The square brackets for the two middle species at the interphase indicate short‐lived intermediates. Note that the entire scheme is stoichiometrically balanced.

The main difference between the [Al(Ohfip)_4_]^−^ and the [TfO]^−^ electrolytes is the majority species in solution. While in the [Al(Ohfip)_4_]^−^‐based, the reactive [Mg(MOEA)_3_]^2+^ ion is the majority species, in the [TfO]^−^ electrolyte the ion‐paired and H‐bonded solvated [Mg(TfO)_2_] is the majority ion. As mentioned earlier, removal of the coordinated [TfO]^−^ ions to give the [Mg(MOEA)_3_]^2+^ ion as found in the [Al(Ohfip)_4_]^−^ electrolyte, is a highly endergonic process. Hence, one expects the solvated [Mg(TfO)_2_] to be too unreactive, to activate and to etch the Mg oxide‐hydroxide interphase. Moreover, [TfO]^−^ is well known for its stability against hydrolysis [67]. The ^19^F NMR spectra of the Mg[TfO]_2_‐based electrolytes clearly exclude the possibility of [TfO]^−^ decomposition induced by trace water (Figure S18). All investigations into the structure and energetics of this process, the formation of water or hydroxide complexes, gave high energy species with the exception of one water adduct, but absolutely no hydroxide complex. These are at least 128 kJ mol^−1^ endergonic and not available for any surface modification. Overall, the Mg‐coordination of the [TfO]^−^ ion in the electrolytes lowers the reactivity so strongly that it can no longer serve to clean the surface. For full details, see the deposited sequence of reactions assessed in the §2.3 ESI. The presence of an appreciable amount of hydroxide ions in moist amine solvents also accounts for the relatively inferior Mg plating/stripping performance of the Mg[TFSA]_2_‐based amine‐integrated systems compared to the Mg[TfO]_2_‐based [22, 25], due to the limited stability of [TFSA]^−^ under highly basic conditions [68].

Overall, the dynamic failure mechanism of the WCA‐system is most likely driven by the MOEA‐mediated and trace‐water‐driven hydroxylation of [Al(Ohfip)_4_]^−^, followed by inhomogeneous passivation and eventual localized utilization. Observation of negligible signals of fluorine‐ and nitrogen‐containing species in the XPS spectra of the Mg metal soaked in the Mg[Al(Ohfip)_4_]_2_/G2‐MOEA (Figure 3d) implies that the hydroxylation reaction byproducts, the Mg^2+^ complex with the dianion [(hfipO)_6_Al_2_(OH)_2_Mg(MOEA)] and [Mg(MOEA)_2_(Ohfip)_2_], might be stable against initial, etched Mg metal. The electrochemical processes, however, promote decomposition of the accumulated byproducts on fresh Mg surfaces exposed through electrochemistry, eventually leading to the resistive interlayer formation. The suggestion of electrochemistry‐promoted decomposition of byproducts is in good agreement with the XPS spectra of the cycled Mg electrodes, where the surface composition was rich in nitrogen and fluorine species (Figure S19). A relatively thick layer of highly amorphous decomposition products for the cycled Mg electrodes in Mg[Al(Ohfip)_4_]_2_/G2‐MOEA also supports the instability of the byproducts against a fresh Mg surface (Figure 2c–g). Moreover, as the stripping of bulk metal preferentially takes place at the dislocation and/or grain boundary [69, 70, 71], the resulting resistive layer would be spatially inhomogeneous. The spatial inhomogeneity in the surface resistance will cause the uneven, localized utilization of Mg metal, ultimately leading to short‐circuiting events at the early stage of cycling. These sequential interfacial processes are likely responsible to the complicated surface chemistry and particularly inferior electrochemical performance in the Mg[Al(Ohfip)_4_]_2_/G2‐MOEA electrolyte (Figure 1a). Note that the equilibrium products between MOEA and H_2_O initiate the above dynamic failure in composition, interface, and consequent electrochemistry. As the dynamic desolvation process at the interface does not directly correlate to the above interfacial decomposition processes, current densities for the galvanostatic cycling measurements have a minor impact on the long‐term cycling stability (Figure S20). On the other hand, the failure of Mg plating/stripping in Mg[B(Ohfip)_4_]_2_/G2‐MOEA (Figure S5) would likely be induced by different mechanisms as the *sp*3 boron center is not capable to accept additional coordination by OH^−^ due to its elemental characteristics. The ^1^H, ^11^B, and ^19^F NMR spectra indeed corroborated the substantial stability of [B(Ohfip)_4_]^−^ even in the presence of hydroxide ions (Figure S21). As [B(Ohfip)_4_]^−^ gradually undergoes hydrolysis by water contamination [72], the decomposition of [B(Ohfip)_4_]^−^ causes impedance growth at a later stage and a short eventually.

It should be emphasized here that the above scenario, especially in the Mg[Al(Ohfip)_4_]_2_/G2‐MOEA electrolyte, are evidently induced by water‐contaminated MOEA; hence, a completely different picture in solution and at the interface can be anticipated for the water‐free system. Nevertheless, due to the intrinsic characteristics of primary and secondary amines to form H‐bonding with water molecules, complete dehydration of MOEA is unfeasible in practical as shown also by our rigorous drying conditions that could not remove the trace water to the desired low ppm level. Moreover, water contamination from other battery components, such as composite positive electrodes, separators, negative electrodes, tub, and casing, is inevitable in practical battery fabrication from a process perspective. Furthermore, side‐reactions during battery operation can also potentially result in the generation of water molecules in the end [73, 74]. Since hydroxylation of [Al(Ohfip)_4_]^−^ is thermodynamically favorable, the following fluctuation of the Mg‐electrolyte interface is also unavoidable once the electrolyte gets water contamination through various undesired events. Therefore, although investigation of the water‐free systems will give distinct and valuable insights into the compositional and interfacial chemistries, electrolytes based on hydroxide‐accepting WCAs with amine solvents are somehow inappropriate to practical battery applications and may reveal surprises with the capacity to donate hydroxide ions along the acid‐base equilibrium. Hence, such failure mechanism – reported here for the first time for battery electrolytes – need to be considered in RMMB and all other battery systems, given that Brønsted basic solvents with amine functions are used.

### 2.4 Full‐Cell Performance and Complex Dilemma

As demonstrated above, no synergistic effect was observed from combining the two concepts—WCA‐based Mg salts and alkoxyalkylamines—in RMMB electrolytes. Alkoxyalkylamines preferentially coordinate with coordinating and H‐bonding anion‐based Mg salts such as Mg[TfO]_2_, leading to excellent electrochemical Mg plating/stripping performance in the Mg[TfO]_2_/G2‐MOEA electrolyte. Although these outstanding characteristics appear promising for practical applications, the reality is less favorable because this electrolyte exhibits serious drawbacks at the positive electrode side. Preliminary discharge–charge tests employing Chevrel phase Mo_6_S_8_ were successful while those employing high‐voltage layered iron vanadate FeV_3_O_9_∙1.1H_2_O (FeVO) failed to exhibit reversible cycling with the Mg[TfO]_2_/G2‐MOEA electrolyte (Figure 5). In contrast, the MOEA‐free simple ethereal electrolytes based on Mg[Al(Ohfip)_4_]_2_ exhibit good compatibility even with FeVO electrodes. The linear sweep voltammograms clearly reveal the inferior anodic stability of amine‐containing electrolytes, regardless of the conductive salt used (Figure S22). A noticeable current increase due to electrolyte oxidation was observed at approximately 2.0 V vs. Mg, which is out of the voltage windows for operating high‐voltage positive electrode materials. Moreover, [TfO]^−^ is known to be highly corrosive toward Al current collectors, even at relatively low potentials [75]. This characteristic was also confirmed in the present study through chronoamperometric measurements and subsequent SEM observations (Figure S22). The intrinsically poor oxidation stability of amine solvents and strong corrosiveness of [TfO]^−^ anions jointly contributed to the failure of cycling with FeVO. These background knowledge of the intrinsic physicochemical characteristics of every Mg[TfO]_2_‐amine system are particularly important in designing high‐performance electrolytes that are compatible with both Mg negative and high‐voltage positive electrodes for the future development.

**FIGURE 5:**
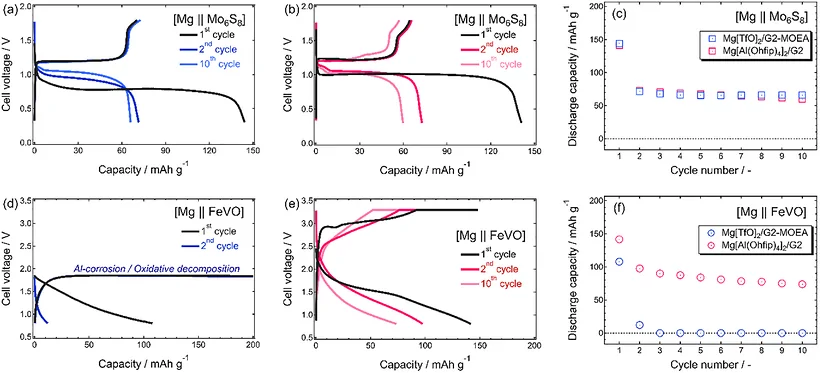
Discharge‐charge profiles of (a, b) [Mg || Mo_6_S_8_] and (d, e) [Mg || FeVO] cells using (a, d) Mg[TfO]_2_/G2‐MOEA and (b, e) Mg[Al(Ohfip)_4_]_2_/G2 as electrolytes. Discharge capacity retention of (c) [Mg || Mo_6_S_8_] and (f) [Mg || FeVO] cells.

Considering all experimental and calculational results collectively, the relationship between compositional and interfacial chemistry is summarized in Figure 6. The mechanistic investigation based on NMR spectroscopy, combined with quantum chemical calculations and vibrational analysis, revealed the MOEA‐mediated and trace‐water‐driven partial hydroxylation of [Al(Ohfip)_4_]^−^ (Figure 6a). The rather reactive [Mg(MOEA)_3_]^2+^ and [Mg(MOEA)_3_(OH_2_)]^2+^ ions present would dissolve the surface Mg(OH)_2_ via the ammonium‐assisted solubilization mechanism [64]. Consumption of water impurity by the partial hydroxylation and [Mg(MOEA)_3_]^2+^‐mediated etching effect may jointly contribute to forming less insulative oxygen‐rich interface, which likely accounts for the exceptionally low interfacial resistance (Figure 1d). However, the hydroxylation of the anion and the resulting complicated cation‐anion complex formation would in turn result at later stages in an unstable interface for the electrochemical Mg plating/stripping reactions (Figure 1a). For the Mg[TfO]_2_‐based electrolyte, Mg^2+^ is coordinated by the strongly associative [TfO]^−^ ion and MOEA in the solutions. The reactivity of trace water molecules is suppressed through H‐bonding with MOEA. The strongly solvated [TfO]^−^ and MOEA species in the primary solvation sheath are prone to reduction at the interface. The mutual cation‐anion, solvent–anion, and solvent–water interactions and the resulting functional interphase may collectively contribute to the stabilization of the interface and remarkable electrochemical performance for the Mg[TfO]_2_/G2‐MOEA electrolyte (Figure 1b). Unfortunately, its limited anodic stability and strong corrosiveness toward the Al current collector in turn hinder its application in practical RMMBs (Figure 6b). Therefore, the development of electrolyte systems still faces an unresolved fundamental dilemma: materials that provide excellent compatibility at one electrode often compromise the performance at the other. Rational, multilateral design strategies are urgently needed to develop high‐performance RMMBs capable of overcoming these complex trade‐offs.

**FIGURE 6:**
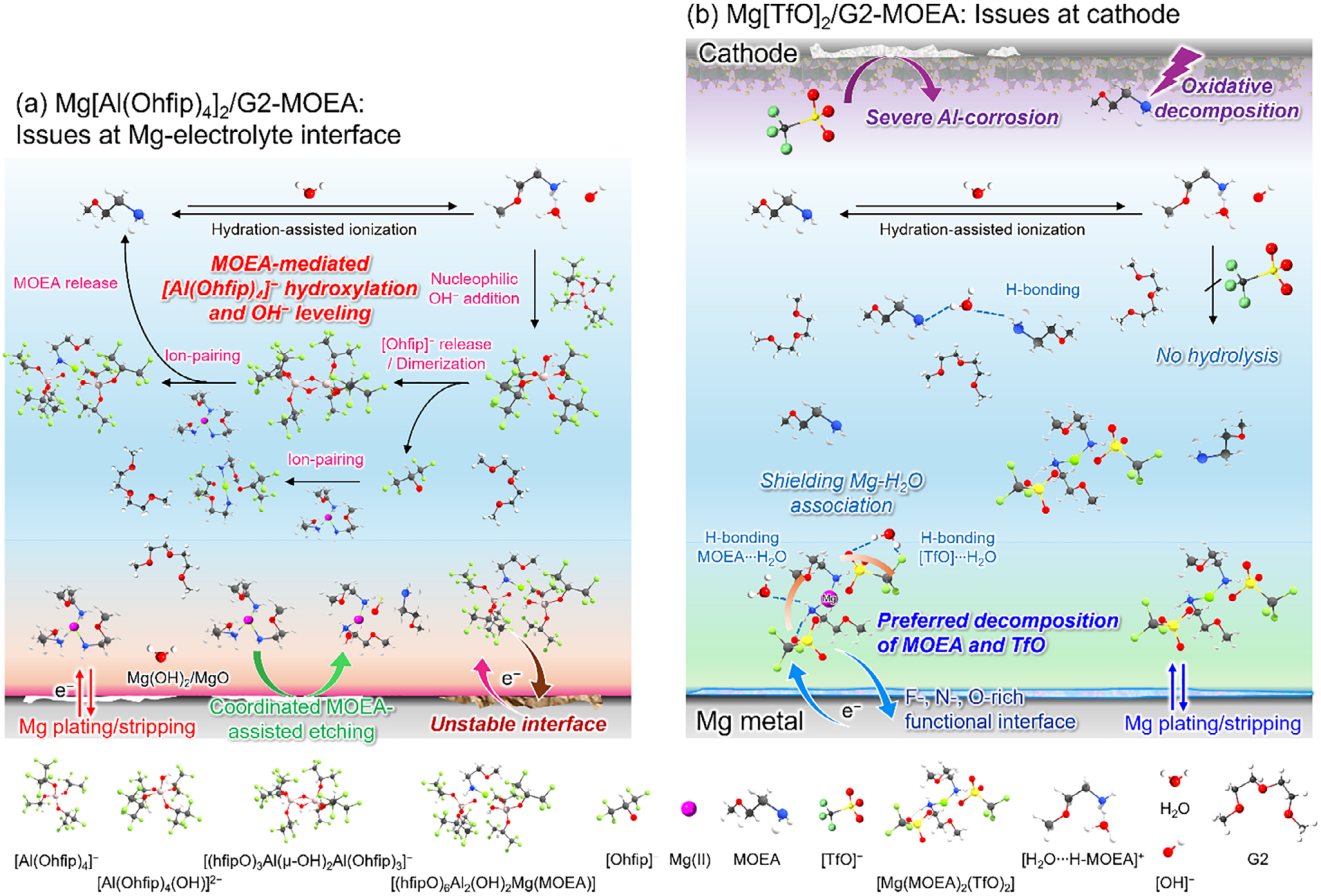
Schematic illustration of the relationship between compositional and interfacial chemistries in (a) Mg[Al(Ohfip)_4_]_2_/G2‐MOEA and (b) Mg[TfO]_2_/G2‐MOEA electrolytes.

## 3 Conclusions

The development of electrolyte materials that meet the requirements of both negative and positive electrode materials remains a long‐standing challenge in RMMB research. Two recent groundbreaking concepts—the adoption of weakly coordinating anions (WCAs) and the use of alkoxyalkylamine‐based solvents in electrolyte formulations—have individually demonstrated excellent compatibility, particularly with Mg negative electrodes. However, combining these two approaches has led to unexpectedly poor electrochemical performance. The electrolyte formulation with rather associative anions, in turn, exhibits remarkably favorable Mg plating/stripping performance. Comprehensive electrochemical, compositional, physical, spectroscopic, and computational analyses revealed a counter‐anion‐dependent solvation structure. In the Mg[Al(Ohfip)_4_]_2_/G2‐MOEA system, Mg^2+^ is primarily surrounded solely by chelating MOEA solvent molecules, whereas in the Mg[TfO]_2_‐based counterpart, both [TfO]^−^ and MOEA participate in coordination of Mg^2+^, and MOEA molecules are also bound to coordinated [TfO]^−^ via H‐bonding, thereby forming a multi‐compositional solvation sheath. Such distinct solvation environments are likely responsible for the observed interfacial compositions and consequent electrochemical characteristics. Moreover, detailed spectroscopic analyses combined with quantum chemical calculations revealed an unprecedented susceptibility of [Al(Ohfip)_4_]^−^ to trace amounts of water in the presence of MOEA. The MOEA‐mediated partial hydroxylation of [Al(Ohfip)_4_]^−^ combined with a peculiar etching effect effectively scavenges detrimental trace water and removes insulative Mg(OH)_2_ from the initial Mg‐interface, thereby leading to the formation of a less‐insulative interphase. Yet, the hydroxylation also results in the formation of large cation‐anion aggregate complexes, which in turn causes undesirable electrochemical performance. In contrast, the hydrophobic and highly associative nature of Mg[TfO]_2_ prevents water molecules from approaching the inner solvation sheath. The passivation of Mg metal by trace water is likely suppressed through the formation of a fluorine‐ and nitrogen‐rich functional interphase derived from [TfO]^−^ and MOEA, as well as by the restriction of water reactivity via H‐bonding with MOEA and [TfO]^−^.

Despite the excellent electrochemical Mg plating/stripping behavior of the Mg[TfO]_2_/G2‐MOEA electrolyte, its limited anodic stability and strong corrosiveness toward the Al current collector hinder its application in practical RMMBs. Regarding compatibility with positive electrodes, conventional ethereal electrolytes based on WCAs exhibit clear advantages over [TfO]^−^–amine systems, although their Mg plating/stripping performance remains insufficient for practical battery implementation. With respect to compositional engineering, regulating the interfacial stability of the WCA‐salt ether systems by rational halide‐free additives would be the most straightforward approaches to satisfy the requirements from both positive and negative electrode sides. Ishfaq et al., recently demonstrated significantly enhanced performance of the benchmarking Mg[Al(Ohfip)_4_]_2_/G2 electrolyte upon the integration of a well‐controlled amount of fluorinated cyclic ether [76]. Our group also developed the mixed‐coordination electrolyte combined with a fluorinated molecular additive which promotes anion‐ and additive‐derived favorable interphase formations and enhances cycling stability in high‐voltage RMBs [77]. As the integration of large quantity of non‐coordinating diluents like fluorinated ether in the ethereal electrolytes is detrimental for Mg plating/stripping performance [20, 21], the dosage needs to be adjusted appropriately. In addition to the above compositional approaches, Mg^2+^‐conductive alloy‐derived artificial interphases have attracted significant attention owing to their favorable characteristics [41, 78, 79]. Certain artificial interphase effectively regulates Mg^2+^‐ion flux at the electrode surface and promote interfacial kinetics, thereby improving overall battery performance. Since the development of electrolyte materials through the adoption of non‐ethereal solvents remains challenging due to the limited compatibility of highly reductive Mg metal with organic compounds [30], combining advanced ethereal electrolyte solutions with functional interphases could pave the way toward realizing high‐energy‐density RMMBs.

## Author Contributions

**Toshihiko Mandai**: Conceptualization, Data curation, Formal analysis, Funding acquisition, Investigation, Project administration, Resources, Validation, Writing – original draft, reviewing, and editing. **Antoine Barthélemy**: Quantum chemical calculations, Formal analysis, Validation, Writing – reviewing and editing. **Hendrik Koger**: Karl‐Fischer titration, FT‐IR measurements, Formal analysis, Resources, Validation, Writing – reviewing and editing. **Harald Scherer**: NMR measurements, Formal analysis, Validation, Writing – reviewing and editing. **Ingo Krossing**: Formal analysis, Validation, Writing – reviewing and editing.

## Conflicts of Interest

The authors declare no conflicts of interest.

## Supporting information




**Supporting File 1**: advs76897‐sup‐0001‐SuppMat.docx.

**Supporting File 2**: advs76897‐sup‐0002‐SuppVideos.zip.

## Data Availability

The data that support the findings of this study are available from the corresponding author upon reasonable request.
