# Supplementary material for: Unsuccessful Coupling of Weakly Coordinating Anion–Based Mg Salts and Alkoxyalkylamine Solvents for Rechargeable Mg Battery Application: Trace Water‐Driven Interfacial Failure
Source: Adv Sci (Weinh). 2026 Jul 29;13(59):e76897. doi: 10.1002/advs.76897 (PMC13418276; doi:10.1002/advs.76897)
Supplement: Supplementary file 1 [file ADVS-13-e76897-s002.docx]

*Supporting Information for:*

Unsuccessful Coupling of Weakly-Coordinating Anion-Based Mg salts and Alkoxyalkylamine Solvents for Rechargeable Mg Battery Applications: Trace Water-Driven Interfacial Failure

Toshihiko Mandai^1,^*, Antoine Barthélemy,^2^ Hendrik Koger,^2^ Harald Scherer,^2^ and Ingo Krossing^2^

^1^Research Center for Energy and Environmental Materials (GREEN), National Institute for Materials Science (NIMS), 1-1 Namiki, Tsukuba, Ibaraki 305-0044, Japan.

^2^Institute for Inorganic Chemistry and Analytic Chemistry as well as Freiburg Materials Research Center FMF, University of Freiburg, Albertstr. 21, 79104 Freiburg, Germany

Corresponding author footnote

TEL: +81-29-860-4464

Eail: MANDAI.Toshihiko@nims.go.jp

**§1 Materials and experimental methods**

1-1. *Materials.*

2-Methoxyethylamine (MOEA), 3-methoxypropylamine (MOPA), *N*-methyl *N*-propylamine (MPA), *N*,*N*-dimethyl propylamine (DMPA), magnesium triflate (Mg[TfO]_2_), trimethylaluminum (Al(CH_3_)_3_; 15% in hexane, *ca*. 1.4 mol dm^−3^), dimethyl sulfide borane (BH_3_∙S(CH_3_)_2_; >90%) were purchased from Tokyo Chemical Industry Co., Ltd. 1,1,1,3,3,3-Hexafluoro-iso-propanol (HOhfip), anhydrous diethyl ether, and anhydrous pentane were obtained from FUJIFILM Wako Chemicals. Di-*n*-butylmagnesium (Mg(*n*-Bu)_2_; 0.5 mol dm^−3^ in heptane) was purchased from Thermo Scientific Chemicals. Monoethylene glycol diethyl ether (G1) and diethylene glycol dimethyl ether (G2) for electrochemistry were obtained from Kanto Chemical Co., Inc. A series of amine-based solvents were dehydrated by distillation over CaH_2_ (Merck) and stored under an Ar-filled glovebox (< 1 ppm H_2_O and O_2_) with activated molecular sieves 4A. HOhfip was purified by vacuum distillation over activated molecular sieves 4A. Mg[TfO]_2_ was dried under vacuum heating at 200 °C for 1 week and stored in the glovebox prior to use. The other chemicals listed above were used as received. Mg[Al(Ohfip)_4_]_2_ was synthesized according to the reported procedures using Mg(*n*-Bu)_2_ or Mg(OCH_3_)_2_, Al(CH_3_)_3_, and HOhfip as reagents and pentane, diethyl ether, and G1 as reaction solvents.^[1,2]^ Mg[B(Ohfip)_4_]_2_ was synthesized through the modified transmetallation reactions between individually obtained B(Ohfip)_3_ and Mg(Ohfip)_2_ in G1.^[3]^ The chemical structures of Mg[Z(Ohfip)_4_]_2_ (Z = Al or B) were identified as adducts of three G1 solvents by ^1^H, ^11^B, ^19^F, and ^27^Al NMR spectroscopy, in consistence with the reported ones. Cu_2_Mo_6_S_8_ was obtained from Kojundo Chemical Laboratory Co. Ltd. Cu was leached out from Cu_2_Mo_6_S_8_ by a standard oxidation protocol to activate Mg^2+^ storage capability.^[4]^ FeV_3_O_9_∙1.1H_2_O (FeVO) was synthesized via an ambient solution process using NH_4_VO_3_ (99%, Sigma Aldrich) and Fe(NO_3_)_3_∙9H_2_O (98%, Sigma-Aldrich) as reagents, according to the literature reported elsewhere.^[5]^

The single amine or G2 solutions were prepared by dissolving the predetermined amounts of conductive salts (Mg[Al(Ohfip)_4_]_2_ and Mg(TfO)_2_) in the solvents, followed by vigorous stirring at 30 °C overnight in an Ar-filled glovebox. For the G2-MOEA dual solvent systems, the conductive salts were dissolved in the premixed solvents (G2:MOEA = 1:1 by vol.), followed by vigorous stirring at 30 °C overnight to ensure complete dissolution of the salts. The Mg salt concentration in solutions was fixed at 0.5 mol dm^−3^ for Mg[TfO]_2_, and 0.3 mol dm^−3^ for Mg[Al(Ohfip)_4_]_2_, while that for the single solvent systems of Mg[TfO]_2_ was less than 0.1 mol dm^−3^ (saturated) due to the limited solubility of the salt. As the water content of the amine-containing electrolytes could not be determined precisely by conventional coulometric Karl Fischer (KF) titration due to the direct reactivity of amines with the KF reagents, we adopted the designated titration protocol with the presence of benzoic acid. The details are given in the next section.

1-2. *Determination of water contents by coulometric KF Titration for MOEA-solvent*

The water determination was carried out with standard procedures using a SI Analytics TitroLine® 7500 KF trace coulometric Karl-Fischer titrator. As the generator electrode, a TZ 1753 electrode with diaphragm and a KF1150 were used as the detector and a micro double Pt electrode, respectively. For the anolyte and catholyte, Hydranal™ Coulomat AG and Hydranal™ Coulomat CG, respectively, were used.

The titration with this configuration gave a value of 165.8 ppm of water in the MOEA solvent, however the corresponding titration curve did not show a definite endpoint, but ended due to set time parameters of 600 s, indicating issues due to unwanted reactivity of the basic MOEA-solvent with the reagents or incomplete titration of the trace water. To improve the experimental accuracy, we followed the protocols of the Hydranal™ Center of Excellence given in Laboratory Report L288.^[6]^ Following the suggestion for MOPA, that structurally analogous to MOEA, in this setup, we performed multiple titrations resulting in 113.7–114.4 ppm with standard settings (*t*_min_ = 60 s, *t*_max_ = 600 s, 300 mV working voltage, ΔDrift = 0.2 µg min^−1^, drift tolerance at endpoint = 0.02 µg min^−1^) and 156.0 ppm with narrowed convergence criteria (*t*_min_ = 60 s, *t*_max_ = 1800 s, 300 mV working voltage, ΔDrift = 0.1 µg min^−1^, drift tolerance at endpoint = 0.02 µg min^−1^), and took the latter as the relatively reliable value. It should be emphasized that all observed titration curves were still not following ideal saturation curves due to the even high reactivity of neutralized amines with KF reagents although changing experimental parameters and adding the benzoic acid had a positive influence on the titration curve. An upswing of the displayed value of the Hydranal™ 1% water-standards from 1000.8 ppm to 1113–1138 ppm by adding MOEA further supports signaling issues with the MOEA-addition and incompatibility of MOEA-solvent with coulometric KF titration. Nevertheless, negligible reaction of the MOEA-based electrolytes with moisture-sensitive chemicals, such as CaH_2_, Mg(*n*-Bu)_2_, and Al(CH_3_)_3_, suggests sufficient dryness of solvents and salts by the proposed drying protocol.

1-3. *Electrochemistry*

The electrochemical Mg plating/stripping property was assessed preliminary by cyclic voltammetry (CV) with three-electrode configuration using Ag^+^/Ag couple as an external reference. Pt (φ = 3 mm; BAS) and Mg (φ = 2 mm; Fujifilm Wako Co., Ltd.) disks served as the respective working electrodes while a Mg strip was used as a counter electrode. An Ag^+^/Ag reference was prepared according to the reported procedure.^[7]^ The CV profiles were acquired using an electrochemical analyzer HSV-110 (Meiden Hokuto Corp.), and a fixed sweep rate of 10 mV s^−1^ was adopted for all CV measurements.

The long-term and rate capabilities of Mg plating/stripping were evaluated by galvanostatic cycling measurements on asymmetric and symmetric two-electrode cells (SB2A; EC Frontier Co., Ltd.). Coulombic efficiencies for Mg plating/stripping reactions were calculated from the charges passed during stripping over plating of the conventional galvanostatic measurements with the asymmetric cells. The electrochemical impedance spectroscopy was performed on the symmetric two-electrode cells by scanning the current frequency from 1 MHz to 10 mHz with a sinusoidally alternating voltage amplitude of 50 mV root-mean-square using a potentiostat/galvanostat (VMP3, BioLogic). Asymmetric cells were fabricated using Cu and Mg foils as working and counter electrodes, respectively. The surface of Mg electrodes (99.94%, *t* = 0.04 mm; Rikazai) was polished mechanically with SiC sandpaper (#400, #2000, and #5000), washed several times with anhydrous G1, and dried under vacuum at ambient temperature for 1 h, irrespective of the cell assemblies. The porous glass filter (GF/A; *t* = 0.26 mm; Whatman) served as a separator. The potentiostatic measurements (chronoamperometry) were conducted to characterize the corrosion stability of Al current collectors in the electrolytes. The two-electrode cells were polarized at predetermined voltages (2.0 and 2.5 V vs. Mg) for 1h each, then corresponding current profiles were monitored. Galvanostatic discharge-charge cycling measurements were conducted to assess the compatibility of the electrolytes with prospective positive electrode materials. Composite positive electrodes were fabricated by mixing active material powder, carbon nanotube/fluoropolymer dispersion (VTD-475N; Daikin Industries, Ltd.), acetylene black (AB), and polyvinylidene difluoride (PVdF) in a weight ratio of 92:0.5:3.5:4 in anhydrous *N*-methylpyrrolidone (FUJIFILM Wako Chemicals) to obtain a slurry. Then, the slurry was spread onto a carbon-coated Al foil current collector and dried at 120 °C for several days. The resultant composite sheet was compressed, cut into circular shape (φ = 16 mm, corresponding to 2.01 cm^2^), and served as working electrodes. The average loading was fixed at *ca*. 4 and 1.5 mg cm^−2^ for Mo_6_S_8_ and FeVO, respectively. Two-electrode cells comprising the composite electrode (φ = 16 mm), Mg negative electrode (φ = 16 mm, *t* = 40 μm), porous glass filter separator (GF/A; φ = 17 mm), and the electrolytes (100 μL) were assembled in a glovebox. The cycling test was conducted within a voltage range of 0.3–1.8 V with a current density of 12.2 mA g^−1^ for Mo_6_S_8_ and a range of 0.8–3.3 V with a current density of 10 mA g^−1^ for FeVO. For [Mg || FeVO] cells, the capacity was limited to 200 mAh g^−1^ to avoid successive electrolyte decomposition. Galvanostatic and potentiostatic measurements were conducted using an automatic charge-discharge instrument (HJ0610SD8C, Meiden-Hokuto Co., Ltd.).

All electrochemical cell assemblies were carried out in an Ar-filled glove-box. The electrochemical measurements were conducted at 30 °C unless otherwise specified, and the same measurements were conducted at least three times to ensure the experimental reproducibility.

1-4. *Chemical/physical analysis*

The structure, surface chemical nature, and morphological aspects of the deposits, cycled Mg electrodes, and polarized current collectors were characterized using X-ray diffraction (XRD; SmartLab, Rigaku), X-ray photoelectron spectroscopy (XPS; VersaProbe II, ULVAC-PHI), Time-of-Flight Secondary Ion Mass Spectrometry (TOF-SIMS; TOF.SIMS5-AD-GCIB, ION-TOF GmbH), scanning and transmission electron microscopy (SEM, JSM-7800F, JEOL; STEM, EM-ARM200F, JEOL) combined with energy-dispersive X-ray spectrometry (EDX) and electron energy loss spectroscopy (EELS). XRD profiles were acquired using Cu Kα radiation (λ = 1.54078 Å). XPS measurements were conducted with an Al Kα X-ray source under a base pressure of less than 6.7 × 10^−8^ Pa. The binding energy of the obtained spectra was calibrated using the C 1s peak of *sp*2-hybridized carbon at 284.5 eV as a reference. A primary ion gun of 30 kV Bi^3+^ and a sputter rate of 4 nm min^−1^ as SiO_2_ film were adopted for acquiring the depth profiles for TOF-SIMS measurements. The surface of the deposited Mg, cycled Mg electrodes, and polarized current collectors was observed using SEM and TEM, and the distribution of elements was subsequently characterized using EDX and EELS. To visualize the dynamic Mg plating/stripping behavior in the different electrolytes, confocal microscopic analysis was performed using the electrochemical confocal system (ECCS B320-N, Lasertec). To avoid any exposure to the air, the samples were fabricated and placed in a designated airtight chamber in an Ar-filled glovebox and transferred for each analysis or measurement.

1-5. *Solution states and characteristics*

The coordination states of solvents and anions in solutions were elucidated by ^1^H, ^19^F, and ^27^Al nuclear magnetic resonance (NMR), Raman spectroscopy, and Fourier-transform attenuated total reflection infrared (FT-ATR-IR) spectroscopy. All NMR spectra were recorded either using Bruker Avance IV Neo 400 MHz or Bruker Avance III HD 300 MHz NMR spectrometers. The samples were placed in the designated NMR tubes in the glove box, sealed by a typical flame sealing method or with a J. Young valve, and transferred to the spectrometer. The chemical shifts were calibrated using tetramethylsilane as a standard. Field correction for hetero nuclei was calculated according to the calibrated ^1^H NMR sprectra using the IUPAC Ξ-table. The Raman spectra were collected at ambient temperature (22±2 °C) using a laser Raman spectrometer (NRS-4500, JASCO) equipped with a 785 nm laser at a spectral resolution of 1 cm^−1^. The sample solutions were hermetically sealed in quartz glass tubes in an Ar-filled glovebox and were subjected to spectrometry without any exposure to moisture. The acquired spectra were calibrated using a Si standard. The spectral deconvolution was carried out by the Voigt function. For FT-ATR-IR measurements, the spectra were recorded in an Ar-filled Glovebox using a Bruker Alpha I spectrometer equipped with a diamond ATR unit. The liquids were contacted with the stamp of the ATR unit. The spectral range of 4000–400 cm^−2^ with a resolution of 2 cm^−2^ was adopted and 64 background scans and 128 sample scans were performed with each sample. The obtained spectra were evaluated using Bruker OPUS 7.5 software to produce three iterations of a baseline correction as well as a normalization of the spectra referring to the largest peak, respectively.

The ionic conductivities of the electrolyte solutions were measured using the complex impedance method with an impedance analyzer (VMP3, Biologic). A commercial cell (CT-57101B, TOA DKK Corporation) was used for the measurements. The cell was placed in a temperature-controlled chamber and held at 30 °C for 1 h to equilibrate the temperature prior to the measurements. The viscosities were measured at the same temperature using a kinematic viscometer (SVM3001, Anton Paar GmbH). The measurements were repeated at least three times for the different batches of the electrolytes prepared separately, and all of the standard deviations in the experimental values were within ±1% of the average.

**§2 Quantum chemical calculations**

*2-1. General considerations and calculation methods*

Quantum chemical calculations were performed with Turbomole (version 7.5.1),^[8–17]^ the Löwdin analysis was performed with the ORCA program package (version 5.0.0).^[18,19]^ Structures were optimized using density functional theory (DFT),^[16]^ internal coordinates, resolution of identity-approximation (RI),^[20–22]^ D3(BJ)-dispersion correction^[23,24]^ and a fine integration gridsize (gridsize = m5). Calculations were performed at the RI-B3LYP(D3BJ)/def2-TZVPP level of theory.^[25–28]^ Thermal and entropic contributions to the Gibbs energy were calculated without scaling factor at standard conditions (298.15 K, 0.1 MPa) with the FREEH module. Every species presented herein was checked in terms of reasonable geometry and electronic occupation with the EIGER module. Vibrational analyses were performed with the AOFORCE module, in order to detect imaginary frequencies.^[17]^

For each molecular species, the standard enthalpy *H*° at 298.15 K and 0.1 MPa was calculated from the electronic SCF energy *E*_SCF_ and the sum of translational, rotational, and vibrational energy including zero-point energy *E*_vrt_ (FREEH energy) using the following equation:

| *H*° = *E*_SCF_ + *E*_vrt_ + R ∙ *T* | equation 1 |
| --- | --- |

*E*_SCF_: electronic SCF energy

*E*_vrt_: sum of translational, rotational, and vibrational energy including zero-point energy

R: universal gas constant (ca. 8.314 J K^–1^ mol^–1^)

*T*: temperature in Kelvin (298.15 K)

The Gibbs free energy *G*° follows from the standard enthalpy *H*° and the standard entropy *S*°:

| *G*° = *H*° – *S*° ∙ *T* | equation 2 |
| --- | --- |

Solvation effects were incorporated using the conductor like screening model (COSMO)^[29]^ for G2 as the solvent. A dielectric constant of 7.23 at 298 K was assumed for this solvent.^[30]^ Single point calculations were performed on the optimized gas phase structures and the results from the vibrational analysis were taken from the respective gas phase calculations.

In the following section, the coordinates of the calculated species are given.

**[Al(Ohfip)_4_]^−^**

```
Method: (RI-)B3LYP(D3BJ)/def2-TZVPP
Symmetry: s4

Cartesian coordinates in Ångström:
Al    0.0000000   -0.0000000    0.0000000
O     0.6627544    1.3378470   -0.9132161
O     1.3378470   -0.6627544    0.9132161
O    -1.3378470    0.6627544    0.9132161
O    -0.6627544   -1.3378470   -0.9132161
C     2.5718666   -0.1154759    1.1213708
H     2.7401119    0.8171802    0.5665777
C    -2.5718666    0.1154759    1.1213708
F    -3.6218081    2.2787855    1.2541324
C    -0.1154759   -2.5718666   -1.1213708
C     0.1154759    2.5718666   -1.1213708
F    -1.7907263   -1.1302447    2.9625517
C    -2.7411598   -0.2502539    2.6082154
F    -3.9357272   -0.8339495    2.8550717
F    -2.6371847    0.8090052    3.4267114
C    -3.6504006    1.0937109    0.6208196
F    -4.8984040    0.5962342    0.7517491
F    -3.4576800    1.3378375   -0.6889177
H    -2.7401119   -0.8171802    0.5665777
F     3.9357272    0.8339495    2.8550717
C     2.7411598    0.2502539    2.6082154
F     2.6371847   -0.8090052    3.4267114
F     1.7907263    1.1302447    2.9625517
F     3.6218081   -2.2787855    1.2541324
C     3.6504006   -1.0937109    0.6208196
F     3.4576800   -1.3378375   -0.6889177
F     4.8984040   -0.5962342    0.7517491
F     1.1302447   -1.7907263   -2.9625517
C     0.2502539   -2.7411598   -2.6082154
F     0.8339495   -3.9357272   -2.8550717
F    -0.8090052   -2.6371847   -3.4267114
F    -0.8339495    3.9357272   -2.8550717
C    -0.2502539    2.7411598   -2.6082154
F     0.8090052    2.6371847   -3.4267114
F    -1.1302447    1.7907263   -2.9625517
H    -0.8171802    2.7401119   -0.5665777
F     1.3378375    3.4576800    0.6889177
C     1.0937109    3.6504006   -0.6208196
F     2.2787855    3.6218081   -1.2541324
F     0.5962342    4.8984040   -0.7517491
F    -0.5962342   -4.8984040   -0.7517491
H     0.8171802   -2.7401119   -0.5665777
C    -1.0937109   -3.6504006   -0.6208196
F    -2.2787855   -3.6218081   -1.2541324
F    -1.3378375   -3.4576800    0.6889177

SCF energy GEOOPT =  -3399.862800190 H
ZPE =   542.0 kJ/mol
FREEH energy =   649.71 kJ/mol
FREEH entropy =   1.14846 kJ/mol/K

$vibrational spectrum
#  mode     symmetry     wave number   IR intensity    selection rules
#                         cm**(-1)        km/mol         IR     RAMAN
     1                       -0.00         0.00000        -       -
     2                       -0.00         0.00000        -       -
     3                        0.00         0.00000        -       -
     4                        0.00         0.00000        -       -
     5                        0.00         0.00000        -       -
     6                        0.00         0.00000        -       -
     7        b               7.51         0.16453       YES     YES
     8        b               7.65         0.00000       YES     YES
     9        b              14.38         0.00000       YES     YES
    10        e              14.44         0.08610       YES     YES
    11        e              14.44         0.08610       YES     YES
    12        b              16.41         0.00428       YES     YES
    13        e              19.26         0.10503       YES     YES
    14        e              19.26         0.10503       YES     YES
    15        b              24.13         0.12888       YES     YES
    16        b              30.54         0.00180       YES     YES
    17        e              30.75         0.04367       YES     YES
    18        e              30.75         0.04367       YES     YES
    19        b              30.80         0.00000       YES     YES
    20        e              41.51         0.54074       YES     YES
    21        e              41.51         0.54074       YES     YES
    22        a              54.29         0.00000       NO      YES
    23        a              65.47         0.00000       NO      YES
    24        b              66.81         0.04849       YES     YES
    25        e              70.08         0.16901       YES     YES
    26        e              70.08         0.16901       YES     YES
    27        a              92.53         0.00000       NO      YES
    28        b              94.30         0.28436       YES     YES
    29        e             151.71         2.10095       YES     YES
    30        e             151.71         2.10095       YES     YES
    31        b             158.57         2.63877       YES     YES
    32        b             160.97         0.00000       YES     YES
    33        e             184.71         0.67592       YES     YES
    34        e             184.71         0.67592       YES     YES
    35        a             189.51         0.00000       NO      YES
    36        b             195.50         0.20066       YES     YES
    37        b             243.95         0.37723       YES     YES
    38        e             261.34         0.46628       YES     YES
    39        e             261.34         0.46628       YES     YES
    40        a             269.50         0.00000       NO      YES
    41        b             290.51         7.85091       YES     YES
    42        e             291.71         0.10472       YES     YES
    43        e             291.71         0.10472       YES     YES
    44        a             291.71         0.00000       NO      YES
    45        b             291.78         0.96842       YES     YES
    46        e             305.06         8.00062       YES     YES
    47        e             305.06         8.00062       YES     YES
    48        a             325.79         0.00000       NO      YES
    49        b             326.37         1.16348       YES     YES
    50        e             329.58         2.24818       YES     YES
    51        e             329.58         2.24818       YES     YES
    52        e             357.29         2.07266       YES     YES
    53        e             357.29         2.07265       YES     YES
    54        a             360.04         0.00000       NO      YES
    55        a             367.17         0.00000       NO      YES
    56        b             379.64         8.62148       YES     YES
    57        b             434.28        37.82844       YES     YES
    58        e             443.51        52.91815       YES     YES
    59        e             443.51        52.91815       YES     YES
    60        a             514.74         0.00000       NO      YES
    61        b             515.87        13.01870       YES     YES
    62        e             516.11        14.23975       YES     YES
    63        e             516.11        14.23974       YES     YES
    64        a             527.85         0.00000       NO      YES
    65        e             527.91         2.70232       YES     YES
    66        e             527.91         2.70232       YES     YES
    67        b             528.10         5.78915       YES     YES
    68        a             533.61         0.00000       NO      YES
    69        e             544.69         0.26541       YES     YES
    70        e             544.69         0.26541       YES     YES
    71        b             544.77         0.00000       YES     YES
    72        b             544.83         1.43937       YES     YES
    73        b             562.01        22.90461       YES     YES
    74        e             570.69        31.37741       YES     YES
    75        e             570.69        31.37741       YES     YES
    76        a             681.04         0.00000       NO      YES
    77        e             681.86        30.34061       YES     YES
    78        e             681.86        30.34060       YES     YES
    79        b             683.62        92.82511       YES     YES
    80        a             692.00         0.00000       NO      YES
    81        b             713.65         0.99438       YES     YES
    82        e             723.96         2.67510       YES     YES
    83        e             723.96         2.67510       YES     YES
    84        a             758.49         0.00000       NO      YES
    85        b             782.91        48.86856       YES     YES
    86        e             810.40        51.00934       YES     YES
    87        e             810.40        51.00931       YES     YES
    88        b             855.71         0.00000       YES     YES
    89        b             858.15       141.23018       YES     YES
    90        e             858.90       131.36215       YES     YES
    91        e             858.90       131.36191       YES     YES
    92        b             886.91         0.00000       YES     YES
    93        e             888.06        44.62793       YES     YES
    94        e             888.06        44.62818       YES     YES
    95        b             889.38        81.20599       YES     YES
    96        a            1084.71         0.00000       NO      YES
    97        e            1090.82        81.57266       YES     YES
    98        e            1090.82        81.57262       YES     YES
    99        b            1095.49       280.47177       YES     YES
   100        e            1120.22        11.47702       YES     YES
   101        e            1120.22        11.47701       YES     YES
   102        a            1120.76         0.00000       NO      YES
   103        b            1127.33        77.85848       YES     YES
   104        a            1171.35         0.00000       NO      YES
   105        e            1171.86       761.12476       YES     YES
   106        e            1171.86       761.12597       YES     YES
   107        b            1174.47         6.21728       YES     YES
   108        b            1189.37        55.34264       YES     YES
   109        e            1199.11       168.73073       YES     YES
   110        e            1199.11       168.72996       YES     YES
   111        a            1205.12         0.00000       NO      YES
   112        b            1206.61       618.63174       YES     YES
   113        e            1209.13       985.34184       YES     YES
   114        e            1209.13       985.34171       YES     YES
   115        a            1244.84         0.00000       NO      YES
   116        e            1254.58       127.36657       YES     YES
   117        e            1254.58       127.36611       YES     YES
   118        b            1256.41         0.00000       YES     YES
   119        b            1256.73       357.34236       YES     YES
   120        a            1289.58         0.00000       NO      YES
   121        e            1292.30       184.95766       YES     YES
   122        e            1292.30       184.95769       YES     YES
   123        b            1293.77       114.64324       YES     YES
   124        b            1382.56         0.00000       YES     YES
   125        e            1384.81        18.72553       YES     YES
   126        e            1384.81        18.72541       YES     YES
   127        b            1389.27         0.00000       YES     YES
   128        b            1389.99         8.57118       YES     YES
   129        e            1392.43        28.59256       YES     YES
   130        e            1392.43        28.59234       YES     YES
   131        b            1394.08       106.12606       YES     YES
   132        b            3001.68         0.67070       YES     YES
   133        e            3001.82        41.00677       YES     YES
   134        e            3001.82        41.00677       YES     YES
   135        b            3002.41         0.00000       YES     YES
$end

Total COSMO energy + OC correction = -3399.8950269961 H
```

**[Al(Ohfip)_3_(MOEA^−H^)]^−^**

```
Method: (RI-)B3LYP(D3BJ)/def2-TZVPP
Symmetry: c1

Cartesian coordinates in Ångström:
F     4.7708093   -0.2376676    0.2950779
F     3.4570048   -0.1660689   -1.4264647
C     3.5112855   -0.5004311   -0.1242365
F     3.6917422    0.3033143    2.7199801
F     3.3379651   -1.8316719   -0.0478053
F     2.7345402    4.7466446   -0.3000753
H     2.7437527    1.3506235    0.5554761
C     2.4495338    0.2967162    0.6579235
C     2.4887959    0.0067632    2.1716076
F     0.9126234    4.9707939   -2.4171240
C     1.6378802    4.5695246    0.4573342
F     2.2240563   -1.2765374    2.4704639
F     1.9976471    3.8007715    1.5029407
F     1.2978513    5.7788041    0.9573412
O     0.8755442    2.6709618   -0.7426083
O     1.2144695    0.0158845    0.1608396
F     1.5755335    0.7641621    2.7984077
C    -0.0260972    4.7486996   -1.4808577
C     0.4784871    3.8972490   -0.2995954
H    -4.2370056   -2.1010962   -1.0451538
H     0.2019434   -1.5084452   -1.7742476
H    -1.7411632   -2.8456392   -2.6179139
F    -0.4899117    5.9565605   -1.0861567
Al    0.0296752    1.1327838   -0.5100280
F    -1.0492877    4.1155274   -2.0843154
H    -1.3091634    0.8720311   -2.5707263
C    -3.3914328   -2.6140327   -0.5673845
O    -2.1794046   -1.9461155   -0.8113971
C    -1.8630077   -1.8381648   -2.1889371
H    -3.5493122   -2.6238516    0.5098893
C    -0.5814680   -1.0309489   -2.3667635
N    -0.6861569    0.3508648   -1.9717351
H    -0.3579338    3.8590731    0.4120391
H    -3.3630685   -3.6485356   -0.9364053
H    -0.2854057   -1.1458625   -3.4221900
H    -2.6929425   -1.3464585   -2.7192926
O    -1.2274061    1.6016193    0.6561498
F    -1.5628082   -0.4076871    2.5012252
C    -2.4296266    0.9672360    0.7916698
F    -3.4270002    2.2958517   -0.8844349
H    -2.5400347    0.0959872    0.1321920
C    -2.5793022    0.4191356    2.2215635
F    -2.5917294    1.3792246    3.1643411
C    -3.5682817    1.9257917    0.4036922
F    -3.5930658    3.0522627    1.1372858
F    -3.7227917   -0.2980816    2.3735656
F    -4.7909724    1.3530437    0.5107534

SCF energy GEOOPT =  -2859.617261818 H
ZPE =   707.8 kJ/mol
FREEH energy =   808.01 kJ/mol
FREEH entropy =   1.06716 kJ/mol/K

$vibrational spectrum
#  mode     symmetry     wave number   IR intensity    selection rules
#                         cm**(-1)        km/mol         IR     RAMAN
     1                       -0.00         0.00000        -       -
     2                       -0.00         0.00000        -       -
     3                        0.00         0.00000        -       -
     4                        0.00         0.00000        -       -
     5                        0.00         0.00000        -       -
     6                        0.00         0.00000        -       -
     7        a               9.68         0.01078       YES     YES
     8        a              11.98         0.14201       YES     YES
     9        a              14.32         0.39485       YES     YES
    10        a              16.91         0.20361       YES     YES
    11        a              18.43         0.02327       YES     YES
    12        a              21.55         0.14022       YES     YES
    13        a              24.32         0.22210       YES     YES
    14        a              29.42         0.05574       YES     YES
    15        a              30.46         0.04476       YES     YES
    16        a              31.74         0.01876       YES     YES
    17        a              34.75         0.50201       YES     YES
    18        a              41.13         0.20349       YES     YES
    19        a              48.24         0.45498       YES     YES
    20        a              60.06         0.36675       YES     YES
    21        a              66.76         0.04862       YES     YES
    22        a              69.53         0.13171       YES     YES
    23        a              73.17         0.26649       YES     YES
    24        a              78.36         0.47670       YES     YES
    25        a              89.26         0.16994       YES     YES
    26        a              97.34         4.13378       YES     YES
    27        a             124.73         0.63566       YES     YES
    28        a             148.19         1.44678       YES     YES
    29        a             157.87         0.76861       YES     YES
    30        a             161.33         1.88546       YES     YES
    31        a             176.00         1.13440       YES     YES
    32        a             186.14         0.75989       YES     YES
    33        a             191.17         0.20475       YES     YES
    34        a             206.48         0.19396       YES     YES
    35        a             233.83         2.32119       YES     YES
    36        a             239.64         3.76357       YES     YES
    37        a             257.61         0.92235       YES     YES
    38        a             264.84         1.91230       YES     YES
    39        a             275.69         2.92897       YES     YES
    40        a             286.87         8.15698       YES     YES
    41        a             290.79         0.09010       YES     YES
    42        a             291.54         0.12142       YES     YES
    43        a             291.61         0.10481       YES     YES
    44        a             310.50         9.16940       YES     YES
    45        a             322.47         2.01566       YES     YES
    46        a             325.81         1.15071       YES     YES
    47        a             329.22         7.45532       YES     YES
    48        a             331.33         1.13374       YES     YES
    49        a             339.73        11.96630       YES     YES
    50        a             353.39         1.45259       YES     YES
    51        a             358.48         2.50043       YES     YES
    52        a             373.73         0.71594       YES     YES
    53        a             396.38        34.92061       YES     YES
    54        a             399.90        45.46700       YES     YES
    55        a             437.74        59.84341       YES     YES
    56        a             449.53        45.71563       YES     YES
    57        a             515.02         3.15042       YES     YES
    58        a             515.91        13.22351       YES     YES
    59        a             516.86        14.02304       YES     YES
    60        a             525.82         3.99076       YES     YES
    61        a             526.76         2.21793       YES     YES
    62        a             527.33         5.49749       YES     YES
    63        a             527.55         6.05878       YES     YES
    64        a             543.85         0.33429       YES     YES
    65        a             543.92         0.39653       YES     YES
    66        a             544.17         0.36139       YES     YES
    67        a             548.61        19.41139       YES     YES
    68        a             560.93        35.51964       YES     YES
    69        a             568.42        37.89768       YES     YES
    70        a             666.30        22.23965       YES     YES
    71        a             680.66        31.26887       YES     YES
    72        a             680.89         4.65653       YES     YES
    73        a             682.35        75.84011       YES     YES
    74        a             706.58         1.85058       YES     YES
    75        a             717.41         2.55892       YES     YES
    76        a             725.56        23.01600       YES     YES
    77        a             763.10        34.88817       YES     YES
    78        a             775.47        73.31553       YES     YES
    79        a             787.50        66.91346       YES     YES
    80        a             845.00        30.34896       YES     YES
    81        a             854.31        22.80487       YES     YES
    82        a             855.44       146.24945       YES     YES
    83        a             856.68       130.43545       YES     YES
    84        a             884.45        39.09905       YES     YES
    85        a             885.73        21.95858       YES     YES
    86        a             887.68        71.94217       YES     YES
    87        a             926.77        25.84131       YES     YES
    88        a            1032.04        44.51815       YES     YES
    89        a            1076.62         4.27342       YES     YES
    90        a            1082.88        32.90410       YES     YES
    91        a            1087.11       109.08919       YES     YES
    92        a            1089.79       118.99295       YES     YES
    93        a            1106.32        60.88743       YES     YES
    94        a            1113.53        19.07965       YES     YES
    95        a            1118.20        75.26275       YES     YES
    96        a            1139.45        59.10045       YES     YES
    97        a            1159.57        87.34913       YES     YES
    98        a            1160.03       701.69359       YES     YES
    99        a            1163.69       367.54237       YES     YES
   100        a            1167.96       371.87781       YES     YES
   101        a            1180.88         6.94785       YES     YES
   102        a            1190.44        57.99495       YES     YES
   103        a            1198.08       480.48622       YES     YES
   104        a            1201.48       260.90197       YES     YES
   105        a            1203.78       144.03090       YES     YES
   106        a            1208.95       652.81218       YES     YES
   107        a            1210.28       256.30221       YES     YES
   108        a            1233.41       206.51168       YES     YES
   109        a            1250.15        18.49427       YES     YES
   110        a            1253.71       101.22409       YES     YES
   111        a            1254.65       180.18387       YES     YES
   112        a            1257.41       176.62678       YES     YES
   113        a            1288.30        15.45503       YES     YES
   114        a            1290.26       180.76658       YES     YES
   115        a            1291.62       144.73364       YES     YES
   116        a            1297.11         4.06795       YES     YES
   117        a            1379.93         3.49973       YES     YES
   118        a            1385.00         9.34631       YES     YES
   119        a            1386.37         8.98266       YES     YES
   120        a            1390.61        24.62098       YES     YES
   121        a            1395.34        48.86821       YES     YES
   122        a            1399.35        24.58365       YES     YES
   123        a            1404.10        14.07053       YES     YES
   124        a            1408.99        18.09292       YES     YES
   125        a            1428.67        45.61769       YES     YES
   126        a            1474.85         1.17948       YES     YES
   127        a            1485.95         2.04166       YES     YES
   128        a            1486.83         7.09291       YES     YES
   129        a            1497.47         9.84323       YES     YES
   130        a            1513.82         1.31951       YES     YES
   131        a            2911.02        30.91519       YES     YES
   132        a            2931.29       182.53778       YES     YES
   133        a            2953.81       124.06060       YES     YES
   134        a            2956.97        93.87593       YES     YES
   135        a            2991.24        65.88253       YES     YES
   136        a            2993.59        21.71571       YES     YES
   137        a            2994.35        25.38302       YES     YES
   138        a            3001.93        25.16848       YES     YES
   139        a            3059.28        19.06627       YES     YES
   140        a            3105.58        21.93627       YES     YES
   141        a            3566.90         0.49796       YES     YES
$end

Total COSMO energy + OC correction = -2859.6659796107 H
```

**MOEA**

```
Method: (RI-)B3LYP(D3BJ)/def2-TZVPP
Symmetry: cs

Cartesian coordinates in Ångström:
C    -2.2289127    0.7132732    0.0000000
O    -0.8457427    0.9856340    0.0000000
H    -2.7507169    1.6683859    0.0000000
H    -2.5252202    0.1438727    0.8899788
H    -2.5252202    0.1438727   -0.8899788
C    -0.0504851   -0.1812632    0.0000000
H    -0.2730722   -0.7953534    0.8860577
C     1.4192382    0.2196677    0.0000000
H    -0.2730722   -0.7953534   -0.8860577
H     1.6159855    0.8413017    0.8752706
H     1.6159855    0.8413017   -0.8752706
N     2.3561448   -0.8993880    0.0000000
H     2.2258944   -1.4859245   -0.8151968
H     2.2258944   -1.4859245    0.8151968

SCF energy GEOOPT =  -249.6600540156 H
ZPE =   329.7 kJ/mol
FREEH energy =   347.38 kJ/mol
FREEH entropy =   0.33225 kJ/mol/K

$vibrational spectrum
#  mode     symmetry     wave number   IR intensity    selection rules
#                         cm**(-1)        km/mol         IR     RAMAN
     1                       -0.00         0.00000        -       -
     2                        0.00         0.00000        -       -
     3                        0.00         0.00000        -       -
     4                        0.00         0.00000        -       -
     5                        0.00         0.00000        -       -
     6                        0.00         0.00000        -       -
     7        a"            101.35         0.00185       YES     YES
     8        a"            122.81         0.68374       YES     YES
     9        a'            195.33         0.14755       YES     YES
    10        a"            232.33         4.81571       YES     YES
    11        a"            281.06        43.11768       YES     YES
    12        a'            427.35         1.52434       YES     YES
    13        a'            436.00        12.65887       YES     YES
    14        a"            794.55         0.00191       YES     YES
    15        a'            829.43       190.68792       YES     YES
    16        a'            980.00        33.01312       YES     YES
    17        a"           1002.51         0.04662       YES     YES
    18        a'           1066.09        30.87728       YES     YES
    19        a'           1095.76         3.03372       YES     YES
    20        a'           1152.04       150.25432       YES     YES
    21        a"           1174.88         1.23140       YES     YES
    22        a"           1217.65         6.46474       YES     YES
    23        a'           1222.91        34.60048       YES     YES
    24        a"           1307.31         0.01769       YES     YES
    25        a'           1350.92        11.44297       YES     YES
    26        a"           1376.38         0.02816       YES     YES
    27        a'           1422.37         5.44344       YES     YES
    28        a'           1478.08         0.93803       YES     YES
    29        a"           1487.36         6.87803       YES     YES
    30        a'           1496.15         5.05094       YES     YES
    31        a'           1504.60         5.68785       YES     YES
    32        a'           1522.27         1.34280       YES     YES
    33        a'           1661.22        20.71726       YES     YES
    34        a'           2936.16        51.46254       YES     YES
    35        a"           2960.41        64.84153       YES     YES
    36        a'           2970.06        74.26840       YES     YES
    37        a"           3009.58        60.09161       YES     YES
    38        a'           3042.10        32.90766       YES     YES
    39        a"           3080.97        31.97102       YES     YES
    40        a'           3110.74        30.16378       YES     YES
    41        a'           3494.27         1.05440       YES     YES
    42        a"           3571.97         0.44627       YES     YES
$end

Total COSMO energy + OC correction = -249.6685783392 H
```

**H_2_O**

```
Method: (RI-)B3LYP(D3BJ)/def2-TZVPP
Symmetry: c2v

Cartesian coordinates in Ångström:
O     0.0000000    0.0000000   -0.3903870
H     0.7618347    0.0000000    0.1951933
H    -0.7618347    0.0000000    0.1951933

SCF energy GEOOPT =    -76.430325351 H
ZPE =   55.92 kJ/mol
FREEH energy =    63.37 kJ/mol
FREEH entropy =   0.18892 kJ/mol/K

$vibrational spectrum
#  mode     symmetry     wave number   IR intensity    selection rules
#                         cm**(-1)        km/mol         IR     RAMAN
     1                       -0.00         0.00000        -       -
     2                       -0.00         0.00000        -       -
     3                        0.00         0.00000        -       -
     4                        0.00         0.00000        -       -
     5                        0.00         0.00000        -       -
     6                        0.00         0.00000        -       -
     7        a1           1632.68        69.68267       YES     YES
     8        a1           3807.14         4.37599       YES     YES
     9        b1           3909.88        48.48977       YES     YES
$end

Total COSMO energy + OC correction = -76.4396557299 H
```

**[Al(Ohfip)_3_OH]^−^**

```
Method: (RI-)B3LYP(D3BJ)/def2-TZVPP
Symmetry: c1

Cartesian coordinates in Ångström:
Al    0.1011862   -0.0268308   -0.0069722
O     0.6946327    1.3450161   -0.9360792
O     1.3940917   -0.7988663    0.8459334
O    -1.2339361    0.6562259    0.9293653
O    -0.6474010   -1.3098141   -0.9629427
C    -2.4708102    0.1204847    1.1308456
F    -3.5159377    2.2885206    1.2700245
C    -0.1494744   -2.5695643   -1.1267746
C     0.0787328    2.5437930   -1.1373625
F    -1.7234987   -1.1422308    2.9786354
C    -2.6544631   -0.2447702    2.6164688
F    -3.8614210   -0.8059975    2.8619825
F    -2.5345991    0.8122622    3.4390009
C    -3.5435110    1.1059172    0.6291679
F    -4.7966112    0.6148166    0.7486584
F    -3.3438775    1.3587656   -0.6776235
H    -2.6468342   -0.8101975    0.5744693
F     1.2519697   -1.8794297   -2.8936948
C     0.2992673   -2.7755903   -2.5861142
F     0.8345714   -4.0038852   -2.7888169
F    -0.6964295   -2.6262014   -3.4745513
F    -0.9246661    3.8758666   -2.8710624
C    -0.2722844    2.7142848   -2.6281068
F     0.8054825    2.6907489   -3.4313661
F    -1.0851505    1.7191650   -3.0157090
H    -0.8717628    2.6517971   -0.5974938
F     1.2127653    3.4917090    0.7036268
C     0.9771252    3.6782451   -0.6090002
F     2.1721799    3.7401053   -1.2217054
F     0.4009697    4.8954588   -0.7338972
F    -0.7547532   -4.8712810   -0.7635463
H     0.7390820   -2.7722115   -0.5128633
C    -1.2050229   -3.5988371   -0.6831507
F    -2.3429006   -3.5334836   -1.3964194
F    -1.5318787   -3.3807324    0.6050608
H     2.1297376   -0.2817887    1.1670231

SCF energy GEOOPT =  -2686.408702673 H
ZPE =   441.2 kJ/mol
FREEH energy =   527.26 kJ/mol
FREEH entropy =   0.95057 kJ/mol/K

$vibrational spectrum
#  mode     symmetry     wave number   IR intensity    selection rules
#                         cm**(-1)        km/mol         IR     RAMAN
     1                       -0.00         0.00000        -       -
     2                       -0.00         0.00000        -       -
     3                        0.00         0.00000        -       -
     4                        0.00         0.00000        -       -
     5                        0.00         0.00000        -       -
     6                        0.00         0.00000        -       -
     7        a               9.43         0.05706       YES     YES
     8        a              12.06         0.22660       YES     YES
     9        a              15.11         0.10762       YES     YES
    10        a              16.70         0.01626       YES     YES
    11        a              21.93         0.19226       YES     YES
    12        a              24.65         0.09620       YES     YES
    13        a              29.61         0.04216       YES     YES
    14        a              30.35         0.00860       YES     YES
    15        a              30.70         0.00389       YES     YES
    16        a              37.44         0.80631       YES     YES
    17        a              49.13         0.51092       YES     YES
    18        a              56.77         0.10668       YES     YES
    19        a              68.26         0.54914       YES     YES
    20        a              71.98         0.16740       YES     YES
    21        a              87.95         0.27174       YES     YES
    22        a             102.79         1.67365       YES     YES
    23        a             125.83         8.82587       YES     YES
    24        a             150.60         2.00425       YES     YES
    25        a             156.29         2.54138       YES     YES
    26        a             170.83        23.59951       YES     YES
    27        a             173.57         2.51783       YES     YES
    28        a             187.56         2.24636       YES     YES
    29        a             191.93         2.28372       YES     YES
    30        a             222.34        20.03393       YES     YES
    31        a             231.49        24.03602       YES     YES
    32        a             256.97         0.25059       YES     YES
    33        a             267.64         0.30577       YES     YES
    34        a             279.69         9.27855       YES     YES
    35        a             290.92         0.18609       YES     YES
    36        a             291.03         0.19196       YES     YES
    37        a             291.38         0.15275       YES     YES
    38        a             296.32         9.28206       YES     YES
    39        a             312.81         9.41052       YES     YES
    40        a             325.81         0.52138       YES     YES
    41        a             327.03         1.49137       YES     YES
    42        a             333.65         0.69583       YES     YES
    43        a             354.90         1.60190       YES     YES
    44        a             359.82         1.03138       YES     YES
    45        a             374.36         5.80591       YES     YES
    46        a             403.45        18.42347       YES     YES
    47        a             435.41        42.93163       YES     YES
    48        a             436.14        53.23267       YES     YES
    49        a             515.45         3.17007       YES     YES
    50        a             516.08        12.61022       YES     YES
    51        a             516.66        11.64534       YES     YES
    52        a             527.27         1.23889       YES     YES
    53        a             527.34         2.94452       YES     YES
    54        a             527.46         4.59457       YES     YES
    55        a             536.78         0.97055       YES     YES
    56        a             543.97         0.46081       YES     YES
    57        a             544.28         0.45548       YES     YES
    58        a             544.42         0.61007       YES     YES
    59        a             560.95        34.02735       YES     YES
    60        a             563.45        26.09205       YES     YES
    61        a             666.05       163.29594       YES     YES
    62        a             680.32         9.73517       YES     YES
    63        a             681.30        31.72928       YES     YES
    64        a             682.60        81.35144       YES     YES
    65        a             687.10        17.85249       YES     YES
    66        a             710.29         0.43061       YES     YES
    67        a             724.96        24.94302       YES     YES
    68        a             747.78         4.57744       YES     YES
    69        a             771.61        58.68292       YES     YES
    70        a             797.78        92.85856       YES     YES
    71        a             815.32        60.08586       YES     YES
    72        a             856.07        91.34316       YES     YES
    73        a             856.81       112.30490       YES     YES
    74        a             858.45       123.89566       YES     YES
    75        a             884.27        19.89557       YES     YES
    76        a             885.66        55.61161       YES     YES
    77        a             886.77        60.55077       YES     YES
    78        a            1084.47        34.24443       YES     YES
    79        a            1088.95        81.12722       YES     YES
    80        a            1091.36       220.43201       YES     YES
    81        a            1112.37        23.26911       YES     YES
    82        a            1114.82         9.19845       YES     YES
    83        a            1119.67        67.20557       YES     YES
    84        a            1165.00       690.83474       YES     YES
    85        a            1166.39       337.47292       YES     YES
    86        a            1168.20       265.03230       YES     YES
    87        a            1192.14        84.05104       YES     YES
    88        a            1198.67       244.03743       YES     YES
    89        a            1202.86        36.91648       YES     YES
    90        a            1205.64       666.70070       YES     YES
    91        a            1206.90       860.50996       YES     YES
    92        a            1235.39       143.75791       YES     YES
    93        a            1253.10       155.58474       YES     YES
    94        a            1253.92       132.77763       YES     YES
    95        a            1256.07       192.42423       YES     YES
    96        a            1287.88        69.35557       YES     YES
    97        a            1290.40       152.24197       YES     YES
    98        a            1291.13       116.83802       YES     YES
    99        a            1378.30         7.50703       YES     YES
   100        a            1381.01        10.48521       YES     YES
   101        a            1384.69         7.36999       YES     YES
   102        a            1390.45        11.51880       YES     YES
   103        a            1393.71        46.80688       YES     YES
   104        a            1395.49        61.62830       YES     YES
   105        a            2993.86        25.91589       YES     YES
   106        a            2999.38        21.29395       YES     YES
   107        a            3000.54        20.01681       YES     YES
   108        a            3938.93        35.43120       YES     YES
$end

Total COSMO energy + OC correction = -2686.4583173086 H
```

**HOhfip**

```
Method: (RI-)B3LYP(D3BJ)/def2-TZVPP
Symmetry: cs

Cartesian coordinates in Ångström:
F     0.2160340   -1.3824187    1.4221858
F     0.2160340   -1.3824187   -1.4221858
H    -1.7861925   -0.8136868    0.0000000
C     0.3553527   -0.0486129    1.2918868
C     0.3553527   -0.0486129   -1.2918868
F     1.6615837    0.2409685    1.3403382
F     1.6615837    0.2409685   -1.3403382
O    -1.6753694    0.1444764    0.0000000
C    -0.3138445    0.4562734    0.0000000
F    -0.2324313    0.5252562    2.3512505
F    -0.2324313    0.5252562   -2.3512505
H    -0.2256093    1.5425194    0.0000000

SCF energy GEOOPT =  -789.8666264350 H
ZPE =   162.6 kJ/mol
FREEH energy =   186.60 kJ/mol
FREEH entropy =   0.40139 kJ/mol/K

$vibrational spectrum
#  mode     symmetry     wave number   IR intensity    selection rules
#                         cm**(-1)        km/mol         IR     RAMAN
     1                       -0.00         0.00000        -       -
     2                       -0.00         0.00000        -       -
     3                       -0.00         0.00000        -       -
     4                       -0.00         0.00000        -       -
     5                        0.00         0.00000        -       -
     6                        0.00         0.00000        -       -
     7        a"             27.28         0.11147       YES     YES
     8        a'             90.01         0.30922       YES     YES
     9        a'            166.57         0.86336       YES     YES
    10        a'            227.74         6.75016       YES     YES
    11        a"            247.10         1.57910       YES     YES
    12        a"            287.48        13.02808       YES     YES
    13        a'            322.01         0.41388       YES     YES
    14        a"            339.38        15.75599       YES     YES
    15        a"            379.69        94.44921       YES     YES
    16        a'            455.78         8.51696       YES     YES
    17        a'            510.36         6.24578       YES     YES
    18        a"            528.05         2.94163       YES     YES
    19        a"            547.72         3.40427       YES     YES
    20        a'            603.71         2.16204       YES     YES
    21        a"            685.03        53.06137       YES     YES
    22        a'            734.12        12.34966       YES     YES
    23        a'            827.65        37.24656       YES     YES
    24        a"            892.68        46.30611       YES     YES
    25        a"           1097.49       148.35459       YES     YES
    26        a'           1112.29       120.57996       YES     YES
    27        a"           1162.37        19.53940       YES     YES
    28        a'           1191.77       380.61565       YES     YES
    29        a'           1220.24       302.67889       YES     YES
    30        a"           1246.36       146.56828       YES     YES
    31        a'           1270.83       132.88567       YES     YES
    32        a'           1307.73       129.09798       YES     YES
    33        a"           1378.35        87.97052       YES     YES
    34        a'           1426.47        11.73967       YES     YES
    35        a'           3102.75         3.49699       YES     YES
    36        a'           3790.14        60.92739       YES     YES
$end

Total COSMO energy + OC correction = -789.8734887128 H
```

**[H–MOEA]^+^**

```
Method: (RI-)B3LYP(D3BJ)/def2-TZVPP
Symmetry: c1

Cartesian coordinates in Ångström:
C    -2.5150209    0.7135518   -0.0797191
O    -1.1191763    0.9784091   -0.2698837
H    -3.0463448    1.6135319   -0.3731444
H    -2.7231402    0.4853543    0.9680309
H    -2.8301018   -0.1206496   -0.7087870
C    -0.2692721   -0.0889841    0.0866739
H    -0.3614130   -0.3252988    1.1547317
C     1.1321267    0.3838114   -0.2586815
H    -0.4888394   -0.9980814   -0.4808605
H     1.2619870    0.4616576   -1.3351822
H     1.9137350   -0.2395515    0.1662926
N     1.2707388    1.7908267    0.2863655
H     0.3684624    2.2514881    0.0896435
H     2.0323309    2.3103607   -0.1490169
H     1.4140977    1.7974837    1.2971671

SCF energy GEOOPT =  -250.0320973860 H
ZPE =   370.0 kJ/mol
FREEH energy =   387.49 kJ/mol
FREEH entropy =   0.33066 kJ/mol/K

$vibrational spectrum
#  mode     symmetry     wave number   IR intensity    selection rules
#                         cm**(-1)        km/mol         IR     RAMAN
     1                       -0.00         0.00000        -       -
     2                        0.00         0.00000        -       -
     3                        0.00         0.00000        -       -
     4                        0.00         0.00000        -       -
     5                        0.00         0.00000        -       -
     6                        0.00         0.00000        -       -
     7        a              96.33        10.77039       YES     YES
     8        a             159.00        14.00838       YES     YES
     9        a             209.18        17.19917       YES     YES
    10        a             240.38         2.78599       YES     YES
    11        a             278.69         4.08932       YES     YES
    12        a             372.20        25.75187       YES     YES
    13        a             528.91         6.37732       YES     YES
    14        a             804.29        17.39551       YES     YES
    15        a             859.81        11.89846       YES     YES
    16        a             946.16        14.05136       YES     YES
    17        a             969.70         9.40264       YES     YES
    18        a            1011.74        24.32826       YES     YES
    19        a            1099.28       117.89894       YES     YES
    20        a            1132.43       118.94330       YES     YES
    21        a            1165.30         2.66893       YES     YES
    22        a            1192.68         2.79108       YES     YES
    23        a            1228.43        12.75558       YES     YES
    24        a            1276.83         5.11818       YES     YES
    25        a            1340.65        15.38595       YES     YES
    26        a            1394.92         6.19099       YES     YES
    27        a            1426.64         3.42309       YES     YES
    28        a            1472.35       104.26216       YES     YES
    29        a            1489.09        25.00802       YES     YES
    30        a            1493.12        37.20615       YES     YES
    31        a            1497.79        14.07031       YES     YES
    32        a            1504.02        24.97574       YES     YES
    33        a            1525.37        13.57105       YES     YES
    34        a            1644.75        31.64745       YES     YES
    35        a            1672.52        34.31706       YES     YES
    36        a            2987.69        25.35696       YES     YES
    37        a            3024.46        26.90384       YES     YES
    38        a            3048.37        10.01101       YES     YES
    39        a            3091.34        18.51071       YES     YES
    40        a            3101.91         0.96281       YES     YES
    41        a            3156.61         4.77914       YES     YES
    42        a            3163.35         0.28883       YES     YES
    43        a            3293.89       134.15204       YES     YES
    44        a            3452.20       108.68011       YES     YES
    45        a            3505.00       100.47359       YES     YES
$end

Total COSMO energy + OC correction = -250.1140116845 H
```

**[OH]^−^**

```
Method: (RI-)B3LYP(D3BJ)/def2-TZVPP
Symmetry: c6v

Cartesian coordinates in Ångström:
O     0.0000000    0.0000000    0.4840151
H     0.0000000    0.0000000   -0.4840151

SCF energy GEOOPT =    -75.776424081 H
ZPE =   21.92 kJ/mol
FREEH energy =    28.11 kJ/mol
FREEH entropy =   0.17254 kJ/mol/K

$vibrational spectrum
#  mode     symmetry     wave number   IR intensity    selection rules
#                         cm**(-1)        km/mol         IR     RAMAN
     1                        0.00         0.00000        -       -
     2                        0.00         0.00000        -       -
     3                        0.00         0.00000        -       -
     4                        0.00         0.00000        -       -
     5                        0.00         0.00000        -       -
     6        a1           3664.18       205.87256       YES     YES
$end

Total COSMO energy + OC correction = -75.9053013571 H
```

**[Al(Ohfip)_4_OH]^2−^**

```
Method: (RI-)B3LYP(D3BJ)/def2-TZVPP
Symmetry: c1

Cartesian coordinates in Ångström:
Al   -0.0659310   -0.0851315    0.6921038
O     0.8549462    1.5499250    0.4669459
O     1.3891427   -0.9612802   -0.0121414
O    -1.4385965    0.6345199   -0.2963205
O    -0.9785559   -1.7924327    0.6135324
C     2.5817320   -0.4613872   -0.4041586
H     2.6429461    0.6334045   -0.3294335
C    -2.5829991    0.0705306   -0.7367590
F    -2.8018062    1.5499296   -2.6444598
C    -0.4370434   -3.0189389    0.5151938
C     0.3233461    2.7683705    0.2681038
F    -3.7110331    0.3938605    1.3221527
C    -3.7944442    0.6643094    0.0104184
F    -4.9869688    0.1528069   -0.4113239
F    -3.8981516    2.0063157   -0.1084108
C    -2.7098933    0.2564680   -2.2623618
F    -3.8049608   -0.3705740   -2.7809625
F    -1.6447119   -0.2637369   -2.8855405
H    -2.6349077   -1.0132265   -0.5613451
F     4.9427504   -0.5458624    0.1460794
C     3.7072684   -1.0036661    0.4987305
F     3.7940177   -2.3536417    0.5014680
F     3.5108873   -0.6235390    1.7692359
F     2.9033453   -2.1234703   -2.1381743
C     2.8314529   -0.7962989   -1.8883516
F     1.8448185   -0.3058356   -2.6489688
F     3.9910633   -0.2579242   -2.3638259
F    -0.5315473   -3.0028303   -1.8546678
C    -0.9014261   -3.7165996   -0.7837385
F    -0.3716862   -4.9632833   -0.9474915
F    -2.2456428   -3.8682347   -0.8588662
F     0.2240500    4.5453167   -1.3915135
C     0.8146026    3.3572721   -1.0758586
F     2.1516736    3.5841548   -1.1045826
F     0.5432437    2.5202508   -2.0842394
H    -0.7749364    2.7757363    0.1900681
F     0.0943058    3.2737009    2.5771065
C     0.6600556    3.7110246    1.4456497
F     1.9850475    3.8258933    1.6810427
F     0.1996132    4.9868220    1.2578127
F    -0.2153766   -5.1071529    1.7446779
H     0.6627672   -3.0217870    0.4569902
C    -0.7863281   -3.8689438    1.7569527
F    -2.1120762   -4.0682863    1.9272331
F    -0.3402672   -3.2648954    2.8751455
O    -0.1930518    0.0507513    2.4500667
H    -0.6379140   -0.7168340    2.8094259

SCF energy GEOOPT =  -3475.676896643 H
ZPE =   570.6 kJ/mol
FREEH energy =   683.29 kJ/mol
FREEH entropy =   1.16393 kJ/mol/K

$vibrational spectrum
#  mode     symmetry     wave number   IR intensity    selection rules
#                         cm**(-1)        km/mol         IR     RAMAN
     1                       -0.00         0.00000        -       -
     2                       -0.00         0.00000        -       -
     3                       -0.00         0.00000        -       -
     4                       -0.00         0.00000        -       -
     5                       -0.00         0.00000        -       -
     6                        0.00         0.00000        -       -
     7        a               9.63         0.11152       YES     YES
     8        a              11.85         0.25530       YES     YES
     9        a              13.75         0.05513       YES     YES
    10        a              15.78         0.07893       YES     YES
    11        a              17.12         0.05417       YES     YES
    12        a              21.59         0.22958       YES     YES
    13        a              24.17         0.12719       YES     YES
    14        a              26.09         0.02952       YES     YES
    15        a              28.46         0.04379       YES     YES
    16        a              30.42         0.09827       YES     YES
    17        a              33.19         0.44762       YES     YES
    18        a              34.32         0.26660       YES     YES
    19        a              35.80         0.26662       YES     YES
    20        a              36.31         0.23326       YES     YES
    21        a              53.97         0.04687       YES     YES
    22        a              55.32         0.24774       YES     YES
    23        a              60.66         0.03732       YES     YES
    24        a              68.94         0.10676       YES     YES
    25        a              70.41         0.03555       YES     YES
    26        a              79.30         0.18974       YES     YES
    27        a              90.99         0.19073       YES     YES
    28        a             100.44         0.03876       YES     YES
    29        a             126.86         0.77108       YES     YES
    30        a             151.53         0.21808       YES     YES
    31        a             160.10         1.10227       YES     YES
    32        a             161.89         0.11354       YES     YES
    33        a             178.55         0.31247       YES     YES
    34        a             187.39         0.53458       YES     YES
    35        a             190.82         1.48115       YES     YES
    36        a             201.06         0.03730       YES     YES
    37        a             206.15         2.92060       YES     YES
    38        a             214.73         0.09424       YES     YES
    39        a             229.30         0.41525       YES     YES
    40        a             262.81         0.15064       YES     YES
    41        a             263.82         0.22050       YES     YES
    42        a             274.73         0.28162       YES     YES
    43        a             285.67         3.74093       YES     YES
    44        a             287.17         0.04504       YES     YES
    45        a             288.33         0.75124       YES     YES
    46        a             288.51         0.10976       YES     YES
    47        a             295.36        10.39876       YES     YES
    48        a             301.19        20.11083       YES     YES
    49        a             309.42         6.03057       YES     YES
    50        a             322.20         0.07401       YES     YES
    51        a             324.95         0.05153       YES     YES
    52        a             328.72         0.24884       YES     YES
    53        a             335.23         0.79134       YES     YES
    54        a             349.13         3.93157       YES     YES
    55        a             354.16         0.21999       YES     YES
    56        a             355.62         7.33344       YES     YES
    57        a             367.72        14.30833       YES     YES
    58        a             381.35        10.76824       YES     YES
    59        a             401.16        17.10811       YES     YES
    60        a             412.01        17.34679       YES     YES
    61        a             432.82       147.78939       YES     YES
    62        a             456.08       120.79406       YES     YES
    63        a             493.00         0.38796       YES     YES
    64        a             514.45         1.25062       YES     YES
    65        a             516.01         0.45433       YES     YES
    66        a             516.83        13.29402       YES     YES
    67        a             517.84        10.61621       YES     YES
    68        a             520.39         3.15669       YES     YES
    69        a             522.06         6.62272       YES     YES
    70        a             522.42         1.75788       YES     YES
    71        a             523.78         1.68327       YES     YES
    72        a             524.01         6.86517       YES     YES
    73        a             525.05        73.13817       YES     YES
    74        a             540.38         0.65300       YES     YES
    75        a             540.88         0.57390       YES     YES
    76        a             541.64         0.37753       YES     YES
    77        a             541.99         0.39094       YES     YES
    78        a             550.91        90.55659       YES     YES
    79        a             626.44         2.29743       YES     YES
    80        a             648.30         6.51427       YES     YES
    81        a             656.67        12.46195       YES     YES
    82        a             676.66        12.64293       YES     YES
    83        a             676.77        10.12608       YES     YES
    84        a             678.28        14.26963       YES     YES
    85        a             679.44        88.11573       YES     YES
    86        a             685.94        10.58876       YES     YES
    87        a             722.29       103.08637       YES     YES
    88        a             734.13        13.29461       YES     YES
    89        a             736.50        23.42519       YES     YES
    90        a             744.22        46.52305       YES     YES
    91        a             744.90        49.73608       YES     YES
    92        a             815.99       144.39263       YES     YES
    93        a             844.20         1.09874       YES     YES
    94        a             845.53       229.37270       YES     YES
    95        a             848.82        16.60440       YES     YES
    96        a             853.13       286.42375       YES     YES
    97        a             865.47        17.38511       YES     YES
    98        a             865.99        46.71752       YES     YES
    99        a             873.51        17.00592       YES     YES
   100        a             874.30        66.60207       YES     YES
   101        a            1054.61        42.54181       YES     YES
   102        a            1059.33        60.14324       YES     YES
   103        a            1063.56        18.28412       YES     YES
   104        a            1063.90        23.68560       YES     YES
   105        a            1090.14        18.20356       YES     YES
   106        a            1093.97        23.98121       YES     YES
   107        a            1098.67        23.29181       YES     YES
   108        a            1109.07       324.50154       YES     YES
   109        a            1125.70        30.91532       YES     YES
   110        a            1127.26       856.49478       YES     YES
   111        a            1130.79       925.94940       YES     YES
   112        a            1131.66        64.02077       YES     YES
   113        a            1190.49       345.49199       YES     YES
   114        a            1197.43       403.03760       YES     YES
   115        a            1200.61       512.34663       YES     YES
   116        a            1206.47       168.16602       YES     YES
   117        a            1212.61        36.80743       YES     YES
   118        a            1219.00       517.60713       YES     YES
   119        a            1223.91       504.31794       YES     YES
   120        a            1238.50        64.97948       YES     YES
   121        a            1239.63       168.24728       YES     YES
   122        a            1244.84        75.35201       YES     YES
   123        a            1247.05       412.33103       YES     YES
   124        a            1249.60        36.43772       YES     YES
   125        a            1272.12        24.52569       YES     YES
   126        a            1275.84       119.27712       YES     YES
   127        a            1281.91       123.69517       YES     YES
   128        a            1283.45         2.38765       YES     YES
   129        a            1329.62         9.15873       YES     YES
   130        a            1343.34         1.18412       YES     YES
   131        a            1347.54         0.07549       YES     YES
   132        a            1358.39        23.71273       YES     YES
   133        a            1385.09        19.28060       YES     YES
   134        a            1386.67        36.15012       YES     YES
   135        a            1397.24        13.51446       YES     YES
   136        a            1397.83        56.91918       YES     YES
   137        a            2963.95        48.20391       YES     YES
   138        a            2966.53        36.09129       YES     YES
   139        a            2990.63        74.39997       YES     YES
   140        a            2991.30        14.48192       YES     YES
   141        a            3896.88         6.13050       YES     YES
$end

Total COSMO energy + OC correction = -3475.8429620786 H
```

**[Ohfip]^−^**

```
Method: (RI-)B3LYP(D3BJ)/def2-TZVPP
Symmetry: cs

Cartesian coordinates in Ångström:
F     0.0967278   -1.4718214    1.4313886
F     0.0967278   -1.4718214   -1.4313886
C     0.1615594   -0.1304857    1.2778167
C     0.1615594   -0.1304857   -1.2778167
F     1.4927043    0.1907166    1.3569983
F     1.4927043    0.1907166   -1.3569983
O    -1.8482578    0.1152877    0.0000000
C    -0.5769492    0.3980635    0.0000000
F    -0.3998017    0.4093422    2.3859884
F    -0.3998017    0.4093422   -2.3859884
H    -0.2771726    1.4911452    0.0000000

SCF energy GEOOPT =  -789.3013746030 H
ZPE =   123.4 kJ/mol
FREEH energy =   146.92 kJ/mol
FREEH entropy =   0.39928 kJ/mol/K

$vibrational spectrum
#  mode     symmetry     wave number   IR intensity    selection rules
#                         cm**(-1)        km/mol         IR     RAMAN
     1                       -0.00         0.00000        -       -
     2                       -0.00         0.00000        -       -
     3                       -0.00         0.00000        -       -
     4                       -0.00         0.00000        -       -
     5                       -0.00         0.00000        -       -
     6                       -0.00         0.00000        -       -
     7        a"             27.68         0.00011       YES     YES
     8        a'             86.60         3.10160       YES     YES
     9        a'            161.82         0.73325       YES     YES
    10        a'            232.54         6.21726       YES     YES
    11        a"            244.11         9.10177       YES     YES
    12        a"            273.44         0.11651       YES     YES
    13        a'            305.87         0.82984       YES     YES
    14        a"            334.07         2.49081       YES     YES
    15        a'            449.94         0.74299       YES     YES
    16        a'            506.23         4.97791       YES     YES
    17        a"            511.81         5.17226       YES     YES
    18        a"            531.27         0.42257       YES     YES
    19        a'            583.63         3.83232       YES     YES
    20        a"            672.36        38.68570       YES     YES
    21        a'            719.65         8.20770       YES     YES
    22        a'            814.01        61.22463       YES     YES
    23        a"            833.74        50.04651       YES     YES
    24        a"           1033.33         5.71850       YES     YES
    25        a"           1058.56       193.67384       YES     YES
    26        a'           1097.70       432.50605       YES     YES
    27        a'           1156.73       265.76538       YES     YES
    28        a"           1213.36       199.94938       YES     YES
    29        a'           1233.61       121.04388       YES     YES
    30        a'           1272.27       221.93660       YES     YES
    31        a"           1328.51         3.97823       YES     YES
    32        a'           1358.18        77.44442       YES     YES
    33        a'           2588.72       315.11200       YES     YES
$end

Total COSMO energy + OC correction = -789.3785506665 H
```

**[(Ohfip)_3_Al(OH)_2_Al(Ohfip)_3_]^2−^**

```
Method: (RI-)B3LYP(D3BJ)/def2-TZVPP
Symmetry: c1

Cartesian coordinates in Ångström:
Al    0.4654640    0.1123935    0.1563624
O     0.4825832    1.9352147    0.0664437
O     1.9274483    0.1645606    1.2703638
O    -1.2515343    0.0642845    0.6713819
O     0.7399055   -0.1868757   -1.5804121
C    -2.1390096   -0.9424028    0.8320693
F    -4.0295850    0.4177360    0.1959724
C     1.2678122   -1.2494747   -2.2305589
C    -0.5529976    2.8026318    0.1249797
F    -1.4722099   -1.3516569    3.0590367
C    -2.5455825   -1.0621634    2.3168971
F    -3.4525104   -2.0509240    2.5258658
F    -3.0848324    0.0711176    2.8041792
C    -3.3552247   -0.7133652   -0.0909275
F    -4.2523767   -1.7303838   -0.0328603
F    -2.9534500   -0.6246404   -1.3664222
H    -1.7628071   -1.9397350    0.5610822
F     3.2358626   -0.0297841   -2.7035427
C     2.2643894   -0.7476053   -3.2953689
F     2.8746520   -1.7579169   -3.9603453
F     1.6958211    0.0424321   -4.2248362
F    -1.9213872    4.2434953   -1.2560738
C    -0.8994201    3.3424114   -1.2793106
F     0.1351770    3.9529099   -1.8879172
F    -1.2941766    2.3369013   -2.0721065
H    -1.4858201    2.3563417    0.4993614
F     0.0843128    3.4268933    2.3164987
C    -0.2185165    3.9394359    1.1111498
F     0.8342006    4.6923040    0.7282034
F    -1.2562326    4.7937821    1.3041440
F     0.6352169   -3.1984194   -3.5143297
H     1.8423065   -1.9385339   -1.5950232
C     0.1533348   -2.1195666   -2.8500758
F    -0.6367011   -1.4519506   -3.7045580
F    -0.6419960   -2.6032262   -1.8697236
H     2.2300505    1.0286272    1.5401865
F     1.3931348    1.6335434    5.7014334
F     0.4898741   -0.3379379    5.8132278
C     1.1145157    0.5154266    4.9817929
F     0.2414848    0.8703213    4.0328042
F     3.8984472    0.6944895    6.0348901
F     3.0741110   -1.2966694    6.3053940
C     3.4735796   -0.4105050    5.3710901
C     2.3757044   -0.0935991    4.3321704
H     2.7975520    0.7155164    3.7180330
F     4.8611485    0.7173913    2.1122942
O     2.0546182   -1.2174773    3.6526460
F     4.5460478   -0.9319564    4.7607607
C     5.5501503   -0.0868103    1.2717416
F     6.4238736   -0.7794382    2.0182711
F     6.2737922    0.7329069    0.4710664
O     0.8351620   -1.7338253    0.6728499
Al    2.1850497   -1.6603427    1.9186311
H     0.4021394   -2.5752182    0.5492086
F    -0.4119607   -4.2240380    3.4142400
O     3.8631631   -1.7724777    1.3246584
H     1.9222416   -3.4300316    4.3145482
C     4.5665665   -0.9789407    0.4843847
H     3.9574273   -0.2766534   -0.1027315
C     1.9503802   -4.0933123    3.4378564
O     1.9349852   -3.4367161    2.2557414
C     0.6922461   -4.9771151    3.5538231
F     0.5896408   -5.5951976    4.7586617
C     5.2953814   -1.8580830   -0.5525148
F     4.3101172   -4.0991076    3.5916813
C     3.2476234   -4.9152470    3.5924832
F     3.2909716   -5.6092301    4.7641175
F     6.1648518   -2.7227543    0.0018780
F     5.9900797   -1.1320257   -1.4607568
F     0.6282946   -5.9466666    2.6168172
F     4.3969811   -2.5848831   -1.2414478
F     3.4308268   -5.8157536    2.6075089

SCF energy GEOOPT =  -5372.786721338 H
ZPE =   889.0 kJ/mol
FREEH energy =  1062.31 kJ/mol
FREEH entropy =   1.66116 kJ/mol/K

$vibrational spectrum
#  mode     symmetry     wave number   IR intensity    selection rules
#                         cm**(-1)        km/mol         IR     RAMAN
     1                       -0.00         0.00000        -       -
     2                        0.00         0.00000        -       -
     3                        0.00         0.00000        -       -
     4                        0.00         0.00000        -       -
     5                        0.00         0.00000        -       -
     6                        0.00         0.00000        -       -
     7        a               4.62         0.16562       YES     YES
     8        a               7.19         0.07248       YES     YES
     9        a               8.82         0.05003       YES     YES
    10        a              12.45         0.00487       YES     YES
    11        a              14.81         0.02092       YES     YES
    12        a              17.50         0.04090       YES     YES
    13        a              19.48         0.06234       YES     YES
    14        a              20.67         0.05368       YES     YES
    15        a              22.55         0.00478       YES     YES
    16        a              23.51         0.07639       YES     YES
    17        a              25.26         0.07042       YES     YES
    18        a              26.03         0.02191       YES     YES
    19        a              26.67         0.01928       YES     YES
    20        a              27.83         0.01323       YES     YES
    21        a              29.78         0.10645       YES     YES
    22        a              30.39         0.08832       YES     YES
    23        a              32.39         0.14149       YES     YES
    24        a              35.15         0.04681       YES     YES
    25        a              37.67         0.04732       YES     YES
    26        a              38.48         0.37359       YES     YES
    27        a              40.63         0.03822       YES     YES
    28        a              42.04         0.12923       YES     YES
    29        a              45.61         0.15436       YES     YES
    30        a              49.60         0.14230       YES     YES
    31        a              53.42         0.04667       YES     YES
    32        a              59.81         0.19963       YES     YES
    33        a              62.83         0.00746       YES     YES
    34        a              67.29         0.10364       YES     YES
    35        a              69.81         0.02215       YES     YES
    36        a              72.46         0.06399       YES     YES
    37        a              73.22         0.01472       YES     YES
    38        a              78.22         0.01783       YES     YES
    39        a              80.28         0.09145       YES     YES
    40        a              84.84         0.06970       YES     YES
    41        a             100.14         0.42219       YES     YES
    42        a             101.89         0.07258       YES     YES
    43        a             134.27         0.03433       YES     YES
    44        a             141.72         4.32142       YES     YES
    45        a             142.87         1.58677       YES     YES
    46        a             151.89         1.12834       YES     YES
    47        a             154.36         0.39687       YES     YES
    48        a             171.29         0.52783       YES     YES
    49        a             173.91         2.11063       YES     YES
    50        a             181.03         0.22887       YES     YES
    51        a             185.82         0.08739       YES     YES
    52        a             191.04         1.43562       YES     YES
    53        a             192.01         0.18771       YES     YES
    54        a             200.84         0.87589       YES     YES
    55        a             203.94         0.10199       YES     YES
    56        a             210.58         2.78532       YES     YES
    57        a             216.53         2.32993       YES     YES
    58        a             250.07         3.01937       YES     YES
    59        a             254.76         1.62308       YES     YES
    60        a             260.35         3.31394       YES     YES
    61        a             265.22         0.21754       YES     YES
    62        a             268.47         1.02264       YES     YES
    63        a             269.09         0.81690       YES     YES
    64        a             281.74        20.73094       YES     YES
    65        a             289.26         0.03371       YES     YES
    66        a             289.27         0.26148       YES     YES
    67        a             289.35         0.18041       YES     YES
    68        a             289.53         0.10138       YES     YES
    69        a             290.85         0.19462       YES     YES
    70        a             291.18         0.24347       YES     YES
    71        a             299.49         0.99972       YES     YES
    72        a             309.36         2.62305       YES     YES
    73        a             319.08         2.80535       YES     YES
    74        a             322.27         9.17425       YES     YES
    75        a             324.06         1.72412       YES     YES
    76        a             325.25         1.54073       YES     YES
    77        a             326.63         0.07395       YES     YES
    78        a             328.54         0.13026       YES     YES
    79        a             330.30         1.05422       YES     YES
    80        a             337.16         1.12828       YES     YES
    81        a             341.46         0.95358       YES     YES
    82        a             346.15         8.05493       YES     YES
    83        a             346.82         2.44647       YES     YES
    84        a             365.80         1.04195       YES     YES
    85        a             368.85         8.91419       YES     YES
    86        a             370.10         0.62472       YES     YES
    87        a             373.10         6.64196       YES     YES
    88        a             382.92         7.67119       YES     YES
    89        a             402.28         0.97044       YES     YES
    90        a             414.89        13.98310       YES     YES
    91        a             420.08       117.74472       YES     YES
    92        a             437.95         3.08781       YES     YES
    93        a             442.90        44.88358       YES     YES
    94        a             457.89       195.94925       YES     YES
    95        a             467.65       101.10417       YES     YES
    96        a             487.62        48.01687       YES     YES
    97        a             515.80        16.15994       YES     YES
    98        a             516.70         0.85486       YES     YES
    99        a             516.80        16.06334       YES     YES
   100        a             517.08        12.02013       YES     YES
   101        a             517.33         8.12500       YES     YES
   102        a             517.52         1.87032       YES     YES
   103        a             525.03         1.56868       YES     YES
   104        a             525.11         4.21028       YES     YES
   105        a             525.67         4.83077       YES     YES
   106        a             526.07         2.77122       YES     YES
   107        a             527.49         2.45510       YES     YES
   108        a             527.70         1.55088       YES     YES
   109        a             530.78         2.32274       YES     YES
   110        a             537.81        97.02850       YES     YES
   111        a             540.79         0.18133       YES     YES
   112        a             541.34         1.30736       YES     YES
   113        a             542.38         0.64404       YES     YES
   114        a             542.50         0.46893       YES     YES
   115        a             542.60         0.40024       YES     YES
   116        a             543.53         3.73210       YES     YES
   117        a             543.75         0.90891       YES     YES
   118        a             555.36         0.68419       YES     YES
   119        a             556.88         0.53452       YES     YES
   120        a             561.05       103.74041       YES     YES
   121        a             621.59       136.83631       YES     YES
   122        a             645.87         0.09708       YES     YES
   123        a             677.22        13.83570       YES     YES
   124        a             678.16         6.67349       YES     YES
   125        a             680.13        47.62168       YES     YES
   126        a             681.64        45.55937       YES     YES
   127        a             681.81        35.71257       YES     YES
   128        a             683.05         1.17882       YES     YES
   129        a             683.76        71.46552       YES     YES
   130        a             684.95        12.49685       YES     YES
   131        a             699.90         0.99301       YES     YES
   132        a             707.56         1.24461       YES     YES
   133        a             708.75        66.67115       YES     YES
   134        a             714.00        16.98492       YES     YES
   135        a             745.99         5.11053       YES     YES
   136        a             746.74        70.75983       YES     YES
   137        a             753.50        67.83827       YES     YES
   138        a             757.98         0.14959       YES     YES
   139        a             764.59        10.46499       YES     YES
   140        a             768.04       118.53861       YES     YES
   141        a             842.92        15.16968       YES     YES
   142        a             849.79       225.34700       YES     YES
   143        a             851.35        32.04028       YES     YES
   144        a             852.63        41.56316       YES     YES
   145        a             853.50       258.67216       YES     YES
   146        a             856.41        68.53891       YES     YES
   147        a             871.49       132.49189       YES     YES
   148        a             877.22        46.01895       YES     YES
   149        a             877.81        32.38002       YES     YES
   150        a             880.02       179.36866       YES     YES
   151        a             881.23        33.59113       YES     YES
   152        a             882.09         9.70620       YES     YES
   153        a             885.40        23.91817       YES     YES
   154        a             892.04       449.68522       YES     YES
   155        a            1075.44        38.40291       YES     YES
   156        a            1075.81        32.48084       YES     YES
   157        a            1076.07       141.11872       YES     YES
   158        a            1080.23        70.10214       YES     YES
   159        a            1082.10        31.89132       YES     YES
   160        a            1082.76       106.42609       YES     YES
   161        a            1099.51        24.27582       YES     YES
   162        a            1101.33        19.75812       YES     YES
   163        a            1101.84        35.14776       YES     YES
   164        a            1102.64        48.06084       YES     YES
   165        a            1111.99       192.33019       YES     YES
   166        a            1121.31       175.82266       YES     YES
   167        a            1144.40       574.51050       YES     YES
   168        a            1144.84       440.40210       YES     YES
   169        a            1151.35       594.40370       YES     YES
   170        a            1151.44        27.54516       YES     YES
   171        a            1157.22       341.49431       YES     YES
   172        a            1161.23       814.75726       YES     YES
   173        a            1186.49       311.23670       YES     YES
   174        a            1187.42       157.83864       YES     YES
   175        a            1199.78        96.55894       YES     YES
   176        a            1202.70       433.59696       YES     YES
   177        a            1210.45       307.66164       YES     YES
   178        a            1214.27         7.17084       YES     YES
   179        a            1216.02       651.88683       YES     YES
   180        a            1216.97       209.50087       YES     YES
   181        a            1218.78       340.83694       YES     YES
   182        a            1220.27       929.86432       YES     YES
   183        a            1240.90       171.37663       YES     YES
   184        a            1247.01       210.72902       YES     YES
   185        a            1248.75        67.92854       YES     YES
   186        a            1249.24       262.64689       YES     YES
   187        a            1250.31       126.94453       YES     YES
   188        a            1253.08       150.07880       YES     YES
   189        a            1254.38       250.62416       YES     YES
   190        a            1254.58        18.06391       YES     YES
   191        a            1281.60         7.90079       YES     YES
   192        a            1282.06       163.95685       YES     YES
   193        a            1284.44       302.72689       YES     YES
   194        a            1286.68        36.44490       YES     YES
   195        a            1287.00         8.17194       YES     YES
   196        a            1287.25       129.03820       YES     YES
   197        a            1363.37         3.87026       YES     YES
   198        a            1364.16         0.84539       YES     YES
   199        a            1373.22        59.45946       YES     YES
   200        a            1375.02        24.23560       YES     YES
   201        a            1375.87        72.19321       YES     YES
   202        a            1376.22         3.88113       YES     YES
   203        a            1386.42        18.03188       YES     YES
   204        a            1388.43        14.80726       YES     YES
   205        a            1391.04        38.97899       YES     YES
   206        a            1391.27        32.48705       YES     YES
   207        a            1394.62        15.74280       YES     YES
   208        a            1399.09        27.10661       YES     YES
   209        a            2976.41         0.54029       YES     YES
   210        a            2976.68        71.34143       YES     YES
   211        a            2982.88        34.91942       YES     YES
   212        a            2984.06        34.26570       YES     YES
   213        a            2986.60        20.60008       YES     YES
   214        a            2988.07        20.21368       YES     YES
   215        a            3942.12         5.68474       YES     YES
   216        a            3945.68        82.10241       YES     YES
$end

Total COSMO energy + OC correction = -5372.9292750812 H
```

**[Mg(MOEA)_3_]^2+^**

```
Method: (RI-)B3LYP(D3BJ)/def2-TZVPP
Symmetry: c1

Cartesian coordinates in Ångström:
C     0.3195855    2.2889266   -1.9683605
O     0.4457639    1.7137685   -0.6551137
H     1.2945722    2.6215697   -2.3235904
H    -0.3676071    3.1352428   -1.9467288
H    -0.0743091    1.5288595   -2.6350577
C     0.7962828    2.7118664    0.3292572
H     1.5874699    3.3497520   -0.0655569
C     1.2705161    2.0088392    1.5789314
H    -0.0839951    3.3318336    0.5223884
H     2.2283470    1.5257707    1.3923323
N     0.2905173    0.9528039    1.9473830
H     1.4108735    2.7421276    2.3738087
Mg   -0.0814732   -0.2143219    0.1242464
H    -0.5810097    1.3931539    2.2339325
H     0.6212956    0.4650906    2.7739402
H    -3.2942169    0.6078141   -1.5666459
C    -2.8979541    1.1204131   -0.6888783
H    -3.7176316    1.5607741   -0.1226009
H    -3.2791905   -1.5262045   -0.1618050
H    -2.2072083    1.8992493   -0.9918825
H    -1.0081251   -2.7940167    0.5983823
C    -3.0018355   -0.8592353    0.6604126
O    -2.1685321    0.1991839    0.1440559
N    -0.8930441   -1.9811369    1.1982915
H    -3.9147555   -0.4325747    1.0771998
C    -2.2241568   -1.5854650    1.7334380
H    -2.8010233   -2.4401537    2.0881571
H    -0.3113513   -2.3104946    1.9628491
H    -2.0598961   -0.9235799    2.5829667
H     1.7395172   -0.1350599   -2.4680270
H    -0.5510932   -0.6385520   -2.6063620
C     1.4168693   -1.1563050   -2.2754815
H     1.5832571   -1.7383279   -3.1823080
N    -0.0200899   -1.1161828   -1.8847524
H    -0.3868583   -2.0647846   -1.8888743
C     2.2272150   -1.7445058   -1.1442303
H     3.2935652   -1.6309291   -1.3431780
O     1.8845403   -1.0438294    0.0683093
C     2.7196280   -1.4343623    1.1718823
H     3.7574195   -1.1761856    0.9637366
H     2.0148481   -2.8094890   -1.0067485
H     2.6389334   -2.5079919    1.3488348
H     2.3843400   -0.8933506    2.0514465

SCF energy GEOOPT =  -948.7543943335 H
ZPE =   1025. kJ/mol
FREEH energy =  1083.13 kJ/mol
FREEH entropy =   0.64291 kJ/mol/K

$vibrational spectrum
#  mode     symmetry     wave number   IR intensity    selection rules
#                         cm**(-1)        km/mol         IR     RAMAN
     1                       -0.00         0.00000        -       -
     2                        0.00         0.00000        -       -
     3                        0.00         0.00000        -       -
     4                        0.00         0.00000        -       -
     5                        0.00         0.00000        -       -
     6                        0.00         0.00000        -       -
     7        a              40.82         0.06638       YES     YES
     8        a              48.23         0.00506       YES     YES
     9        a              60.03         0.34153       YES     YES
    10        a              68.78         0.13236       YES     YES
    11        a              84.04         0.43029       YES     YES
    12        a              94.74         0.18658       YES     YES
    13        a             112.60         1.34228       YES     YES
    14        a             120.46         2.59866       YES     YES
    15        a             127.51         0.33955       YES     YES
    16        a             146.94         0.39522       YES     YES
    17        a             148.71         3.92828       YES     YES
    18        a             150.99         1.72163       YES     YES
    19        a             160.73         0.76569       YES     YES
    20        a             170.38         1.69317       YES     YES
    21        a             180.71         0.31928       YES     YES
    22        a             208.70         3.18961       YES     YES
    23        a             214.90         0.83176       YES     YES
    24        a             225.90         3.97943       YES     YES
    25        a             251.89         4.84284       YES     YES
    26        a             261.28        10.58791       YES     YES
    27        a             282.53         3.99892       YES     YES
    28        a             292.38         7.58930       YES     YES
    29        a             306.55         4.31673       YES     YES
    30        a             308.36         3.26427       YES     YES
    31        a             321.17        31.88076       YES     YES
    32        a             337.13        17.01241       YES     YES
    33        a             371.84        15.46330       YES     YES
    34        a             400.65         0.13078       YES     YES
    35        a             407.37        33.41735       YES     YES
    36        a             425.74        25.69722       YES     YES
    37        a             481.86        24.07520       YES     YES
    38        a             500.37        31.42014       YES     YES
    39        a             513.14        35.53693       YES     YES
    40        a             574.34         6.16549       YES     YES
    41        a             596.70        24.69076       YES     YES
    42        a             615.04        63.80602       YES     YES
    43        a             830.06        18.70224       YES     YES
    44        a             833.93        10.90397       YES     YES
    45        a             836.49         0.38917       YES     YES
    46        a             896.64        53.73380       YES     YES
    47        a             900.62        27.19346       YES     YES
    48        a             903.15        16.15413       YES     YES
    49        a             985.51        95.84136       YES     YES
    50        a             986.63       149.58373       YES     YES
    51        a             994.46         2.13804       YES     YES
    52        a            1015.48        15.80920       YES     YES
    53        a            1024.53        25.49718       YES     YES
    54        a            1025.72         3.77593       YES     YES
    55        a            1043.29        29.39745       YES     YES
    56        a            1047.60        36.49035       YES     YES
    57        a            1048.56       173.19843       YES     YES
    58        a            1094.51       116.58924       YES     YES
    59        a            1095.72        57.33838       YES     YES
    60        a            1096.94       193.59806       YES     YES
    61        a            1124.54        67.05666       YES     YES
    62        a            1126.25        31.27382       YES     YES
    63        a            1127.96        67.75268       YES     YES
    64        a            1172.64         1.02604       YES     YES
    65        a            1174.21         2.17335       YES     YES
    66        a            1174.37         0.00664       YES     YES
    67        a            1215.71         2.29964       YES     YES
    68        a            1217.07         2.32566       YES     YES
    69        a            1219.31         5.04889       YES     YES
    70        a            1221.05         4.73196       YES     YES
    71        a            1222.00         3.29345       YES     YES
    72        a            1223.53         7.19413       YES     YES
    73        a            1310.18         7.58590       YES     YES
    74        a            1312.00        11.37165       YES     YES
    75        a            1316.90         8.05495       YES     YES
    76        a            1334.24        14.40137       YES     YES
    77        a            1337.91        10.52018       YES     YES
    78        a            1341.32        12.55604       YES     YES
    79        a            1411.32         6.52537       YES     YES
    80        a            1413.45         3.29909       YES     YES
    81        a            1416.57         8.03043       YES     YES
    82        a            1443.11         0.92969       YES     YES
    83        a            1446.45         1.26900       YES     YES
    84        a            1450.73         0.90423       YES     YES
    85        a            1483.35        16.80457       YES     YES
    86        a            1484.84         0.42097       YES     YES
    87        a            1487.08         2.42759       YES     YES
    88        a            1489.11        11.19396       YES     YES
    89        a            1490.61         4.06746       YES     YES
    90        a            1497.94         2.26820       YES     YES
    91        a            1505.47        10.08140       YES     YES
    92        a            1507.26         8.33240       YES     YES
    93        a            1507.89        23.43280       YES     YES
    94        a            1508.71         2.09168       YES     YES
    95        a            1510.53         6.44539       YES     YES
    96        a            1511.49         4.43761       YES     YES
    97        a            1521.96        17.05759       YES     YES
    98        a            1522.44        18.99139       YES     YES
    99        a            1523.39         2.17781       YES     YES
   100        a            1657.82        48.67290       YES     YES
   101        a            1658.58        43.51533       YES     YES
   102        a            1660.09        43.68279       YES     YES
   103        a            3020.83        14.78634       YES     YES
   104        a            3022.81        15.12780       YES     YES
   105        a            3028.48        12.97343       YES     YES
   106        a            3039.73        15.63040       YES     YES
   107        a            3042.27        13.61821       YES     YES
   108        a            3043.76        13.74961       YES     YES
   109        a            3063.17         5.50878       YES     YES
   110        a            3064.70         5.33946       YES     YES
   111        a            3069.05         4.09161       YES     YES
   112        a            3088.94         6.31480       YES     YES
   113        a            3090.87         5.94355       YES     YES
   114        a            3092.79         5.23233       YES     YES
   115        a            3109.59         9.90306       YES     YES
   116        a            3112.21         7.30005       YES     YES
   117        a            3113.50        12.38095       YES     YES
   118        a            3114.32         4.46804       YES     YES
   119        a            3115.56         4.04121       YES     YES
   120        a            3122.26         6.38586       YES     YES
   121        a            3154.78        16.22335       YES     YES
   122        a            3161.16        18.09470       YES     YES
   123        a            3175.64         6.46535       YES     YES
   124        a            3465.73        12.25912       YES     YES
   125        a            3470.04         8.75312       YES     YES
   126        a            3472.37        13.12477       YES     YES
   127        a            3529.14        14.98634       YES     YES
   128        a            3530.33        16.79073       YES     YES
   129        a            3530.99        20.15746       YES     YES
$end

Total COSMO energy + OC correction = -948.9540772748 H
```

SP energy of [Mg(MOEA)_3_]^+^ with the optimized structure for [Mg(MOEA)_3_]^2+^ = -‍948.9537953267 H (No negative HOMO/LUMO gap is found for the radical cation).

**[(Ohfip)_6_Al_2_(OH)_2_Mg(MOEA)]**

```
Method: (RI-)B3LYP(D3BJ)/def2-TZVPP
Symmetry: c1

Cartesian coordinates in Ångström:
Al    3.4454696   10.6777967    5.8964397
O     4.6844943   11.3558422    6.9028723
O     4.3403387    9.8184838    4.6102725
O     2.6433186    9.3702020    6.7571815
O     2.2839630   11.8229681    5.3502522
C     1.3613675    9.2931073    7.2404521
F     2.1590207    8.3315817    9.2835244
C     1.5384752   12.0427006    4.2241495
C     4.6452235   12.1769681    8.0007920
F     0.6043776    8.1526564    5.3244870
C     0.6853058    8.0375894    6.6654899
F    -0.5473818    7.8358158    7.1305914
F     1.4049050    6.9111026    6.9125279
C     1.3940043    9.3151561    8.7797959
F     0.1748046    9.2134010    9.3245708
F     1.9222020   10.4869728    9.1835981
H     0.7255234   10.1338435    6.9388132
F     3.5067473   12.3756575    2.9774945
C     2.3088087   12.9568118    3.2521381
F     1.6691605   13.1053124    2.0833382
F     2.5612372   14.1670699    3.7522584
F     5.2161731   14.4009012    8.6777643
C     5.2040862   13.5641496    7.6250847
F     6.4513544   13.4982662    7.1359944
F     4.4242347   14.1098809    6.6789128
H     3.6361604   12.3591332    8.3895151
F     4.9160050   10.2815582    9.3837790
C     5.4176391   11.5156396    9.1585456
F     6.7271249   11.3594481    8.8891452
F     5.3117759   12.2052633   10.3012102
F    -0.6147234   12.8528272    3.5748202
H     1.3162500   11.1328879    3.6548045
C     0.1698993   12.6129746    4.6362238
F     0.2675892   13.7387409    5.3484729
F    -0.4647150   11.6989946    5.4066815
H     4.4697013   10.3146499    3.7984846
F     5.9168756    9.4156431    0.1540393
F     6.4875646    9.2130099    2.2388978
C     5.4773364    9.0560038    1.3589990
F     4.5122508    9.9250110    1.7195134
F     5.6993654    6.9018157   -0.7507145
F     7.0926982    6.6908589    0.8999818
C     5.7899147    6.6483435    0.5567854
C     4.9202326    7.6221259    1.3811013
H     3.9609657    7.6648511    0.8566356
F     1.9428690    8.9517547    2.7743712
O     4.7650939    7.1756983    2.6936616
F     5.3635864    5.3907617    0.7625707
C     1.0956747    8.4542327    1.8386283
F     1.6543941    8.6978027    0.6426576
F    -0.0393845    9.1558519    1.9222586
O     3.4028215    6.6633803    4.7429787
Al    3.4297626    5.9954942    3.1178172
H     2.7949836    6.4856420    5.4598494
F     5.9871786    5.0266502    4.7640237
O     2.1022871    6.3045910    2.0802319
H     5.7208721    3.6056100    2.6325658
C     0.8959864    6.9510519    2.1095013
H     0.3891995    6.8940406    3.0796589
C     4.8897913    3.4914956    3.3406533
O     3.8557988    4.3398270    3.0884651
C     5.4989131    3.7490479    4.7335244
F     6.5296917    2.9446360    5.0142396
C    -0.0531981    6.2775030    1.0993507
F     3.9122271    1.8771469    1.9347414
C     4.3961164    2.0388670    3.1724952
F     5.4000097    1.1570769    3.3369943
F     0.4353457    6.2912899   -0.1471373
F    -1.2577586    6.8743776    1.0819754
F     4.6101398    3.6588456    5.7280782
F    -0.2374381    4.9976508    1.4571406
F     3.4259458    1.7270704    4.0421964
H     4.4006793    8.3954415    7.3279200
H     5.1498155    6.9511888    7.2465522
N     5.1647600    7.8962433    6.8761501
H     6.7711459    8.4874242    8.1807141
C     6.4656702    8.5402070    7.1347221
H     6.3692933    9.5918407    6.8730232
Mg    4.9210318    7.9423939    4.7201954
C     7.5101504    7.8720379    6.2711290
H     7.7289007    6.8561713    6.6159635
O     7.0045240    7.7833925    4.9261518
H     8.4371023    8.4482793    6.2690382
C     7.9748474    7.2324255    4.0260525
H     7.4840865    7.0564524    3.0805035
H     8.3515597    6.2899333    4.4246068
H     8.7952886    7.9386236    3.8951382

SCF energy GEOOPT =  -5822.553849920 H
ZPE =   1245. kJ/mol
FREEH energy =  1440.90 kJ/mol
FREEH entropy =   1.79888 kJ/mol/K

$vibrational spectrum
#  mode     symmetry     wave number   IR intensity    selection rules
#                         cm**(-1)        km/mol         IR     RAMAN
     1                       -0.00         0.00000        -       -
     2                       -0.00         0.00000        -       -
     3                        0.00         0.00000        -       -
     4                        0.00         0.00000        -       -
     5                        0.00         0.00000        -       -
     6                        0.00         0.00000        -       -
     7        a               5.63         0.04476       YES     YES
     8        a               9.42         0.29971       YES     YES
     9        a              11.10         0.18814       YES     YES
    10        a              13.34         0.15784       YES     YES
    11        a              16.38         0.14081       YES     YES
    12        a              17.39         0.07984       YES     YES
    13        a              17.72         0.14812       YES     YES
    14        a              21.52         0.27065       YES     YES
    15        a              22.34         0.73928       YES     YES
    16        a              24.69         0.61114       YES     YES
    17        a              25.71         1.12096       YES     YES
    18        a              29.60         0.37032       YES     YES
    19        a              30.52         0.51372       YES     YES
    20        a              32.06         0.18985       YES     YES
    21        a              34.17         0.36294       YES     YES
    22        a              35.36         0.16208       YES     YES
    23        a              37.63         0.03669       YES     YES
    24        a              38.24         0.13437       YES     YES
    25        a              39.30         0.09028       YES     YES
    26        a              41.83         0.55948       YES     YES
    27        a              43.92         0.26570       YES     YES
    28        a              44.93         0.47100       YES     YES
    29        a              48.40         0.22921       YES     YES
    30        a              52.95         0.28132       YES     YES
    31        a              55.94         1.29503       YES     YES
    32        a              57.51         1.20746       YES     YES
    33        a              60.63         0.42816       YES     YES
    34        a              61.34         0.05777       YES     YES
    35        a              66.27         1.09481       YES     YES
    36        a              68.57         0.27458       YES     YES
    37        a              72.56         0.18423       YES     YES
    38        a              74.79         0.41590       YES     YES
    39        a              78.24         1.23631       YES     YES
    40        a              85.01         0.15831       YES     YES
    41        a              86.07         0.32392       YES     YES
    42        a              91.84         1.73357       YES     YES
    43        a              95.41         2.40744       YES     YES
    44        a              97.08         0.69281       YES     YES
    45        a             107.34         5.72344       YES     YES
    46        a             108.45         2.03225       YES     YES
    47        a             113.40         1.82248       YES     YES
    48        a             120.91         3.99497       YES     YES
    49        a             128.58         4.43666       YES     YES
    50        a             133.87         1.43026       YES     YES
    51        a             143.25         4.02299       YES     YES
    52        a             147.74         4.26101       YES     YES
    53        a             154.62         4.43477       YES     YES
    54        a             157.15         0.12253       YES     YES
    55        a             162.29         1.75413       YES     YES
    56        a             163.83         0.83375       YES     YES
    57        a             165.72         2.27616       YES     YES
    58        a             175.82         0.87181       YES     YES
    59        a             177.56         2.69024       YES     YES
    60        a             181.50         0.48295       YES     YES
    61        a             183.32         1.40734       YES     YES
    62        a             190.36         4.23941       YES     YES
    63        a             201.60         2.57937       YES     YES
    64        a             203.70         1.10693       YES     YES
    65        a             227.88         8.72093       YES     YES
    66        a             234.95         2.04883       YES     YES
    67        a             245.67         7.48867       YES     YES
    68        a             250.48         2.47118       YES     YES
    69        a             252.92         0.29921       YES     YES
    70        a             255.79         4.92352       YES     YES
    71        a             258.42         3.77313       YES     YES
    72        a             270.69         8.22607       YES     YES
    73        a             275.36         3.46515       YES     YES
    74        a             284.36         7.86849       YES     YES
    75        a             287.60         0.93290       YES     YES
    76        a             290.25         3.82880       YES     YES
    77        a             291.60         0.76677       YES     YES
    78        a             292.08         3.00148       YES     YES
    79        a             293.85         0.12048       YES     YES
    80        a             294.72         0.29228       YES     YES
    81        a             295.06         4.34039       YES     YES
    82        a             295.81         0.78801       YES     YES
    83        a             296.79         8.08849       YES     YES
    84        a             306.02         5.22103       YES     YES
    85        a             309.98         5.56578       YES     YES
    86        a             315.28        18.14938       YES     YES
    87        a             322.49         3.89971       YES     YES
    88        a             323.67         3.07636       YES     YES
    89        a             324.56         1.60057       YES     YES
    90        a             325.85         1.87551       YES     YES
    91        a             328.47         1.68721       YES     YES
    92        a             328.79         4.50810       YES     YES
    93        a             331.44        15.89975       YES     YES
    94        a             348.66         3.47238       YES     YES
    95        a             351.50         9.56812       YES     YES
    96        a             355.08        33.65026       YES     YES
    97        a             363.09        66.39301       YES     YES
    98        a             370.78        15.91123       YES     YES
    99        a             376.88        26.46183       YES     YES
   100        a             383.90        68.65780       YES     YES
   101        a             386.28        40.95246       YES     YES
   102        a             393.84         3.84475       YES     YES
   103        a             419.95        39.04195       YES     YES
   104        a             424.05         2.44095       YES     YES
   105        a             426.50        69.83156       YES     YES
   106        a             431.09        14.28738       YES     YES
   107        a             443.55        67.20432       YES     YES
   108        a             463.49        38.52357       YES     YES
   109        a             487.12        51.05365       YES     YES
   110        a             509.47        35.48244       YES     YES
   111        a             512.74         4.46575       YES     YES
   112        a             513.15         7.48922       YES     YES
   113        a             514.29         6.81015       YES     YES
   114        a             515.32        19.18705       YES     YES
   115        a             516.49         7.68527       YES     YES
   116        a             520.79        15.82506       YES     YES
   117        a             529.22         2.71087       YES     YES
   118        a             529.77         2.02941       YES     YES
   119        a             530.09         2.76112       YES     YES
   120        a             530.56         3.83257       YES     YES
   121        a             530.66         2.60172       YES     YES
   122        a             533.02         4.44630       YES     YES
   123        a             535.72         2.43005       YES     YES
   124        a             537.79         3.15136       YES     YES
   125        a             543.11         0.96834       YES     YES
   126        a             544.65         0.31212       YES     YES
   127        a             545.80         6.67765       YES     YES
   128        a             546.12         1.75214       YES     YES
   129        a             546.36         0.09752       YES     YES
   130        a             547.61        10.15555       YES     YES
   131        a             550.56        11.59265       YES     YES
   132        a             563.45         4.31518       YES     YES
   133        a             567.94        16.56752       YES     YES
   134        a             569.70        34.95993       YES     YES
   135        a             570.60        81.87663       YES     YES
   136        a             651.43        46.62042       YES     YES
   137        a             676.63        59.38429       YES     YES
   138        a             680.40        43.70732       YES     YES
   139        a             682.95        86.98672       YES     YES
   140        a             683.62        13.82514       YES     YES
   141        a             684.28        74.81892       YES     YES
   142        a             684.50         7.96762       YES     YES
   143        a             684.74        56.86217       YES     YES
   144        a             693.96        41.58825       YES     YES
   145        a             697.37        37.40784       YES     YES
   146        a             717.76        14.88040       YES     YES
   147        a             721.60        31.75107       YES     YES
   148        a             724.50        24.24785       YES     YES
   149        a             726.23        18.57307       YES     YES
   150        a             735.34        43.46186       YES     YES
   151        a             754.43        22.29032       YES     YES
   152        a             775.20        36.05282       YES     YES
   153        a             800.24        71.71811       YES     YES
   154        a             808.15        57.73257       YES     YES
   155        a             827.33        25.22058       YES     YES
   156        a             834.36        48.28204       YES     YES
   157        a             837.48       131.99565       YES     YES
   158        a             848.79        29.33030       YES     YES
   159        a             849.89        30.76128       YES     YES
   160        a             851.94        81.93697       YES     YES
   161        a             856.61        82.35018       YES     YES
   162        a             857.34        16.22693       YES     YES
   163        a             858.46        52.55203       YES     YES
   164        a             859.67       207.02646       YES     YES
   165        a             887.01        52.62740       YES     YES
   166        a             888.30        53.57455       YES     YES
   167        a             890.49        44.91985       YES     YES
   168        a             892.60        44.06403       YES     YES
   169        a             894.32       104.70896       YES     YES
   170        a             898.85       111.93919       YES     YES
   171        a             918.37         9.27707       YES     YES
   172        a             965.63       358.30261       YES     YES
   173        a             995.98       122.70940       YES     YES
   174        a            1023.21        17.21338       YES     YES
   175        a            1061.54       163.73560       YES     YES
   176        a            1064.47        77.91024       YES     YES
   177        a            1071.50       244.89617       YES     YES
   178        a            1073.80       100.65131       YES     YES
   179        a            1078.15        14.43266       YES     YES
   180        a            1082.10       129.63261       YES     YES
   181        a            1089.17       312.01371       YES     YES
   182        a            1104.37        70.98665       YES     YES
   183        a            1106.95        96.61016       YES     YES
   184        a            1141.52        50.72603       YES     YES
   185        a            1144.47        82.75577       YES     YES
   186        a            1146.35        27.11178       YES     YES
   187        a            1148.19        24.28773       YES     YES
   188        a            1148.84         0.11432       YES     YES
   189        a            1160.87        29.80150       YES     YES
   190        a            1167.34       128.46097       YES     YES
   191        a            1170.00        46.45654       YES     YES
   192        a            1173.19       148.75002       YES     YES
   193        a            1182.80        40.02897       YES     YES
   194        a            1182.91       164.31758       YES     YES
   195        a            1184.74        64.38187       YES     YES
   196        a            1190.68       224.66006       YES     YES
   197        a            1191.78       235.34923       YES     YES
   198        a            1193.66        27.84929       YES     YES
   199        a            1195.33       147.85671       YES     YES
   200        a            1199.23       274.14833       YES     YES
   201        a            1201.27       545.13350       YES     YES
   202        a            1205.09       353.45971       YES     YES
   203        a            1214.58       667.36125       YES     YES
   204        a            1220.01       174.33870       YES     YES
   205        a            1222.70      1033.08530       YES     YES
   206        a            1224.39        41.77433       YES     YES
   207        a            1231.04        11.18212       YES     YES
   208        a            1231.38      1074.61247       YES     YES
   209        a            1245.28       883.51069       YES     YES
   210        a            1247.92         9.74917       YES     YES
   211        a            1249.96        87.97469       YES     YES
   212        a            1251.03       264.75006       YES     YES
   213        a            1254.72       209.29457       YES     YES
   214        a            1256.84       271.95794       YES     YES
   215        a            1261.63        43.78934       YES     YES
   216        a            1266.27       143.57807       YES     YES
   217        a            1284.52        77.90786       YES     YES
   218        a            1288.54        79.39199       YES     YES
   219        a            1291.26       129.66389       YES     YES
   220        a            1302.13       121.83750       YES     YES
   221        a            1307.64        59.56540       YES     YES
   222        a            1308.07       116.47050       YES     YES
   223        a            1312.94       180.72462       YES     YES
   224        a            1335.68         8.51561       YES     YES
   225        a            1378.79        56.87238       YES     YES
   226        a            1379.68        34.55760       YES     YES
   227        a            1382.91        96.53466       YES     YES
   228        a            1388.33        53.86114       YES     YES
   229        a            1389.34        10.14808       YES     YES
   230        a            1392.34         7.97284       YES     YES
   231        a            1395.08        25.34881       YES     YES
   232        a            1397.30        29.11661       YES     YES
   233        a            1400.35         8.11898       YES     YES
   234        a            1402.63        10.38092       YES     YES
   235        a            1403.36        14.85601       YES     YES
   236        a            1403.45        15.26373       YES     YES
   237        a            1414.87         6.63630       YES     YES
   238        a            1450.19         6.35384       YES     YES
   239        a            1490.31         0.55660       YES     YES
   240        a            1500.82        10.12838       YES     YES
   241        a            1503.16        10.54810       YES     YES
   242        a            1520.48         9.70943       YES     YES
   243        a            1525.63         4.92930       YES     YES
   244        a            1677.69        28.72403       YES     YES
   245        a            3003.93        23.74426       YES     YES
   246        a            3012.58        25.99979       YES     YES
   247        a            3017.19        16.01052       YES     YES
   248        a            3024.96        15.55223       YES     YES
   249        a            3028.41        13.18250       YES     YES
   250        a            3030.69        11.29965       YES     YES
   251        a            3041.45        32.85735       YES     YES
   252        a            3052.73        11.49754       YES     YES
   253        a            3058.60        15.28642       YES     YES
   254        a            3073.49        25.34275       YES     YES
   255        a            3103.53        17.48126       YES     YES
   256        a            3122.69         5.43064       YES     YES
   257        a            3231.48         0.59980       YES     YES
   258        a            3441.97       138.16637       YES     YES
   259        a            3524.47        52.63191       YES     YES
   260        a            3862.94       147.20780       YES     YES
   261        a            3907.85       239.92031       YES     YES
$end

Total COSMO energy + OC correction = -5822.5735146300 H
```

**[MOEA(H_2_O)]**

```
Method: (RI-)B3LYP(D3BJ)/def2-TZVPP
Symmetry: c1

Cartesian coordinates in Ångström:
C    -2.1793264    0.6686869   -0.1539338
O    -0.7988469    0.8812605   -0.3737814
H    -2.7163581    1.4787683   -0.6436386
H    -2.4162385    0.6720471    0.9161235
H    -2.5028036   -0.2859403   -0.5822344
C     0.0268828   -0.1176940    0.2142139
H    -0.1514063   -0.1471805    1.2983374
C     1.4937313    0.1753638   -0.0725498
H    -0.2474776   -1.0979038   -0.1985772
H     1.6240253    0.2634485   -1.1533822
H     2.0631245   -0.7053914    0.2390401
N     2.0702638    1.3545200    0.5526405
H     1.6602193    2.2075215    0.1890004
O     0.1258623    3.5106127   -0.7914527
H    -0.2734473    2.6317238   -0.6782606
H    -0.2785479    4.0641252   -0.1192554
H     1.9395432    1.3427516    1.5563804

SCF energy GEOOPT =  -326.1039230736 H
ZPE =   394.8 kJ/mol
FREEH energy =   420.53 kJ/mol
FREEH entropy =   0.40259 kJ/mol/K

$vibrational spectrum
#  mode     symmetry     wave number   IR intensity    selection rules
#                         cm**(-1)        km/mol         IR     RAMAN
     1                       -0.00         0.00000        -       -
     2                       -0.00         0.00000        -       -
     3                       -0.00         0.00000        -       -
     4                        0.00         0.00000        -       -
     5                        0.00         0.00000        -       -
     6                        0.00         0.00000        -       -
     7        a              37.42         4.79008       YES     YES
     8        a              87.05         4.81931       YES     YES
     9        a              96.04         9.06235       YES     YES
    10        a             157.32         4.70168       YES     YES
    11        a             167.98         2.22874       YES     YES
    12        a             188.80        82.86364       YES     YES
    13        a             226.83         2.62201       YES     YES
    14        a             278.80         5.78882       YES     YES
    15        a             328.62        55.54128       YES     YES
    16        a             358.18        48.89530       YES     YES
    17        a             370.32        19.37251       YES     YES
    18        a             525.48         4.80088       YES     YES
    19        a             664.81       142.33766       YES     YES
    20        a             812.79        32.76563       YES     YES
    21        a             855.24       136.71131       YES     YES
    22        a             906.40        23.33902       YES     YES
    23        a             998.93        16.45336       YES     YES
    24        a            1053.74        59.12413       YES     YES
    25        a            1127.33        36.53249       YES     YES
    26        a            1141.42       105.90852       YES     YES
    27        a            1178.05         3.36144       YES     YES
    28        a            1197.20         8.61600       YES     YES
    29        a            1241.40        16.38154       YES     YES
    30        a            1293.63         6.13083       YES     YES
    31        a            1388.75         7.00900       YES     YES
    32        a            1397.99         6.16571       YES     YES
    33        a            1412.98         8.09739       YES     YES
    34        a            1475.83         1.73863       YES     YES
    35        a            1479.46         1.85714       YES     YES
    36        a            1487.91         7.35041       YES     YES
    37        a            1500.77        10.51234       YES     YES
    38        a            1516.57         2.58500       YES     YES
    39        a            1648.17        39.31056       YES     YES
    40        a            1680.89        20.13372       YES     YES
    41        a            2956.41        43.20519       YES     YES
    42        a            2985.96        68.29714       YES     YES
    43        a            2989.19        50.00020       YES     YES
    44        a            3022.30        63.55738       YES     YES
    45        a            3033.40        43.79171       YES     YES
    46        a            3066.50        27.49221       YES     YES
    47        a            3114.53        21.16366       YES     YES
    48        a            3481.93        31.57046       YES     YES
    49        a            3566.52        17.32682       YES     YES
    50        a            3627.47       447.10878       YES     YES
    51        a            3874.91        72.64294       YES     YES
$end

Total COSMO energy + OC correction = -326.1171417577 H
```

**[(H_2_O)∙∙∙H-MOEA]^+^**

```
Method: (RI-)B3LYP(D3BJ)/def2-TZVPP
Symmetry: c1

Cartesian coordinates in Ångström:
C    -2.2186802    0.5623956    0.0309057
O    -0.8377867    0.8868561   -0.1629422
H    -2.7949442    1.4423996   -0.2401924
H    -2.4123931    0.3086483    1.0759783
H    -2.5093480   -0.2738429   -0.6085845
C     0.0256443   -0.1780304    0.1663224
H    -0.0906883   -0.4538007    1.2240728
C     1.4532829    0.2296154   -0.1230711
H    -0.1969731   -1.0705258   -0.4290551
H     1.5855945    0.4890550   -1.1710968
H     2.1364244   -0.5750352    0.1395194
N     1.8380176    1.4553197    0.6684597
H     1.3306704    2.2981156    0.2795611
H     2.8392195    1.6348235    0.6010523
O     0.5015947    3.3083973   -0.6933843
H    -0.3421976    2.8606572   -0.8353504
H     0.3660721    4.2565102   -0.7782331
H     1.6049307    1.3499613    1.6557483

SCF energy GEOOPT =  -326.4901206580 H
ZPE =   433.3 kJ/mol
FREEH energy =   458.72 kJ/mol
FREEH entropy =   0.39675 kJ/mol/K

$vibrational spectrum
#  mode     symmetry     wave number   IR intensity    selection rules
#                         cm**(-1)        km/mol         IR     RAMAN
     1                       -0.00         0.00000        -       -
     2                       -0.00         0.00000        -       -
     3                       -0.00         0.00000        -       -
     4                       -0.00         0.00000        -       -
     5                       -0.00         0.00000        -       -
     6                        0.00         0.00000        -       -
     7        a              50.43         1.68890       YES     YES
     8        a              93.61        13.41646       YES     YES
     9        a             101.48        12.90488       YES     YES
    10        a             132.65         6.22017       YES     YES
    11        a             197.97       145.41460       YES     YES
    12        a             210.02         4.13008       YES     YES
    13        a             277.21         1.34482       YES     YES
    14        a             320.71        23.04354       YES     YES
    15        a             333.74        86.77851       YES     YES
    16        a             354.46         9.07419       YES     YES
    17        a             384.72       109.37720       YES     YES
    18        a             470.83        60.77514       YES     YES
    19        a             530.44        14.39602       YES     YES
    20        a             824.21         7.01468       YES     YES
    21        a             862.35         2.24931       YES     YES
    22        a             976.75         2.12728       YES     YES
    23        a             988.31        37.84978       YES     YES
    24        a            1030.93        42.78243       YES     YES
    25        a            1127.49       174.64431       YES     YES
    26        a            1146.64         1.37353       YES     YES
    27        a            1168.92         4.44860       YES     YES
    28        a            1193.48         3.39669       YES     YES
    29        a            1235.67        13.82965       YES     YES
    30        a            1294.96         4.83154       YES     YES
    31        a            1351.59        11.11133       YES     YES
    32        a            1407.72        10.27377       YES     YES
    33        a            1440.96         4.52704       YES     YES
    34        a            1487.05         2.11408       YES     YES
    35        a            1490.21        12.36430       YES     YES
    36        a            1494.79        14.26576       YES     YES
    37        a            1503.05        10.58483       YES     YES
    38        a            1519.95         5.32502       YES     YES
    39        a            1573.02       103.86712       YES     YES
    40        a            1588.24        75.19031       YES     YES
    41        a            1662.64         3.32434       YES     YES
    42        a            1697.73        51.21741       YES     YES
    43        a            2831.45      1102.23715       YES     YES
    44        a            2971.17        38.37175       YES     YES
    45        a            3018.30        28.75472       YES     YES
    46        a            3026.39        15.14351       YES     YES
    47        a            3081.90        20.77840       YES     YES
    48        a            3092.46         0.78510       YES     YES
    49        a            3145.22         9.80824       YES     YES
    50        a            3147.88         0.21167       YES     YES
    51        a            3449.10        60.08468       YES     YES
    52        a            3507.66        72.98721       YES     YES
    53        a            3760.29        71.14123       YES     YES
    54        a            3882.01       207.29656       YES     YES
$end

Total COSMO energy + OC correction = -326.5661702379 H
```

**[Mg(MOEA)_2_(TfO)_2_]**

```
Method: (RI-)B3LYP(D3BJ)/def2-TZVPP
Symmetry: c1

Cartesian coordinates in Ångström:
C    -5.5991215   -0.3784371   -0.2211645
O    -6.3970722    0.5038872    0.5695006
H    -4.6358709    0.0869880   -0.4377426
H    -6.1131815   -0.6331468   -1.1482376
H    -5.4403306   -1.2820359    0.3597935
C    -6.6626671    1.7493461   -0.0863564
H    -5.7202412    2.2752126   -0.2589786
C    -7.5739448    2.5660766    0.8062857
H    -7.1348864    1.5451122   -1.0532673
H    -7.0788543    2.7504165    1.7580204
N    -8.8095675    1.8112410    1.0873624
H    -7.7659866    3.5316854    0.3321897
Mg   -8.1569235   -0.2055401    1.5681656
H    -9.4498597    1.8510720    0.3032980
H    -9.2689843    2.1839092    1.9164963
H   -11.6167714   -1.0821290    1.2916586
C   -11.2425091   -0.5608050    2.1730679
H   -11.8784331   -0.7547354    3.0377772
H   -10.1198669   -2.9807118    2.0075206
H   -11.2106327    0.5102485    1.9975432
H    -7.4925250   -2.8090198    1.3704313
C    -9.8036655   -2.3536360    2.8456408
O    -9.9062863   -0.9774038    2.4605187
N    -7.4498537   -2.1752715    2.1676779
H   -10.4623501   -2.5361862    3.6973228
C    -8.3661380   -2.6186047    3.2442744
H    -8.2542456   -3.6814481    3.4701631
H    -6.4999084   -2.1716996    2.5187717
H    -8.1342444   -2.0454025    4.1394676
F    -6.7340797    3.1223751    4.4528923
F    -6.1326049    1.5063309    5.7727930
C    -7.1852062    2.1866960    5.3076805
F    -7.7809649    2.8039328    6.3287799
S    -8.3786324    1.0529347    4.4475579
O    -7.5155757    0.5141581    3.3662130
O    -9.4135894    1.9365317    3.9433245
O    -8.7582422    0.0749308    5.4308194
F    -9.8427313   -0.8382469   -3.0414485
F    -9.3453236   -2.9321974   -3.3258228
C    -9.0338832   -1.8287827   -2.6480935
F    -7.7717813   -1.4821729   -2.9563601
S    -9.1983512   -2.1193196   -0.8212739
O   -10.5861014   -2.4341323   -0.6049536
O    -8.7994187   -0.7939274   -0.2887451
O    -8.2283221   -3.1537728   -0.5207940

SCF energy GEOOPT =  -2622.677886838 H
ZPE =   838.2 kJ/mol
FREEH energy =   921.38 kJ/mol
FREEH entropy =   0.86673 kJ/mol/K

$vibrational spectrum
#  mode     symmetry     wave number   IR intensity    selection rules
#                         cm**(-1)        km/mol         IR     RAMAN
     1                       -0.00         0.00000        -       -
     2                       -0.00         0.00000        -       -
     3                       -0.00         0.00000        -       -
     4                        0.00         0.00000        -       -
     5                        0.00         0.00000        -       -
     6                        0.00         0.00000        -       -
     7        a              13.93         0.40660       YES     YES
     8        a              25.25         0.57673       YES     YES
     9        a              25.60         1.87812       YES     YES
    10        a              40.10         1.20052       YES     YES
    11        a              44.54         0.84812       YES     YES
    12        a              47.06         0.84830       YES     YES
    13        a              57.38         1.66395       YES     YES
    14        a              59.21         2.65054       YES     YES
    15        a              77.06         1.78173       YES     YES
    16        a              84.80         1.93503       YES     YES
    17        a              91.25         0.38747       YES     YES
    18        a              93.63         6.36224       YES     YES
    19        a              98.49         2.58292       YES     YES
    20        a             111.35         7.30694       YES     YES
    21        a             120.81         1.98145       YES     YES
    22        a             137.41         1.79937       YES     YES
    23        a             145.20         4.57458       YES     YES
    24        a             150.81         0.97773       YES     YES
    25        a             157.45         2.03464       YES     YES
    26        a             166.92         3.82975       YES     YES
    27        a             172.09        12.72573       YES     YES
    28        a             178.81         3.22137       YES     YES
    29        a             202.96         0.48305       YES     YES
    30        a             206.45         1.05894       YES     YES
    31        a             214.68         4.34707       YES     YES
    32        a             225.40         4.95054       YES     YES
    33        a             241.96        10.58468       YES     YES
    34        a             246.31        14.13653       YES     YES
    35        a             268.16        11.22168       YES     YES
    36        a             290.26        10.19160       YES     YES
    37        a             294.29        14.06885       YES     YES
    38        a             300.52         3.37790       YES     YES
    39        a             313.02         0.48151       YES     YES
    40        a             320.37         4.41847       YES     YES
    41        a             322.82         9.63703       YES     YES
    42        a             333.96        30.79319       YES     YES
    43        a             346.50         5.95963       YES     YES
    44        a             346.82         3.63484       YES     YES
    45        a             378.09         3.41957       YES     YES
    46        a             380.68        20.84505       YES     YES
    47        a             408.41         3.12585       YES     YES
    48        a             423.86        55.98861       YES     YES
    49        a             455.43        33.92548       YES     YES
    50        a             506.63        11.25424       YES     YES
    51        a             513.41        39.79551       YES     YES
    52        a             513.97        10.08597       YES     YES
    53        a             518.07        23.50662       YES     YES
    54        a             528.08         9.34895       YES     YES
    55        a             533.92        56.54494       YES     YES
    56        a             568.10         8.64146       YES     YES
    57        a             569.35        17.40950       YES     YES
    58        a             577.46         2.88133       YES     YES
    59        a             583.55         8.98331       YES     YES
    60        a             618.44       102.01758       YES     YES
    61        a             630.98       341.23439       YES     YES
    62        a             653.52        93.54186       YES     YES
    63        a             657.21        14.34962       YES     YES
    64        a             756.94         3.51247       YES     YES
    65        a             758.50         5.30001       YES     YES
    66        a             835.56         7.02652       YES     YES
    67        a             847.77         7.47371       YES     YES
    68        a             903.47        19.64993       YES     YES
    69        a             905.90        25.84870       YES     YES
    70        a             983.35       153.23972       YES     YES
    71        a             998.45        87.82800       YES     YES
    72        a            1016.82       487.23338       YES     YES
    73        a            1026.28       116.46062       YES     YES
    74        a            1034.70        15.40899       YES     YES
    75        a            1047.71        12.42584       YES     YES
    76        a            1068.54        17.46864       YES     YES
    77        a            1071.97        29.65025       YES     YES
    78        a            1095.09        82.65097       YES     YES
    79        a            1105.21       180.94426       YES     YES
    80        a            1112.49        37.73087       YES     YES
    81        a            1117.23        50.29109       YES     YES
    82        a            1154.67       232.64423       YES     YES
    83        a            1155.07       177.95185       YES     YES
    84        a            1175.46        35.52868       YES     YES
    85        a            1179.54        32.25953       YES     YES
    86        a            1180.78        10.42946       YES     YES
    87        a            1186.83         2.37692       YES     YES
    88        a            1198.75       248.55951       YES     YES
    89        a            1199.37       250.32693       YES     YES
    90        a            1219.33         7.72462       YES     YES
    91        a            1229.68       328.86704       YES     YES
    92        a            1230.52        30.57333       YES     YES
    93        a            1233.96       126.67451       YES     YES
    94        a            1236.18        19.38549       YES     YES
    95        a            1240.35        90.21274       YES     YES
    96        a            1309.26        15.20829       YES     YES
    97        a            1313.86        43.18497       YES     YES
    98        a            1326.59       283.01941       YES     YES
    99        a            1329.80       289.36967       YES     YES
   100        a            1344.56         7.41867       YES     YES
   101        a            1346.43        15.83019       YES     YES
   102        a            1411.46         5.45383       YES     YES
   103        a            1415.08        11.75663       YES     YES
   104        a            1442.14         2.50143       YES     YES
   105        a            1449.26         2.87677       YES     YES
   106        a            1485.30         0.30862       YES     YES
   107        a            1486.27         0.35622       YES     YES
   108        a            1490.81        12.87917       YES     YES
   109        a            1492.91         8.86612       YES     YES
   110        a            1501.08         7.06638       YES     YES
   111        a            1502.59         4.08958       YES     YES
   112        a            1506.28         6.36525       YES     YES
   113        a            1512.24         2.86399       YES     YES
   114        a            1517.43         7.52251       YES     YES
   115        a            1526.25         8.36671       YES     YES
   116        a            1657.14        39.96490       YES     YES
   117        a            1659.31        13.97934       YES     YES
   118        a            3008.11        20.09612       YES     YES
   119        a            3026.03         8.41463       YES     YES
   120        a            3033.16        38.55067       YES     YES
   121        a            3035.83        34.81609       YES     YES
   122        a            3037.22        38.62731       YES     YES
   123        a            3041.67        45.96128       YES     YES
   124        a            3057.40        38.18861       YES     YES
   125        a            3072.67        17.26784       YES     YES
   126        a            3103.57        14.09518       YES     YES
   127        a            3106.60         9.08621       YES     YES
   128        a            3114.06         3.37700       YES     YES
   129        a            3119.77         0.80933       YES     YES
   130        a            3148.97        13.26188       YES     YES
   131        a            3150.77        13.08519       YES     YES
   132        a            3425.18       101.87459       YES     YES
   133        a            3431.87       133.90926       YES     YES
   134        a            3551.13        36.18043       YES     YES
   135        a            3552.30        34.71341       YES     YES
$end

Total COSMO energy + OC correction = -2622.7026948607 H
```

**[Mg(MOEA)_2_(Ohfip)_2_]**

```
Method: (RI-)B3LYP(D3BJ)/def2-TZVPP
Symmetry: c1

Cartesian coordinates in Ångström:
F    -1.4132835   -1.6100315   -2.4979913
F     0.3653533   -1.4143288   -0.3030244
C    -2.2280329   -2.1766066   -1.5881464
C    -0.6467173   -1.9387182    0.4117560
F    -2.0521966   -3.5069598   -1.6589178
F    -0.4214692   -3.2591728    0.5271617
O    -2.2476405   -0.2790209   -0.1549251
C    -2.0417180   -1.5937824   -0.1663233
F    -3.4992415   -1.9284778   -2.0001232
F    -0.5503621   -1.4153871    1.6616054
H    -2.7395071   -2.1920594    0.4562339
Mg   -2.9530378    1.5293029   -0.0202746
F    -2.9247816    4.9899274    2.1899247
F    -3.6595170    6.1447907    0.5062494
C    -4.0295167    5.2694076    1.4507170
F    -4.8894997    5.9009993    2.2775612
O    -3.6693620    3.3273586    0.1323436
C    -4.5912854    3.9489716    0.8770963
H    -4.9375734    3.3897738    1.7689000
C    -5.8873353    4.1801720    0.0658504
F    -5.6855492    4.8529401   -1.0771441
F    -6.8585176    4.8137362    0.7473454
F    -6.4019255    2.9641960   -0.2878915
H    -5.8440527   -0.3021846    2.2904858
H    -4.0060818    0.4243410    3.6750980
H    -1.7891996    1.4521750    3.8260778
H    -1.1743713    2.7534575    1.7146006
C    -3.3998502    0.5331310    2.7707620
C    -2.4023781    1.6600717    2.9446833
C    -5.2356829   -0.1227141    1.4008145
N    -1.5992071    1.8345343    1.7211427
O    -4.2460360    0.8612419    1.6684323
H    -5.8695025    0.2685403    0.6094995
H    -0.8706847    1.1319130    1.6679917
H    -2.9381373    2.5901907    3.1235762
H    -2.8899670   -0.4131477    2.5690550
H    -4.7749293   -1.0580072    1.0749416
H    -1.9526235    0.8574298   -3.1746018
H    -4.0930634    2.0025006   -3.8035896
H    -4.2685879    0.2828122   -2.1174437
H    -0.0570348    0.7641926   -1.6844878
C    -3.6585701    2.1366793   -2.8087384
C    -2.1671668    1.8931385   -2.8912638
N    -4.2547275    1.2443066   -1.7970090
O    -1.5973777    2.1537917   -1.6074972
C    -0.2119506    1.8344920   -1.5461540
H    -1.7139511    2.5595123   -3.6309768
H    -3.8388718    3.1594037   -2.4822738
H    -5.2041947    1.5364359   -1.6023270
H     0.1441683    2.1268944   -0.5617731
H     0.3363088    2.3987471   -2.3044181

SCF energy GEOOPT =  -2278.148782472 H
ZPE =   946.0 kJ/mol
FREEH energy =  1039.85 kJ/mol
FREEH entropy =   0.96871 kJ/mol/K

$vibrational spectrum
#  mode     symmetry     wave number   IR intensity    selection rules
#                         cm**(-1)        km/mol         IR     RAMAN
     1                       -0.00         0.00000        -       -
     2                        0.00         0.00000        -       -
     3                        0.00         0.00000        -       -
     4                        0.00         0.00000        -       -
     5                        0.00         0.00000        -       -
     6                        0.00         0.00000        -       -
     7        a               6.28         0.08916       YES     YES
     8        a              17.72         0.88192       YES     YES
     9        a              22.75         0.05584       YES     YES
    10        a              26.39         0.01463       YES     YES
    11        a              33.89         0.12374       YES     YES
    12        a              37.90         1.98952       YES     YES
    13        a              41.77         0.92429       YES     YES
    14        a              42.74         0.03143       YES     YES
    15        a              55.22         2.97891       YES     YES
    16        a              62.61         2.23073       YES     YES
    17        a              68.16         0.53338       YES     YES
    18        a              73.53         1.39773       YES     YES
    19        a              78.23         0.64361       YES     YES
    20        a              84.28         5.19052       YES     YES
    21        a              93.44         1.05448       YES     YES
    22        a             109.63        10.96241       YES     YES
    23        a             115.55         6.31933       YES     YES
    24        a             117.81         5.23698       YES     YES
    25        a             133.27         4.29657       YES     YES
    26        a             147.50         3.59591       YES     YES
    27        a             149.80         4.60621       YES     YES
    28        a             159.16         0.75182       YES     YES
    29        a             166.94         1.19859       YES     YES
    30        a             168.95         0.37266       YES     YES
    31        a             173.30         3.12849       YES     YES
    32        a             185.24         1.32303       YES     YES
    33        a             208.51         1.62048       YES     YES
    34        a             227.55         4.96936       YES     YES
    35        a             244.18         5.70649       YES     YES
    36        a             256.59         1.00219       YES     YES
    37        a             261.95         2.34607       YES     YES
    38        a             270.76         6.22153       YES     YES
    39        a             273.95        11.47519       YES     YES
    40        a             286.47         7.24365       YES     YES
    41        a             290.49         0.17939       YES     YES
    42        a             292.53         0.36953       YES     YES
    43        a             299.30         4.29770       YES     YES
    44        a             303.04         1.52126       YES     YES
    45        a             310.79        54.23818       YES     YES
    46        a             320.87         7.04212       YES     YES
    47        a             323.01        11.66545       YES     YES
    48        a             344.57         6.11215       YES     YES
    49        a             348.58         2.77740       YES     YES
    50        a             361.66        27.54575       YES     YES
    51        a             374.27         8.73462       YES     YES
    52        a             393.11        31.49321       YES     YES
    53        a             411.56        42.51609       YES     YES
    54        a             490.81        20.34420       YES     YES
    55        a             496.21         2.49097       YES     YES
    56        a             501.78        23.56530       YES     YES
    57        a             510.23         6.52272       YES     YES
    58        a             511.51         8.77388       YES     YES
    59        a             524.23         2.47911       YES     YES
    60        a             525.22         2.31231       YES     YES
    61        a             533.53        41.00326       YES     YES
    62        a             540.25         0.39475       YES     YES
    63        a             540.67         0.70946       YES     YES
    64        a             583.62        39.04067       YES     YES
    65        a             599.28        98.14330       YES     YES
    66        a             635.98         2.40433       YES     YES
    67        a             661.39        20.73654       YES     YES
    68        a             678.91        31.07894       YES     YES
    69        a             680.68        37.89753       YES     YES
    70        a             735.16        11.42736       YES     YES
    71        a             738.05        43.91854       YES     YES
    72        a             833.85        15.99897       YES     YES
    73        a             839.95        36.23556       YES     YES
    74        a             841.56         9.45325       YES     YES
    75        a             849.84        61.54267       YES     YES
    76        a             869.00        38.90329       YES     YES
    77        a             875.98        62.16715       YES     YES
    78        a             900.10        51.66168       YES     YES
    79        a             901.64        27.21850       YES     YES
    80        a             961.98       195.70589       YES     YES
    81        a             971.22        71.60560       YES     YES
    82        a            1027.76        19.55872       YES     YES
    83        a            1030.27         6.91909       YES     YES
    84        a            1048.23        82.66369       YES     YES
    85        a            1054.33       182.73790       YES     YES
    86        a            1063.36         9.93622       YES     YES
    87        a            1075.38        13.79176       YES     YES
    88        a            1083.77        95.42158       YES     YES
    89        a            1085.14        18.82659       YES     YES
    90        a            1120.67        59.82995       YES     YES
    91        a            1121.94        17.63335       YES     YES
    92        a            1126.54       108.73102       YES     YES
    93        a            1132.60        30.72505       YES     YES
    94        a            1149.59       273.90558       YES     YES
    95        a            1156.18       187.97273       YES     YES
    96        a            1176.47         5.52063       YES     YES
    97        a            1177.39       153.45658       YES     YES
    98        a            1179.40       271.78848       YES     YES
    99        a            1181.76       437.88193       YES     YES
   100        a            1216.94         8.06138       YES     YES
   101        a            1219.91         7.26557       YES     YES
   102        a            1233.29        19.06478       YES     YES
   103        a            1235.85        19.26315       YES     YES
   104        a            1241.38       127.13055       YES     YES
   105        a            1243.33       569.71441       YES     YES
   106        a            1252.50       143.20667       YES     YES
   107        a            1256.95       172.49120       YES     YES
   108        a            1283.27       148.12150       YES     YES
   109        a            1297.53       238.69771       YES     YES
   110        a            1310.07         7.29277       YES     YES
   111        a            1313.69         9.35922       YES     YES
   112        a            1336.98         4.65523       YES     YES
   113        a            1345.45         7.01473       YES     YES
   114        a            1364.85        23.22359       YES     YES
   115        a            1370.57        12.08360       YES     YES
   116        a            1380.62        14.12171       YES     YES
   117        a            1385.35        15.34676       YES     YES
   118        a            1410.43         5.10964       YES     YES
   119        a            1413.86        11.25944       YES     YES
   120        a            1440.80         4.22169       YES     YES
   121        a            1448.89         2.58166       YES     YES
   122        a            1479.26         0.01830       YES     YES
   123        a            1483.30         0.20859       YES     YES
   124        a            1492.47        11.22153       YES     YES
   125        a            1493.99         8.62622       YES     YES
   126        a            1494.92         4.30762       YES     YES
   127        a            1497.22         7.62496       YES     YES
   128        a            1500.51         1.39970       YES     YES
   129        a            1501.35         6.92095       YES     YES
   130        a            1512.59         8.94287       YES     YES
   131        a            1518.02         4.99947       YES     YES
   132        a            1645.15        33.80788       YES     YES
   133        a            1652.40        23.73132       YES     YES
   134        a            2850.42        87.84459       YES     YES
   135        a            2878.49        70.76217       YES     YES
   136        a            3007.98         8.55706       YES     YES
   137        a            3011.27         7.28422       YES     YES
   138        a            3018.22        49.13873       YES     YES
   139        a            3022.21        50.76384       YES     YES
   140        a            3025.26        80.32835       YES     YES
   141        a            3028.58        58.79379       YES     YES
   142        a            3049.46        31.92585       YES     YES
   143        a            3056.34        26.42849       YES     YES
   144        a            3076.23        28.50385       YES     YES
   145        a            3095.91        16.75174       YES     YES
   146        a            3112.10         2.45974       YES     YES
   147        a            3114.03         8.63253       YES     YES
   148        a            3136.12        19.08362       YES     YES
   149        a            3137.78        13.82330       YES     YES
   150        a            3496.98         4.94559       YES     YES
   151        a            3500.65         6.97269       YES     YES
   152        a            3581.75        17.60303       YES     YES
   153        a            3582.49        41.40971       YES     YES
$end

Total COSMO energy + OC correction = -2278.1637881143 H
```

**[Mg(TfO)_2_]**

```
Method: (RI-)B3LYP(D3BJ)/def2-TZVPP
Symmetry: c2

Cartesian coordinates in Ångström:
O     1.4782719   -1.1015693    1.4544686
F     2.6501751   -1.1592010   -1.2378028
O    -0.0174756   -1.8291938   -0.2080205
F    -3.6878356    2.6139786   -0.0010670
S     1.1534243   -2.2848508    0.6011077
C     2.5501523   -2.3464743   -0.6290212
Mg    0.0000000    0.0000000    0.6392655
F     3.6878356   -2.6139786   -0.0010670
F     2.3061265   -3.2841658   -1.5356308
C    -2.5501523    2.3464743   -0.6290212
F    -2.3061265    3.2841658   -1.5356308
O     1.0802852   -3.5586542    1.2246507
F    -2.6501751    1.1592010   -1.2378028
O    -1.0802852    3.5586542    1.2246507
S    -1.1534243    2.2848508    0.6011077
O    -1.4782719    1.1015693    1.4544686
O     0.0174756    1.8291938   -0.2080205

SCF energy GEOOPT =  -2123.212660231 H
ZPE =   155.4 kJ/mol
FREEH energy =   199.99 kJ/mol
FREEH entropy =   0.58261 kJ/mol/K

$vibrational spectrum
#  mode     symmetry     wave number   IR intensity    selection rules
#                         cm**(-1)        km/mol         IR     RAMAN
     1                       -0.00         0.00000        -       -
     2                       -0.00         0.00000        -       -
     3                       -0.00         0.00000        -       -
     4                       -0.00         0.00000        -       -
     5                       -0.00         0.00000        -       -
     6                        0.00         0.00000        -       -
     7        a              18.91         0.01243       YES     YES
     8        a              25.50         1.37169       YES     YES
     9        b              27.05         1.66270       YES     YES
    10        a              47.96         0.02188       YES     YES
    11        b              48.44         0.10930       YES     YES
    12        a              98.28        10.30268       YES     YES
    13        b             102.21        10.70495       YES     YES
    14        a             166.06         0.38929       YES     YES
    15        b             180.87         0.28430       YES     YES
    16        a             184.38         0.05471       YES     YES
    17        b             217.89         8.78658       YES     YES
    18        a             221.09         0.37483       YES     YES
    19        b             301.20         0.54064       YES     YES
    20        a             304.94         0.04716       YES     YES
    21        b             313.18         6.45513       YES     YES
    22        a             314.93         6.56698       YES     YES
    23        b             328.36         1.10654       YES     YES
    24        a             328.93         0.98178       YES     YES
    25        b             338.48         9.79082       YES     YES
    26        a             373.79         0.96074       YES     YES
    27        b             511.72        11.05212       YES     YES
    28        a             512.00        14.23527       YES     YES
    29        b             516.81        63.78931       YES     YES
    30        a             546.77         1.18664       YES     YES
    31        b             565.76        84.45348       YES     YES
    32        a             571.31         4.02232       YES     YES
    33        b             571.41         8.39269       YES     YES
    34        a             608.45        71.34689       YES     YES
    35        b             631.15       141.12515       YES     YES
    36        b             662.18       309.58151       YES     YES
    37        a             672.04        47.51971       YES     YES
    38        b             766.76         0.79430       YES     YES
    39        a             766.79         0.64287       YES     YES
    40        b             981.00       383.60465       YES     YES
    41        a             990.42       115.32166       YES     YES
    42        a            1097.20       182.35209       YES     YES
    43        b            1097.23       186.93934       YES     YES
    44        a            1179.38       101.39049       YES     YES
    45        b            1179.78       269.39911       YES     YES
    46        b            1199.93       266.46319       YES     YES
    47        a            1200.90        19.23676       YES     YES
    48        b            1220.05       305.68575       YES     YES
    49        a            1220.39       297.97847       YES     YES
    50        b            1384.46       577.37722       YES     YES
    51        a            1391.85        45.13758       YES     YES
$end

Total COSMO energy + OC correction = -2123.2295960988 H
```

**[Mg(MOEA)(TfO)_2_]**

```
Method: (RI-)B3LYP(D3BJ)/def2-TZVPP
Symmetry: c1

Cartesian coordinates in Ångström:
Mg   -7.9905944   -0.1315580    1.5278496
H   -11.4375161   -0.1046880    1.3638400
C   -10.9597052    0.1454008    2.3120126
H   -11.6442774   -0.0272586    3.1433218
H   -10.4455762   -2.3859369    1.6027662
H   -10.6422585    1.1831805    2.3027020
H    -7.8493366   -2.7572787    0.8967717
C   -10.0077763   -2.0505065    2.5496537
O    -9.7781225   -0.6441070    2.4919008
N    -7.7058463   -2.2642935    1.7711305
H   -10.7063973   -2.2774712    3.3583292
C    -8.6656246   -2.7078633    2.8037449
H    -8.7862559   -3.7927946    2.8323179
H    -6.7524259   -2.4475830    2.0625323
H    -8.2850921   -2.3797669    3.7700707
F    -9.0128557    0.8725524    4.9273186
F    -8.4844252    2.9736505    5.0467957
C    -7.9933970    1.7447383    4.8945984
F    -7.1733336    1.4719922    5.9090909
S    -7.0716838    1.6215553    3.2821074
O    -8.1109749    1.8396865    2.2492425
O    -6.7072381    0.1882115    3.1841492
O    -6.0162025    2.5733452    3.3805068
F    -8.7487866   -1.5477084   -2.9469578
F    -7.7011130   -2.5102034   -1.3079943
C    -7.7168621   -1.4243509   -2.1160887
F    -6.5868442   -1.4139951   -2.8144681
S    -7.8785817    0.1340255   -1.1116205
O    -9.0937477   -0.0979248   -0.2960408
O    -7.9082256    1.1880449   -2.0694988
O    -6.7434230    0.0766353   -0.1664942

SCF energy GEOOPT =  -2372.953587972 H
ZPE =   496.0 kJ/mol
FREEH energy =   560.10 kJ/mol
FREEH entropy =   0.72554 kJ/mol/K

$vibrational spectrum
#  mode     symmetry     wave number   IR intensity    selection rules
#                         cm**(-1)        km/mol         IR     RAMAN
     1                       -0.00         0.00000        -       -
     2                       -0.00         0.00000        -       -
     3                       -0.00         0.00000        -       -
     4                       -0.00         0.00000        -       -
     5                       -0.00         0.00000        -       -
     6                        0.00         0.00000        -       -
     7        a              25.38         0.32462       YES     YES
     8        a              29.97         0.33658       YES     YES
     9        a              35.25         0.52717       YES     YES
    10        a              37.52         1.51539       YES     YES
    11        a              40.80         0.95270       YES     YES
    12        a              50.87         1.60814       YES     YES
    13        a              64.45         0.43881       YES     YES
    14        a              73.55         0.67707       YES     YES
    15        a              79.32         0.56249       YES     YES
    16        a             110.56         4.48688       YES     YES
    17        a             122.66         3.08465       YES     YES
    18        a             151.13         4.55616       YES     YES
    19        a             157.88         0.60775       YES     YES
    20        a             167.09         0.14252       YES     YES
    21        a             176.86         2.52582       YES     YES
    22        a             206.25         2.47196       YES     YES
    23        a             211.92         5.33491       YES     YES
    24        a             215.47         2.03513       YES     YES
    25        a             220.67         2.82665       YES     YES
    26        a             224.86         0.52930       YES     YES
    27        a             268.03         3.75863       YES     YES
    28        a             286.69         5.53710       YES     YES
    29        a             294.64         8.94179       YES     YES
    30        a             308.45         1.10052       YES     YES
    31        a             321.37        18.35165       YES     YES
    32        a             332.07         5.65552       YES     YES
    33        a             333.04         6.62005       YES     YES
    34        a             334.79        15.46120       YES     YES
    35        a             359.35         6.82401       YES     YES
    36        a             373.66         4.18680       YES     YES
    37        a             416.36        80.30593       YES     YES
    38        a             441.96        60.59484       YES     YES
    39        a             505.31        11.73432       YES     YES
    40        a             508.83        17.95047       YES     YES
    41        a             515.30        77.91610       YES     YES
    42        a             539.76        20.54983       YES     YES
    43        a             541.28        13.26774       YES     YES
    44        a             566.66         3.65565       YES     YES
    45        a             569.50         5.16244       YES     YES
    46        a             592.96        33.04655       YES     YES
    47        a             597.17        59.37583       YES     YES
    48        a             605.76        65.59538       YES     YES
    49        a             647.91       284.89106       YES     YES
    50        a             658.14        10.05914       YES     YES
    51        a             758.11         4.40013       YES     YES
    52        a             761.13         2.95549       YES     YES
    53        a             844.51        12.85108       YES     YES
    54        a             907.76        16.54776       YES     YES
    55        a             988.92       132.81241       YES     YES
    56        a            1004.29       405.33499       YES     YES
    57        a            1010.77        45.64484       YES     YES
    58        a            1021.48        13.49699       YES     YES
    59        a            1069.00        25.98342       YES     YES
    60        a            1087.51       127.10245       YES     YES
    61        a            1115.65        58.72112       YES     YES
    62        a            1136.14       147.83874       YES     YES
    63        a            1142.40       142.04026       YES     YES
    64        a            1156.89       118.10715       YES     YES
    65        a            1168.52       269.17768       YES     YES
    66        a            1181.80         0.50816       YES     YES
    67        a            1199.28       308.91339       YES     YES
    68        a            1201.61        96.48966       YES     YES
    69        a            1205.92       221.91573       YES     YES
    70        a            1212.29       264.75382       YES     YES
    71        a            1220.51         7.27594       YES     YES
    72        a            1233.79         6.10771       YES     YES
    73        a            1303.78         7.19835       YES     YES
    74        a            1336.89         6.82192       YES     YES
    75        a            1370.38       325.71226       YES     YES
    76        a            1378.00       388.68129       YES     YES
    77        a            1407.20         8.39734       YES     YES
    78        a            1436.59         2.01948       YES     YES
    79        a            1485.17         0.63192       YES     YES
    80        a            1496.69        10.01878       YES     YES
    81        a            1499.92        11.64489       YES     YES
    82        a            1502.78         4.20564       YES     YES
    83        a            1519.84         8.62767       YES     YES
    84        a            1654.51        38.71952       YES     YES
    85        a            3005.08        24.03874       YES     YES
    86        a            3033.25        33.44808       YES     YES
    87        a            3043.67        22.79443       YES     YES
    88        a            3061.78        34.52053       YES     YES
    89        a            3101.90        21.85572       YES     YES
    90        a            3105.84         8.20180       YES     YES
    91        a            3162.19         1.69986       YES     YES
    92        a            3500.98        22.94859       YES     YES
    93        a            3570.36        35.76710       YES     YES
$end

Total COSMO energy + OC correction = -2372.9755897416 H
```

**[Mg(MOEA)_3_(TfO)_2_]**

```
Method: (RI-)B3LYP(D3BJ)/def2-TZVPP
Symmetry: c1

Cartesian coordinates in Ångström:
C    -6.9246544    2.4040503   -0.1683104
O    -6.4355352    1.7119717    0.9817455
H    -6.1378056    3.0402081   -0.5793368
H    -7.7925421    3.0059858    0.1010581
H    -7.2008423    1.6642491   -0.9132534
C    -5.9905644    2.6471548    1.9830883
H    -5.1874519    3.2588303    1.5644281
C    -5.4952546    1.8611291    3.1734159
H    -6.8358638    3.2793490    2.2558292
H    -4.5797552    1.3271375    2.9143515
N    -6.5226363    0.8761877    3.5455624
H    -5.2661539    2.5606623    3.9814463
Mg   -7.1521861   -0.2263290    1.7813458
H    -7.3777966    1.3429830    3.8890969
H    -6.1946540    0.3118120    4.3195902
H   -10.1485682   -0.3317264   -0.3075134
C    -9.9684231    0.4394874    0.4454627
H   -10.9075637    0.8568875    0.8029634
H   -10.1412689   -1.9813613    1.5240887
H    -9.3704100    1.2475488    0.0411821
H    -7.7551745   -2.7241862    2.5785935
C   -10.0117122   -1.1446072    2.2146142
O    -9.2491296   -0.1201498    1.5476680
N    -7.8412546   -1.8386028    3.0862265
H   -10.9793981   -0.7274804    2.4929275
C    -9.2472479   -1.5520381    3.4511530
H    -9.7410124   -2.4135855    3.9054324
H    -7.2999350   -1.9648189    3.9327854
H    -9.2572114   -0.7265766    4.1618424
F    -9.1428392    4.7373089    4.5179033
F   -10.9514282    3.8518776    5.3301537
C   -10.2637672    4.0627497    4.2029720
F   -11.0001572    4.8380693    3.3976766
S    -9.8386159    2.4583467    3.3663981
O    -9.0296984    1.7720852    4.3796918
O    -9.0681991    2.8823162    2.2034640
O   -11.1152620    1.8415583    3.0890126
F    -6.2863387   -4.4824830   -1.3669460
F    -8.0272339   -5.6999960   -1.8198118
C    -7.2126961   -5.3165533   -0.8352778
F    -6.5705618   -6.3904475   -0.3682347
S    -8.1493710   -4.4477749    0.5164373
O    -8.7018964   -3.2780495   -0.1715464
O    -7.0819796   -4.0847900    1.4554828
O    -9.1079916   -5.4048939    0.9932505
H    -5.2335366   -0.1579903   -0.8288760
H    -7.5682422   -0.4066428   -0.9320910
C    -5.6721927   -1.1444623   -0.6738776
H    -5.5784968   -1.7128909   -1.6019109
N    -7.0727391   -0.9715381   -0.2534769
H    -7.5719090   -1.8734988   -0.2552043
C    -4.9206663   -1.8758072    0.4138028
H    -3.8474407   -1.8796027    0.2079217
O    -5.1596254   -1.1911189    1.6589902
C    -4.5709560   -1.8877901    2.7544361
H    -3.4900966   -1.9604569    2.6164621
H    -5.2791576   -2.9008318    0.5109158
H    -4.9965197   -2.8893029    2.8414050
H    -4.7717791   -1.3169622    3.6571233

SCF energy GEOOPT =  -2872.344076904 H
ZPE =   1180. kJ/mol
FREEH energy =  1282.06 kJ/mol
FREEH entropy =   1.00596 kJ/mol/K

$vibrational spectrum
#  mode     symmetry     wave number   IR intensity    selection rules
#                         cm**(-1)        km/mol         IR     RAMAN
     1                       -0.00         0.00000        -       -
     2                       -0.00         0.00000        -       -
     3                       -0.00         0.00000        -       -
     4                       -0.00         0.00000        -       -
     5                        0.00         0.00000        -       -
     6                        0.00         0.00000        -       -
     7        a              17.18         2.32145       YES     YES
     8        a              19.72         1.18492       YES     YES
     9        a              29.73         1.17135       YES     YES
    10        a              30.88         0.50219       YES     YES
    11        a              34.38         1.22111       YES     YES
    12        a              38.57         0.26295       YES     YES
    13        a              42.39         3.10300       YES     YES
    14        a              43.19         2.11062       YES     YES
    15        a              51.11         3.58528       YES     YES
    16        a              56.79         2.26761       YES     YES
    17        a              73.18         2.28327       YES     YES
    18        a              79.78         6.54095       YES     YES
    19        a              84.67         1.21156       YES     YES
    20        a              88.46         6.97937       YES     YES
    21        a             104.00         5.02474       YES     YES
    22        a             109.38         1.61040       YES     YES
    23        a             118.60         7.78194       YES     YES
    24        a             120.97         2.92009       YES     YES
    25        a             129.00         6.47726       YES     YES
    26        a             136.81         3.28546       YES     YES
    27        a             143.73         1.72653       YES     YES
    28        a             151.79         1.62172       YES     YES
    29        a             168.59         3.40553       YES     YES
    30        a             174.36         8.85226       YES     YES
    31        a             175.31         6.46986       YES     YES
    32        a             182.17         6.96264       YES     YES
    33        a             193.87         3.02167       YES     YES
    34        a             196.63         1.29810       YES     YES
    35        a             199.16         3.48333       YES     YES
    36        a             205.08         1.87823       YES     YES
    37        a             214.05         4.13998       YES     YES
    38        a             217.43         1.61410       YES     YES
    39        a             222.85         2.57616       YES     YES
    40        a             237.85         3.05116       YES     YES
    41        a             248.44         8.40866       YES     YES
    42        a             260.46         5.93642       YES     YES
    43        a             269.23        21.57495       YES     YES
    44        a             284.43        26.76608       YES     YES
    45        a             300.76         8.51747       YES     YES
    46        a             304.23         7.14287       YES     YES
    47        a             309.45        15.24177       YES     YES
    48        a             310.17        22.34901       YES     YES
    49        a             316.06         1.81617       YES     YES
    50        a             323.79        34.47677       YES     YES
    51        a             328.64         0.68003       YES     YES
    52        a             342.47         2.77869       YES     YES
    53        a             342.97         0.40270       YES     YES
    54        a             346.44        10.96854       YES     YES
    55        a             357.49         8.48653       YES     YES
    56        a             361.93         4.92912       YES     YES
    57        a             390.68        21.12650       YES     YES
    58        a             413.20         1.06684       YES     YES
    59        a             428.96        20.57941       YES     YES
    60        a             445.15        23.75150       YES     YES
    61        a             510.32        27.78154       YES     YES
    62        a             512.41        11.97721       YES     YES
    63        a             514.95        17.69039       YES     YES
    64        a             516.21        46.07818       YES     YES
    65        a             530.69        13.83288       YES     YES
    66        a             543.61         8.61337       YES     YES
    67        a             544.53        50.71785       YES     YES
    68        a             568.06         7.72052       YES     YES
    69        a             569.72         5.94925       YES     YES
    70        a             573.54         6.03429       YES     YES
    71        a             574.35        19.87660       YES     YES
    72        a             603.92        59.60926       YES     YES
    73        a             632.89       344.33330       YES     YES
    74        a             635.47        60.96762       YES     YES
    75        a             645.03        33.94653       YES     YES
    76        a             663.27        19.50570       YES     YES
    77        a             751.36         4.62710       YES     YES
    78        a             753.75         5.17555       YES     YES
    79        a             846.88        30.63561       YES     YES
    80        a             851.45         8.24634       YES     YES
    81        a             852.00         5.69360       YES     YES
    82        a             915.90        28.37490       YES     YES
    83        a             918.17        11.31601       YES     YES
    84        a             922.36        11.74709       YES     YES
    85        a             999.78        60.55847       YES     YES
    86        a            1001.39        94.89688       YES     YES
    87        a            1007.23         4.31487       YES     YES
    88        a            1018.41       341.03962       YES     YES
    89        a            1023.77       196.56728       YES     YES
    90        a            1057.28         5.37122       YES     YES
    91        a            1065.37        22.03962       YES     YES
    92        a            1068.79         5.40131       YES     YES
    93        a            1072.38        90.89596       YES     YES
    94        a            1079.57        38.45683       YES     YES
    95        a            1080.39        23.71579       YES     YES
    96        a            1109.14        69.10756       YES     YES
    97        a            1112.26       157.20642       YES     YES
    98        a            1114.12        31.90055       YES     YES
    99        a            1127.57       207.90049       YES     YES
   100        a            1134.16        49.56456       YES     YES
   101        a            1147.24        27.95112       YES     YES
   102        a            1150.80        82.14804       YES     YES
   103        a            1152.75       227.24119       YES     YES
   104        a            1168.09        85.08251       YES     YES
   105        a            1174.59        31.43332       YES     YES
   106        a            1182.07         1.08822       YES     YES
   107        a            1183.49         5.81756       YES     YES
   108        a            1183.72         0.16845       YES     YES
   109        a            1208.34       104.79697       YES     YES
   110        a            1210.34        78.99932       YES     YES
   111        a            1215.07       264.33587       YES     YES
   112        a            1222.08         8.13999       YES     YES
   113        a            1224.55       119.67570       YES     YES
   114        a            1229.29       353.13475       YES     YES
   115        a            1233.15        14.05653       YES     YES
   116        a            1240.09        16.37087       YES     YES
   117        a            1244.19         9.49542       YES     YES
   118        a            1245.71        34.03325       YES     YES
   119        a            1296.99       357.27437       YES     YES
   120        a            1317.95        21.25378       YES     YES
   121        a            1318.83         6.12218       YES     YES
   122        a            1324.73       245.23849       YES     YES
   123        a            1326.74       137.29718       YES     YES
   124        a            1348.27        64.06235       YES     YES
   125        a            1354.51         5.06941       YES     YES
   126        a            1360.74        10.60357       YES     YES
   127        a            1419.23         8.13566       YES     YES
   128        a            1420.31         3.31102       YES     YES
   129        a            1421.81         8.97814       YES     YES
   130        a            1449.39         5.61450       YES     YES
   131        a            1451.31         0.62347       YES     YES
   132        a            1454.00         2.50827       YES     YES
   133        a            1480.31         7.59741       YES     YES
   134        a            1484.39         2.95056       YES     YES
   135        a            1486.94         6.35546       YES     YES
   136        a            1487.30         1.60233       YES     YES
   137        a            1493.34         6.32830       YES     YES
   138        a            1494.80         2.83570       YES     YES
   139        a            1504.56         5.59350       YES     YES
   140        a            1508.60         5.39379       YES     YES
   141        a            1510.44         2.90272       YES     YES
   142        a            1511.18         2.86925       YES     YES
   143        a            1512.15         5.68854       YES     YES
   144        a            1516.19         5.80616       YES     YES
   145        a            1518.67         5.29726       YES     YES
   146        a            1521.57        14.60509       YES     YES
   147        a            1523.11         9.28034       YES     YES
   148        a            1682.56        27.30465       YES     YES
   149        a            1692.31        40.33287       YES     YES
   150        a            1694.12        39.70281       YES     YES
   151        a            3022.88        27.87241       YES     YES
   152        a            3025.13         1.98115       YES     YES
   153        a            3026.77        30.91793       YES     YES
   154        a            3028.33        47.26757       YES     YES
   155        a            3031.81        18.63917       YES     YES
   156        a            3035.41        93.05712       YES     YES
   157        a            3039.15         7.13201       YES     YES
   158        a            3040.80        75.49217       YES     YES
   159        a            3043.96        28.98700       YES     YES
   160        a            3069.81        33.39180       YES     YES
   161        a            3077.68        24.34618       YES     YES
   162        a            3086.37        18.17763       YES     YES
   163        a            3096.86         3.62359       YES     YES
   164        a            3098.81        13.64958       YES     YES
   165        a            3100.79         6.55838       YES     YES
   166        a            3103.60         5.50101       YES     YES
   167        a            3109.19         3.54794       YES     YES
   168        a            3116.98         5.83052       YES     YES
   169        a            3135.51        26.80911       YES     YES
   170        a            3149.43        26.78851       YES     YES
   171        a            3178.20         5.49787       YES     YES
   172        a            3195.44       637.08523       YES     YES
   173        a            3233.99       551.04947       YES     YES
   174        a            3342.97       473.17288       YES     YES
   175        a            3537.96        13.66626       YES     YES
   176        a            3540.63        11.71221       YES     YES
   177        a            3542.72        11.88367       YES     YES
$end

Total COSMO energy + OC correction = -2872.3810899659 H
```

**[TfO]^−^**

```
Method: (RI-)B3LYP(D3BJ)/def2-TZVPP
Symmetry: c3v

Cartesian coordinates in Ångström:
F    -0.6262086    1.0846250   -1.3566066
F    -0.6262086   -1.0846250   -1.3566066
C    -0.0000000    0.0000000   -0.8483611
F     1.2524171    0.0000000   -1.3566066
S     0.0000000    0.0000000    1.0217783
O    -1.4192266    0.0000000    1.3310388
O     0.7096133    1.2290863    1.3310388
O     0.7096133   -1.2290863    1.3310388

SCF energy GEOOPT =  -961.5921348483 H
ZPE =   71.63 kJ/mol
FREEH energy =    90.31 kJ/mol
FREEH entropy =   0.34711 kJ/mol/K

$vibrational spectrum
#  mode     symmetry     wave number   IR intensity    selection rules
#                         cm**(-1)        km/mol         IR     RAMAN
     1                       -0.00         0.00000        -       -
     2                       -0.00         0.00000        -       -
     3                       -0.00         0.00000        -       -
     4                       -0.00         0.00000        -       -
     5                        0.00         0.00000        -       -
     6                        0.00         0.00000        -       -
     7        a2             63.00         0.00000       NO      NO
     8        e             195.44         1.00415       YES     YES
     9        e             195.44         1.00415       YES     YES
    10        a1            288.39         0.05773       YES     YES
    11        e             338.02         0.03593       YES     YES
    12        e             338.02         0.03593       YES     YES
    13        e             511.24        10.99928       YES     YES
    14        e             511.24        10.99928       YES     YES
    15        e             564.89        12.07499       YES     YES
    16        e             564.89        12.07499       YES     YES
    17        a1            635.39       170.11608       YES     YES
    18        a1            735.20         8.95304       YES     YES
    19        a1           1031.41       173.80236       YES     YES
    20        e            1113.81       188.61759       YES     YES
    21        e            1113.81       188.61759       YES     YES
    22        a1           1199.73        64.65076       YES     YES
    23        e            1287.83       372.26413       YES     YES
    24        e            1287.83       372.26413       YES     YES
$end

Total COSMO energy + OC correction = -961.6674198200 H
```

**G2**

```
Method: (RI-)B3LYP(D3BJ)/def2-TZVPP
Symmetry: c2v

Cartesian coordinates in Ångström:
C     0.0000000   -4.7101375   -0.2783660
O     0.0000000   -3.5380682    0.5055604
H     0.8900173   -4.7639520   -0.9174622
H    -0.8900173   -4.7639520   -0.9174622
H     0.0000000   -5.5583196    0.4033726
C     0.0000000   -2.3589868   -0.2709400
C     0.0000000   -1.1791908    0.6788087
H    -0.8864977   -2.3169615   -0.9170251
H     0.8864977   -2.3169615   -0.9170251
O     0.0000000    0.0000000   -0.0987491
H     0.8863792   -1.2201458    1.3239831
H    -0.8863792   -1.2201458    1.3239831
C     0.0000000    1.1791908    0.6788087
C     0.0000000    2.3589868   -0.2709400
H     0.8863792    1.2201458    1.3239831
H    -0.8863792    1.2201458    1.3239831
H    -0.8864977    2.3169615   -0.9170251
H     0.8864977    2.3169615   -0.9170251
O     0.0000000    3.5380682    0.5055604
C     0.0000000    4.7101375   -0.2783660
H     0.0000000    5.5583196    0.4033726
H    -0.8900173    4.7639520   -0.9174622
H     0.8900173    4.7639520   -0.9174622

SCF energy GEOOPT =  -462.6415740778 H
ZPE =   530.5 kJ/mol
FREEH energy =   561.72 kJ/mol
FREEH entropy =   0.45354 kJ/mol/K

$vibrational spectrum
#  mode     symmetry     wave number   IR intensity    selection rules
#                         cm**(-1)        km/mol         IR     RAMAN
     1                       -0.00         0.00000        -       -
     2                       -0.00         0.00000        -       -
     3                        0.00         0.00000        -       -
     4                        0.00         0.00000        -       -
     5                        0.00         0.00000        -       -
     6                        0.00         0.00000        -       -
     7        b1             36.73         0.05108       YES     YES
     8        a2             56.99         0.00000       NO      YES
     9        a1             69.06         0.09718       YES     YES
    10        a2             88.54         0.00000       NO      YES
    11        b1            105.28         9.05023       YES     YES
    12        a2            137.15         0.00000       NO      YES
    13        b1            138.49         5.71777       YES     YES
    14        b2            180.18         5.48128       YES     YES
    15        a2            229.05         0.00000       NO      YES
    16        b1            230.09         8.49348       YES     YES
    17        a1            266.95         0.00002       YES     YES
    18        a1            314.42         0.66427       YES     YES
    19        b2            435.91         0.32792       YES     YES
    20        b2            500.48         0.00003       YES     YES
    21        a1            534.11         5.06591       YES     YES
    22        a2            834.71         0.00000       NO      YES
    23        b1            844.76         0.51897       YES     YES
    24        a1            966.01        89.59051       YES     YES
    25        b2            991.20         0.29048       YES     YES
    26        a1           1043.02         1.01927       YES     YES
    27        b2           1068.81         1.50998       YES     YES
    28        a1           1116.71         5.83096       YES     YES
    29        a2           1149.64         0.00000       NO      YES
    30        b2           1153.99       103.97383       YES     YES
    31        b1           1163.54         1.05063       YES     YES
    32        a1           1165.60         2.59338       YES     YES
    33        b2           1165.82       489.23671       YES     YES
    34        a2           1182.97         0.00000       NO      YES
    35        b1           1190.29         3.34741       YES     YES
    36        b2           1225.49        84.99511       YES     YES
    37        a1           1231.46         2.56965       YES     YES
    38        a2           1231.52         0.00000       NO      YES
    39        b1           1242.42        19.96030       YES     YES
    40        a2           1299.15         0.00000       NO      YES
    41        b1           1299.44         0.04840       YES     YES
    42        b2           1348.94        34.93052       YES     YES
    43        a1           1379.69         9.44414       YES     YES
    44        b2           1432.98         1.95554       YES     YES
    45        a1           1461.94         0.36043       YES     YES
    46        b2           1480.16         1.31455       YES     YES
    47        a1           1482.91         0.00269       YES     YES
    48        a2           1487.35         0.00000       NO      YES
    49        b1           1487.36        13.82713       YES     YES
    50        b2           1504.16        15.60393       YES     YES
    51        a1           1504.59         1.59846       YES     YES
    52        b2           1516.35         7.30279       YES     YES
    53        a1           1526.70         0.72492       YES     YES
    54        b2           1531.15         4.76531       YES     YES
    55        a1           1536.33         1.99266       YES     YES
    56        b2           2965.91        47.27637       YES     YES
    57        a1           2966.41         0.00350       YES     YES
    58        b2           2972.87        53.40256       YES     YES
    59        a1           2976.60        82.32644       YES     YES
    60        b2           2977.58         1.35166       YES     YES
    61        a1           2983.72       138.86146       YES     YES
    62        b1           2990.71         7.63966       YES     YES
    63        a2           2991.42         0.00000       NO      YES
    64        a2           3013.29         0.00000       NO      YES
    65        b1           3013.34       174.95225       YES     YES
    66        b1           3019.33       133.24711       YES     YES
    67        a2           3021.00         0.00000       NO      YES
    68        b2           3112.99        38.74545       YES     YES
    69        a1           3113.06        20.35561       YES     YES
$end

Total COSMO energy + OC correction = -462.6496655211 H
```

**[Mg(G2)_2_]^2+^**

```
Method: (RI-)B3LYP(D3BJ)/def2-TZVPP
Symmetry: c2

Cartesian coordinates in Ångström:
H     2.9998186   -1.4418697   -0.1498989
H     1.1971347    1.1571890    3.2159179
H    -2.9890962   -1.2078965   -1.6631868
H     1.7037310   -1.2250549    2.6256229
H    -1.3546412   -2.9955985   -0.7157116
O    -0.9048405   -1.1619558   -1.5748676
O     1.5338819    0.3688999    1.3136185
C     0.7742487   -2.7431935   -1.0235274
C     2.3029900   -1.7750023    0.6221921
C    -2.1395397   -0.8486982   -2.2450135
C     1.5971594    1.4649481    2.2490345
H    -2.1831814    0.2316341   -2.3473523
H     0.9964649    2.2707524    1.8401377
C     2.2394769   -0.8005528    1.7736834
C    -0.6641440   -2.5756842   -1.4509073
O     0.9746222   -1.8254284    0.0686191
H     2.6022949   -2.7632790    0.9720491
H     2.6300529    1.7902081    2.3641175
H    -2.1468496   -1.3015784   -3.2351927
H     3.2465470   -0.5264626    2.0901980
H    -0.8297012   -3.0664400   -2.4106004
H     1.4626321   -2.4927548   -1.8334762
H     0.9657295   -3.7646335   -0.6937780
Mg    0.0000000    0.0000000   -0.0758697
H    -2.6300529   -1.7902081    2.3641175
H    -0.9964649   -2.2707524    1.8401377
C    -1.5971594   -1.4649481    2.2490345
H    -2.9998186    1.4418697   -0.1498989
H     2.1831814   -0.2316341   -2.3473523
O    -1.5338819   -0.3688999    1.3136185
H    -1.4626321    2.4927548   -1.8334762
H     2.1468496    1.3015784   -3.2351927
O     0.9048405    1.1619558   -1.5748676
C     2.1395397    0.8486982   -2.2450135
H    -3.2465470    0.5264626    2.0901980
C    -2.2394769    0.8005528    1.7736834
C    -2.3029900    1.7750023    0.6221921
C    -0.7742487    2.7431935   -1.0235274
H     0.8297012    3.0664400   -2.4106004
C     0.6641440    2.5756842   -1.4509073
O    -0.9746222    1.8254284    0.0686191
H    -1.1971347   -1.1571890    3.2159179
H    -2.6022949    2.7632790    0.9720491
H     2.9890962    1.2078965   -1.6631868
H    -0.9657295    3.7646335   -0.6937780
H    -1.7037310    1.2250549    2.6256229
H     1.3546412    2.9955985   -0.7157116

SCF energy GEOOPT =  -1125.037062688 H
ZPE =   1088. kJ/mol
FREEH energy =  1151.99 kJ/mol
FREEH entropy =   0.68456 kJ/mol/K

$vibrational spectrum
#  mode     symmetry     wave number   IR intensity    selection rules
#                         cm**(-1)        km/mol         IR     RAMAN
     1                       -0.00         0.00000        -       -
     2                       -0.00         0.00000        -       -
     3                       -0.00         0.00000        -       -
     4                        0.00         0.00000        -       -
     5                        0.00         0.00000        -       -
     6                        0.00         0.00000        -       -
     7        a              41.10         0.21948       YES     YES
     8        b              43.41         0.03717       YES     YES
     9        a              52.53         0.21106       YES     YES
    10        a              65.57         0.21357       YES     YES
    11        b              78.72         0.15901       YES     YES
    12        b              84.04         0.50801       YES     YES
    13        a              92.48         0.43801       YES     YES
    14        a             113.67         0.06900       YES     YES
    15        b             115.91         3.64690       YES     YES
    16        a             130.30         3.01451       YES     YES
    17        b             131.59         0.27620       YES     YES
    18        a             152.48         1.29898       YES     YES
    19        b             156.02         3.35981       YES     YES
    20        a             165.39         1.43443       YES     YES
    21        b             173.07         0.17958       YES     YES
    22        a             175.36         0.17038       YES     YES
    23        b             194.66         0.02743       YES     YES
    24        a             197.28         0.87035       YES     YES
    25        b             208.85         6.48842       YES     YES
    26        a             211.89         9.44357       YES     YES
    27        b             223.06         1.94918       YES     YES
    28        a             245.37         0.00138       YES     YES
    29        b             265.92         1.02075       YES     YES
    30        a             269.18         0.72082       YES     YES
    31        b             292.94         1.14156       YES     YES
    32        a             293.15         1.13238       YES     YES
    33        b             306.26        13.65279       YES     YES
    34        a             323.83        55.90228       YES     YES
    35        b             325.20        51.16604       YES     YES
    36        b             352.80         3.69289       YES     YES
    37        a             359.03         0.08236       YES     YES
    38        a             373.55         0.25519       YES     YES
    39        b             426.99        33.85016       YES     YES
    40        a             427.46        32.35908       YES     YES
    41        b             453.98        58.82091       YES     YES
    42        a             556.22         0.07927       YES     YES
    43        b             558.50         3.39087       YES     YES
    44        a             572.67         1.04408       YES     YES
    45        b             572.84         0.99477       YES     YES
    46        b             830.14         8.52186       YES     YES
    47        a             830.61         8.14478       YES     YES
    48        b             842.14        22.43925       YES     YES
    49        a             842.70        20.07339       YES     YES
    50        b             878.39       107.72677       YES     YES
    51        a             894.62         0.99572       YES     YES
    52        a             963.22        16.62555       YES     YES
    53        b             963.23        20.42939       YES     YES
    54        b            1015.02        30.90254       YES     YES
    55        a            1015.21        31.88234       YES     YES
    56        b            1028.10         5.77166       YES     YES
    57        a            1033.41         3.67162       YES     YES
    58        a            1053.66       229.82802       YES     YES
    59        b            1054.25       251.63065       YES     YES
    60        a            1069.66        42.51634       YES     YES
    61        b            1071.26        13.49693       YES     YES
    62        a            1089.12         0.00020       YES     YES
    63        b            1095.63       503.33286       YES     YES
    64        b            1117.05        44.43252       YES     YES
    65        a            1117.15        26.20342       YES     YES
    66        a            1122.24         4.31210       YES     YES
    67        b            1122.37         7.70114       YES     YES
    68        b            1143.53         7.61980       YES     YES
    69        a            1143.74         0.11243       YES     YES
    70        b            1179.06         0.18405       YES     YES
    71        a            1179.07         1.41347       YES     YES
    72        b            1182.09         3.50464       YES     YES
    73        a            1182.75         1.02953       YES     YES
    74        b            1217.89         6.86579       YES     YES
    75        a            1218.06         3.27709       YES     YES
    76        b            1225.16        13.72870       YES     YES
    77        a            1225.73         2.45119       YES     YES
    78        b            1260.64         0.15272       YES     YES
    79        a            1260.78         3.21921       YES     YES
    80        a            1271.81        20.56469       YES     YES
    81        b            1272.46        24.08202       YES     YES
    82        b            1291.77        11.17883       YES     YES
    83        a            1292.23         7.48213       YES     YES
    84        b            1305.68        22.25572       YES     YES
    85        a            1305.96         0.01956       YES     YES
    86        b            1385.61        13.30640       YES     YES
    87        a            1385.65        12.76049       YES     YES
    88        a            1413.94         0.11836       YES     YES
    89        b            1416.82         9.69020       YES     YES
    90        a            1428.02         0.71851       YES     YES
    91        b            1428.06         0.51447       YES     YES
    92        b            1457.80         0.49684       YES     YES
    93        a            1458.40         0.01123       YES     YES
    94        a            1484.37         0.73696       YES     YES
    95        b            1484.75         0.89456       YES     YES
    96        a            1484.97         0.53795       YES     YES
    97        b            1486.66         1.93812       YES     YES
    98        a            1488.29        11.30388       YES     YES
    99        b            1488.57         4.04544       YES     YES
   100        b            1494.24        20.21312       YES     YES
   101        a            1494.37        12.36018       YES     YES
   102        a            1501.97         2.59641       YES     YES
   103        b            1502.40        24.53983       YES     YES
   104        a            1503.46         1.94596       YES     YES
   105        b            1505.53        12.42065       YES     YES
   106        b            1509.07        13.30145       YES     YES
   107        a            1510.77         9.17795       YES     YES
   108        b            1516.41         0.16961       YES     YES
   109        a            1518.59         0.32595       YES     YES
   110        b            1519.09        14.30249       YES     YES
   111        a            1521.36        26.51658       YES     YES
   112        b            1524.69        22.70204       YES     YES
   113        a            1525.83         0.29449       YES     YES
   114        a            3039.75         0.02514       YES     YES
   115        b            3039.76        10.95009       YES     YES
   116        a            3040.52         0.08160       YES     YES
   117        b            3041.50        23.09011       YES     YES
   118        b            3042.91         5.59610       YES     YES
   119        a            3042.91         9.83502       YES     YES
   120        b            3043.09        16.06423       YES     YES
   121        a            3044.46        13.30192       YES     YES
   122        b            3045.67         0.83809       YES     YES
   123        a            3045.94         0.52394       YES     YES
   124        a            3047.77        29.73894       YES     YES
   125        b            3047.88        33.01605       YES     YES
   126        b            3095.47         9.27971       YES     YES
   127        a            3095.52         0.44848       YES     YES
   128        b            3096.27         2.94921       YES     YES
   129        a            3096.33         2.29701       YES     YES
   130        b            3105.53        20.00948       YES     YES
   131        a            3105.59        19.94641       YES     YES
   132        b            3107.80         0.03505       YES     YES
   133        a            3107.94         0.48037       YES     YES
   134        b            3117.84         9.26118       YES     YES
   135        a            3118.23         2.33566       YES     YES
   136        b            3121.04         2.46271       YES     YES
   137        a            3121.23         8.31562       YES     YES
   138        a            3153.72         0.09954       YES     YES
   139        b            3153.79        19.94684       YES     YES
   140        a            3165.62         2.05242       YES     YES
   141        b            3165.74         8.71432       YES     YES
$end

Total COSMO energy + OC correction = -1125.2248579199 H
```

SP energy of [Mg(G2)_2_]^+^ with the optimized structure for [Mg(G2)_2_]^2+^ = -‍1125.19776684326 H (A very small negative HOMO/LUMO gap of 0.01 eV is found in the radical cation).

**[Mg(G2)(TfO)_2_]**

```
Method: (RI-)B3LYP(D3BJ)/def2-TZVPP
Symmetry: c1

Cartesian coordinates in Ångström:
Mg   -7.7883360   -0.1985047    2.2213359
F    -9.0783459    2.7215503    4.1667636
F    -7.4686681    4.1706414    4.0233407
C    -7.8032687    2.9724337    4.4981243
F    -7.7056477    2.9851404    5.8271293
S    -6.6836938    1.6655086    3.7941672
O    -6.9592474    1.6986914    2.3361965
O    -7.2370923    0.3931777    4.2883368
O    -5.3646462    2.0258195    4.2104190
F    -8.8098869   -1.6964509   -3.1350919
F    -6.8714143   -1.1612544   -2.3171238
C    -8.1849165   -0.9170809   -2.2489532
F    -8.3975911    0.3605462   -2.5771180
S    -8.8240198   -1.2681264   -0.5420328
O    -8.4950678   -2.6525931   -0.2852893
O   -10.2245325   -0.9250397   -0.5705259
O    -8.0057871   -0.3251767    0.2640566
H    -4.4386237   -0.5951861    3.2024519
H    -6.0750858   -2.3295342    4.0687449
H   -10.6136855   -0.7024782    4.1648411
H   -11.2066207    1.6773089    2.5939380
C    -4.7396372   -0.8979554    2.1981973
C    -6.2275582   -2.5952632    3.0189869
C   -10.5776658   -0.8989441    3.0913276
C   -10.5774977    1.1885772    1.8496867
O    -8.5006774   -2.0050669    3.0248560
O    -6.0704288   -1.4261875    2.2115230
H    -4.7506738   -0.0305138    1.5453060
H   -10.2676016   -2.9981759    3.4720795
O    -9.8189254    0.1400861    2.4673682
H    -7.8531725   -3.9245816    3.4807692
C    -9.8976691   -2.2227977    2.7970101
C    -7.6267184   -3.1154859    2.7827386
H   -11.5909850   -0.9141813    2.6898953
H    -5.4948961   -3.3496093    2.7232036
H    -9.8591726    1.9011162    1.4557556
H    -4.0529259   -1.6488149    1.8054279
H   -11.1675236    0.7771251    1.0327004
H   -10.0532544   -2.5318437    1.7630090
H    -7.7548590   -3.4525962    1.7536079

SCF energy GEOOPT =  -2585.948413051 H
ZPE =   698.1 kJ/mol
FREEH energy =   775.40 kJ/mol
FREEH entropy =   0.83070 kJ/mol/K

$vibrational spectrum
#  mode     symmetry     wave number   IR intensity    selection rules
#                         cm**(-1)        km/mol         IR     RAMAN
     1                       -0.00         0.00000        -       -
     2                        0.00         0.00000        -       -
     3                        0.00         0.00000        -       -
     4                        0.00         0.00000        -       -
     5                        0.00         0.00000        -       -
     6                        0.00         0.00000        -       -
     7        a              10.22         0.89404       YES     YES
     8        a              25.57         0.21836       YES     YES
     9        a              28.94         0.71076       YES     YES
    10        a              33.35         1.19730       YES     YES
    11        a              46.40         0.67046       YES     YES
    12        a              47.89         0.45948       YES     YES
    13        a              54.83         1.35921       YES     YES
    14        a              64.18         1.63367       YES     YES
    15        a              78.91         2.14962       YES     YES
    16        a              85.54         2.36367       YES     YES
    17        a              90.89         4.59473       YES     YES
    18        a             108.97         3.29414       YES     YES
    19        a             113.18         8.76911       YES     YES
    20        a             117.56         4.65843       YES     YES
    21        a             132.42         0.43313       YES     YES
    22        a             140.16         1.72424       YES     YES
    23        a             151.54         1.38173       YES     YES
    24        a             159.84         1.76194       YES     YES
    25        a             175.10         0.18831       YES     YES
    26        a             194.87         2.39447       YES     YES
    27        a             204.20         2.01744       YES     YES
    28        a             206.63         0.59700       YES     YES
    29        a             213.06         1.31823       YES     YES
    30        a             214.81         6.57248       YES     YES
    31        a             231.69         5.65567       YES     YES
    32        a             246.29        34.15056       YES     YES
    33        a             266.00         2.11980       YES     YES
    34        a             285.52        10.86946       YES     YES
    35        a             297.14        10.20577       YES     YES
    36        a             307.38         3.50278       YES     YES
    37        a             321.04        13.35742       YES     YES
    38        a             321.83        23.29237       YES     YES
    39        a             331.90        35.35824       YES     YES
    40        a             334.91         3.03412       YES     YES
    41        a             344.61         2.57310       YES     YES
    42        a             363.36         4.53879       YES     YES
    43        a             374.48         7.66217       YES     YES
    44        a             416.52        36.54746       YES     YES
    45        a             433.29        27.24831       YES     YES
    46        a             453.40       124.02889       YES     YES
    47        a             509.04         9.39048       YES     YES
    48        a             510.31        21.03600       YES     YES
    49        a             519.11         3.04639       YES     YES
    50        a             540.06        15.86911       YES     YES
    51        a             549.69         2.61280       YES     YES
    52        a             566.02         0.65301       YES     YES
    53        a             567.83         5.32200       YES     YES
    54        a             569.79         6.35655       YES     YES
    55        a             587.95         2.93293       YES     YES
    56        a             596.83        45.96781       YES     YES
    57        a             643.02       194.89700       YES     YES
    58        a             653.02       155.36973       YES     YES
    59        a             761.33         2.86402       YES     YES
    60        a             762.66         3.73890       YES     YES
    61        a             831.58         5.25604       YES     YES
    62        a             852.98        20.67972       YES     YES
    63        a             890.42        35.61704       YES     YES
    64        a             960.78        10.04576       YES     YES
    65        a            1009.98       336.12391       YES     YES
    66        a            1022.36       116.47045       YES     YES
    67        a            1024.97       143.94016       YES     YES
    68        a            1035.11        63.32491       YES     YES
    69        a            1078.08       183.29828       YES     YES
    70        a            1084.04        12.60940       YES     YES
    71        a            1105.97       179.44697       YES     YES
    72        a            1127.25        36.85335       YES     YES
    73        a            1140.09        17.07086       YES     YES
    74        a            1156.54        62.87998       YES     YES
    75        a            1160.16        17.35576       YES     YES
    76        a            1170.37       228.24241       YES     YES
    77        a            1173.28        44.21962       YES     YES
    78        a            1176.54       163.14180       YES     YES
    79        a            1186.36         4.25391       YES     YES
    80        a            1190.54         2.84688       YES     YES
    81        a            1199.98       196.88436       YES     YES
    82        a            1203.03       292.20545       YES     YES
    83        a            1205.43       257.18521       YES     YES
    84        a            1226.32         4.08987       YES     YES
    85        a            1233.61       181.51698       YES     YES
    86        a            1237.52       118.09364       YES     YES
    87        a            1264.77         0.26942       YES     YES
    88        a            1276.97        19.24106       YES     YES
    89        a            1299.83         2.56051       YES     YES
    90        a            1312.00        40.06633       YES     YES
    91        a            1324.67       295.97948       YES     YES
    92        a            1355.71       325.57566       YES     YES
    93        a            1387.62        21.45378       YES     YES
    94        a            1413.46         7.04614       YES     YES
    95        a            1423.53         1.89390       YES     YES
    96        a            1460.42         0.66493       YES     YES
    97        a            1485.05         2.28923       YES     YES
    98        a            1486.43         0.57308       YES     YES
    99        a            1494.04        15.81934       YES     YES
   100        a            1494.33         7.33481       YES     YES
   101        a            1499.56         9.62720       YES     YES
   102        a            1500.82        17.04553       YES     YES
   103        a            1506.87         7.00363       YES     YES
   104        a            1508.66         0.76192       YES     YES
   105        a            1515.21        10.16036       YES     YES
   106        a            1530.68         7.59404       YES     YES
   107        a            3023.81        10.98058       YES     YES
   108        a            3031.60        33.03985       YES     YES
   109        a            3035.77        10.90932       YES     YES
   110        a            3039.06         3.00685       YES     YES
   111        a            3040.83        79.81610       YES     YES
   112        a            3044.37        52.23137       YES     YES
   113        a            3071.69        20.89410       YES     YES
   114        a            3090.78         5.15251       YES     YES
   115        a            3099.64        19.84607       YES     YES
   116        a            3103.66         2.14364       YES     YES
   117        a            3105.64        10.62284       YES     YES
   118        a            3122.70         8.46665       YES     YES
   119        a            3155.57         3.93559       YES     YES
   120        a            3157.12         5.23871       YES     YES
$end

Total COSMO energy + OC correction = -2585.9701649150 H
```

**[Mg(MOEA)_3_(H_2_O)]^2+^**

```
Method: (RI-)B3LYP(D3BJ)/def2-TZVPP
Symmetry: c1

Cartesian coordinates in Ångström:
C     0.5529437    2.0755926   -1.8593788
O     0.6132496    1.5370261   -0.5259871
H     1.5405990    2.4230015   -2.1630706
H    -0.1479968    2.9097067   -1.9010879
H     0.2164261    1.2791196   -2.5140202
C     0.9529984    2.5357101    0.4562425
H     1.8189869    3.1046174    0.1157029
C     1.2636504    1.8329283    1.7578258
H     0.1096906    3.2248802    0.5621249
H     2.1926402    1.2722501    1.6652857
N     0.1801461    0.8638236    2.0620322
H     1.3950297    2.5750322    2.5462069
Mg   -0.1687688   -0.2992205    0.2208120
H    -0.6776359    1.3699539    2.2694603
H     0.4091404    0.3686025    2.9174469
H    -3.1055906    1.0797056   -1.5369234
C    -2.7742360    1.4558608   -0.5675714
H    -3.6178353    1.9029281   -0.0428432
H    -3.4449208   -1.1704002   -0.3611213
H    -1.9984067    2.2005376   -0.7068254
H    -1.3674857   -2.6645203    0.3621023
C    -3.1817750   -0.6223358    0.5493081
O    -2.2136396    0.3936345    0.2230022
N    -1.2447062   -2.0059846    1.1279341
H    -4.0860049   -0.1526526    0.9381896
C    -2.5749211   -1.5329591    1.5909180
H    -3.2611003   -2.3561459    1.7942459
H    -0.8130635   -2.5466989    1.8697018
H    -2.4297717   -0.9850806    2.5216069
H     2.4244381    0.0836251   -1.8635818
H     1.9917124   -1.6152629   -3.5665058
C     2.8252925   -0.9275426   -1.8147973
H     3.8281608   -0.8973859   -2.2480321
N     1.9272092   -1.8208823   -2.5740317
H     2.2103390   -2.7910609   -2.4708570
C     3.0027036   -1.3558347   -0.3784528
H     3.7639514   -0.7260280    0.0872316
O     1.7960346   -1.2715867    0.4225139
C     2.0596783   -1.8471880    1.7163073
H     2.8993446   -1.3392322    2.1931433
H     3.3540299   -2.3905841   -0.3357812
H     2.2892723   -2.9082259    1.6166578
H     1.1735691   -1.7271559    2.3296545
O    -0.3560438   -1.1054391   -1.6177587
H     0.4975050   -1.4947216   -2.0798205
H    -1.0935791   -1.1372871   -2.2304889

SCF energy GEOOPT =  -1025.210958360 H
ZPE =   1089. kJ/mol
FREEH energy =  1153.06 kJ/mol
FREEH entropy =   0.68536 kJ/mol/K

$vibrational spectrum
#  mode     symmetry     wave number   IR intensity    selection rules
#                         cm**(-1)        km/mol         IR     RAMAN
     1                       -0.00         0.00000        -       -
     2                        0.00         0.00000        -       -
     3                        0.00         0.00000        -       -
     4                        0.00         0.00000        -       -
     5                        0.00         0.00000        -       -
     6                        0.00         0.00000        -       -
     7        a              37.70         0.29923       YES     YES
     8        a              46.07         0.15887       YES     YES
     9        a              55.91         0.49779       YES     YES
    10        a              59.54         0.26991       YES     YES
    11        a              71.09         0.03647       YES     YES
    12        a              86.66         0.80591       YES     YES
    13        a             100.21         0.41067       YES     YES
    14        a             103.16         0.90129       YES     YES
    15        a             125.57         2.71347       YES     YES
    16        a             138.59         2.97737       YES     YES
    17        a             147.71         5.22862       YES     YES
    18        a             153.94         2.99880       YES     YES
    19        a             158.45         3.32753       YES     YES
    20        a             161.43        11.40789       YES     YES
    21        a             169.22         1.05894       YES     YES
    22        a             182.08         3.10499       YES     YES
    23        a             193.70         1.58194       YES     YES
    24        a             212.60         3.63627       YES     YES
    25        a             220.18         5.30254       YES     YES
    26        a             240.10         1.47883       YES     YES
    27        a             249.40        11.22661       YES     YES
    28        a             266.48        19.66964       YES     YES
    29        a             279.60        35.50903       YES     YES
    30        a             282.76        55.34629       YES     YES
    31        a             293.07         1.85452       YES     YES
    32        a             301.25         6.58044       YES     YES
    33        a             309.98        23.06678       YES     YES
    34        a             313.66         5.15257       YES     YES
    35        a             343.53        44.84980       YES     YES
    36        a             352.94        31.40089       YES     YES
    37        a             373.32        21.31303       YES     YES
    38        a             400.62        18.35360       YES     YES
    39        a             413.89        32.24915       YES     YES
    40        a             418.27        11.04132       YES     YES
    41        a             472.85         8.94630       YES     YES
    42        a             501.71        26.88927       YES     YES
    43        a             513.68        40.77510       YES     YES
    44        a             529.18         3.20695       YES     YES
    45        a             581.64        11.79470       YES     YES
    46        a             614.16        58.57234       YES     YES
    47        a             774.18        99.62242       YES     YES
    48        a             821.50         3.70412       YES     YES
    49        a             835.23        11.12694       YES     YES
    50        a             836.93         8.68750       YES     YES
    51        a             895.20        18.94886       YES     YES
    52        a             902.03        51.25385       YES     YES
    53        a             903.77        15.55936       YES     YES
    54        a             981.63       107.99099       YES     YES
    55        a             987.43       142.59208       YES     YES
    56        a             992.86        21.13621       YES     YES
    57        a             999.01         7.53256       YES     YES
    58        a            1020.73        14.53235       YES     YES
    59        a            1022.63        22.15458       YES     YES
    60        a            1038.86        78.99935       YES     YES
    61        a            1048.87        72.82808       YES     YES
    62        a            1053.53       140.68094       YES     YES
    63        a            1098.34        99.44721       YES     YES
    64        a            1100.45       127.89966       YES     YES
    65        a            1111.89        10.89859       YES     YES
    66        a            1115.40        66.56944       YES     YES
    67        a            1126.58        53.69687       YES     YES
    68        a            1127.72        73.64226       YES     YES
    69        a            1169.32        32.77533       YES     YES
    70        a            1174.82         1.86488       YES     YES
    71        a            1177.93         1.87343       YES     YES
    72        a            1184.45        22.70336       YES     YES
    73        a            1216.52        12.01129       YES     YES
    74        a            1219.26         9.82747       YES     YES
    75        a            1220.57         4.55141       YES     YES
    76        a            1221.81         1.91224       YES     YES
    77        a            1224.31         1.45496       YES     YES
    78        a            1235.13        17.03282       YES     YES
    79        a            1311.92        10.63659       YES     YES
    80        a            1313.77         9.36599       YES     YES
    81        a            1326.74        11.55804       YES     YES
    82        a            1332.41        17.07052       YES     YES
    83        a            1335.49         8.09685       YES     YES
    84        a            1339.05         4.23935       YES     YES
    85        a            1412.85         5.47074       YES     YES
    86        a            1414.63         6.84047       YES     YES
    87        a            1423.64        12.33621       YES     YES
    88        a            1446.68         1.75987       YES     YES
    89        a            1448.64         1.48761       YES     YES
    90        a            1453.59         6.45707       YES     YES
    91        a            1482.61         1.47714       YES     YES
    92        a            1485.54         0.99905       YES     YES
    93        a            1488.10        16.08901       YES     YES
    94        a            1489.13         1.35309       YES     YES
    95        a            1499.81         4.06275       YES     YES
    96        a            1501.47        11.47250       YES     YES
    97        a            1504.52         1.67648       YES     YES
    98        a            1505.28        15.49822       YES     YES
    99        a            1507.81        15.01491       YES     YES
   100        a            1509.34        10.74178       YES     YES
   101        a            1509.99         4.55230       YES     YES
   102        a            1511.32         8.07211       YES     YES
   103        a            1517.80        16.15561       YES     YES
   104        a            1522.63        11.20620       YES     YES
   105        a            1525.34        15.92347       YES     YES
   106        a            1655.05        69.07222       YES     YES
   107        a            1658.84        34.34831       YES     YES
   108        a            1660.23        29.38308       YES     YES
   109        a            1706.96       131.51965       YES     YES
   110        a            2301.75      2341.49009       YES     YES
   111        a            3018.86        15.97646       YES     YES
   112        a            3024.28        15.12655       YES     YES
   113        a            3025.50        11.17974       YES     YES
   114        a            3039.18         7.86325       YES     YES
   115        a            3039.32        20.45362       YES     YES
   116        a            3041.90        18.06391       YES     YES
   117        a            3045.00         5.27373       YES     YES
   118        a            3058.79         6.35077       YES     YES
   119        a            3062.57         7.04093       YES     YES
   120        a            3071.05        10.42990       YES     YES
   121        a            3087.63         9.63596       YES     YES
   122        a            3087.80         5.99846       YES     YES
   123        a            3105.03         8.56728       YES     YES
   124        a            3105.50        11.63176       YES     YES
   125        a            3108.38        16.26286       YES     YES
   126        a            3110.58         5.34637       YES     YES
   127        a            3111.51        19.00512       YES     YES
   128        a            3111.95         1.02953       YES     YES
   129        a            3164.26        22.48763       YES     YES
   130        a            3169.43         5.03864       YES     YES
   131        a            3171.73         9.96728       YES     YES
   132        a            3469.95        16.02293       YES     YES
   133        a            3470.34         8.72541       YES     YES
   134        a            3478.05        20.43854       YES     YES
   135        a            3537.99        13.03150       YES     YES
   136        a            3539.36        23.12959       YES     YES
   137        a            3541.91        25.38249       YES     YES
   138        a            3877.94        88.36973       YES     YES
$end

Total COSMO energy + OC correction = -1025.4046146171 H
```

**[Mg(MOEA)_3_(OH)]^+^**

```
Method: (RI-)B3LYP(D3BJ)/def2-TZVPP
Symmetry: c1

Cartesian coordinates in Ångström:
C     1.1134893    1.8738041   -2.0728910
O     0.7261215    1.7143181   -0.7093605
H     2.1313356    2.2611988   -2.1349420
H     0.4323908    2.5528437   -2.5912019
H     1.0778399    0.8928707   -2.5344170
C     0.7614341    2.9300828    0.0513308
H     1.6178108    3.5319086   -0.2579215
C     0.8737876    2.5506215    1.5169270
H    -0.1501672    3.5034465   -0.1472813
H     1.8680814    2.1484110    1.7081515
N    -0.1019865    1.4945003    1.8024059
H     0.7559549    3.4417325    2.1377783
Mg    0.0098513   -0.1211174    0.2908657
H    -1.0228430    1.8711686    1.9897148
H     0.1539171    0.9013639    2.5866545
H    -3.2926968    1.1891375   -1.5550748
C    -2.6021816    1.4214394   -0.7410981
H    -3.0963093    2.1044757   -0.0443773
H    -4.0467665   -0.6198746   -0.3652313
H    -1.7141780    1.8965561   -1.1469042
H    -3.2519478   -3.0873381   -0.5964466
C    -3.3194608   -0.4995052    0.4452399
O    -2.1915041    0.2292377   -0.0788376
N    -2.4654084   -2.7700687   -0.0400408
H    -3.7872499    0.1018230    1.2306006
C    -2.9102282   -1.8393385    1.0105425
H    -3.7676429   -2.2186504    1.5777277
H    -2.0694279   -3.5973979    0.3903181
H    -2.0760306   -1.6950938    1.6984977
H     1.4044542   -1.4561435   -2.7559679
H    -0.7361663   -0.9205131   -2.2781591
C     0.9677712   -2.0255181   -1.9355016
H     0.7741986   -3.0350538   -2.3046280
N    -0.2625931   -1.3429271   -1.4891393
H    -0.9498673   -1.9972121   -1.0835124
C     1.9667901   -2.0880160   -0.7978941
H     2.9274892   -2.4686640   -1.1532672
O     2.1248194   -0.7580155   -0.3004158
C     3.1232022   -0.6599045    0.7154459
H     4.0759122   -1.0451056    0.3477173
H     1.6093803   -2.7341058    0.0098834
H     2.8107138   -1.2026936    1.6086267
H     3.2350975    0.3956589    0.9513687
O     0.0667121   -1.2381286    1.8072344
H     0.3188505   -2.1199436    2.0658001

SCF energy GEOOPT =  -1024.888400285 H
ZPE =   1051. kJ/mol
FREEH energy =  1116.84 kJ/mol
FREEH entropy =   0.70067 kJ/mol/K

$vibrational spectrum
#  mode     symmetry     wave number   IR intensity    selection rules
#                         cm**(-1)        km/mol         IR     RAMAN
     1                       -0.00         0.00000        -       -
     2                        0.00         0.00000        -       -
     3                        0.00         0.00000        -       -
     4                        0.00         0.00000        -       -
     5                        0.00         0.00000        -       -
     6                        0.00         0.00000        -       -
     7        a              32.11         0.05208       YES     YES
     8        a              45.20         0.17291       YES     YES
     9        a              50.20         0.86239       YES     YES
    10        a              65.36         0.28707       YES     YES
    11        a              78.62         1.03427       YES     YES
    12        a              79.14         0.29664       YES     YES
    13        a              83.09         0.19590       YES     YES
    14        a             103.59         0.92832       YES     YES
    15        a             112.83         2.56773       YES     YES
    16        a             124.40         2.12461       YES     YES
    17        a             128.47         0.88951       YES     YES
    18        a             142.09        12.74094       YES     YES
    19        a             144.69        12.84722       YES     YES
    20        a             157.84         4.43282       YES     YES
    21        a             164.38         9.93182       YES     YES
    22        a             169.58        16.27173       YES     YES
    23        a             184.68        13.13747       YES     YES
    24        a             196.06         4.77793       YES     YES
    25        a             200.88         4.49001       YES     YES
    26        a             220.60        19.95186       YES     YES
    27        a             229.07        56.12917       YES     YES
    28        a             241.99        17.85064       YES     YES
    29        a             252.17        19.49725       YES     YES
    30        a             258.31         9.09098       YES     YES
    31        a             287.17        33.80548       YES     YES
    32        a             299.38        42.37083       YES     YES
    33        a             306.68        42.05664       YES     YES
    34        a             311.50         2.31411       YES     YES
    35        a             328.21        23.21514       YES     YES
    36        a             344.02         0.39215       YES     YES
    37        a             362.28        17.95186       YES     YES
    38        a             389.10        13.13278       YES     YES
    39        a             400.51        22.59411       YES     YES
    40        a             410.63        61.15434       YES     YES
    41        a             439.47        85.05021       YES     YES
    42        a             497.32        12.83752       YES     YES
    43        a             535.78         3.36793       YES     YES
    44        a             557.58        11.79355       YES     YES
    45        a             578.95        12.50399       YES     YES
    46        a             590.34         6.73841       YES     YES
    47        a             631.73       114.22968       YES     YES
    48        a             824.97         9.27949       YES     YES
    49        a             830.41         5.59050       YES     YES
    50        a             841.65         7.66251       YES     YES
    51        a             883.41        59.78369       YES     YES
    52        a             911.63        16.15197       YES     YES
    53        a             919.91        34.17577       YES     YES
    54        a             943.11       205.59458       YES     YES
    55        a             951.58       121.25837       YES     YES
    56        a            1003.82         5.51212       YES     YES
    57        a            1008.81        54.76621       YES     YES
    58        a            1018.36         0.82662       YES     YES
    59        a            1041.50        29.74239       YES     YES
    60        a            1044.31         8.10653       YES     YES
    61        a            1063.27         4.55041       YES     YES
    62        a            1074.92        43.97237       YES     YES
    63        a            1076.91        68.33763       YES     YES
    64        a            1080.59       103.42030       YES     YES
    65        a            1114.38        43.83314       YES     YES
    66        a            1116.14        78.92538       YES     YES
    67        a            1120.61       171.83940       YES     YES
    68        a            1153.34        71.30764       YES     YES
    69        a            1175.78         1.40327       YES     YES
    70        a            1177.94         0.59541       YES     YES
    71        a            1178.59         1.44100       YES     YES
    72        a            1212.36         4.82304       YES     YES
    73        a            1215.32         3.87596       YES     YES
    74        a            1217.30         7.31102       YES     YES
    75        a            1221.37         9.04964       YES     YES
    76        a            1237.07        17.47265       YES     YES
    77        a            1245.71         9.16899       YES     YES
    78        a            1301.61         5.61807       YES     YES
    79        a            1305.07         6.76330       YES     YES
    80        a            1326.26         2.75777       YES     YES
    81        a            1337.56         4.29231       YES     YES
    82        a            1344.42         5.47762       YES     YES
    83        a            1362.78        10.21451       YES     YES
    84        a            1405.58        11.32362       YES     YES
    85        a            1406.20         7.45431       YES     YES
    86        a            1421.10        10.94529       YES     YES
    87        a            1426.91         1.08755       YES     YES
    88        a            1432.34         7.62848       YES     YES
    89        a            1469.13         5.50997       YES     YES
    90        a            1477.30         0.44284       YES     YES
    91        a            1480.89         0.89915       YES     YES
    92        a            1485.00         1.40791       YES     YES
    93        a            1487.78         3.02443       YES     YES
    94        a            1490.71         6.46349       YES     YES
    95        a            1496.03         4.57346       YES     YES
    96        a            1498.21        10.08121       YES     YES
    97        a            1501.17         6.03560       YES     YES
    98        a            1503.42         4.59202       YES     YES
    99        a            1503.61         9.37920       YES     YES
   100        a            1505.82        16.59235       YES     YES
   101        a            1508.28         3.52947       YES     YES
   102        a            1513.42        12.04416       YES     YES
   103        a            1518.89         7.59401       YES     YES
   104        a            1523.27         8.12658       YES     YES
   105        a            1654.50        36.13147       YES     YES
   106        a            1668.92        25.01843       YES     YES
   107        a            1689.15        49.95711       YES     YES
   108        a            2994.41        15.33904       YES     YES
   109        a            3001.38        78.77984       YES     YES
   110        a            3009.81        14.05657       YES     YES
   111        a            3013.08        60.13758       YES     YES
   112        a            3015.50        18.68290       YES     YES
   113        a            3025.01        38.08790       YES     YES
   114        a            3027.89        33.05968       YES     YES
   115        a            3040.56        36.59311       YES     YES
   116        a            3041.22        25.69697       YES     YES
   117        a            3041.84        25.19295       YES     YES
   118        a            3060.81        28.60502       YES     YES
   119        a            3068.08        18.75090       YES     YES
   120        a            3071.33        18.64610       YES     YES
   121        a            3072.61        13.39571       YES     YES
   122        a            3088.53        17.45548       YES     YES
   123        a            3089.34        37.95716       YES     YES
   124        a            3096.98        17.11178       YES     YES
   125        a            3098.15        13.40539       YES     YES
   126        a            3130.70        20.09285       YES     YES
   127        a            3150.62         9.22511       YES     YES
   128        a            3163.06        11.84524       YES     YES
   129        a            3207.63       507.21703       YES     YES
   130        a            3478.66        41.47969       YES     YES
   131        a            3488.08         1.75486       YES     YES
   132        a            3542.06        10.83337       YES     YES
   133        a            3561.69         6.79521       YES     YES
   134        a            3565.16        23.60453       YES     YES
   135        a            3954.67        14.50001       YES     YES
$end

Total COSMO energy + OC correction = -1024.9451574422 H
```

**[Mg(MOEA)_2_(OH)]^+^**

```
Method: (RI-)B3LYP(D3BJ)/def2-TZVPP
Symmetry: c1

Cartesian coordinates in Ångström:
Mg   -0.3022895    0.8061409    0.1263875
O    -1.1263738    2.4320004    0.2917767
H    -2.3619643   -2.2021271    1.5948445
H    -1.0577402   -1.1396927    3.3315104
H     0.7246177    0.5493926    3.8924407
H     1.2743527    1.9824130    1.8878616
C    -0.2753650   -0.8577434    2.6248537
C     0.2269489    0.5398057    2.9213565
C    -1.4939753   -2.0677073    0.9488677
N     1.1063616    0.9844112    1.8143436
O    -0.8106466   -0.8570053    1.2900467
H    -1.8173457   -1.9778808   -0.0830075
H     2.0097427    0.5276034    1.8793614
H    -0.6147040    1.2308521    2.9606915
H     0.5279122   -1.5994483    2.6785650
H    -0.8214070   -2.9210886    1.0492541
H     0.3846860   -0.7732479   -2.9900209
H    -1.3263548    0.9067236   -3.6493953
H    -2.1594177   -0.3702899   -1.8521865
H     2.4516026   -1.1816423   -1.6523266
C    -0.8400091    0.9965765   -2.6765787
C     0.4935708    0.2798051   -2.7128223
N    -1.6579444    0.4616545   -1.5666972
O     1.0662430    0.3460687   -1.3907299
C     2.4108165   -0.1385212   -1.3339299
H     1.1698765    0.7605687   -3.4216393
H    -0.6805736    2.0549391   -2.4734989
H    -2.3343078    1.1621291   -1.2731300
H     2.7453211   -0.0585123   -0.3043462
H     3.0547308    0.4671733   -1.9721450
H    -1.1566745    3.3126792    0.6488526

SCF energy GEOOPT =  -775.1911908956 H
ZPE =   714.2 kJ/mol
FREEH energy =   760.23 kJ/mol
FREEH entropy =   0.56396 kJ/mol/K

$vibrational spectrum
#  mode     symmetry     wave number   IR intensity    selection rules
#                         cm**(-1)        km/mol         IR     RAMAN
     1                       -0.00         0.00000        -       -
     2                       -0.00         0.00000        -       -
     3                        0.00         0.00000        -       -
     4                        0.00         0.00000        -       -
     5                        0.00         0.00000        -       -
     6                        0.00         0.00000        -       -
     7        a              24.74         0.15607       YES     YES
     8        a              47.98         0.17496       YES     YES
     9        a              57.48         0.61384       YES     YES
    10        a              72.54         5.55774       YES     YES
    11        a              97.07         5.44099       YES     YES
    12        a             108.64         4.99512       YES     YES
    13        a             130.62         4.72356       YES     YES
    14        a             131.85         9.23762       YES     YES
    15        a             139.76        42.89597       YES     YES
    16        a             171.43         2.45531       YES     YES
    17        a             189.10         7.42029       YES     YES
    18        a             192.11         2.14605       YES     YES
    19        a             210.51         0.47600       YES     YES
    20        a             244.09        23.31221       YES     YES
    21        a             268.16        42.36118       YES     YES
    22        a             275.80        53.08312       YES     YES
    23        a             284.87        15.27023       YES     YES
    24        a             311.22         4.32000       YES     YES
    25        a             325.95        33.73964       YES     YES
    26        a             345.17        52.32619       YES     YES
    27        a             376.20        68.03676       YES     YES
    28        a             401.70         7.67423       YES     YES
    29        a             424.90        69.79799       YES     YES
    30        a             492.75         8.74385       YES     YES
    31        a             503.03        12.32409       YES     YES
    32        a             591.81         3.92769       YES     YES
    33        a             601.22         6.16926       YES     YES
    34        a             687.91       128.85270       YES     YES
    35        a             829.73         3.93331       YES     YES
    36        a             838.89         4.20983       YES     YES
    37        a             901.82        61.06901       YES     YES
    38        a             905.39        18.08780       YES     YES
    39        a             978.02       262.99866       YES     YES
    40        a             987.48        20.56737       YES     YES
    41        a            1015.29         6.43811       YES     YES
    42        a            1025.78         5.65028       YES     YES
    43        a            1056.87        15.52241       YES     YES
    44        a            1059.22        57.28910       YES     YES
    45        a            1089.43       163.82765       YES     YES
    46        a            1094.59        96.58393       YES     YES
    47        a            1106.97        40.24949       YES     YES
    48        a            1109.66       105.83944       YES     YES
    49        a            1178.15         0.61469       YES     YES
    50        a            1178.43         0.87522       YES     YES
    51        a            1216.24         2.15915       YES     YES
    52        a            1217.85         7.43416       YES     YES
    53        a            1227.51         7.13224       YES     YES
    54        a            1230.86         8.77812       YES     YES
    55        a            1306.12         8.17507       YES     YES
    56        a            1307.43         4.20862       YES     YES
    57        a            1335.64         7.27286       YES     YES
    58        a            1344.91         3.56526       YES     YES
    59        a            1408.04         2.95262       YES     YES
    60        a            1411.94        10.35369       YES     YES
    61        a            1436.94         2.27079       YES     YES
    62        a            1442.82         1.59799       YES     YES
    63        a            1484.62         0.05737       YES     YES
    64        a            1485.31         0.25937       YES     YES
    65        a            1488.81         8.69060       YES     YES
    66        a            1491.22         8.77198       YES     YES
    67        a            1500.12         8.70282       YES     YES
    68        a            1503.60        17.82435       YES     YES
    69        a            1504.53         1.97992       YES     YES
    70        a            1505.56         4.26431       YES     YES
    71        a            1520.54         7.10596       YES     YES
    72        a            1521.70         7.67892       YES     YES
    73        a            1631.19        50.62024       YES     YES
    74        a            1645.32        32.44364       YES     YES
    75        a            3016.93        21.52309       YES     YES
    76        a            3019.00        21.01060       YES     YES
    77        a            3034.28        26.59044       YES     YES
    78        a            3035.77        28.96215       YES     YES
    79        a            3052.62        23.56156       YES     YES
    80        a            3052.73         4.13198       YES     YES
    81        a            3078.47        18.46175       YES     YES
    82        a            3079.03        16.05443       YES     YES
    83        a            3101.33        12.56766       YES     YES
    84        a            3103.05        12.81984       YES     YES
    85        a            3106.36         2.74085       YES     YES
    86        a            3109.11         2.92435       YES     YES
    87        a            3153.68        16.85402       YES     YES
    88        a            3159.77        13.61274       YES     YES
    89        a            3469.52        38.05156       YES     YES
    90        a            3485.55        12.28791       YES     YES
    91        a            3555.65        18.96339       YES     YES
    92        a            3561.60        21.77825       YES     YES
    93        a            3994.08        50.24634       YES     YES
$end

Total COSMO energy + OC correction = -775.2571513894 H
```

**[Mg(MOEA)_3_(OH)(H_2_O)]^+^**

```
Method: (RI-)B3LYP(D3BJ)/def2-TZVPP
Symmetry: c1

Cartesian coordinates in Ångström:
C    -0.0998145    1.9855569   -1.8994382
O     0.5846231    1.6944345   -0.6823709
H     0.3602867    2.8422849   -2.3937504
H    -1.1540908    2.1992663   -1.7074336
H    -0.0077396    1.1176273   -2.5440750
C     0.7066938    2.8457564    0.1624644
H     1.1424047    3.6709880   -0.4044808
C     1.6000905    2.4710088    1.3226169
H    -0.2884831    3.1439755    0.5086650
H     2.5965079    2.2351295    0.9490600
N     1.0540458    1.2624535    1.9660618
H     1.6871059    3.3200333    2.0041905
Mg    0.2707757   -0.1776976    0.4799932
H     0.2144462    1.4896111    2.4914424
H     1.7069318    0.8764037    2.6372473
H    -4.0391880   -0.4750994   -2.8404110
C    -4.0442264    0.0542884   -1.8830251
H    -5.0781403    0.2802485   -1.6083317
H    -4.0067980   -2.5702289   -1.5550918
H    -3.4921771    0.9870237   -1.9837829
H    -1.9741090   -4.1496993    0.0926658
C    -4.0520694   -1.9701283   -0.6396489
O    -3.4034178   -0.7155842   -0.8757304
N    -2.0182813   -3.1462596    0.2011171
H    -5.1050227   -1.7873233   -0.3983812
C    -3.3829309   -2.7023855    0.5080972
H    -4.0311956   -3.5410769    0.7751546
H    -1.3726033   -2.8603614    0.9411522
H    -3.3647746   -2.0445550    1.3795859
H     1.2806140   -1.0617354   -2.6341540
H    -0.9328920   -0.7992935   -1.9742994
C     0.8595105   -1.8174197   -1.9697519
H     0.6350288   -2.7017068   -2.5716557
N    -0.3323189   -1.2634396   -1.3050872
H    -0.9213403   -2.0184457   -0.9096965
C     1.8924077   -2.1855613   -0.9267785
H     2.8177847   -2.5206368   -1.3992419
O     2.1604335   -1.0114034   -0.1403371
C     3.1888799   -1.2260583    0.8266573
H     4.0941990   -1.5877039    0.3375443
H     1.5217163   -2.9740453   -0.2651254
H     2.8620766   -1.9455802    1.5801874
H     3.4008816   -0.2685916    1.2950343
O    -0.2245905   -1.5274537    1.7881557
H     0.1518634   -1.8439886    2.6065340
O    -1.7153101    0.4321365    0.9803032
H    -2.4186085    0.1264278    0.3729775
H    -1.6010653   -0.3003915    1.6289914

SCF energy GEOOPT =  -1101.354844533 H
ZPE =   1122. kJ/mol
FREEH energy =  1191.55 kJ/mol
FREEH entropy =   0.73782 kJ/mol/K

$vibrational spectrum
#  mode     symmetry     wave number   IR intensity    selection rules
#                         cm**(-1)        km/mol         IR     RAMAN
     1                       -0.00         0.00000        -       -
     2                       -0.00         0.00000        -       -
     3                       -0.00         0.00000        -       -
     4                       -0.00         0.00000        -       -
     5                        0.00         0.00000        -       -
     6                        0.00         0.00000        -       -
     7        a              28.35         0.24153       YES     YES
     8        a              40.49         0.07364       YES     YES
     9        a              48.98         0.36376       YES     YES
    10        a              56.02         0.78433       YES     YES
    11        a              58.86         1.45714       YES     YES
    12        a              68.46         0.38291       YES     YES
    13        a              76.46         1.46400       YES     YES
    14        a              92.75         0.82616       YES     YES
    15        a             105.82         8.33623       YES     YES
    16        a             110.45         1.00492       YES     YES
    17        a             116.11         1.09631       YES     YES
    18        a             122.01         1.94509       YES     YES
    19        a             127.04         5.37081       YES     YES
    20        a             153.27         0.96931       YES     YES
    21        a             175.01         7.07190       YES     YES
    22        a             179.88        10.80917       YES     YES
    23        a             182.38        11.83941       YES     YES
    24        a             184.93         3.96212       YES     YES
    25        a             199.87         3.49732       YES     YES
    26        a             210.47         3.12412       YES     YES
    27        a             218.48         5.71412       YES     YES
    28        a             225.89         8.63105       YES     YES
    29        a             236.23        11.29363       YES     YES
    30        a             250.20        45.12125       YES     YES
    31        a             266.48         6.69993       YES     YES
    32        a             269.28         2.57166       YES     YES
    33        a             277.53         1.85541       YES     YES
    34        a             298.93        12.32689       YES     YES
    35        a             308.78         2.43609       YES     YES
    36        a             322.52        13.79272       YES     YES
    37        a             337.51        24.84612       YES     YES
    38        a             342.64        12.46645       YES     YES
    39        a             359.90        32.64836       YES     YES
    40        a             378.79        21.16194       YES     YES
    41        a             405.24         4.08871       YES     YES
    42        a             425.73        61.58243       YES     YES
    43        a             444.42         5.07010       YES     YES
    44        a             494.36        46.83640       YES     YES
    45        a             516.31        93.31298       YES     YES
    46        a             536.16        18.09507       YES     YES
    47        a             541.15       103.96285       YES     YES
    48        a             556.81         9.68195       YES     YES
    49        a             591.34        42.90595       YES     YES
    50        a             651.47        54.12836       YES     YES
    51        a             666.15        25.55228       YES     YES
    52        a             793.83        81.17987       YES     YES
    53        a             807.47        13.07083       YES     YES
    54        a             839.65        77.51244       YES     YES
    55        a             841.94        19.89645       YES     YES
    56        a             853.02       140.66210       YES     YES
    57        a             901.08        51.88465       YES     YES
    58        a             914.97        12.00068       YES     YES
    59        a             918.37        27.05871       YES     YES
    60        a             968.42       213.98369       YES     YES
    61        a             987.64       126.99367       YES     YES
    62        a            1004.63        25.02235       YES     YES
    63        a            1018.87        17.50093       YES     YES
    64        a            1019.87        30.95868       YES     YES
    65        a            1056.02         0.57461       YES     YES
    66        a            1064.12        13.03537       YES     YES
    67        a            1071.90        19.32438       YES     YES
    68        a            1074.88        79.79143       YES     YES
    69        a            1089.64        68.54729       YES     YES
    70        a            1101.41       135.99566       YES     YES
    71        a            1111.22        89.48251       YES     YES
    72        a            1117.12       152.00505       YES     YES
    73        a            1132.27        34.01477       YES     YES
    74        a            1163.45        34.96845       YES     YES
    75        a            1177.22         0.81193       YES     YES
    76        a            1178.04         1.91707       YES     YES
    77        a            1179.20         1.82715       YES     YES
    78        a            1205.63         3.04790       YES     YES
    79        a            1216.64         4.08688       YES     YES
    80        a            1219.18         2.73829       YES     YES
    81        a            1224.15         5.09564       YES     YES
    82        a            1239.92        15.22156       YES     YES
    83        a            1250.45        12.99852       YES     YES
    84        a            1305.73         6.64985       YES     YES
    85        a            1309.45         5.82380       YES     YES
    86        a            1311.98         9.77295       YES     YES
    87        a            1334.43         7.77185       YES     YES
    88        a            1366.35         5.22021       YES     YES
    89        a            1389.57         9.12496       YES     YES
    90        a            1403.72        12.62971       YES     YES
    91        a            1410.49         3.63862       YES     YES
    92        a            1411.31         9.10317       YES     YES
    93        a            1423.73         3.88769       YES     YES
    94        a            1441.02         5.32408       YES     YES
    95        a            1441.91         4.72012       YES     YES
    96        a            1480.05         0.87368       YES     YES
    97        a            1481.15         0.53669       YES     YES
    98        a            1482.44         1.54378       YES     YES
    99        a            1485.17         7.07885       YES     YES
   100        a            1487.08         7.58196       YES     YES
   101        a            1494.18         9.42393       YES     YES
   102        a            1496.11        11.75764       YES     YES
   103        a            1500.49         3.63169       YES     YES
   104        a            1501.46         4.85861       YES     YES
   105        a            1503.17         8.16618       YES     YES
   106        a            1504.89        12.60707       YES     YES
   107        a            1507.57         3.89597       YES     YES
   108        a            1515.16        12.90690       YES     YES
   109        a            1517.20         3.77876       YES     YES
   110        a            1519.21         6.92689       YES     YES
   111        a            1656.09        37.53400       YES     YES
   112        a            1678.39        29.90194       YES     YES
   113        a            1691.00        27.27345       YES     YES
   114        a            1703.53        92.99480       YES     YES
   115        a            2993.18        26.97928       YES     YES
   116        a            3004.80        57.51587       YES     YES
   117        a            3013.19        15.17479       YES     YES
   118        a            3021.48        10.72302       YES     YES
   119        a            3023.17        17.98196       YES     YES
   120        a            3024.47        38.58787       YES     YES
   121        a            3028.48        29.22234       YES     YES
   122        a            3032.02        47.96561       YES     YES
   123        a            3037.06        64.18783       YES     YES
   124        a            3041.91        20.07349       YES     YES
   125        a            3060.81        24.17692       YES     YES
   126        a            3068.18        22.86551       YES     YES
   127        a            3069.15        24.29893       YES     YES
   128        a            3070.58        19.27452       YES     YES
   129        a            3084.19        66.19234       YES     YES
   130        a            3088.07        25.14059       YES     YES
   131        a            3094.85        10.23896       YES     YES
   132        a            3094.97        15.03478       YES     YES
   133        a            3114.18        12.17037       YES     YES
   134        a            3130.75       579.06649       YES     YES
   135        a            3137.25        18.03965       YES     YES
   136        a            3155.18        16.81270       YES     YES
   137        a            3340.03       294.95369       YES     YES
   138        a            3415.90       284.34161       YES     YES
   139        a            3484.43        10.43265       YES     YES
   140        a            3516.00       771.77516       YES     YES
   141        a            3553.00         7.92083       YES     YES
   142        a            3561.20        15.53807       YES     YES
   143        a            3571.87        28.34334       YES     YES
   144        a            3930.07        18.77744       YES     YES
$end

Total COSMO energy + OC correction = -1101.4094995496 H
```

**[Mg(MOEA)_2_(OH)_2_]^+^**

```
Method: (RI-)B3LYP(D3BJ)/def2-TZVPP
Symmetry: c1

Cartesian coordinates in Ångström:
O     0.6423734   -1.4717789    0.0200957
Mg   -0.2337711    0.2727182   -0.0035371
O    -1.3199080    1.8520578   -0.0454407
H    -2.8225726   -1.9173442    2.4260574
H    -0.9251749   -1.0883707    3.7907617
H     1.2255064    0.0538964    3.8015993
H     1.7922614    1.3722757    1.7759602
C    -0.4327707   -0.8757698    2.8349397
C     0.5262704    0.2867539    2.9906750
C    -2.2865556   -1.5713975    1.5364988
N     1.1995933    0.5560064    1.7125686
O    -1.4044697   -0.5111321    1.8630637
H    -3.0036569   -1.1758571    0.8198549
H     1.7480510   -0.2499910    1.4194570
H    -0.0337167    1.1787805    3.2745084
H     0.1016860   -1.7663202    2.4907025
H    -1.7318421   -2.4000904    1.0885193
H     0.9693528   -0.5777702   -2.9587704
H    -1.0439119    0.6128740   -3.9670679
H    -1.8090595   -0.8218281   -2.2992000
H     2.7187025   -0.4699248   -1.2365948
C    -0.6920995    0.8060782   -2.9490450
C     0.7896435    0.5008026   -2.8726234
N    -1.4318051    0.0411307   -1.9352680
O     1.2708625    0.9599361   -1.6132232
C     2.6287058    0.6076100   -1.3896580
H     1.3318014    1.0105956   -3.6772062
H    -0.8388394    1.8626818   -2.7297866
H    -2.1602377    0.6203756   -1.5269249
H     2.9538553    1.1292026   -0.4921110
H     3.2501199    0.9325834   -2.2293648
H    -1.5263191    2.4254549    0.6891952
H     0.5479247   -2.1842391   -0.6086353

SCF energy GEOOPT =  -851.2134264734 H
ZPE =   743.1 kJ/mol
FREEH energy =   794.43 kJ/mol
FREEH entropy =   0.59258 kJ/mol/K

$vibrational spectrum
#  mode     symmetry     wave number   IR intensity    selection rules
#                         cm**(-1)        km/mol         IR     RAMAN
     1                       -0.00         0.00000        -       -
     2                       -0.00         0.00000        -       -
     3                        0.00         0.00000        -       -
     4                        0.00         0.00000        -       -
     5                        0.00         0.00000        -       -
     6                        0.00         0.00000        -       -
     7        a              34.79         3.79342       YES     YES
     8        a              43.66         0.95159       YES     YES
     9        a              51.90         0.85967       YES     YES
    10        a              94.97         0.88666       YES     YES
    11        a             103.86         0.56986       YES     YES
    12        a             114.96         1.80574       YES     YES
    13        a             127.15        15.46213       YES     YES
    14        a             138.26         8.24836       YES     YES
    15        a             150.75        14.64769       YES     YES
    16        a             168.94         6.34351       YES     YES
    17        a             187.53        17.61436       YES     YES
    18        a             194.45         2.38664       YES     YES
    19        a             194.97         1.69138       YES     YES
    20        a             218.65        45.26803       YES     YES
    21        a             226.83        31.16099       YES     YES
    22        a             233.79         1.52665       YES     YES
    23        a             256.93       115.06650       YES     YES
    24        a             268.99         9.06776       YES     YES
    25        a             283.43        26.49159       YES     YES
    26        a             296.96        24.53046       YES     YES
    27        a             297.91        11.97926       YES     YES
    28        a             316.18        94.65083       YES     YES
    29        a             320.15        53.20139       YES     YES
    30        a             370.05         7.08794       YES     YES
    31        a             384.99         1.86423       YES     YES
    32        a             429.94         5.55907       YES     YES
    33        a             475.63        77.90950       YES     YES
    34        a             484.00        34.91473       YES     YES
    35        a             521.13        14.56442       YES     YES
    36        a             534.30       228.74154       YES     YES
    37        a             573.52         6.72655       YES     YES
    38        a             580.46        16.46864       YES     YES
    39        a             643.70       104.89060       YES     YES
    40        a             815.15        14.84428       YES     YES
    41        a             845.31        12.47432       YES     YES
    42        a             896.19       181.48417       YES     YES
    43        a             899.92         5.08445       YES     YES
    44        a             930.20       129.85762       YES     YES
    45        a             953.25       110.38511       YES     YES
    46        a            1026.36        12.14106       YES     YES
    47        a            1031.23        20.14212       YES     YES
    48        a            1053.02        29.11194       YES     YES
    49        a            1073.27        19.46659       YES     YES
    50        a            1085.61        29.09707       YES     YES
    51        a            1091.64        42.57961       YES     YES
    52        a            1125.16        77.70062       YES     YES
    53        a            1136.62       136.20448       YES     YES
    54        a            1175.66         0.74613       YES     YES
    55        a            1177.81         2.30549       YES     YES
    56        a            1210.68        11.58895       YES     YES
    57        a            1216.26         4.68786       YES     YES
    58        a            1234.40         3.95061       YES     YES
    59        a            1243.36         8.13406       YES     YES
    60        a            1301.08         4.61221       YES     YES
    61        a            1307.33         6.34740       YES     YES
    62        a            1332.86         7.72277       YES     YES
    63        a            1357.32         2.43343       YES     YES
    64        a            1403.58         6.99358       YES     YES
    65        a            1411.97         8.71874       YES     YES
    66        a            1428.20         3.73875       YES     YES
    67        a            1440.98         4.31523       YES     YES
    68        a            1470.80         2.11243       YES     YES
    69        a            1472.33         1.14084       YES     YES
    70        a            1477.19         4.93565       YES     YES
    71        a            1489.74         8.05211       YES     YES
    72        a            1490.87         7.36847       YES     YES
    73        a            1495.90         3.93797       YES     YES
    74        a            1502.12         3.58405       YES     YES
    75        a            1505.32         6.94642       YES     YES
    76        a            1506.89        18.30109       YES     YES
    77        a            1508.57         3.90368       YES     YES
    78        a            1620.98        31.35921       YES     YES
    79        a            1645.45        24.92993       YES     YES
    80        a            2982.25        27.53606       YES     YES
    81        a            2988.38         2.13057       YES     YES
    82        a            2997.34        69.23854       YES     YES
    83        a            3004.15       148.24966       YES     YES
    84        a            3005.60        62.47124       YES     YES
    85        a            3013.24        37.98230       YES     YES
    86        a            3022.72        87.04674       YES     YES
    87        a            3041.94        11.27220       YES     YES
    88        a            3058.13        27.80348       YES     YES
    89        a            3072.32        19.92812       YES     YES
    90        a            3076.25        25.97537       YES     YES
    91        a            3103.56         4.61391       YES     YES
    92        a            3116.68        18.69498       YES     YES
    93        a            3119.66        28.47364       YES     YES
    94        a            3444.88        26.43203       YES     YES
    95        a            3457.15        61.98686       YES     YES
    96        a            3579.12        13.71368       YES     YES
    97        a            3591.30        17.96046       YES     YES
    98        a            3918.74         2.31665       YES     YES
    99        a            3926.92         2.81279       YES     YES
$end

Total COSMO energy + OC correction = -851.2368824988 H
```

**[Mg(MOEA)_2_(OH)_2_(MOEA)]^+^**

```
Method: (RI-)B3LYP(D3BJ)/def2-TZVPP
Symmetry: c1

Cartesian coordinates in Ångström:
C     0.4537088    2.6557430   -1.6874313
O     1.0528413    1.9918003   -0.5794135
H     1.0170833    3.5600421   -1.9371316
H    -0.5845185    2.8979452   -1.4516393
H     0.4942214    1.9713172   -2.5310283
C     1.2028647    2.8665795    0.5364133
H     1.9372155    3.6435825    0.2969418
C     1.6699094    2.0533957    1.7253830
H     0.2358417    3.3293617    0.7531837
H     2.6228914    1.5765491    1.4877918
N     0.6826891    1.0094054    2.0119686
H     1.8377632    2.7368167    2.5655956
Mg   -0.2725941    0.1956941    0.1622328
H    -0.2319299    1.4225447    2.1956679
H     0.9504650    0.4187002    2.7867524
H    -3.4139630   -0.8342647   -1.5635780
C    -4.0738306   -0.1012577   -1.0837705
H    -5.0469013   -0.1265912   -1.5926229
H    -3.9562438   -2.4033408    0.0493243
H    -3.6284679    0.8865090   -1.1779349
H    -2.9118587   -3.0459916    2.2902338
C    -4.6306343   -1.6932100    0.5486080
O    -4.2081967   -0.3718876    0.2926121
N    -3.2871982   -2.1469875    2.5668985
H    -5.6415429   -1.8488308    0.1433821
C    -4.6392216   -1.9561601    2.0430572
H    -5.3049436   -2.8105439    2.2316322
H    -3.2744088   -2.1100453    3.5771410
H    -5.0803893   -1.0887628    2.5368790
H     1.3614210   -0.3461868   -2.8767212
H    -1.0323773   -0.0274326   -2.6315263
C     0.7174266   -1.1373687   -2.4871435
H     0.5208272   -1.8414229   -3.3040081
N    -0.4966184   -0.5302655   -1.9384446
H    -1.0840104   -1.2380425   -1.4911948
C     1.4406567   -1.8807223   -1.3843471
H     2.3554813   -2.3414652   -1.7737592
O     1.7732195   -0.9540981   -0.3542322
C     2.2863343   -1.6169873    0.7948251
H     3.1226715   -2.2666334    0.5180745
H     0.7905596   -2.6547318   -0.9657138
H     1.4898744   -2.1939315    1.2702789
H     2.6492052   -0.8499377    1.4745464
O    -0.8780457   -1.5951980    0.5816918
H    -1.5942428   -1.6330841    1.2212835
O    -1.5696906    1.6105832    0.4265375
H    -2.4442739    1.3322129    0.7008341

SCF energy GEOOPT =  -1100.885168008 H
ZPE =   1081. kJ/mol
FREEH energy =  1152.47 kJ/mol
FREEH entropy =   0.77270 kJ/mol/K

$vibrational spectrum
#  mode     symmetry     wave number   IR intensity    selection rules
#                         cm**(-1)        km/mol         IR     RAMAN
     1                       -0.00         0.00000        -       -
     2                        0.00         0.00000        -       -
     3                        0.00         0.00000        -       -
     4                        0.00         0.00000        -       -
     5                        0.00         0.00000        -       -
     6                        0.00         0.00000        -       -
     7        a              16.58         2.33736       YES     YES
     8        a              22.91         3.01104       YES     YES
     9        a              32.17         0.32921       YES     YES
    10        a              38.65         2.37857       YES     YES
    11        a              45.27         0.22182       YES     YES
    12        a              59.36         2.13471       YES     YES
    13        a              78.54         3.83289       YES     YES
    14        a              81.20         3.54443       YES     YES
    15        a              85.53         0.67006       YES     YES
    16        a              90.52         1.50686       YES     YES
    17        a             109.83         1.77634       YES     YES
    18        a             121.91         1.02038       YES     YES
    19        a             129.58         3.32984       YES     YES
    20        a             134.21         5.38513       YES     YES
    21        a             139.13         4.65768       YES     YES
    22        a             147.90         2.08857       YES     YES
    23        a             155.72        11.01912       YES     YES
    24        a             182.06        85.69236       YES     YES
    25        a             187.60        57.81293       YES     YES
    26        a             198.97         0.53364       YES     YES
    27        a             200.86         8.96613       YES     YES
    28        a             215.13         4.16662       YES     YES
    29        a             225.45        10.89272       YES     YES
    30        a             230.17         6.73942       YES     YES
    31        a             238.33         4.54189       YES     YES
    32        a             261.17         9.77759       YES     YES
    33        a             274.46        13.68279       YES     YES
    34        a             288.51         8.42245       YES     YES
    35        a             292.50         3.49949       YES     YES
    36        a             301.42         2.41924       YES     YES
    37        a             306.57        10.13214       YES     YES
    38        a             343.39        36.03449       YES     YES
    39        a             366.30         0.22635       YES     YES
    40        a             391.85         5.26244       YES     YES
    41        a             396.79         9.31859       YES     YES
    42        a             483.44         6.31339       YES     YES
    43        a             492.36        22.74851       YES     YES
    44        a             496.93       116.40608       YES     YES
    45        a             516.69        40.76245       YES     YES
    46        a             522.32        11.21252       YES     YES
    47        a             555.40        60.17476       YES     YES
    48        a             572.96        19.13157       YES     YES
    49        a             583.21         3.36597       YES     YES
    50        a             617.32       172.47397       YES     YES
    51        a             620.54       183.39815       YES     YES
    52        a             756.83       119.32264       YES     YES
    53        a             819.09        51.67196       YES     YES
    54        a             843.25        12.23847       YES     YES
    55        a             849.95         8.11034       YES     YES
    56        a             867.06        78.07996       YES     YES
    57        a             906.80        91.70727       YES     YES
    58        a             910.30        28.13141       YES     YES
    59        a             929.08        23.08791       YES     YES
    60        a             951.98       221.66209       YES     YES
    61        a             958.51        26.94889       YES     YES
    62        a            1010.28         0.04259       YES     YES
    63        a            1029.60        52.02761       YES     YES
    64        a            1034.30         7.78933       YES     YES
    65        a            1055.76        20.96749       YES     YES
    66        a            1072.93        28.54262       YES     YES
    67        a            1077.47        14.78475       YES     YES
    68        a            1083.59        17.31258       YES     YES
    69        a            1087.24        29.17019       YES     YES
    70        a            1099.58        26.52490       YES     YES
    71        a            1122.71        43.11946       YES     YES
    72        a            1125.45       160.13822       YES     YES
    73        a            1153.97       120.92596       YES     YES
    74        a            1175.09         2.51999       YES     YES
    75        a            1180.16         4.60187       YES     YES
    76        a            1181.45         2.37034       YES     YES
    77        a            1202.97         6.23067       YES     YES
    78        a            1216.26         3.25663       YES     YES
    79        a            1216.98         9.54860       YES     YES
    80        a            1234.94         0.99826       YES     YES
    81        a            1235.15         3.85430       YES     YES
    82        a            1243.09        14.17194       YES     YES
    83        a            1302.67         6.31000       YES     YES
    84        a            1304.28         6.35125       YES     YES
    85        a            1306.94         3.29932       YES     YES
    86        a            1329.68         5.15477       YES     YES
    87        a            1331.48         5.28418       YES     YES
    88        a            1335.64         6.94175       YES     YES
    89        a            1409.40        11.57247       YES     YES
    90        a            1411.36         3.86709       YES     YES
    91        a            1412.39         7.94711       YES     YES
    92        a            1434.80        14.60120       YES     YES
    93        a            1438.62         1.39079       YES     YES
    94        a            1440.04         6.62517       YES     YES
    95        a            1470.59         4.65793       YES     YES
    96        a            1473.39         0.41757       YES     YES
    97        a            1478.22         5.00028       YES     YES
    98        a            1485.62         9.21204       YES     YES
    99        a            1487.12         5.46073       YES     YES
   100        a            1488.22         4.80527       YES     YES
   101        a            1494.11         3.41482       YES     YES
   102        a            1499.45         8.77645       YES     YES
   103        a            1501.33         4.86434       YES     YES
   104        a            1502.59         5.32374       YES     YES
   105        a            1505.61         3.21584       YES     YES
   106        a            1506.94         8.51357       YES     YES
   107        a            1511.52         7.14843       YES     YES
   108        a            1515.38         5.48586       YES     YES
   109        a            1517.40         3.32481       YES     YES
   110        a            1642.66        31.25848       YES     YES
   111        a            1650.93        41.14135       YES     YES
   112        a            1657.72        23.97188       YES     YES
   113        a            2938.14         4.19108       YES     YES
   114        a            2955.45       103.56803       YES     YES
   115        a            2965.16       156.73161       YES     YES
   116        a            2977.54        63.14621       YES     YES
   117        a            2986.91         3.88376       YES     YES
   118        a            2989.66         4.27904       YES     YES
   119        a            2996.82        68.31343       YES     YES
   120        a            2999.83        75.17160       YES     YES
   121        a            3003.11       187.37031       YES     YES
   122        a            3005.37        75.83050       YES     YES
   123        a            3006.54        96.43385       YES     YES
   124        a            3040.47         5.20924       YES     YES
   125        a            3044.04         5.29972       YES     YES
   126        a            3062.65        38.03136       YES     YES
   127        a            3066.11        36.41630       YES     YES
   128        a            3067.90        23.85749       YES     YES
   129        a            3068.38        13.89693       YES     YES
   130        a            3074.56        12.84860       YES     YES
   131        a            3120.78         7.82613       YES     YES
   132        a            3125.65        24.88057       YES     YES
   133        a            3128.58        29.20949       YES     YES
   134        a            3370.84        91.83445       YES     YES
   135        a            3412.92        56.24193       YES     YES
   136        a            3503.21         0.14121       YES     YES
   137        a            3578.81         9.30689       YES     YES
   138        a            3580.30         9.26261       YES     YES
   139        a            3585.70         2.02881       YES     YES
   140        a            3819.70       181.50988       YES     YES
   141        a            3883.58        24.54202       YES     YES
$end

Total COSMO energy + OC correction = -1100.9109631997 H
```

**[Mg(MOEA)_2_(H_2_O)_2_(TfO)_2_]**

```
Method: (RI-)B3LYP(D3BJ)/def2-TZVPP
Symmetry: c1

Cartesian coordinates in Ångström:
C    -6.8118726    2.2938816   -2.2810594
O    -7.0146999    2.5624343   -0.8993786
H    -5.8078226    2.5930730   -2.5962952
H    -7.5513260    2.8248229   -2.8870834
H    -6.9338969    1.2221509   -2.4199265
C    -6.9138797    3.9385330   -0.5515722
H    -5.8948912    4.2954593   -0.7413764
C    -7.2626667    4.1335809    0.9123963
H    -7.5955185    4.5188312   -1.1859471
H    -6.6549615    3.4676152    1.5257193
N    -8.6706513    3.8417474    1.1945662
H    -6.9793487    5.1553046    1.1882742
Mg   -8.3226279   -0.1496848    1.4584162
H    -9.2704108    4.5846622    0.8558746
H    -8.8256786    3.7499762    2.1990096
H   -11.6707796   -1.6373887    1.1552362
C   -11.3952269   -0.8601584    1.8696344
H   -12.0578240   -0.8821752    2.7365601
H   -10.0149540   -3.1035210    2.3261460
H   -11.4435992    0.1124897    1.3925356
H    -7.4992581   -2.6555353    1.7000501
C    -9.8326420   -2.2674507    3.0068251
O   -10.0434337   -1.0423928    2.2986906
N    -7.4927667   -1.9236225    2.4097399
H   -10.5294870   -2.3234124    3.8459285
C    -8.4057323   -2.2718942    3.5169799
H    -8.1903577   -3.2522551    3.9495513
H    -6.5449336   -1.8234468    2.7500892
H    -8.3004918   -1.5181882    4.2950474
F    -6.1379002    2.7696824    4.6093586
F    -6.3498744    0.8386250    5.5794367
C    -6.9903168    1.9815927    5.2912064
F    -7.3057570    2.5916908    6.4322514
S    -8.5080464    1.6414493    4.2760444
O    -7.8818418    1.0203121    3.0848478
O    -9.0544341    2.9523144    3.9990464
O    -9.2981552    0.7340022    5.0621218
F    -8.4085161   -1.5976044   -3.2415342
F    -8.4481109   -3.7496366   -2.9514675
C    -8.1134533   -2.5891886   -2.3858330
F    -6.7890108   -2.5799392   -2.1824204
S    -9.0247179   -2.3564282   -0.7843024
O   -10.4204224   -2.3740200   -1.1299357
O    -8.5277595   -1.0189254   -0.3799533
O    -8.5375001   -3.4162979    0.0765987
O    -6.4215764    0.5884455    0.8683608
H    -6.0990148    1.0336010    1.6593353
H    -6.5279929    1.2914972    0.1925867
O    -9.4518004    1.3431301    0.6099586
H    -9.1830619    2.3070746    0.7425039
H    -9.5279071    1.1767668   -0.3338326

SCF energy GEOOPT =  -2775.575319210 H
ZPE =   968.6 kJ/mol
FREEH energy =  1065.80 kJ/mol
FREEH entropy =   0.99799 kJ/mol/K

$vibrational spectrum
#  mode     symmetry     wave number   IR intensity    selection rules
#                         cm**(-1)        km/mol         IR     RAMAN
     1                       -0.00         0.00000        -       -
     2                        0.00         0.00000        -       -
     3                        0.00         0.00000        -       -
     4                        0.00         0.00000        -       -
     5                        0.00         0.00000        -       -
     6                        0.00         0.00000        -       -
     7        a               6.60         0.50846       YES     YES
     8        a               9.95         0.85907       YES     YES
     9        a              16.59         0.25457       YES     YES
    10        a              17.53         1.20524       YES     YES
    11        a              32.08         1.83852       YES     YES
    12        a              38.77         0.28588       YES     YES
    13        a              45.22         1.16089       YES     YES
    14        a              49.44         2.55322       YES     YES
    15        a              57.95         0.81615       YES     YES
    16        a              62.79         2.01353       YES     YES
    17        a              69.32         2.02817       YES     YES
    18        a              78.35         0.72029       YES     YES
    19        a              83.24         2.53754       YES     YES
    20        a              85.23         1.44563       YES     YES
    21        a              88.63         1.12146       YES     YES
    22        a             101.30         0.48808       YES     YES
    23        a             108.06         6.82279       YES     YES
    24        a             115.42         8.48808       YES     YES
    25        a             130.36         8.37625       YES     YES
    26        a             140.50         2.36013       YES     YES
    27        a             145.92         0.69110       YES     YES
    28        a             153.30         7.62666       YES     YES
    29        a             158.18         6.81952       YES     YES
    30        a             169.51        10.99654       YES     YES
    31        a             174.88         4.50947       YES     YES
    32        a             196.10         0.31386       YES     YES
    33        a             202.18         1.33153       YES     YES
    34        a             203.72         2.05801       YES     YES
    35        a             211.24        14.26304       YES     YES
    36        a             215.07         2.90312       YES     YES
    37        a             231.87         8.24905       YES     YES
    38        a             235.43         3.52850       YES     YES
    39        a             240.25        10.57598       YES     YES
    40        a             246.91         4.13116       YES     YES
    41        a             272.42        37.17044       YES     YES
    42        a             287.26        17.82290       YES     YES
    43        a             295.12         7.51290       YES     YES
    44        a             302.19         9.34753       YES     YES
    45        a             311.09         0.35344       YES     YES
    46        a             317.79         5.46840       YES     YES
    47        a             332.65         8.46880       YES     YES
    48        a             343.46         0.51884       YES     YES
    49        a             344.37         0.66036       YES     YES
    50        a             354.29        27.90239       YES     YES
    51        a             366.62        16.87342       YES     YES
    52        a             380.40         3.91557       YES     YES
    53        a             397.04        14.69397       YES     YES
    54        a             415.17        38.55529       YES     YES
    55        a             429.11        86.62999       YES     YES
    56        a             441.71        56.10613       YES     YES
    57        a             464.09        68.10815       YES     YES
    58        a             492.26        22.74320       YES     YES
    59        a             508.64        44.02495       YES     YES
    60        a             512.70        32.81680       YES     YES
    61        a             516.09        18.22662       YES     YES
    62        a             518.74        13.50541       YES     YES
    63        a             527.41        14.93840       YES     YES
    64        a             557.65        47.35368       YES     YES
    65        a             567.10         2.73909       YES     YES
    66        a             569.12        12.90457       YES     YES
    67        a             580.71        14.60613       YES     YES
    68        a             583.35        29.81057       YES     YES
    69        a             596.96        46.07255       YES     YES
    70        a             633.80       348.53768       YES     YES
    71        a             638.83       146.88287       YES     YES
    72        a             653.62        31.24375       YES     YES
    73        a             721.27       220.29232       YES     YES
    74        a             754.72         3.85644       YES     YES
    75        a             757.33         4.61345       YES     YES
    76        a             799.51        64.60480       YES     YES
    77        a             828.37        19.44417       YES     YES
    78        a             853.89         8.10169       YES     YES
    79        a             894.65        20.50218       YES     YES
    80        a             910.97        18.60541       YES     YES
    81        a             959.43        80.07276       YES     YES
    82        a             994.59        87.92147       YES     YES
    83        a            1023.96       305.64551       YES     YES
    84        a            1027.09       163.01234       YES     YES
    85        a            1032.39       111.13231       YES     YES
    86        a            1051.00        21.94945       YES     YES
    87        a            1062.61        18.09427       YES     YES
    88        a            1070.61        24.68365       YES     YES
    89        a            1095.06        67.85274       YES     YES
    90        a            1103.47        81.60529       YES     YES
    91        a            1110.13        97.65886       YES     YES
    92        a            1118.31        82.63552       YES     YES
    93        a            1132.34       112.93440       YES     YES
    94        a            1146.87       196.09836       YES     YES
    95        a            1157.13       151.19848       YES     YES
    96        a            1176.25        98.90339       YES     YES
    97        a            1177.83        85.18923       YES     YES
    98        a            1179.50         0.58508       YES     YES
    99        a            1190.05         0.30159       YES     YES
   100        a            1199.14       230.26249       YES     YES
   101        a            1201.36       198.14269       YES     YES
   102        a            1210.35         6.11686       YES     YES
   103        a            1230.60        77.63639       YES     YES
   104        a            1234.31       279.90496       YES     YES
   105        a            1238.45       107.17924       YES     YES
   106        a            1240.95       196.49988       YES     YES
   107        a            1241.77        37.38280       YES     YES
   108        a            1307.22         9.48489       YES     YES
   109        a            1313.21        32.44855       YES     YES
   110        a            1330.75       280.77622       YES     YES
   111        a            1336.36       265.60939       YES     YES
   112        a            1342.90        80.91072       YES     YES
   113        a            1346.00        25.65191       YES     YES
   114        a            1411.03        10.74254       YES     YES
   115        a            1415.96         7.24475       YES     YES
   116        a            1436.50         6.24252       YES     YES
   117        a            1448.69         2.57686       YES     YES
   118        a            1482.56         1.17489       YES     YES
   119        a            1484.35         0.64690       YES     YES
   120        a            1493.04         8.36076       YES     YES
   121        a            1497.59         7.49672       YES     YES
   122        a            1499.46         5.04074       YES     YES
   123        a            1502.25         9.72338       YES     YES
   124        a            1506.28         9.55546       YES     YES
   125        a            1516.07         5.57203       YES     YES
   126        a            1518.17         2.52773       YES     YES
   127        a            1520.39         8.12670       YES     YES
   128        a            1650.80        32.78673       YES     YES
   129        a            1669.67        24.10387       YES     YES
   130        a            1684.59        78.14525       YES     YES
   131        a            1689.74        47.07721       YES     YES
   132        a            2891.35      1752.03863       YES     YES
   133        a            2980.90        43.85371       YES     YES
   134        a            3002.18        54.34696       YES     YES
   135        a            3005.39        20.82514       YES     YES
   136        a            3018.71        47.36336       YES     YES
   137        a            3022.80         2.97500       YES     YES
   138        a            3029.67        37.93679       YES     YES
   139        a            3034.91        62.20741       YES     YES
   140        a            3058.05        30.53259       YES     YES
   141        a            3070.15        20.21581       YES     YES
   142        a            3083.59        23.87194       YES     YES
   143        a            3096.36        14.67021       YES     YES
   144        a            3114.13         3.20200       YES     YES
   145        a            3131.11         7.53500       YES     YES
   146        a            3166.49         2.97748       YES     YES
   147        a            3407.62       205.30660       YES     YES
   148        a            3428.22       108.93921       YES     YES
   149        a            3457.40       899.03140       YES     YES
   150        a            3542.62        38.09536       YES     YES
   151        a            3561.05        25.52712       YES     YES
   152        a            3826.88        90.93723       YES     YES
   153        a            3852.87        65.47619       YES     YES
$end

Total COSMO energy + OC correction = -2775.6021394595 H
```

**[Mg(MOEA)(H_2_O)(OH)(TfO)_2_]^−^**

```
Method: (RI-)B3LYP(D3BJ)/def2-TZVPP
Symmetry: c1

Cartesian coordinates in Ångström:
Mg   -8.2539327   -0.0020231    1.5830002
H   -11.5517319   -1.0154203    1.2026543
C   -11.2654584   -0.4487154    2.0908376
H   -11.9979772   -0.6018264    2.8890738
H   -10.1455217   -2.8284868    1.9712125
H   -11.1819049    0.6076451    1.8492585
H    -7.4806563   -2.6217749    1.4125047
C    -9.8575973   -2.2370972    2.8448483
O    -9.9732621   -0.8503138    2.5402072
N    -7.4948885   -1.9994496    2.2165782
H   -10.5318450   -2.4880537    3.6710663
C    -8.4185117   -2.5020859    3.2468200
H    -8.3011462   -3.5745931    3.4322686
H    -6.5640600   -1.9360298    2.6131424
H    -8.1944155   -1.9714147    4.1699285
F    -7.2558028    0.8026375    6.4642228
F    -6.7849405    2.6887481    5.5002715
C    -6.3657769    1.4299897    5.6776532
F    -5.1949718    1.4711034    6.3340190
S    -6.1735240    0.5439233    4.0556860
O    -7.5374534    0.5984194    3.5180761
O    -5.7380347   -0.7868647    4.4302721
O    -5.2148272    1.3471654    3.3208753
F    -9.4614225   -1.1332025   -3.1191394
F    -9.1396907   -3.2784549   -3.1822059
C    -8.8141630   -2.1496155   -2.5297563
F    -7.4985838   -1.9495631   -2.6839572
S    -9.2708598   -2.2822709   -0.7330387
O   -10.7010895   -2.4892770   -0.7412522
O    -8.8518718   -0.9746197   -0.2180981
O    -8.4666265   -3.3904531   -0.2469796
O    -6.4988172    0.9469010    0.7426860
H    -5.9036773    1.2646239    1.4385469
H    -7.2258300    1.6242602    0.6594146
O    -8.9516795    1.7225718    1.0376257
H    -9.4667377    1.7675473    0.2317870

SCF energy GEOOPT =  -2525.354231270 H
ZPE =   596.8 kJ/mol
FREEH energy =   672.38 kJ/mol
FREEH entropy =   0.80920 kJ/mol/K

$vibrational spectrum
#  mode     symmetry     wave number   IR intensity    selection rules
#                         cm**(-1)        km/mol         IR     RAMAN
     1                       -0.00         0.00000        -       -
     2                       -0.00         0.00000        -       -
     3                        0.00         0.00000        -       -
     4                        0.00         0.00000        -       -
     5                        0.00         0.00000        -       -
     6                        0.00         0.00000        -       -
     7        a              18.48         0.46326       YES     YES
     8        a              19.36         0.65948       YES     YES
     9        a              27.89         1.34400       YES     YES
    10        a              40.94         0.35309       YES     YES
    11        a              44.20         0.17727       YES     YES
    12        a              46.90         0.55575       YES     YES
    13        a              68.47         0.58168       YES     YES
    14        a              74.47         2.56223       YES     YES
    15        a              89.58         1.63876       YES     YES
    16        a              91.26         2.69456       YES     YES
    17        a              96.14         1.63364       YES     YES
    18        a             102.53         2.45691       YES     YES
    19        a             118.45         0.74542       YES     YES
    20        a             138.53         1.00740       YES     YES
    21        a             146.52         5.88503       YES     YES
    22        a             158.12         4.24122       YES     YES
    23        a             164.43        10.54726       YES     YES
    24        a             184.20         8.26660       YES     YES
    25        a             194.68        16.19639       YES     YES
    26        a             206.75         1.23565       YES     YES
    27        a             208.88         2.88691       YES     YES
    28        a             211.94        18.69422       YES     YES
    29        a             228.62        19.42400       YES     YES
    30        a             234.55         8.72958       YES     YES
    31        a             247.35         8.24652       YES     YES
    32        a             257.80        19.73905       YES     YES
    33        a             263.94        28.42744       YES     YES
    34        a             296.34         1.44755       YES     YES
    35        a             306.41        16.88791       YES     YES
    36        a             310.79         5.61064       YES     YES
    37        a             318.88        10.42883       YES     YES
    38        a             334.98        37.18773       YES     YES
    39        a             344.04         0.43453       YES     YES
    40        a             348.66        12.71049       YES     YES
    41        a             361.31         2.49601       YES     YES
    42        a             377.04        28.45303       YES     YES
    43        a             400.81       107.48382       YES     YES
    44        a             404.54        16.54519       YES     YES
    45        a             507.67        30.94255       YES     YES
    46        a             511.70        34.32559       YES     YES
    47        a             513.43         4.90884       YES     YES
    48        a             514.58        18.56090       YES     YES
    49        a             533.26       128.44373       YES     YES
    50        a             540.45       117.70442       YES     YES
    51        a             564.79         6.78291       YES     YES
    52        a             566.05         9.17988       YES     YES
    53        a             570.81        32.38262       YES     YES
    54        a             573.92         2.28716       YES     YES
    55        a             578.53         0.66147       YES     YES
    56        a             620.56        93.92676       YES     YES
    57        a             628.75       468.27815       YES     YES
    58        a             639.18        32.57442       YES     YES
    59        a             683.49        87.58209       YES     YES
    60        a             748.92        47.77974       YES     YES
    61        a             753.40         8.17396       YES     YES
    62        a             776.77       131.52096       YES     YES
    63        a             847.49        11.42921       YES     YES
    64        a             902.99        14.21886       YES     YES
    65        a             978.03       155.62723       YES     YES
    66        a            1004.71        96.43811       YES     YES
    67        a            1030.74       453.77459       YES     YES
    68        a            1036.25       116.10135       YES     YES
    69        a            1047.16        11.52028       YES     YES
    70        a            1079.55        16.06643       YES     YES
    71        a            1111.58       127.83434       YES     YES
    72        a            1129.37        42.31908       YES     YES
    73        a            1148.94       195.08720       YES     YES
    74        a            1149.14        79.77690       YES     YES
    75        a            1160.89       106.81715       YES     YES
    76        a            1160.94       210.68945       YES     YES
    77        a            1193.51         1.44743       YES     YES
    78        a            1212.02        58.14916       YES     YES
    79        a            1212.28        83.68045       YES     YES
    80        a            1228.99        16.26340       YES     YES
    81        a            1241.56        94.89463       YES     YES
    82        a            1245.44       378.73182       YES     YES
    83        a            1250.29       308.54286       YES     YES
    84        a            1293.89       276.69647       YES     YES
    85        a            1301.27       337.83447       YES     YES
    86        a            1311.26        93.80860       YES     YES
    87        a            1351.32         4.56882       YES     YES
    88        a            1410.96         7.30023       YES     YES
    89        a            1443.52         2.52932       YES     YES
    90        a            1474.70         3.25956       YES     YES
    91        a            1499.51         5.60983       YES     YES
    92        a            1501.01         2.39614       YES     YES
    93        a            1512.33         5.19147       YES     YES
    94        a            1532.32         6.06937       YES     YES
    95        a            1647.27        40.13593       YES     YES
    96        a            1647.83        31.91650       YES     YES
    97        a            2997.00         4.59837       YES     YES
    98        a            3003.98        90.55312       YES     YES
    99        a            3014.15       111.32001       YES     YES
   100        a            3049.03        14.59986       YES     YES
   101        a            3075.70        14.08021       YES     YES
   102        a            3111.78        10.51395       YES     YES
   103        a            3138.57         1.80777       YES     YES
   104        a            3195.30       527.66187       YES     YES
   105        a            3450.20        62.10423       YES     YES
   106        a            3543.43       120.00761       YES     YES
   107        a            3699.32       295.90051       YES     YES
   108        a            3888.98         2.64411       YES     YES
$end

Total COSMO energy + OC correction = -2525.4219177792 H
```

**[Mg(MOEA)_2_(H_2_O)(OH)(TfO)]**

```
Method: (RI-)B3LYP(D3BJ)/def2-TZVPP
Symmetry: c1

Cartesian coordinates in Ångström:
C    -5.9871910   -0.6011153   -0.5020086
O    -6.6264723    0.4321332    0.2552146
H    -5.1584002   -1.0335588    0.0674621
H    -5.5995081   -0.1887631   -1.4370309
H    -6.7595416   -1.3401892   -0.7059491
C    -5.7645820    1.5410213    0.5028349
H    -5.0539720    1.2908923    1.2965948
C    -6.5299958    2.7859122    0.8965198
H    -5.1946918    1.7336747   -0.4140471
H    -7.0041686    2.6379874    1.8614520
N    -7.5746450    3.1281463   -0.0830646
H    -5.7850557    3.5799635    1.0182882
Mg   -8.4006642   -0.3479457    1.3337097
H    -7.2336192    3.0494171   -1.0340211
H    -7.8835299    4.0835719    0.0476453
H   -11.6206543   -1.4091039    1.3902042
C   -11.3525868   -0.8089545    2.2630665
H   -12.0113731   -1.0459111    3.1013611
H   -10.0782586   -3.0622690    2.1825166
H   -11.4349745    0.2443152    2.0178101
H    -7.6705512   -2.7874916    1.3567876
C    -9.7728615   -2.4116933    3.0092833
O    -9.9983902   -1.0555320    2.6283704
N    -7.4928016   -2.1926443    2.1614464
H   -10.3746373   -2.6442552    3.8919776
C    -8.3029310   -2.5939919    3.3231452
H    -8.1416612   -3.6364642    3.6158724
H    -6.5075143   -2.2165915    2.3867282
H    -8.0344290   -1.9546890    4.1618937
F    -6.5622834    3.1497805    4.1731486
F    -5.9773966    1.4764009    5.4243986
C    -7.0218254    2.2123441    5.0238483
F    -7.5367870    2.8340912    6.0856816
S    -8.3057818    1.1391902    4.2113574
O    -7.5423606    0.5473382    3.0926904
O    -9.3159484    2.0800942    3.7468483
O    -8.6880747    0.2133086    5.2425447
O    -8.8640186   -1.5112010   -0.1151697
H    -9.4324972   -1.3105260   -0.8559926
O    -9.4383267    1.4311637    1.0500092
H    -8.9093680    2.0574177    0.4954437
H    -9.5377498    1.8558859    1.9241182

SCF energy GEOOPT =  -1813.397032632 H
ZPE =   859.0 kJ/mol
FREEH energy =   931.67 kJ/mol
FREEH entropy =   0.76677 kJ/mol/K

$vibrational spectrum
#  mode     symmetry     wave number   IR intensity    selection rules
#                         cm**(-1)        km/mol         IR     RAMAN
     1                       -0.00         0.00000        -       -
     2                       -0.00         0.00000        -       -
     3                       -0.00         0.00000        -       -
     4                        0.00         0.00000        -       -
     5                        0.00         0.00000        -       -
     6                        0.00         0.00000        -       -
     7        a              14.87         0.42918       YES     YES
     8        a              35.22         0.31114       YES     YES
     9        a              44.36         0.83258       YES     YES
    10        a              50.40         2.10569       YES     YES
    11        a              64.30         5.11266       YES     YES
    12        a              70.31         2.08881       YES     YES
    13        a              73.54         1.21121       YES     YES
    14        a              77.37         1.62024       YES     YES
    15        a              91.48         2.04544       YES     YES
    16        a             101.42         3.20352       YES     YES
    17        a             111.78         4.61521       YES     YES
    18        a             122.49         3.22241       YES     YES
    19        a             137.02         1.26152       YES     YES
    20        a             148.64         5.82353       YES     YES
    21        a             166.51         3.60926       YES     YES
    22        a             175.65        21.80712       YES     YES
    23        a             182.37         0.66712       YES     YES
    24        a             195.85         6.11481       YES     YES
    25        a             201.23         1.23563       YES     YES
    26        a             204.64        16.31536       YES     YES
    27        a             219.46         1.86881       YES     YES
    28        a             226.73         2.91939       YES     YES
    29        a             238.49         1.89988       YES     YES
    30        a             254.07         6.61446       YES     YES
    31        a             264.96        15.17182       YES     YES
    32        a             270.18         4.10009       YES     YES
    33        a             287.34        67.72792       YES     YES
    34        a             294.78        10.68171       YES     YES
    35        a             300.30        10.57741       YES     YES
    36        a             309.89         2.62867       YES     YES
    37        a             317.36         5.44462       YES     YES
    38        a             334.01        24.92719       YES     YES
    39        a             341.72        51.10774       YES     YES
    40        a             348.61         9.03852       YES     YES
    41        a             368.95         9.88335       YES     YES
    42        a             385.62        14.31141       YES     YES
    43        a             398.69        20.08135       YES     YES
    44        a             424.15        32.95937       YES     YES
    45        a             500.60         6.47575       YES     YES
    46        a             510.41        18.22726       YES     YES
    47        a             517.22        54.45451       YES     YES
    48        a             526.84       187.79849       YES     YES
    49        a             533.35        10.06871       YES     YES
    50        a             566.55        17.92852       YES     YES
    51        a             569.35        25.65419       YES     YES
    52        a             580.39         6.63596       YES     YES
    53        a             597.23        30.15804       YES     YES
    54        a             636.83       206.78836       YES     YES
    55        a             692.69        46.60547       YES     YES
    56        a             754.14         6.26327       YES     YES
    57        a             817.58         9.73245       YES     YES
    58        a             840.50        45.01324       YES     YES
    59        a             848.68        89.14297       YES     YES
    60        a             877.51        76.78307       YES     YES
    61        a             904.47        53.41221       YES     YES
    62        a             927.05        15.59932       YES     YES
    63        a             957.40       171.27182       YES     YES
    64        a             982.71       199.53688       YES     YES
    65        a             995.44        31.27693       YES     YES
    66        a            1018.45       229.30825       YES     YES
    67        a            1024.13        20.41721       YES     YES
    68        a            1075.59        49.43690       YES     YES
    69        a            1079.40        47.13516       YES     YES
    70        a            1081.38        13.98200       YES     YES
    71        a            1099.18        27.12718       YES     YES
    72        a            1124.29       103.87627       YES     YES
    73        a            1124.87        93.34564       YES     YES
    74        a            1148.49       196.80846       YES     YES
    75        a            1172.11        44.24607       YES     YES
    76        a            1179.15         0.92453       YES     YES
    77        a            1184.65         4.93885       YES     YES
    78        a            1199.92       227.27597       YES     YES
    79        a            1215.56        52.23418       YES     YES
    80        a            1223.28       147.12187       YES     YES
    81        a            1224.43       113.72455       YES     YES
    82        a            1235.13         0.86865       YES     YES
    83        a            1235.25         6.11903       YES     YES
    84        a            1308.12        11.05368       YES     YES
    85        a            1313.81        38.54759       YES     YES
    86        a            1323.56       193.87955       YES     YES
    87        a            1326.53         2.88894       YES     YES
    88        a            1330.51       127.51411       YES     YES
    89        a            1414.11         6.42910       YES     YES
    90        a            1419.72         8.64393       YES     YES
    91        a            1446.58         4.45758       YES     YES
    92        a            1448.47         6.17833       YES     YES
    93        a            1464.42         7.25973       YES     YES
    94        a            1482.09         0.82098       YES     YES
    95        a            1491.10         8.50369       YES     YES
    96        a            1494.01         7.77031       YES     YES
    97        a            1500.29         4.82638       YES     YES
    98        a            1507.88         9.15236       YES     YES
    99        a            1512.90         0.39874       YES     YES
   100        a            1513.55         9.55381       YES     YES
   101        a            1523.08        10.98976       YES     YES
   102        a            1526.21         5.11354       YES     YES
   103        a            1648.86        27.99733       YES     YES
   104        a            1649.23        49.91450       YES     YES
   105        a            1700.58        54.80294       YES     YES
   106        a            2983.70         9.08361       YES     YES
   107        a            2993.55        81.68046       YES     YES
   108        a            3001.01         6.67388       YES     YES
   109        a            3005.99        84.59902       YES     YES
   110        a            3013.11        68.24361       YES     YES
   111        a            3018.33        69.12224       YES     YES
   112        a            3036.71        34.98113       YES     YES
   113        a            3051.31        35.29649       YES     YES
   114        a            3055.01        39.68188       YES     YES
   115        a            3076.88        25.66197       YES     YES
   116        a            3113.85         0.98209       YES     YES
   117        a            3114.14        14.11474       YES     YES
   118        a            3150.55         0.20405       YES     YES
   119        a            3163.36         5.77681       YES     YES
   120        a            3272.22       963.17768       YES     YES
   121        a            3470.78        15.50442       YES     YES
   122        a            3496.78         1.48190       YES     YES
   123        a            3545.35       441.16551       YES     YES
   124        a            3574.23         7.65611       YES     YES
   125        a            3581.25        12.24155       YES     YES
   126        a            3920.98         5.86140       YES     YES
$end

Total COSMO energy + OC correction = -1813.4208861126 H
```

**[Mg(MOEA)(OH)_2_(TfO)]^−^**

```
Method: (RI-)B3LYP(D3BJ)/def2-TZVPP
Symmetry: c1

Cartesian coordinates in Ångström:
Mg   -7.9431260   -0.2057184    1.6323065
H   -11.4155953   -0.9501732    1.6128236
C   -10.9643740   -0.5193892    2.5094393
H   -11.6151633   -0.6890391    3.3752614
H   -10.1287774   -2.9753893    1.9750312
H   -10.7659406    0.5398941    2.3620974
H    -7.4639868   -2.9817535    1.1804333
C    -9.7094261   -2.5234686    2.8801226
O    -9.6866556   -1.1122089    2.7437687
N    -7.4465656   -2.4274232    2.0270914
H   -10.3381541   -2.8070421    3.7341879
C    -8.2703080   -2.9664614    3.0952431
H    -8.2400299   -4.0604601    3.1781663
H    -6.5031477   -2.1662456    2.2913433
H    -7.9156133   -2.5534660    4.0415175
F    -9.8202956   -0.5535769   -2.9606055
F    -9.7094304   -2.6829483   -3.3604560
C    -9.1603206   -1.6795327   -2.6509288
F    -7.8902670   -1.5387434   -3.0623238
S    -9.2490308   -2.0427998   -0.8288460
O   -10.6658536   -2.1457485   -0.5588019
O    -8.6019636   -0.8557466   -0.2629938
O    -8.4847842   -3.2690447   -0.6867203
O    -6.0668913   -0.1501169    2.0674123
H    -5.6235106    0.6853354    2.2088460
O    -8.8679670    1.4534910    1.8344559
H    -8.3908516    2.2773556    1.7509784

SCF energy GEOOPT =  -1563.155770402 H
ZPE =   480.4 kJ/mol
FREEH energy =   535.05 kJ/mol
FREEH entropy =   0.64109 kJ/mol/K

$vibrational spectrum
#  mode     symmetry     wave number   IR intensity    selection rules
#                         cm**(-1)        km/mol         IR     RAMAN
     1                       -0.00         0.00000        -       -
     2                       -0.00         0.00000        -       -
     3                        0.00         0.00000        -       -
     4                        0.00         0.00000        -       -
     5                        0.00         0.00000        -       -
     6                        0.00         0.00000        -       -
     7        a              17.00         2.08267       YES     YES
     8        a              30.94         0.63370       YES     YES
     9        a              54.99         0.45435       YES     YES
    10        a              56.44         5.30592       YES     YES
    11        a              65.82         4.28670       YES     YES
    12        a              79.88         0.63255       YES     YES
    13        a              94.55         0.23244       YES     YES
    14        a             103.83        43.54114       YES     YES
    15        a             128.52         0.97168       YES     YES
    16        a             143.70         1.71898       YES     YES
    17        a             159.67         3.39655       YES     YES
    18        a             174.40         2.84408       YES     YES
    19        a             202.12         1.99920       YES     YES
    20        a             205.83         3.44291       YES     YES
    21        a             213.51        27.29811       YES     YES
    22        a             218.78        31.72942       YES     YES
    23        a             235.86        26.86764       YES     YES
    24        a             248.31        16.32737       YES     YES
    25        a             262.97        11.78574       YES     YES
    26        a             285.31         0.12082       YES     YES
    27        a             308.68         6.19601       YES     YES
    28        a             319.54        13.60941       YES     YES
    29        a             330.53       142.75256       YES     YES
    30        a             343.18         0.11675       YES     YES
    31        a             371.41        22.74618       YES     YES
    32        a             396.82        12.09038       YES     YES
    33        a             489.78       169.09207       YES     YES
    34        a             505.30        17.48279       YES     YES
    35        a             511.20       123.68106       YES     YES
    36        a             518.47        86.42761       YES     YES
    37        a             534.65        24.74376       YES     YES
    38        a             565.15         3.98261       YES     YES
    39        a             573.60        21.73625       YES     YES
    40        a             577.81        37.60550       YES     YES
    41        a             611.62        70.56170       YES     YES
    42        a             626.81       186.19620       YES     YES
    43        a             658.43       100.33627       YES     YES
    44        a             750.80         9.85785       YES     YES
    45        a             836.70        53.16023       YES     YES
    46        a             874.23       192.31762       YES     YES
    47        a             921.39         2.78381       YES     YES
    48        a            1021.39         1.12703       YES     YES
    49        a            1034.87       241.19481       YES     YES
    50        a            1035.73        86.60031       YES     YES
    51        a            1099.10        38.88804       YES     YES
    52        a            1131.53        77.83196       YES     YES
    53        a            1141.71       141.77129       YES     YES
    54        a            1154.61       172.40409       YES     YES
    55        a            1191.28         0.34947       YES     YES
    56        a            1211.50        71.21046       YES     YES
    57        a            1217.83         3.94530       YES     YES
    58        a            1241.63         5.59037       YES     YES
    59        a            1252.54       399.31940       YES     YES
    60        a            1289.66        31.57274       YES     YES
    61        a            1301.98       281.29491       YES     YES
    62        a            1345.14         3.98487       YES     YES
    63        a            1400.67        11.01394       YES     YES
    64        a            1420.21         3.98876       YES     YES
    65        a            1467.03         9.13725       YES     YES
    66        a            1492.96         1.44520       YES     YES
    67        a            1495.51         5.56784       YES     YES
    68        a            1516.60         4.41960       YES     YES
    69        a            1527.42         4.27482       YES     YES
    70        a            1667.63        16.95155       YES     YES
    71        a            2967.03         7.24317       YES     YES
    72        a            2976.52        77.57379       YES     YES
    73        a            2983.95       231.98483       YES     YES
    74        a            3026.76         7.96453       YES     YES
    75        a            3061.99        28.73015       YES     YES
    76        a            3063.37        32.07053       YES     YES
    77        a            3126.35        12.73417       YES     YES
    78        a            3472.48        63.16999       YES     YES
    79        a            3571.30       121.63486       YES     YES
    80        a            3896.43         0.68522       YES     YES
    81        a            3906.94         0.82629       YES     YES
$end

Total COSMO energy + OC correction = -1563.2353639080 H
```

**[Mg(MOEA)(H_2_O)(OH)(TfO)]**

```
Method: (RI-)B3LYP(D3BJ)/def2-TZVPP
Symmetry: c1

Cartesian coordinates in Ångström:
Mg   -8.2129808   -0.0882446    1.7048973
H   -11.3516749   -1.0427364    1.1227950
C   -11.2033788   -0.6261126    2.1204288
H   -11.9871037   -0.9748469    2.7955556
H    -9.8122046   -2.8658945    1.7409069
H   -11.2142395    0.4593594    2.0701777
H    -7.1626663   -2.4703020    1.6459694
C    -9.7313886   -2.4140565    2.7340721
O    -9.9206352   -1.0003094    2.6287279
N    -7.3529735   -1.9310841    2.4917855
H   -10.4996303   -2.8406655    3.3838554
C    -8.3516113   -2.6330304    3.3221999
H    -8.1568785   -3.7053035    3.3969933
H    -6.4749856   -1.8252639    2.9847958
H    -8.3190979   -2.2100739    4.3261496
F   -10.2580395   -2.8537717   -1.7218574
F    -8.6365752   -2.9577030   -3.1631366
C    -9.2316810   -2.1792172   -2.2596454
F    -9.7122745   -1.0951099   -2.8713463
S    -8.0227744   -1.7003515   -0.9352134
O    -7.6146895   -2.9498734   -0.3250809
O    -8.8777711   -0.8872910   -0.0339994
O    -6.9990974   -0.9255471   -1.5973972
O    -6.3719575    0.5046716    0.8376790
H    -6.5975110    1.4317491    1.0341576
H    -6.3190719    0.3809873   -0.1219390
O    -8.2570338    1.6939206    2.2006925
H    -8.7595736    2.2836111    2.7535762

SCF energy GEOOPT =  -1563.706239605 H
ZPE =   519.9 kJ/mol
FREEH energy =   573.73 kJ/mol
FREEH entropy =   0.62648 kJ/mol/K

$vibrational spectrum
#  mode     symmetry     wave number   IR intensity    selection rules
#                         cm**(-1)        km/mol         IR     RAMAN
     1                       -0.00         0.00000        -       -
     2                       -0.00         0.00000        -       -
     3                       -0.00         0.00000        -       -
     4                       -0.00         0.00000        -       -
     5                        0.00         0.00000        -       -
     6                        0.00         0.00000        -       -
     7        a              26.87         1.26325       YES     YES
     8        a              38.35         0.71162       YES     YES
     9        a              54.29         0.89549       YES     YES
    10        a              64.11         0.97466       YES     YES
    11        a              75.49         6.35037       YES     YES
    12        a              85.19         1.45696       YES     YES
    13        a             107.72         2.73267       YES     YES
    14        a             114.11         5.01310       YES     YES
    15        a             137.42         7.14892       YES     YES
    16        a             155.13        12.03038       YES     YES
    17        a             160.77        33.34145       YES     YES
    18        a             182.13         1.06446       YES     YES
    19        a             196.63         3.92094       YES     YES
    20        a             200.83        10.46413       YES     YES
    21        a             213.10         4.41896       YES     YES
    22        a             241.66        22.97947       YES     YES
    23        a             250.91        83.45968       YES     YES
    24        a             255.35        17.37327       YES     YES
    25        a             269.48         8.80329       YES     YES
    26        a             300.68        15.50322       YES     YES
    27        a             316.92        11.46926       YES     YES
    28        a             325.49        18.67972       YES     YES
    29        a             346.87         2.46377       YES     YES
    30        a             367.87        54.48889       YES     YES
    31        a             407.04         3.20356       YES     YES
    32        a             416.82        12.44802       YES     YES
    33        a             453.20       146.95794       YES     YES
    34        a             480.97        34.42508       YES     YES
    35        a             506.39        53.82056       YES     YES
    36        a             513.62        34.84967       YES     YES
    37        a             527.93        26.74314       YES     YES
    38        a             565.03        22.41275       YES     YES
    39        a             576.51        16.87203       YES     YES
    40        a             617.12        28.54298       YES     YES
    41        a             632.95       400.54226       YES     YES
    42        a             668.95       116.02933       YES     YES
    43        a             675.21       111.44207       YES     YES
    44        a             753.19        44.99773       YES     YES
    45        a             768.21        25.72294       YES     YES
    46        a             846.09         7.81785       YES     YES
    47        a             909.27        11.99173       YES     YES
    48        a             996.71        74.21025       YES     YES
    49        a            1020.35       293.46156       YES     YES
    50        a            1046.35         5.74007       YES     YES
    51        a            1072.27        36.33294       YES     YES
    52        a            1108.43       183.73904       YES     YES
    53        a            1116.64        30.43719       YES     YES
    54        a            1169.47       167.29818       YES     YES
    55        a            1178.96        42.61883       YES     YES
    56        a            1182.25         2.29103       YES     YES
    57        a            1201.67       285.44132       YES     YES
    58        a            1221.01         3.24573       YES     YES
    59        a            1229.43       240.47154       YES     YES
    60        a            1235.55        66.86675       YES     YES
    61        a            1306.26        48.35921       YES     YES
    62        a            1313.37       314.89020       YES     YES
    63        a            1353.67         6.25333       YES     YES
    64        a            1407.73         5.89350       YES     YES
    65        a            1437.74         3.65564       YES     YES
    66        a            1481.12         0.28738       YES     YES
    67        a            1495.37         9.21393       YES     YES
    68        a            1501.43         8.41390       YES     YES
    69        a            1502.73         2.53400       YES     YES
    70        a            1520.51         8.92823       YES     YES
    71        a            1591.52        79.60955       YES     YES
    72        a            1658.89        27.21800       YES     YES
    73        a            3018.93        10.98662       YES     YES
    74        a            3026.31        42.10268       YES     YES
    75        a            3038.80        32.35261       YES     YES
    76        a            3063.26        28.26513       YES     YES
    77        a            3092.55        33.27627       YES     YES
    78        a            3093.98         9.12172       YES     YES
    79        a            3142.84         6.01986       YES     YES
    80        a            3407.70       140.89223       YES     YES
    81        a            3556.17        34.13943       YES     YES
    82        a            3619.94        84.91334       YES     YES
    83        a            3733.48       329.86521       YES     YES
    84        a            3975.78        22.94222       YES     YES
$end

Total COSMO energy + OC correction = -1563.7291104511 H
```

All thermodynamic values calculated for this work are summarized in **Table S1**–**Table S4** (tables) and **Scheme S1**–**Scheme S 7** (reaction schemes).

**Table S1**: Calculated reaction enthalpies Δ_r_H° and free reaction enthalpies Δ_r_G° in the gas phase and in a G2 solution (RI-B3LYP(D3BJ)/def2-TZVPP; ε_r_ = 7.23).^[9]^

| **Reaction** | **Δ_r_*H*° (gas phase) [kJ mol^−1^]** | **Δ_r_*G*° (gas phase) [kJ mol^−1^]** | **Δ_r_*G*° (solution) [kJ mol^−1^]** |
| --- | --- | --- | --- |
| [Al(Ohfip)_4_]^−^ + MOEA 🡪 [Al(Ohfip)_3_(MOEA^−H^)]^−^ + HOhfip | +100 | +103 | +65 |
| [Al(Ohfip)_4_]^−^ + H_2_O 🡪 [Al(Ohfip)_3_(OH)]^−^ + HOhfip | +48 | +43 | −119 |
| MOEA + H_2_O 🡪 [MOEA–H]^+^ + [OH]^−^ | +745 | +750 | +120 |
| 2 H_2_O + MOEA 🡪 OH^−^ + [(H_2_O)∙∙∙H-MOEA]^+^ | +678 | +720 | +6 |
| [Mg(MOEA)_3_]^2+^ + 2 G2 🡪 [Mg(G2)_2_]^2+^ + 3 MOEA | +43 | +4 | +11 |
| [Mg(G2)_2_]^2+^ + e^−^ 🡪 [Mg(G2)_2_]^⋅+^ (single point calculation on structure of dication) | −422 | −422 | −72 |
| [Mg(MOEA)_3_]^2+^ + e^−^ 🡪 [Mg(MOEA)_3_]^⋅+^ (single point calculation on structure of dication) | −524 | −524 | −149 |
| [Mg(MOEA)_3_]^2+^ + 2 [Ohfip]^−^ 🡪 [Mg(MOEA)_2_(Ohfip)_2_] + MOEA | −1178 | −1136 | −269 |

**Table S2**: Calculated reaction enthalpies Δ_r_H° and free reaction enthalpies Δ_r_G° in the gas phase and in a G2 solution (RI-B3LYP(D3BJ)/def2-TZVPP; ε_r_ = 7.23).

| **Reaction** | **Δ_r_*H*° (gas phase) [kJ mol^−1^]** | **Δ_r_*G*° (gas phase) [kJ mol^−1^]** | **Δ_r_*G*° (solution) [kJ mol^−1^]** |
| --- | --- | --- | --- |
| [Al(Ohfip)_4_]^−^ + OH^−^ 🡪 [Al(Ohfip)_4_OH]^2−^ | −96 | −49 | −62 |
| [Al(Ohfip)_4_OH]^2−^ 🡪 [Al(Ohfip)_3_OH]^−^ + [Ohfip]^−^ | −94 | −149 | −46 |
| [Ohfip]^−^ + [(H_2_O)∙∙∙H-MOEA]^+^ 🡪 [(H_2_O)∙∙∙MOEA] + HOhfip | −469 | −471 | −121 |
| 2 [Al(Ohfip)_3_OH]^−^ 🡪 [(Ohfip)_3_Al(OH)_2_Al(Ohfip)_3_]^2−^ | +86 | +157 | +44 |
| [(Ohfip)_3_Al(OH)_2_Al(Ohfip)_3_]^2−^ + [Mg(MOEA)_3_]^2+^ 🡪 [(Ohfip)_6_Al_2_(OH)_2_Mg(MOEA)] + 2 MOEA | −881 | −929 | −127 |
|  |  |  |  |
| [Mg(MOEA)_2_(TfO)_2_] 🡪 [Mg(TfO)_2_] + 2 MOEA | +359 | +246 | +222 |
| [Mg(MOEA)_2_(TfO)_2_] 🡪 [Mg(MOEA)( TfO)_2_] + MOEA | +157 | +100 | +85 |
| [Mg(MOEA)_2_(TfO)_2_] + MOEA 🡪 [Mg(MOEA)_3_(TfO)_2_] | −5 | +52 | +43 |
| [Mg(MOEA)_2_(TfO)_2_] + MOEA 🡪 [Mg(MOEA)_3_]^2+^ + 2 [TfO]^−^ | +1046 | +1005 | +173 |
| [Mg(MOEA)_2_(TfO)_2_] + 2 G2 🡪 [Mg(G2)_2_]^2+^ + 2 MOEA + 2 [TfO]^−^ | +1089 | +1009 | +183 |
| [Mg(MOEA)_2_(TfO)_2_] + G2 🡪 [Mg(G2)( TfO)_2_] + 2 MOEA | +123 | +71 | +56 |

**Table S3**: Calculated reaction enthalpies Δ_r_H° and free reaction enthalpies Δ_r_G° in the gas phase and in a G2 solution (RI-B3LYP(D3BJ)/def2-TZVPP; ε_r_ = 7.23).

| **Reaction** | **Δ_r_*H*° (gas phase) [kJ mol^−1^]** | **Δ_r_*G*° (gas phase) [kJ mol^−1^]** | **Δ_r_*G*° (solution) [kJ mol^−1^]** |
| --- | --- | --- | --- |
| [MOEA(H_2_O)] + [Mg(MOEA)_3_]^2+^ 🡪 [HMOEA]^+^ + [Mg(MOEA)_3_(OH)]^+^ | −163 | −158 | +37 |
| [MOEA(H_2_O)] + [Mg(MOEA)_3_(OH)]^+^ 🡪 [HMOEA]^+^ + [Mg(MOEA)_3_(OH)_2_] | +193 | +200 | +109 |
| [MOEA(H_2_O)] + [Mg(MOEA)_3_]^2+^ 🡪 MOEA + [Mg(MOEA)_3_(H_2_O)]^2+^ | −37 | −28 | 0 |
| [Mg(MOEA)_3_(H_2_O)]^2+^ 🡪 [Mg(MOEA)_2_(OH)]^+^ + [HMOEA]^+^ | −35 | −98 | +23 |
| [Mg(MOEA)_2_(OH)]^+^ + [MOEA(H_2_O)] 🡪 [Mg(MOEA)_3_(OH)(H_2_O)]^+^ | −149 | −80 | −16 |
| [Mg(MOEA)_3_(OH)(H_2_O)]^+^ 🡪 [HMOEA]^+^ + [Mg(MOEA)_2_(OH)_2_] | +280 | +225 | +91 |
| [Mg(MOEA)_2_(OH)_2_] + MOEA 🡪 [Mg(MOEA)_2_(OH)_2_(MOEA)] | −23 | +23 | +39 |

**Table S4**: Calculated reaction enthalpies Δ_r_H° and free reaction enthalpies Δ_r_G° in the gas phase and in a G2 solution (RI-B3LYP(D3BJ)/def2-TZVPP; ε_r_ = 7.23).

| **Reaction** | **Δ_r_*H*° (gas phase) [kJ mol^−1^]** | **Δ_r_*G*° (gas phase) [kJ mol^−1^]** | **Δ_r_*G*° (solution) [kJ mol^−1^]** |
| --- | --- | --- | --- |
| [Mg(MOEA)_2_(TfO)_2_] + 2 [MOEA(H_2_O)] 🡪 2 MOEA + [Mg(MOEA)_2_(H_2_O)_2_(TfO)_2_] | −27 | −25 | −5 |
| [Mg(MOEA)_2_(H_2_O)_2_(TfO)_2_] 🡪 [HMOEA]^+^ + [Mg(MOEA)(H_2_O)(OH)( TfO)_2_]^−^ | +493 | +450 | +128 |
| [Mg(MOEA)(H_2_O)(OH)( TfO)_2_]^−^ + MOEA 🡪 [Mg(MOEA)_2_(H_2_O)(OH)( TfO)] + [TfO]^−^ | +68 | +76 | +16 |
| [Mg(MOEA)_2_(H_2_O)(OH)( TfO)] 🡪 [HMOEA]^+^ + [Mg(MOEA)(OH)_2_(TfO)]^−^ | +543 | +481 | +120 |
| [Mg(MOEA)(OH)_2_(TfO)]^−^ + MOEA 🡪 [TfO]^−^ + [Mg(MOEA)_2_(OH)_2_] | +29 | +39 | +11 |
| [Mg(MOEA)_2_(H_2_O)_2_(TfO)_2_] 🡪 [HMOEA]^+^ + [TfO]^−^ + [Mg(MOEA)(H_2_O)(OH)( TfO)] | +634 | +542 | +140 |

**Scheme S1**: Calculated reaction enthalpies Δ_r_H° and free reaction enthalpies Δ_r_G° in the gas phase and in a G2 solution for a reaction path describing the decomposition of the [Al(Ohfip)_4_]^−^ anion (RI-B3LYP(D3BJ)/def2-TZVPP; ε_r_ = 7.23). Note that a very small negative HOMO/LUMO gap is found for the [Mg(G2)_2_]^⋅+^ radical cation (0.01 eV).

**Scheme S2**: Calculated reaction enthalpies Δ_r_H° and free reaction enthalpies Δ_r_G° in the gas phase and in a G2 solution for a reaction path describing the decomposition of the [Al(Ohfip)_4_]^−^ anion (RI-B3LYP(D3BJ)/def2-TZVPP; ε_r_ = 7.23).

**Scheme S3**: Calculated reaction enthalpies Δ_r_H° and free reaction enthalpies Δ_r_G° in the gas phase and in a G2 solution. The reaction equations are designed to compare the stability of different complexes in a G2/MOEA solution of Mg[O_3_SCF_3_]_2_ (RI-B3LYP(D3BJ)/def2-TZVPP; ε_r_ = 7.23).

**Scheme S4**: Calculated reaction enthalpies Δ_r_H° and free reaction enthalpies Δ_r_G° in the gas phase and in a G2 solution. The reaction equations are designed to compare the stability of different complexes in a G2/MOEA solution of Mg[O_3_SCF_3_]_2_ (RI-B3LYP(D3BJ)/def2-TZVPP; ε_r_ = 7.23).

*2.2 Löwdin Spin Population Analysis for [Mg(G2)_2_]^⋅+^ and [Mg(MOEA)_3_]^⋅+^*

The Löwdin analysis for the [Mg(G2)_2_]^⋅+^ and [Mg(MOEA)_3_]^⋅+^ radical cations was also performed on the RI-B3LYP(D3BJ)/def2-TZVPP level of theory. The results are summarized in **Scheme S5** and **Table S5**.

**Scheme S5**. Investigation of the LUMO orbitals of the [Mg(MOEA)_3_]^2+^ and [Mg(G2)_2_]^2+^ ions and their first vertical 1 e^–^ reduction by DFT calculation (RI−B3LYP(D3BJ)/def2-TZVPP; isosurface values: 0.045 a.u.).

**Table S5**: Summary of the Löwdin population analysis for the [Mg(G2)_2_]^⋅+^ and [Mg(MOEA)_3_]^⋅+^ radical cations (RI-B3LYP(D3BJ)/def2-TZVPP; single point calculations were performed on the optimized structures of the dications, thus investigating the complex obtained upon vertical one-electron reduction, without relaxation).

|  | **Spin Population on Atom Types** | | | | | |
| --- | --- | --- | --- | --- | --- | --- |
| **Complex** | Mg atom | C atoms | O atoms | C**H** atoms | N atoms | N**H** atoms |
| [Mg(G2)_2_]^⋅+^ | 0.097 | 0.028–0.034 | 0.015–0.017 | 0.010–0.020 | - | - |
| [Mg(MOEA)_3_]^⋅+^ | 0.129 | 0.010–0.018 | 0.005–0.007 | 0.003–0.023 | 0.018–0.027 | 0.045–0.093 |

*2.3 DFT Calculations to investigate the role of Mg-hydroxide complexes in the two electrolyte systems.*

The analysis of the hydroxylation of the [Al(Ohfip)_4_]^−^ anion revealed that hydroxide ions are present in the electrolyte system. In the following we analyze the potential importance of those in the two electrolyte systems and also with respect to the observed surface etching of the Mg interface in [Al(Ohfip)_4_]^−^ electrolyte, but its absence in the triflate electrolyte. This first involved the question, as to whether solvated magnesium-hydroxide complexes are favorable in solution in the two distinctly different electrolyte environments, that we address separately.

**Situation in the Mg[Al(Ohfip)_4_]_2_/G2-MOEA system:**

**Scheme S6**: Calculated free reaction enthalpies Δ_r_G° in a G2 solution for the formation of solvated [Mg(OH)]^+^ and [Mg(OH)_2_]^0^ species, in the absence of the [TfO]^−^ anion (RI-B3LYP(D3BJ)/def2-‍‍TZVPP; ε_r_ = 7.23).

**Scheme S7**: Calculated reaction enthalpies Δ_r_H° and free reaction enthalpies Δ_r_G° in the gas phase and in a G2 solution, describing different plausible equilibria in a solution of Mg[Al(Ohfip)_4_]_2_ (RI-B3LYP(D3BJ)/def2-TZVPP; ε_r_ = 7.23).

**Situation in the Mg[TfO]_2_/G2-MOEA system:**

**Scheme S8**: Calculated reaction enthalpies Δ_r_H° and free reaction enthalpies Δ_r_G° in the gas phase and in a G2 solution, describing different plausible equilibria in a solution of Mg[TfO]_2_ (RI-B3LYP(D3BJ)/def2-TZVPP; ε_r_ = 7.23)

**§3 Solubility and basic electrochemistry**

3-1. *Salt solubility.*

Amines are recognized as strongly solvating solvents owing to their relatively high Gutmann donor numbers (*DNs*). The *DN* of a representative amine, triethylamine, is 61.0—approximately three times higher than those of conventional ethers, which typically range from 18 to 20.^[31,32]^ Despite the large *DNs* of amines, the solubility of Mg salts in alkylamines remains limited, as illustrated in Figures S1 and S2. Even Mg salts containing weakly-coordinated anions (WCAs) are insoluble in ternary amines but dissolve in secondary amines, indicating a significant contribution of hydrogen bonding for nitrogen-based solvation to the stabilization of ionic species. Mismatch against the Pearson’s hardness/softness concept also contributes to the rather low solubility of Mg salts especially in tertiary amine solvents, where bond energies between the alkylated nitrogen (soft base) and Mg^2+^ (hard acid) are relatively low and provide not enough driving force for solubilization. Moreover, the inherently low dielectric nature of amines contributes to the limited solubilization they offer for salts; the dielectric constants of typical alkylamines are below 4, approaching those of carbon disulfide and toluene.^[32]^ Hence, the low dielectric constant of soft alkylamines prevents effective stabilization of isolated ionic species. In contrast, glymes exhibit higher permittivities than amines.^[32]^ The hard base nature of ether oxygens, based on Pearson’s concept, also help to explain the marked stabilization of Mg^2+^ in solutions. Functionalization of amines with such ether oxygen atoms significantly enhances the solubility of Mg salts owing to the improved dielectric characteristics and additional chelating effects provided by polyether oxygen donors. Consequently, coupling multidentate glymes with strongly solvating alkoxyalkylamines possessing both, hydrogen substituted nitrogen-, as well as oxygen-donors, is an effective strategy to substantially increase the solubility of the highly associated Mg[TfO]_2_ salt by establishing a balanced solvation–dissociation equilibrium in solution.

**Figure S1**. Chemical structures of the screened amine molecules.

**Figure S2**. Digital photographs of the amine-Mg salt mixtures.

3-2. *Basic electrochemistry*

Figure S3 summarizes the CV profiles of Pt and Mg working electrodes recorded in single G2, single MOEA, and G2–MOEA (1:1 v/v) dual solvent mixtures using Mg[Al(Ohfip)_4_]_2_ and Mg[TfO]_2_ as conductive salts. All measurements were performed in a three-electrode configuration, employing magnesium metal and Ag^+^/Ag as the counter and reference electrodes, respectively. The electrode potential of the Ag^+^/Ag reference was calibrated using typical Grignard reagent–based (C_2_H_5_MgCl/THF) and Mg[Z(Ohfip)_4_]_2_/G2 (Z = B or Al) electrolytes.^[7,33]^

For electrolyte solutions containing the dissociative Mg[Al(Ohfip)_4_]_2_ salt, they exhibited favorable Mg plating/stirpping performance irrespective of the solvents. Incorporation of MOEA is found to be particularly effective in minimizing the overpotential during the Mg stripping process. In the case of Mg[TfO]_2_, their electrochemical Mg plating/stripping performance was markedly improved using G2-MOEA mixture. Although the current density is smaller than that of the corresponding Mg[Al(Ohfip)_4_]_2_-based system, possibly because of its lower ionic conductivity (Figure S12), the electrochemical response of the Mg electrodes exhibited a linear current–potential relationship throughout the scanning range. This behavior indicates that no significant activation barrier is required to initiate the Mg plating/stripping reactions and that Mg^2+^ ions diffuse smoothly under the present experimental conditions.

It should be mentioned that the Mg[TfO]_2_/G2-MOEA system displayed non-trivial behavior in which the potentials corresponding to Mg plating and stripping differed between the working electrodes. Subsequent visual and microscopic observations confirmed that the reversible responses at both electrodes can be attributed to genuine Mg plating/stripping reactions. Although the underlying origin of this phenomenon remains unclear, the unique interfacial characteristics of amines (vide infra) and electrode-dependent characteristics for hydrogen evolution reactions may contribute to such anomalous behavior. Elucidating the detailed mechanism responsible for the observed potential shift lies beyond the scope of the present study.

**Figure S3**. CV profiles of Pt and Mg electrodes recorded in (a, d) single G2-, (b, e) single MOEA-, and (c, f) dual G2–MOEA-based electrolyte solutions at a scan rate of 10 mV s^−1^, using Ag^+^/Ag as an external reference. The potentials vs. Ag^+^/Ag with only G2 are by about 400 mV less negative than those using the MOEA solvent. The conductive Mg salts employed were (a–c) Mg[Al(Ohfip)_4_]_2_ and (d–f) Mg[TfO]_2_. Due to the extremely low solubility of Mg[TfO]_2_ in G2, as shown in (d), the corresponding CV profile could not be obtained. The profiles of the initial cycles are presented to highlight the initial plating/stripping overpotential. Note that for the parallel CV measurements, various unidentified factors including the surface state of electrodes and spatial arrangement of each electrode can affect the observed current density.

In the electrolyte solutions, G2 plays the specific role not only offering good ionic conductivity but also constructing favorable interface. The galvanostatic cycling profiles using the 1:9 (v/v) G2:MOEA mixture with the Mg[TfO]_2_ salt are obviously inferior to those using the 1:1 (v/v) mixture (Figure S4). According to this brief compositional study, the 1:1 (v/v) mixture would be selected as the benchmarking solvent composition in this study.

**Figure S4**. Galvanostatic cycling profiles of (a, c) [Mg || Cu] and (b, d) [Mg || Mg] cells using (a, b) Mg[TfO]_2_/G2-MOEA (1:9 v/v) and (c, d) Mg[TfO]_2_/G2-MOEA (1:1 v/v) measured at a current density of 1 mA cm^−2^ and 30 °C.

**§4 Supporting Figures**

**Figure S5**. Galvanostatic Mg plating/stripping cycling profiles of (a) asymmetric [Mg || Cu] and (b) symmetric [Mg || Mg] cells recorded in Mg[B(Ohfip)_4_]_2_/G2-MOEA at a current density of 1 mA cm^−2^ at 30 °C. (c) Nyquist plot of the [Mg || Mg] cell before the cycling measurements.

**Figure S6**. Galvanostatic cycling-hold measurements of symmetric [Mg || Mg] cells recorded in (a) Mg[Al(Ohfip)_4_]_2_/G2-MOEA and (b) Mg[TfO]_2_/G2-MOEA at a current density of 1 mA cm^−2^ at 30 °C. Arrows in the figures indicate the rest period (3 hour). The voltage fluctuations observed in (a) are indicative of occurrence of successive micro-short-circuiting events.

**Figure S7**. SEM images of Mg deposits obtained by galvanostatic deposition at 1 mA cm^−2^ for 2 h from (a, b) Mg[Al(Ohfip)_4_]_2_/G2-MOEA and (c, d) Mg[TfO]_2_/G2-MOEA electrolytes. Panels (a, c) show bird’s-eye views, and (b, d) present the corresponding magnified images. (e) pXRD profiles of the same deposits on the carbon-coated Al (Al/C) substrate. Simulated profiles of Mg as well as the experimental profile of Al/C are included as references.

**Figure S8**. Snapshots of the corresponding ECCS measurements (Videos 1 and 2).

**Figure S9**. Galvanostatic Mg plating/stripping cycling profiles of asymmetric [Mg || Cu] cells using pristine Mg electrodes and Mg electrodes precycled in Mg[TfO]_2_/G2-MOEA. An ethereal electrolyte of Mg[Al(Ohfip)_4_]_2_/G2 was used for the measurements.

**Figure S10**. ^1^H NMR (400.17 or 300.18 MHz) spectra of Mg[Al(Ohfip)_4_]_2_/G2-MOEA and Mg[TfO]_2_/G2-MOEA electrolytes measured at 25 °C. The corresponding spectra of the individual solvents and the dual-solvent mixture are included as references. Asterisks (*) indicate the protons of G1, which acts as a crystal solvent in the Mg[Al(Ohfip)_4_]_2_ salt (three molar equivalents).

**Figure S11**. ^1^H NMR spectra (400.17 or 300.18 MHz) of MOEA, Mg[Al(Ohfip)_4_]_2_/MOEA, and Mg[TfO]_2_/MOEA at 298 K.

**Figure S12**. (a) Ionic conductivities and viscosities and (b) Walden plots of the G2 and G2-MOEA solutions of Mg[Al(Ohfip)_4_]_2_ and Mg[TfO]_2_ measured at 30 °C.

**Figure S13**. Raman spectrum showing the δ_s_(CF_3_) modes of [TfO]^−^ for Mg[TfO]_2_/G2-MOEA. A spectral deconvolution was performed using the Voigt function. Relative peak areas are included in the figure.

**Figure S14**. SEM images of Mg electrodes soaked in Mg[Al(Ohfip)_4_]_2_/G2 for 48 h at 30 °C.

**Figure S15**. ^1^H, ^19^F, and ^27^Al NMR spectra (either 400.17, 376.4 and 104.27 MHz or 300.18, 282.45, and 78.22 MHz, respectively) of the as-prepared Mg[Al(Ohfip)_4_]_2_/G2-MOEA and that containing ca. 0.5 mol dm^−3^ of water at 298 K. The spectra shown here were not calibrated with TMS. An additional small peak at about −76.5 ppm in the ^19^F spectrum of the electrolyte containing excess water is assignable to the signal from the reaction products. The intensive broad resonance between 0 and 100 ppm in the ^27^Al-NMR spectrum is a background signal from the probehead, whereas the small broad shoulder at 30 ppm is a signal from the sample.

**Figure S16**. ^1^H, ^19^F, and ^27^Al NMR spectra (400.17, 376.4 and 104.27 MHz) of the as-prepared and aged Mg[Al(Ohfip)_4_]_2_/G2-MOEA at 298 K. Certain spectral ranges relevant for [Al(Ohfip)_4_]^−^ are shown. The intensive broad resonance between 0 and 100 ppm in the ^27^Al-NMR spectrum is a background signal from the probehead, whereas the small broad shoulder at 30 ppm is a signal from the sample.

**Figure S17**. FT-ATR-IR spectra of Mg[Al(Ohfip)_4_]_2_/G2-MOEA and Mg[TfO]_2_/G2-MOEA showing the specific spectral range relevant to N-H, O-H, and C-H stretching vibration. (a) 3600–2600 cm^−1^ and (b) 3500–3100 cm^−1^ with spectral deconvolution by Voigt functions (magnified region marked in (a)). The spectra of G2-MOEA are also included as a reference. The redshift of N-H vibrational modes (at 3383 and 3310 cm^−1^ in the blank G2-MOEA solution) in the electrolytes suggests H-bonding. The shoulder peak at around 3308 cm^−1^ for Mg[Al(Ohfip)_4_]_2_/G2-MOEA is attributable to O-H stretching of the reaction products.

**Figure S18**. ^19^F NMR spectra (376.54 or 282.45 MHz) of Mg[TfO]_2_/G2-MOEA and Mg[TfO]_2_/MOEA at 298 K.

**Figure S19**. Mg 2p, N 1s, O 1s, and F 1s (left to right) XPS spectra of Mg electrodes cycled in (upper) Mg[Al(Ohfip)_4_]_2_/G2-MOEA and (lower) Mg[TfO]_2_/G2-MOEA. After electrochemical Mg plating/stripping cycling, the nitrogen and fluorine species can be detected on the Mg surface cycled in Mg[Al(Ohfip)_4_]_2_/G2-MOEA.

**Figure S20**. Galvanostatic Mg plating/stripping cycling profiles of [Mg || Mg] symmetric cells recorded in Mg[Al(Ohfip)_4_]_2_/G2-MOEA at a current density of (a) 0.1, (b) 0.5, and (c) 1 mA cm^−2^ at 30 °C.

**Figure S21**. ^1^H, ^11^B, and ^19^F NMR spectra (either 400.17, 128.4, and 376.4 MHz, or 300.18, 96.31, and 282.45 MHz, respectively) of Mg[B(Ohfip)_4_]_2_/G2-MOEA as prepared and aged for 3days measured at 298 K. The spectra of Mg[B(Ohfip)_4_]_2_/G1 are also included as a reference. The asterisks shown in the spectra are assignable to impurities, such as Mg(Ohfip)_2_ and B(Ohfip)_3_, in the parent salt. The substantial upfield shift of the ^19^F signal of Mg[B(Ohfip)_4_]_2_/G2-MOEA from that of Mg[B(Ohfip)_4_]_2_/G1 is due to the change in the solution environment. Observation of no new peak evolution upon mixing Mg[B(Ohfip)_4_]_2_ salt with the G2-MOEA solvent indicates sufficient stability of [B(Ohfip)_4_]^−^ against hydroxide ions. Broadening of the peaks over time however suggests gradual decomposition (hydrolysis) of the anion.

**Figure S22**. (a) LSV and (b) chronoamperometric profiles of Pt and Al working electrodes, respectively, recorded in Mg[Al(Ohfip)_4_]_2_/G2-MOEA and Mg[TfO]_2_/G2-MOEA electrolytes. An LSV profile of Pt working electrode recorded in the MOEA-free Mg[Al(Ohfip)4]2/G2 electrolyte was included as a reference in (a). (c)-(f) SEM images of Al current collector after corresponding chronoamperometry measurements polarized at (c,e) 2.0 V and (d,f) at 2.5 V in (c,d) Mg[Al(Ohfip)_4_]_2_/G2-MOEA and (e,f) Mg[TfO]_2_/G2-MOEA.

**References**

[1]	T. Mandai, Y. Youn, Y. Tateyama, *Mater. Adv.* **2021**, *2*, 6283.

[2]	T. Mandai, *Batteries & Supercaps* **2025**, *8*, e202500348.

[3]	T. Mandai, *ACS Appl. Mater. Interfaces* **2020**, *12*, 39135.

[4]	E. Levi, Y. Gofer, Y. Vestfreed, E. Lancry, D. Aurbach, *Chem. Mater.* **2002**, *14*, 2767.

[5]	D. Setiawan, H. J. Kim, J. Lyoo, S.-T. Hong, M. S. Chae, *Chem. Eng. J.* **2023**, *474*, 145596.

[6]	Hydranal^TM^ Laboratory Report L288, *Water determination in nitrogen bases*, http://lab.solstice.com/en/resources/hydranal-lab-reports/water-determination-in-nitrogen-bases. Accessed on 3^rd^ of June, 2026.

[7]	T. Mandai, Y. Akita, S. Yagi, M. Egashira, H. Munakata, K. Kanamura, *J. Mater. Chem. A* **2017**, *5*, 3152.

[8]	J. P. Perdew, *Phys. Rev. B* **1986**, *33*, 8822.

[9]	J. P. Perdew, *Phys. Rev. B* **1986**, *34*, 7406.

[10]	A. D. Becke, *Phys. Rev. A* **1988**, *38*, 3098.

[11]	R. Ahlrichs, M. Bär, M. Häser, H. Horn, C. Kölmel, *Chem. Phys. Lett.* **1989**, *162*, 165.

[12]	A. Schäfer, H. Horn, R. Ahlrichs, *J. Chem. Phys.* **1992**, *97*, 2571.

[13]	K. Eichkorn, O. Treutler, H. Öhm, M. Häser, R. Ahlrichs, *Chem. Phys. Lett.* **1995**, *242*, 652.

[14]	M. Von Arnim, R. Ahlrichs, *J. Comput. Chem.* **1998**, *19*, 1746.

[15]	P. Deglmann, F. Furche, *J. Chem. Phys.* **2002**, *117*, 9535.

[16]	O. Treutler, R. Ahlrichs, *J. Chem. Phys.* **1995**, *102*, 346.

[17]	P. Deglmann, F. Furche, R. Ahlrichs, *Chem. Phys. Lett.* **2002**, *362*, 511.

[18]	F. Neese, *WIREs Comput. Mol. Sci.* **2012**, *2*, 73.

[19]	F. Neese, *WIREs Comput. Mol. Sci.* **2022**, *12*, e1606.

[20]	M. Sierka, A. Hogekamp, R. Ahlrichs, *J. Chem. Phys.* **2003**, *118*, 9136.

[21]	R. Ahlrichs, *Phys. Chem. Chem. Phys.* **2004**, *6*, 5119.

[22]	F. Weigend, *Phys. Chem. Chem. Phys.* **2006**, *8*, 1057.

[23]	S. Grimme, J. Antony, S. Ehrlich, H. Krieg, *J. Chem. Phys.* **2010**, *132*, 154104.

[24]	S. Grimme, S. Ehrlich, L. Goerigk, *J. Comput. Chem.* **2011**, *32*, 1456.

[25]	C. Lee, W. Yang, R. G. Parr, *Phys. Rev. B* **1988**, *37*, 785.

[26]	A. D. Becke, *J. Chem. Phys.* **1993**, *98*, 1372.

[27]	A. D. Becke, *J. Chem. Phys.* **1993**, *98*, 5648.

[28]	F. Weigend, R. Ahlrichs, *Phys. Chem. Chem. Phys.* **2005**, *7*, 3297.

[29]	A. Klamt, G. Schüürmann, *J. Chem. Soc., Perkin Trans. 2* **1993**, 799.

[30]	K. Hoshi, T. Chiba, J. Sato, Y. Hayashi, Y. Takahashi, H. Ebe, S. Ohisa, J. Kido, *ACS Appl. Mater. Interfaces* **2018**, *10*, 24607.

[31]	P. Zhou, Y. Xiang, K. Liu, *Energy Environ. Sci.* **2024**, *17*, 8057.

[32]	Y. Marcus, *Chem. Soc. Rev.* **1993**, *22*, 409.

[33]	T. Mandai, *ChemSusChem* **2025**, *18*, e202500418.
