# Supplementary material for: Unsuccessful Coupling of Weakly Coordinating Anion–Based Mg Salts and Alkoxyalkylamine Solvents for Rechargeable Mg Battery Application: Trace Water‐Driven Interfacial Failure
Source: Adv Sci (Weinh). 2026 Jul 29:e76897. Online ahead of print. doi: 10.1002/advs.76897 (PMC13418276; doi:10.1002/advs.76897)
Supplement: Supplementary file 1 — Supporting File 1: advs76897‐sup‐0001‐SuppMat.docx. [file ADVS-9999-e76897-s001.docx]

*Supporting Information for:*

Unsuccessful Coupling of Weakly-Coordinating Anion-Based Mg salts and Alkoxyalkylamine Solvents for Rechargeable Mg Battery Applications: Trace Water-Driven Interfacial Failure

Toshihiko Mandai^1,^*, Antoine Barthélemy,^2^ Hendrik Koger,^2^ Harald Scherer,^2^ and Ingo Krossing^2^

^1^Research Center for Energy and Environmental Materials (GREEN), National Institute for Materials Science (NIMS), 1-1 Namiki, Tsukuba, Ibaraki 305-0044, Japan.

^2^Institute for Inorganic Chemistry and Analytic Chemistry as well as Freiburg Materials Research Center FMF, University of Freiburg, Albertstr. 21, 79104 Freiburg, Germany

Corresponding author footnote

TEL: +81-29-860-4464

Eail: MANDAI.Toshihiko@nims.go.jp

**§1 Materials and experimental methods**

- 1. *Materials.*

2-Methoxyethylamine (MOEA), 3-methoxypropylamine (MOPA), *N*-methyl *N*-propylamine (MPA), *N*,*N*-dimethyl propylamine (DMPA), magnesium triflate (Mg[TfO]_2_), trimethylaluminum (Al(CH_3_)_3_; 15% in hexane, *ca*. 1.4 mol dm^−3^), dimethyl sulfide borane (BH_3_∙S(CH_3_)_2_; >90%) were purchased from Tokyo Chemical Industry Co., Ltd. 1,1,1,3,3,3-Hexafluoro-iso-propanol (HOhfip), anhydrous diethyl ether, and anhydrous pentane were obtained from FUJIFILM Wako Chemicals. Di-*n*-butylmagnesium (Mg(*n*-Bu)_2_; 0.5 mol dm^−3^ in heptane) was purchased from Thermo Scientific Chemicals. Monoethylene glycol diethyl ether (G1) and diethylene glycol dimethyl ether (G2) for electrochemistry were obtained from Kanto Chemical Co., Inc. A series of amine-based solvents were dehydrated by distillation over CaH_2_ (Merck) and stored under an Ar-filled glovebox (< 1 ppm H_2_O and O_2_) with activated molecular sieves 4A. HOhfip was purified by vacuum distillation over activated molecular sieves 4A. Mg[TfO]_2_ was dried under vacuum heating at 200 °C for 1 week and stored in the glovebox prior to use. The other chemicals listed above were used as received. Mg[Al(Ohfip)_4_]_2_ was synthesized according to the reported procedures using Mg(*n*-Bu)_2_ or Mg(OCH_3_)_2_, Al(CH_3_)_3_, and HOhfip as reagents and pentane, diethyl ether, and G1 as reaction solvents.^[1,2]^ Mg[B(Ohfip)_4_]_2_ was synthesized through the modified transmetallation reactions between individually obtained B(Ohfip)_3_ and Mg(Ohfip)_2_ in G1.^[3]^ The chemical structures of Mg[Z(Ohfip)_4_]_2_ (Z = Al or B) were identified as adducts of three G1 solvents by ^1^H, ^11^B, ^19^F, and ^27^Al NMR spectroscopy, in consistence with the reported ones. Cu_2_Mo_6_S_8_ was obtained from Kojundo Chemical Laboratory Co. Ltd. Cu was leached out from Cu_2_Mo_6_S_8_ by a standard oxidation protocol to activate Mg^2+^ storage capability.^[4]^ FeV_3_O_9_∙1.1H_2_O (FeVO) was synthesized via an ambient solution process using NH_4_VO_3_ (99%, Sigma Aldrich) and Fe(NO_3_)_3_∙9H_2_O (98%, Sigma-Aldrich) as reagents, according to the literature reported elsewhere.^[5]^

The single amine or G2 solutions were prepared by dissolving the predetermined amounts of conductive salts (Mg[Al(Ohfip)_4_]_2_ and Mg(TfO)_2_) in the solvents, followed by vigorous stirring at 30 °C overnight in an Ar-filled glovebox. For the G2-MOEA dual solvent systems, the conductive salts were dissolved in the premixed solvents (G2:MOEA = 1:1 by vol.), followed by vigorous stirring at 30 °C overnight to ensure complete dissolution of the salts. The Mg salt concentration in solutions was fixed at 0.5 mol dm^−3^ for Mg[TfO]_2_, and 0.3 mol dm^−3^ for Mg[Al(Ohfip)_4_]_2_, while that for the single solvent systems of Mg[TfO]_2_ was less than 0.1 mol dm^−3^ (saturated) due to the limited solubility of the salt. As the water content of the amine-containing electrolytes could not be determined precisely by conventional coulometric Karl Fischer (KF) titration due to the direct reactivity of amines with the KF reagents, we adopted the designated titration protocol with the presence of benzoic acid. The details are given in the next section.

- 1. *Determination of water contents by coulometric KF Titration for MOEA-solvent*

The water determination was carried out with standard procedures using a SI Analytics TitroLine® 7500 KF trace coulometric Karl-Fischer titrator. As the generator electrode, a TZ 1753 electrode with diaphragm and a KF1150 were used as the detector and a micro double Pt electrode, respectively. For the anolyte and catholyte, Hydranal™ Coulomat AG and Hydranal™ Coulomat CG, respectively, were used.

The titration with this configuration gave a value of 165.8 ppm of water in the MOEA solvent, however the corresponding titration curve did not show a definite endpoint, but ended due to set time parameters of 600 s, indicating issues due to unwanted reactivity of the basic MOEA-solvent with the reagents or incomplete titration of the trace water. To improve the experimental accuracy, we followed the protocols of the Hydranal™ Center of Excellence given in Laboratory Report L288.^[6]^ Following the suggestion for MOPA, that structurally analogous to MOEA, in this setup, we performed multiple titrations resulting in 113.7–114.4 ppm with standard settings (*t*_min_ = 60 s, *t*_max_ = 600 s, 300 mV working voltage, ΔDrift = 0.2 µg min^−1^, drift tolerance at endpoint = 0.02 µg min^−1^) and 156.0 ppm with narrowed convergence criteria (*t*_min_ = 60 s, *t*_max_ = 1800 s, 300 mV working voltage, ΔDrift = 0.1 µg min^−1^, drift tolerance at endpoint = 0.02 µg min^−1^), and took the latter as the relatively reliable value. It should be emphasized that all observed titration curves were still not following ideal saturation curves due to the even high reactivity of neutralized amines with KF reagents although changing experimental parameters and adding the benzoic acid had a positive influence on the titration curve. An upswing of the displayed value of the Hydranal™ 1% water-standards from 1000.8 ppm to 1113–1138 ppm by adding MOEA further supports signaling issues with the MOEA-addition and incompatibility of MOEA-solvent with coulometric KF titration. Nevertheless, negligible reaction of the MOEA-based electrolytes with moisture-sensitive chemicals, such as CaH_2_, Mg(*n*-Bu)_2_, and Al(CH_3_)_3_, suggests sufficient dryness of solvents and salts by the proposed drying protocol.

- 1. *Electrochemistry*

The electrochemical Mg plating/stripping property was assessed preliminary by cyclic voltammetry (CV) with three-electrode configuration using Ag^+^/Ag couple as an external reference. Pt (φ = 3 mm; BAS) and Mg (φ = 2 mm; Fujifilm Wako Co., Ltd.) disks served as the respective working electrodes while a Mg strip was used as a counter electrode. An Ag^+^/Ag reference was prepared according to the reported procedure.^[7]^ The CV profiles were acquired using an electrochemical analyzer HSV-110 (Meiden Hokuto Corp.), and a fixed sweep rate of 10 mV s^−1^ was adopted for all CV measurements.

The long-term and rate capabilities of Mg plating/stripping were evaluated by galvanostatic cycling measurements on asymmetric and symmetric two-electrode cells (SB2A; EC Frontier Co., Ltd.). Coulombic efficiencies for Mg plating/stripping reactions were calculated from the charges passed during stripping over plating of the conventional galvanostatic measurements with the asymmetric cells. The electrochemical impedance spectroscopy was performed on the symmetric two-electrode cells by scanning the current frequency from 1 MHz to 10 mHz with a sinusoidally alternating voltage amplitude of 50 mV root-mean-square using a potentiostat/galvanostat (VMP3, BioLogic). Asymmetric cells were fabricated using Cu and Mg foils as working and counter electrodes, respectively. The surface of Mg electrodes (99.94%, *t* = 0.04 mm; Rikazai) was polished mechanically with SiC sandpaper (#400, #2000, and #5000), washed several times with anhydrous G1, and dried under vacuum at ambient temperature for 1 h, irrespective of the cell assemblies. The porous glass filter (GF/A; *t* = 0.26 mm; Whatman) served as a separator. The potentiostatic measurements (chronoamperometry) were conducted to characterize the corrosion stability of Al current collectors in the electrolytes. The two-electrode cells were polarized at predetermined voltages (2.0 and 2.5 V vs. Mg) for 1h each, then corresponding current profiles were monitored. Galvanostatic discharge-charge cycling measurements were conducted to assess the compatibility of the electrolytes with prospective positive electrode materials. Composite positive electrodes were fabricated by mixing active material powder, carbon nanotube/fluoropolymer dispersion (VTD-475N; Daikin Industries, Ltd.), acetylene black (AB), and polyvinylidene difluoride (PVdF) in a weight ratio of 92:0.5:3.5:4 in anhydrous *N*-methylpyrrolidone (FUJIFILM Wako Chemicals) to obtain a slurry. Then, the slurry was spread onto a carbon-coated Al foil current collector and dried at 120 °C for several days. The resultant composite sheet was compressed, cut into circular shape (φ = 16 mm, corresponding to 2.01 cm^2^), and served as working electrodes. The average loading was fixed at *ca*. 4 and 1.5 mg cm^−2^ for Mo_6_S_8_ and FeVO, respectively. Two-electrode cells comprising the composite electrode (φ = 16 mm), Mg negative electrode (φ = 16 mm, *t* = 40 μm), porous glass filter separator (GF/A; φ = 17 mm), and the electrolytes (100 μL) were assembled in a glovebox. The cycling test was conducted within a voltage range of 0.3–1.8 V with a current density of 12.2 mA g^−1^ for Mo_6_S_8_ and a range of 0.8–3.3 V with a current density of 10 mA g^−1^ for FeVO. For [Mg || FeVO] cells, the capacity was limited to 200 mAh g^−1^ to avoid successive electrolyte decomposition. Galvanostatic and potentiostatic measurements were conducted using an automatic charge-discharge instrument (HJ0610SD8C, Meiden-Hokuto Co., Ltd.).

All electrochemical cell assemblies were carried out in an Ar-filled glove-box. The electrochemical measurements were conducted at 30 °C unless otherwise specified, and the same measurements were conducted at least three times to ensure the experimental reproducibility.

- 1. *Chemical/physical analysis*

The structure, surface chemical nature, and morphological aspects of the deposits, cycled Mg electrodes, and polarized current collectors were characterized using X-ray diffraction (XRD; SmartLab, Rigaku), X-ray photoelectron spectroscopy (XPS; VersaProbe II, ULVAC-PHI), Time-of-Flight Secondary Ion Mass Spectrometry (TOF-SIMS; TOF.SIMS5-AD-GCIB, ION-TOF GmbH), scanning and transmission electron microscopy (SEM, JSM-7800F, JEOL; STEM, EM-ARM200F, JEOL) combined with energy-dispersive X-ray spectrometry (EDX) and electron energy loss spectroscopy (EELS). XRD profiles were acquired using Cu Kα radiation (λ = 1.54078 Å). XPS measurements were conducted with an Al Kα X-ray source under a base pressure of less than 6.7 × 10^−8^ Pa. The binding energy of the obtained spectra was calibrated using the C 1s peak of *sp*2-hybridized carbon at 284.5 eV as a reference. A primary ion gun of 30 kV Bi^3+^ and a sputter rate of 4 nm min^−1^ as SiO_2_ film were adopted for acquiring the depth profiles for TOF-SIMS measurements. The surface of the deposited Mg, cycled Mg electrodes, and polarized current collectors was observed using SEM and TEM, and the distribution of elements was subsequently characterized using EDX and EELS. To visualize the dynamic Mg plating/stripping behavior in the different electrolytes, confocal microscopic analysis was performed using the electrochemical confocal system (ECCS B320-N, Lasertec). To avoid any exposure to the air, the samples were fabricated and placed in a designated airtight chamber in an Ar-filled glovebox and transferred for each analysis or measurement.

- 1. *Solution states and characteristics*

The coordination states of solvents and anions in solutions were elucidated by ^1^H, ^19^F, and ^27^Al nuclear magnetic resonance (NMR), Raman spectroscopy, and Fourier-transform attenuated total reflection infrared (FT-ATR-IR) spectroscopy. All NMR spectra were recorded either using Bruker Avance IV Neo 400 MHz or Bruker Avance III HD 300 MHz NMR spectrometers. The samples were placed in the designated NMR tubes in the glove box, sealed by a typical flame sealing method or with a J. Young valve, and transferred to the spectrometer. The chemical shifts were calibrated using tetramethylsilane as a standard. Field correction for hetero nuclei was calculated according to the calibrated ^1^H NMR sprectra using the IUPAC Ξ-table. The Raman spectra were collected at ambient temperature (22±2 °C) using a laser Raman spectrometer (NRS-4500, JASCO) equipped with a 785 nm laser at a spectral resolution of 1 cm^−1^. The sample solutions were hermetically sealed in quartz glass tubes in an Ar-filled glovebox and were subjected to spectrometry without any exposure to moisture. The acquired spectra were calibrated using a Si standard. The spectral deconvolution was carried out by the Voigt function. For FT-ATR-IR measurements, the spectra were recorded in an Ar-filled Glovebox using a Bruker Alpha I spectrometer equipped with a diamond ATR unit. The liquids were contacted with the stamp of the ATR unit. The spectral range of 4000–400 cm^−2^ with a resolution of 2 cm^−2^ was adopted and 64 background scans and 128 sample scans were performed with each sample. The obtained spectra were evaluated using Bruker OPUS 7.5 software to produce three iterations of a baseline correction as well as a normalization of the spectra referring to the largest peak, respectively.

The ionic conductivities of the electrolyte solutions were measured using the complex impedance method with an impedance analyzer (VMP3, Biologic). A commercial cell (CT-57101B, TOA DKK Corporation) was used for the measurements. The cell was placed in a temperature-controlled chamber and held at 30 °C for 1 h to equilibrate the temperature prior to the measurements. The viscosities were measured at the same temperature using a kinematic viscometer (SVM3001, Anton Paar GmbH). The measurements were repeated at least three times for the different batches of the electrolytes prepared separately, and all of the standard deviations in the experimental values were within ±1% of the average.

**§2 Quantum chemical calculations**

*2-1. General considerations and calculation methods*

Quantum chemical calculations were performed with Turbomole (version 7.5.1),^[8–17]^ the Löwdin analysis was performed with the ORCA program package (version 5.0.0).^[18,19]^ Structures were optimized using density functional theory (DFT),^[16]^ internal coordinates, resolution of identity-approximation (RI),^[20–22]^ D3(BJ)-dispersion correction^[23,24]^ and a fine integration gridsize (gridsize = m5). Calculations were performed at the RI-B3LYP(D3BJ)/def2-TZVPP level of theory.^[25–28]^ Thermal and entropic contributions to the Gibbs energy were calculated without scaling factor at standard conditions (298.15 K, 0.1 MPa) with the FREEH module. Every species presented herein was checked in terms of reasonable geometry and electronic occupation with the EIGER module. Vibrational analyses were performed with the AOFORCE module, in order to detect imaginary frequencies.^[17]^

For each molecular species, the standard enthalpy *H*° at 298.15 K and 0.1 MPa was calculated from the electronic SCF energy *E*_SCF_ and the sum of translational, rotational, and vibrational energy including zero-point energy *E*_vrt_ (FREEH energy) using the following equation:

| *H*° = *E*_SCF_ + *E*_vrt_ + R ∙ *T* | equation 1 |
| --- | --- |

*E*_SCF_: electronic SCF energy

*E*_vrt_: sum of translational, rotational, and vibrational energy including zero-point energy

R: universal gas constant (ca. 8.314 J K^–1^ mol^–1^)

*T*: temperature in Kelvin (298.15 K)

The Gibbs free energy *G*° follows from the standard enthalpy *H*° and the standard entropy *S*°:

| *G*° = *H*° – *S*° ∙ *T* | equation 2 |
| --- | --- |

Solvation effects were incorporated using the conductor like screening model (COSMO)^[29]^ for G2 as the solvent. A dielectric constant of 7.23 at 298 K was assumed for this solvent.^[30]^ Single point calculations were performed on the optimized gas phase structures and the results from the vibrational analysis were taken from the respective gas phase calculations.

In the following section, the coordinates of the calculated species are given.

**[Al(Ohfip)_4_]^−^**


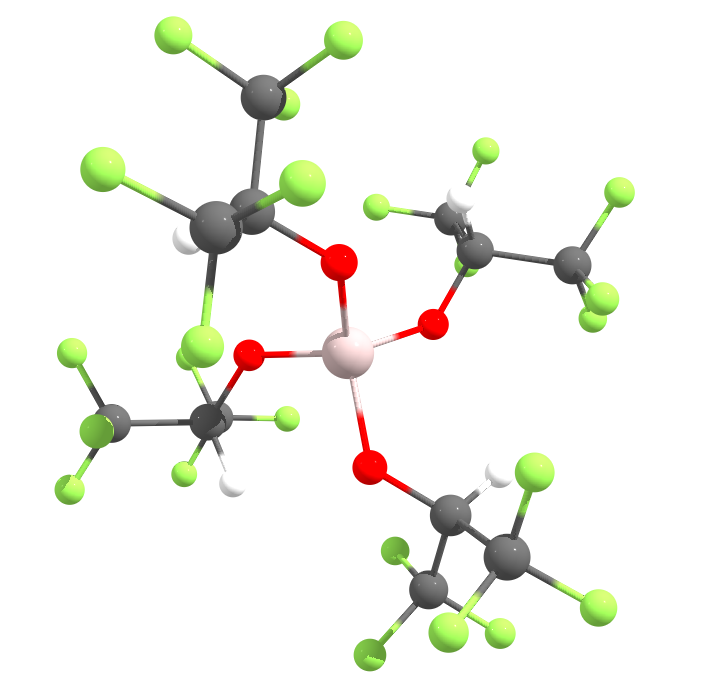


Method: (RI-)B3LYP(D3BJ)/def2-TZVPP

Symmetry: s4

Cartesian coordinates in Ångström:

Al 0.0000000 -0.0000000 0.0000000

O 0.6627544 1.3378470 -0.9132161

O 1.3378470 -0.6627544 0.9132161

O -1.3378470 0.6627544 0.9132161

O -0.6627544 -1.3378470 -0.9132161

C 2.5718666 -0.1154759 1.1213708

H 2.7401119 0.8171802 0.5665777

C -2.5718666 0.1154759 1.1213708

F -3.6218081 2.2787855 1.2541324

C -0.1154759 -2.5718666 -1.1213708

C 0.1154759 2.5718666 -1.1213708

F -1.7907263 -1.1302447 2.9625517

C -2.7411598 -0.2502539 2.6082154

F -3.9357272 -0.8339495 2.8550717

F -2.6371847 0.8090052 3.4267114

C -3.6504006 1.0937109 0.6208196

F -4.8984040 0.5962342 0.7517491

F -3.4576800 1.3378375 -0.6889177

H -2.7401119 -0.8171802 0.5665777

F 3.9357272 0.8339495 2.8550717

C 2.7411598 0.2502539 2.6082154

F 2.6371847 -0.8090052 3.4267114

F 1.7907263 1.1302447 2.9625517

F 3.6218081 -2.2787855 1.2541324

C 3.6504006 -1.0937109 0.6208196

F 3.4576800 -1.3378375 -0.6889177

F 4.8984040 -0.5962342 0.7517491

F 1.1302447 -1.7907263 -2.9625517

C 0.2502539 -2.7411598 -2.6082154

F 0.8339495 -3.9357272 -2.8550717

F -0.8090052 -2.6371847 -3.4267114

F -0.8339495 3.9357272 -2.8550717

C -0.2502539 2.7411598 -2.6082154

F 0.8090052 2.6371847 -3.4267114

F -1.1302447 1.7907263 -2.9625517

H -0.8171802 2.7401119 -0.5665777

F 1.3378375 3.4576800 0.6889177

C 1.0937109 3.6504006 -0.6208196

F 2.2787855 3.6218081 -1.2541324

F 0.5962342 4.8984040 -0.7517491

F -0.5962342 -4.8984040 -0.7517491

H 0.8171802 -2.7401119 -0.5665777

C -1.0937109 -3.6504006 -0.6208196

F -2.2787855 -3.6218081 -1.2541324

F -1.3378375 -3.4576800 0.6889177

SCF energy GEOOPT = -3399.862800190 H

ZPE = 542.0 kJ/mol

FREEH energy = 649.71 kJ/mol

FREEH entropy = 1.14846 kJ/mol/K

$vibrational spectrum

# mode symmetry wave number IR intensity selection rules

# cm**(-1) km/mol IR RAMAN

1 -0.00 0.00000 - -

2 -0.00 0.00000 - -

3 0.00 0.00000 - -

4 0.00 0.00000 - -

5 0.00 0.00000 - -

6 0.00 0.00000 - -

7 b 7.51 0.16453 YES YES

8 b 7.65 0.00000 YES YES

9 b 14.38 0.00000 YES YES

10 e 14.44 0.08610 YES YES

11 e 14.44 0.08610 YES YES

12 b 16.41 0.00428 YES YES

13 e 19.26 0.10503 YES YES

14 e 19.26 0.10503 YES YES

15 b 24.13 0.12888 YES YES

16 b 30.54 0.00180 YES YES

17 e 30.75 0.04367 YES YES

18 e 30.75 0.04367 YES YES

19 b 30.80 0.00000 YES YES

20 e 41.51 0.54074 YES YES

21 e 41.51 0.54074 YES YES

22 a 54.29 0.00000 NO YES

23 a 65.47 0.00000 NO YES

24 b 66.81 0.04849 YES YES

25 e 70.08 0.16901 YES YES

26 e 70.08 0.16901 YES YES

27 a 92.53 0.00000 NO YES

28 b 94.30 0.28436 YES YES

29 e 151.71 2.10095 YES YES

30 e 151.71 2.10095 YES YES

31 b 158.57 2.63877 YES YES

32 b 160.97 0.00000 YES YES

33 e 184.71 0.67592 YES YES

34 e 184.71 0.67592 YES YES

35 a 189.51 0.00000 NO YES

36 b 195.50 0.20066 YES YES

37 b 243.95 0.37723 YES YES

38 e 261.34 0.46628 YES YES

39 e 261.34 0.46628 YES YES

40 a 269.50 0.00000 NO YES

41 b 290.51 7.85091 YES YES

42 e 291.71 0.10472 YES YES

43 e 291.71 0.10472 YES YES

44 a 291.71 0.00000 NO YES

45 b 291.78 0.96842 YES YES

46 e 305.06 8.00062 YES YES

47 e 305.06 8.00062 YES YES

48 a 325.79 0.00000 NO YES

49 b 326.37 1.16348 YES YES

50 e 329.58 2.24818 YES YES

51 e 329.58 2.24818 YES YES

52 e 357.29 2.07266 YES YES

53 e 357.29 2.07265 YES YES

54 a 360.04 0.00000 NO YES

55 a 367.17 0.00000 NO YES

56 b 379.64 8.62148 YES YES

57 b 434.28 37.82844 YES YES

58 e 443.51 52.91815 YES YES

59 e 443.51 52.91815 YES YES

60 a 514.74 0.00000 NO YES

61 b 515.87 13.01870 YES YES

62 e 516.11 14.23975 YES YES

63 e 516.11 14.23974 YES YES

64 a 527.85 0.00000 NO YES

65 e 527.91 2.70232 YES YES

66 e 527.91 2.70232 YES YES

67 b 528.10 5.78915 YES YES

68 a 533.61 0.00000 NO YES

69 e 544.69 0.26541 YES YES

70 e 544.69 0.26541 YES YES

71 b 544.77 0.00000 YES YES

72 b 544.83 1.43937 YES YES

73 b 562.01 22.90461 YES YES

74 e 570.69 31.37741 YES YES

75 e 570.69 31.37741 YES YES

76 a 681.04 0.00000 NO YES

77 e 681.86 30.34061 YES YES

78 e 681.86 30.34060 YES YES

79 b 683.62 92.82511 YES YES

80 a 692.00 0.00000 NO YES

81 b 713.65 0.99438 YES YES

82 e 723.96 2.67510 YES YES

83 e 723.96 2.67510 YES YES

84 a 758.49 0.00000 NO YES

85 b 782.91 48.86856 YES YES

86 e 810.40 51.00934 YES YES

87 e 810.40 51.00931 YES YES

88 b 855.71 0.00000 YES YES

89 b 858.15 141.23018 YES YES

90 e 858.90 131.36215 YES YES

91 e 858.90 131.36191 YES YES

92 b 886.91 0.00000 YES YES

93 e 888.06 44.62793 YES YES

94 e 888.06 44.62818 YES YES

95 b 889.38 81.20599 YES YES

96 a 1084.71 0.00000 NO YES

97 e 1090.82 81.57266 YES YES

98 e 1090.82 81.57262 YES YES

99 b 1095.49 280.47177 YES YES

100 e 1120.22 11.47702 YES YES

101 e 1120.22 11.47701 YES YES

102 a 1120.76 0.00000 NO YES

103 b 1127.33 77.85848 YES YES

104 a 1171.35 0.00000 NO YES

105 e 1171.86 761.12476 YES YES

106 e 1171.86 761.12597 YES YES

107 b 1174.47 6.21728 YES YES

108 b 1189.37 55.34264 YES YES

109 e 1199.11 168.73073 YES YES

110 e 1199.11 168.72996 YES YES

111 a 1205.12 0.00000 NO YES

112 b 1206.61 618.63174 YES YES

113 e 1209.13 985.34184 YES YES

114 e 1209.13 985.34171 YES YES

115 a 1244.84 0.00000 NO YES

116 e 1254.58 127.36657 YES YES

117 e 1254.58 127.36611 YES YES

118 b 1256.41 0.00000 YES YES

119 b 1256.73 357.34236 YES YES

120 a 1289.58 0.00000 NO YES

121 e 1292.30 184.95766 YES YES

122 e 1292.30 184.95769 YES YES

123 b 1293.77 114.64324 YES YES

124 b 1382.56 0.00000 YES YES

125 e 1384.81 18.72553 YES YES

126 e 1384.81 18.72541 YES YES

127 b 1389.27 0.00000 YES YES

128 b 1389.99 8.57118 YES YES

129 e 1392.43 28.59256 YES YES

130 e 1392.43 28.59234 YES YES

131 b 1394.08 106.12606 YES YES

132 b 3001.68 0.67070 YES YES

133 e 3001.82 41.00677 YES YES

134 e 3001.82 41.00677 YES YES

135 b 3002.41 0.00000 YES YES

$end

Total COSMO energy + OC correction = -3399.8950269961 H

**[Al(Ohfip)_3_(MOEA^−H^)]^−^**


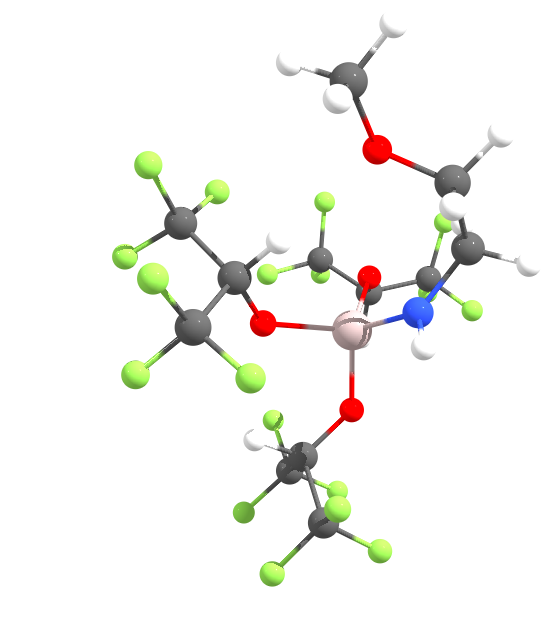


Method: (RI-)B3LYP(D3BJ)/def2-TZVPP

Symmetry: c1

Cartesian coordinates in Ångström:

F 4.7708093 -0.2376676 0.2950779

F 3.4570048 -0.1660689 -1.4264647

C 3.5112855 -0.5004311 -0.1242365

F 3.6917422 0.3033143 2.7199801

F 3.3379651 -1.8316719 -0.0478053

F 2.7345402 4.7466446 -0.3000753

H 2.7437527 1.3506235 0.5554761

C 2.4495338 0.2967162 0.6579235

C 2.4887959 0.0067632 2.1716076

F 0.9126234 4.9707939 -2.4171240

C 1.6378802 4.5695246 0.4573342

F 2.2240563 -1.2765374 2.4704639

F 1.9976471 3.8007715 1.5029407

F 1.2978513 5.7788041 0.9573412

O 0.8755442 2.6709618 -0.7426083

O 1.2144695 0.0158845 0.1608396

F 1.5755335 0.7641621 2.7984077

C -0.0260972 4.7486996 -1.4808577

C 0.4784871 3.8972490 -0.2995954

H -4.2370056 -2.1010962 -1.0451538

H 0.2019434 -1.5084452 -1.7742476

H -1.7411632 -2.8456392 -2.6179139

F -0.4899117 5.9565605 -1.0861567

Al 0.0296752 1.1327838 -0.5100280

F -1.0492877 4.1155274 -2.0843154

H -1.3091634 0.8720311 -2.5707263

C -3.3914328 -2.6140327 -0.5673845

O -2.1794046 -1.9461155 -0.8113971

C -1.8630077 -1.8381648 -2.1889371

H -3.5493122 -2.6238516 0.5098893

C -0.5814680 -1.0309489 -2.3667635

N -0.6861569 0.3508648 -1.9717351

H -0.3579338 3.8590731 0.4120391

H -3.3630685 -3.6485356 -0.9364053

H -0.2854057 -1.1458625 -3.4221900

H -2.6929425 -1.3464585 -2.7192926

O -1.2274061 1.6016193 0.6561498

F -1.5628082 -0.4076871 2.5012252

C -2.4296266 0.9672360 0.7916698

F -3.4270002 2.2958517 -0.8844349

H -2.5400347 0.0959872 0.1321920

C -2.5793022 0.4191356 2.2215635

F -2.5917294 1.3792246 3.1643411

C -3.5682817 1.9257917 0.4036922

F -3.5930658 3.0522627 1.1372858

F -3.7227917 -0.2980816 2.3735656

F -4.7909724 1.3530437 0.5107534

SCF energy GEOOPT = -2859.617261818 H

ZPE = 707.8 kJ/mol

FREEH energy = 808.01 kJ/mol

FREEH entropy = 1.06716 kJ/mol/K

$vibrational spectrum

# mode symmetry wave number IR intensity selection rules

# cm**(-1) km/mol IR RAMAN

1 -0.00 0.00000 - -

2 -0.00 0.00000 - -

3 0.00 0.00000 - -

4 0.00 0.00000 - -

5 0.00 0.00000 - -

6 0.00 0.00000 - -

7 a 9.68 0.01078 YES YES

8 a 11.98 0.14201 YES YES

9 a 14.32 0.39485 YES YES

10 a 16.91 0.20361 YES YES

11 a 18.43 0.02327 YES YES

12 a 21.55 0.14022 YES YES

13 a 24.32 0.22210 YES YES

14 a 29.42 0.05574 YES YES

15 a 30.46 0.04476 YES YES

16 a 31.74 0.01876 YES YES

17 a 34.75 0.50201 YES YES

18 a 41.13 0.20349 YES YES

19 a 48.24 0.45498 YES YES

20 a 60.06 0.36675 YES YES

21 a 66.76 0.04862 YES YES

22 a 69.53 0.13171 YES YES

23 a 73.17 0.26649 YES YES

24 a 78.36 0.47670 YES YES

25 a 89.26 0.16994 YES YES

26 a 97.34 4.13378 YES YES

27 a 124.73 0.63566 YES YES

28 a 148.19 1.44678 YES YES

29 a 157.87 0.76861 YES YES

30 a 161.33 1.88546 YES YES

31 a 176.00 1.13440 YES YES

32 a 186.14 0.75989 YES YES

33 a 191.17 0.20475 YES YES

34 a 206.48 0.19396 YES YES

35 a 233.83 2.32119 YES YES

36 a 239.64 3.76357 YES YES

37 a 257.61 0.92235 YES YES

38 a 264.84 1.91230 YES YES

39 a 275.69 2.92897 YES YES

40 a 286.87 8.15698 YES YES

41 a 290.79 0.09010 YES YES

42 a 291.54 0.12142 YES YES

43 a 291.61 0.10481 YES YES

44 a 310.50 9.16940 YES YES

45 a 322.47 2.01566 YES YES

46 a 325.81 1.15071 YES YES

47 a 329.22 7.45532 YES YES

48 a 331.33 1.13374 YES YES

49 a 339.73 11.96630 YES YES

50 a 353.39 1.45259 YES YES

51 a 358.48 2.50043 YES YES

52 a 373.73 0.71594 YES YES

53 a 396.38 34.92061 YES YES

54 a 399.90 45.46700 YES YES

55 a 437.74 59.84341 YES YES

56 a 449.53 45.71563 YES YES

57 a 515.02 3.15042 YES YES

58 a 515.91 13.22351 YES YES

59 a 516.86 14.02304 YES YES

60 a 525.82 3.99076 YES YES

61 a 526.76 2.21793 YES YES

62 a 527.33 5.49749 YES YES

63 a 527.55 6.05878 YES YES

64 a 543.85 0.33429 YES YES

65 a 543.92 0.39653 YES YES

66 a 544.17 0.36139 YES YES

67 a 548.61 19.41139 YES YES

68 a 560.93 35.51964 YES YES

69 a 568.42 37.89768 YES YES

70 a 666.30 22.23965 YES YES

71 a 680.66 31.26887 YES YES

72 a 680.89 4.65653 YES YES

73 a 682.35 75.84011 YES YES

74 a 706.58 1.85058 YES YES

75 a 717.41 2.55892 YES YES

76 a 725.56 23.01600 YES YES

77 a 763.10 34.88817 YES YES

78 a 775.47 73.31553 YES YES

79 a 787.50 66.91346 YES YES

80 a 845.00 30.34896 YES YES

81 a 854.31 22.80487 YES YES

82 a 855.44 146.24945 YES YES

83 a 856.68 130.43545 YES YES

84 a 884.45 39.09905 YES YES

85 a 885.73 21.95858 YES YES

86 a 887.68 71.94217 YES YES

87 a 926.77 25.84131 YES YES

88 a 1032.04 44.51815 YES YES

89 a 1076.62 4.27342 YES YES

90 a 1082.88 32.90410 YES YES

91 a 1087.11 109.08919 YES YES

92 a 1089.79 118.99295 YES YES

93 a 1106.32 60.88743 YES YES

94 a 1113.53 19.07965 YES YES

95 a 1118.20 75.26275 YES YES

96 a 1139.45 59.10045 YES YES

97 a 1159.57 87.34913 YES YES

98 a 1160.03 701.69359 YES YES

99 a 1163.69 367.54237 YES YES

100 a 1167.96 371.87781 YES YES

101 a 1180.88 6.94785 YES YES

102 a 1190.44 57.99495 YES YES

103 a 1198.08 480.48622 YES YES

104 a 1201.48 260.90197 YES YES

105 a 1203.78 144.03090 YES YES

106 a 1208.95 652.81218 YES YES

107 a 1210.28 256.30221 YES YES

108 a 1233.41 206.51168 YES YES

109 a 1250.15 18.49427 YES YES

110 a 1253.71 101.22409 YES YES

111 a 1254.65 180.18387 YES YES

112 a 1257.41 176.62678 YES YES

113 a 1288.30 15.45503 YES YES

114 a 1290.26 180.76658 YES YES

115 a 1291.62 144.73364 YES YES

116 a 1297.11 4.06795 YES YES

117 a 1379.93 3.49973 YES YES

118 a 1385.00 9.34631 YES YES

119 a 1386.37 8.98266 YES YES

120 a 1390.61 24.62098 YES YES

121 a 1395.34 48.86821 YES YES

122 a 1399.35 24.58365 YES YES

123 a 1404.10 14.07053 YES YES

124 a 1408.99 18.09292 YES YES

125 a 1428.67 45.61769 YES YES

126 a 1474.85 1.17948 YES YES

127 a 1485.95 2.04166 YES YES

128 a 1486.83 7.09291 YES YES

129 a 1497.47 9.84323 YES YES

130 a 1513.82 1.31951 YES YES

131 a 2911.02 30.91519 YES YES

132 a 2931.29 182.53778 YES YES

133 a 2953.81 124.06060 YES YES

134 a 2956.97 93.87593 YES YES

135 a 2991.24 65.88253 YES YES

136 a 2993.59 21.71571 YES YES

137 a 2994.35 25.38302 YES YES

138 a 3001.93 25.16848 YES YES

139 a 3059.28 19.06627 YES YES

140 a 3105.58 21.93627 YES YES

141 a 3566.90 0.49796 YES YES

$end

Total COSMO energy + OC correction = -2859.6659796107 H

**MOEA**


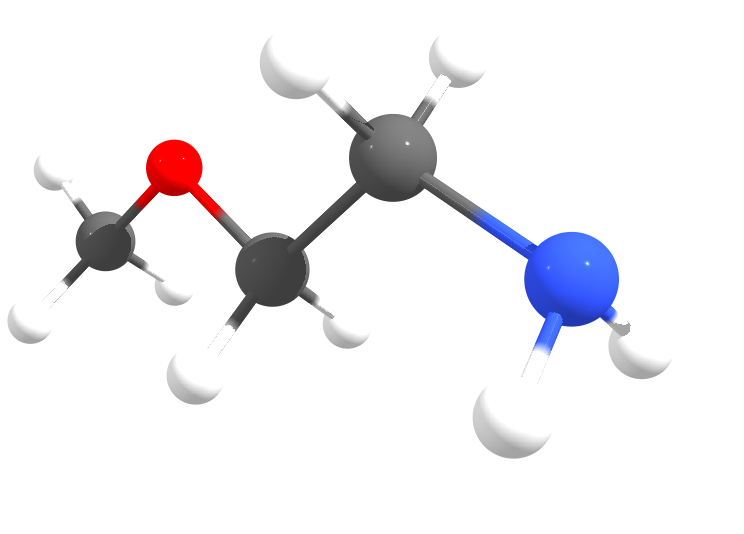


Method: (RI-)B3LYP(D3BJ)/def2-TZVPP

Symmetry: cs

Cartesian coordinates in Ångström:

C -2.2289127 0.7132732 0.0000000

O -0.8457427 0.9856340 0.0000000

H -2.7507169 1.6683859 0.0000000

H -2.5252202 0.1438727 0.8899788

H -2.5252202 0.1438727 -0.8899788

C -0.0504851 -0.1812632 0.0000000

H -0.2730722 -0.7953534 0.8860577

C 1.4192382 0.2196677 0.0000000

H -0.2730722 -0.7953534 -0.8860577

H 1.6159855 0.8413017 0.8752706

H 1.6159855 0.8413017 -0.8752706

N 2.3561448 -0.8993880 0.0000000

H 2.2258944 -1.4859245 -0.8151968

H 2.2258944 -1.4859245 0.8151968

SCF energy GEOOPT = -249.6600540156 H

ZPE = 329.7 kJ/mol

FREEH energy = 347.38 kJ/mol

FREEH entropy = 0.33225 kJ/mol/K

$vibrational spectrum

# mode symmetry wave number IR intensity selection rules

# cm**(-1) km/mol IR RAMAN

1 -0.00 0.00000 - -

2 0.00 0.00000 - -

3 0.00 0.00000 - -

4 0.00 0.00000 - -

5 0.00 0.00000 - -

6 0.00 0.00000 - -

7 a" 101.35 0.00185 YES YES

8 a" 122.81 0.68374 YES YES

9 a' 195.33 0.14755 YES YES

10 a" 232.33 4.81571 YES YES

11 a" 281.06 43.11768 YES YES

12 a' 427.35 1.52434 YES YES

13 a' 436.00 12.65887 YES YES

14 a" 794.55 0.00191 YES YES

15 a' 829.43 190.68792 YES YES

16 a' 980.00 33.01312 YES YES

17 a" 1002.51 0.04662 YES YES

18 a' 1066.09 30.87728 YES YES

19 a' 1095.76 3.03372 YES YES

20 a' 1152.04 150.25432 YES YES

21 a" 1174.88 1.23140 YES YES

22 a" 1217.65 6.46474 YES YES

23 a' 1222.91 34.60048 YES YES

24 a" 1307.31 0.01769 YES YES

25 a' 1350.92 11.44297 YES YES

26 a" 1376.38 0.02816 YES YES

27 a' 1422.37 5.44344 YES YES

28 a' 1478.08 0.93803 YES YES

29 a" 1487.36 6.87803 YES YES

30 a' 1496.15 5.05094 YES YES

31 a' 1504.60 5.68785 YES YES

32 a' 1522.27 1.34280 YES YES

33 a' 1661.22 20.71726 YES YES

34 a' 2936.16 51.46254 YES YES

35 a" 2960.41 64.84153 YES YES

36 a' 2970.06 74.26840 YES YES

37 a" 3009.58 60.09161 YES YES

38 a' 3042.10 32.90766 YES YES

39 a" 3080.97 31.97102 YES YES

40 a' 3110.74 30.16378 YES YES

41 a' 3494.27 1.05440 YES YES

42 a" 3571.97 0.44627 YES YES

$end

Total COSMO energy + OC correction = -249.6685783392 H

**H_2_O**


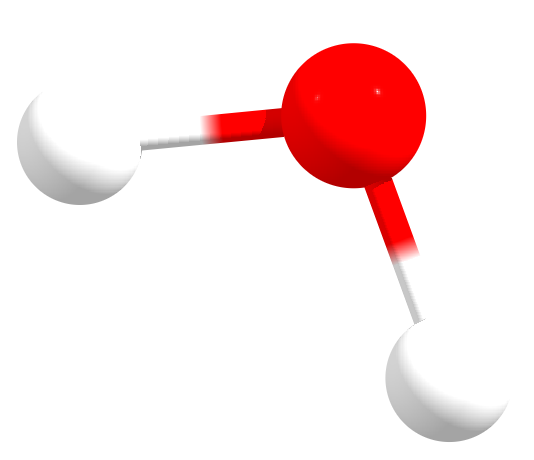


Method: (RI-)B3LYP(D3BJ)/def2-TZVPP

Symmetry: c2v

Cartesian coordinates in Ångström:

O 0.0000000 0.0000000 -0.3903870

H 0.7618347 0.0000000 0.1951933

H -0.7618347 0.0000000 0.1951933

SCF energy GEOOPT = -76.430325351 H

ZPE = 55.92 kJ/mol

FREEH energy = 63.37 kJ/mol

FREEH entropy = 0.18892 kJ/mol/K

$vibrational spectrum

# mode symmetry wave number IR intensity selection rules

# cm**(-1) km/mol IR RAMAN

1 -0.00 0.00000 - -

2 -0.00 0.00000 - -

3 0.00 0.00000 - -

4 0.00 0.00000 - -

5 0.00 0.00000 - -

6 0.00 0.00000 - -

7 a1 1632.68 69.68267 YES YES

8 a1 3807.14 4.37599 YES YES

9 b1 3909.88 48.48977 YES YES

$end

Total COSMO energy + OC correction = -76.4396557299 H

**[Al(Ohfip)_3_OH]^−^**


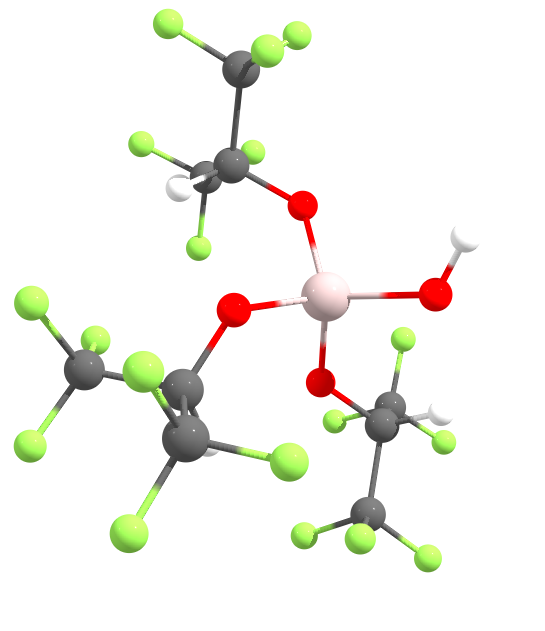


Method: (RI-)B3LYP(D3BJ)/def2-TZVPP

Symmetry: c1

Cartesian coordinates in Ångström:

Al 0.1011862 -0.0268308 -0.0069722

O 0.6946327 1.3450161 -0.9360792

O 1.3940917 -0.7988663 0.8459334

O -1.2339361 0.6562259 0.9293653

O -0.6474010 -1.3098141 -0.9629427

C -2.4708102 0.1204847 1.1308456

F -3.5159377 2.2885206 1.2700245

C -0.1494744 -2.5695643 -1.1267746

C 0.0787328 2.5437930 -1.1373625

F -1.7234987 -1.1422308 2.9786354

C -2.6544631 -0.2447702 2.6164688

F -3.8614210 -0.8059975 2.8619825

F -2.5345991 0.8122622 3.4390009

C -3.5435110 1.1059172 0.6291679

F -4.7966112 0.6148166 0.7486584

F -3.3438775 1.3587656 -0.6776235

H -2.6468342 -0.8101975 0.5744693

F 1.2519697 -1.8794297 -2.8936948

C 0.2992673 -2.7755903 -2.5861142

F 0.8345714 -4.0038852 -2.7888169

F -0.6964295 -2.6262014 -3.4745513

F -0.9246661 3.8758666 -2.8710624

C -0.2722844 2.7142848 -2.6281068

F 0.8054825 2.6907489 -3.4313661

F -1.0851505 1.7191650 -3.0157090

H -0.8717628 2.6517971 -0.5974938

F 1.2127653 3.4917090 0.7036268

C 0.9771252 3.6782451 -0.6090002

F 2.1721799 3.7401053 -1.2217054

F 0.4009697 4.8954588 -0.7338972

F -0.7547532 -4.8712810 -0.7635463

H 0.7390820 -2.7722115 -0.5128633

C -1.2050229 -3.5988371 -0.6831507

F -2.3429006 -3.5334836 -1.3964194

F -1.5318787 -3.3807324 0.6050608

H 2.1297376 -0.2817887 1.1670231

SCF energy GEOOPT = -2686.408702673 H

ZPE = 441.2 kJ/mol

FREEH energy = 527.26 kJ/mol

FREEH entropy = 0.95057 kJ/mol/K

$vibrational spectrum

# mode symmetry wave number IR intensity selection rules

# cm**(-1) km/mol IR RAMAN

1 -0.00 0.00000 - -

2 -0.00 0.00000 - -

3 0.00 0.00000 - -

4 0.00 0.00000 - -

5 0.00 0.00000 - -

6 0.00 0.00000 - -

7 a 9.43 0.05706 YES YES

8 a 12.06 0.22660 YES YES

9 a 15.11 0.10762 YES YES

10 a 16.70 0.01626 YES YES

11 a 21.93 0.19226 YES YES

12 a 24.65 0.09620 YES YES

13 a 29.61 0.04216 YES YES

14 a 30.35 0.00860 YES YES

15 a 30.70 0.00389 YES YES

16 a 37.44 0.80631 YES YES

17 a 49.13 0.51092 YES YES

18 a 56.77 0.10668 YES YES

19 a 68.26 0.54914 YES YES

20 a 71.98 0.16740 YES YES

21 a 87.95 0.27174 YES YES

22 a 102.79 1.67365 YES YES

23 a 125.83 8.82587 YES YES

24 a 150.60 2.00425 YES YES

25 a 156.29 2.54138 YES YES

26 a 170.83 23.59951 YES YES

27 a 173.57 2.51783 YES YES

28 a 187.56 2.24636 YES YES

29 a 191.93 2.28372 YES YES

30 a 222.34 20.03393 YES YES

31 a 231.49 24.03602 YES YES

32 a 256.97 0.25059 YES YES

33 a 267.64 0.30577 YES YES

34 a 279.69 9.27855 YES YES

35 a 290.92 0.18609 YES YES

36 a 291.03 0.19196 YES YES

37 a 291.38 0.15275 YES YES

38 a 296.32 9.28206 YES YES

39 a 312.81 9.41052 YES YES

40 a 325.81 0.52138 YES YES

41 a 327.03 1.49137 YES YES

42 a 333.65 0.69583 YES YES

43 a 354.90 1.60190 YES YES

44 a 359.82 1.03138 YES YES

45 a 374.36 5.80591 YES YES

46 a 403.45 18.42347 YES YES

47 a 435.41 42.93163 YES YES

48 a 436.14 53.23267 YES YES

49 a 515.45 3.17007 YES YES

50 a 516.08 12.61022 YES YES

51 a 516.66 11.64534 YES YES

52 a 527.27 1.23889 YES YES

53 a 527.34 2.94452 YES YES

54 a 527.46 4.59457 YES YES

55 a 536.78 0.97055 YES YES

56 a 543.97 0.46081 YES YES

57 a 544.28 0.45548 YES YES

58 a 544.42 0.61007 YES YES

59 a 560.95 34.02735 YES YES

60 a 563.45 26.09205 YES YES

61 a 666.05 163.29594 YES YES

62 a 680.32 9.73517 YES YES

63 a 681.30 31.72928 YES YES

64 a 682.60 81.35144 YES YES

65 a 687.10 17.85249 YES YES

66 a 710.29 0.43061 YES YES

67 a 724.96 24.94302 YES YES

68 a 747.78 4.57744 YES YES

69 a 771.61 58.68292 YES YES

70 a 797.78 92.85856 YES YES

71 a 815.32 60.08586 YES YES

72 a 856.07 91.34316 YES YES

73 a 856.81 112.30490 YES YES

74 a 858.45 123.89566 YES YES

75 a 884.27 19.89557 YES YES

76 a 885.66 55.61161 YES YES

77 a 886.77 60.55077 YES YES

78 a 1084.47 34.24443 YES YES

79 a 1088.95 81.12722 YES YES

80 a 1091.36 220.43201 YES YES

81 a 1112.37 23.26911 YES YES

82 a 1114.82 9.19845 YES YES

83 a 1119.67 67.20557 YES YES

84 a 1165.00 690.83474 YES YES

85 a 1166.39 337.47292 YES YES

86 a 1168.20 265.03230 YES YES

87 a 1192.14 84.05104 YES YES

88 a 1198.67 244.03743 YES YES

89 a 1202.86 36.91648 YES YES

90 a 1205.64 666.70070 YES YES

91 a 1206.90 860.50996 YES YES

92 a 1235.39 143.75791 YES YES

93 a 1253.10 155.58474 YES YES

94 a 1253.92 132.77763 YES YES

95 a 1256.07 192.42423 YES YES

96 a 1287.88 69.35557 YES YES

97 a 1290.40 152.24197 YES YES

98 a 1291.13 116.83802 YES YES

99 a 1378.30 7.50703 YES YES

100 a 1381.01 10.48521 YES YES

101 a 1384.69 7.36999 YES YES

102 a 1390.45 11.51880 YES YES

103 a 1393.71 46.80688 YES YES

104 a 1395.49 61.62830 YES YES

105 a 2993.86 25.91589 YES YES

106 a 2999.38 21.29395 YES YES

107 a 3000.54 20.01681 YES YES

108 a 3938.93 35.43120 YES YES

$end

Total COSMO energy + OC correction = -2686.4583173086 H

**HOhfip**


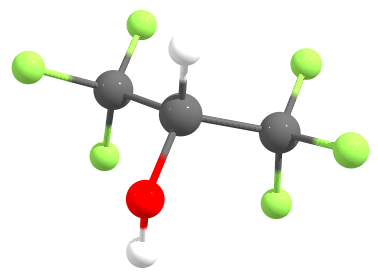


Method: (RI-)B3LYP(D3BJ)/def2-TZVPP

Symmetry: cs

Cartesian coordinates in Ångström:

F 0.2160340 -1.3824187 1.4221858

F 0.2160340 -1.3824187 -1.4221858

H -1.7861925 -0.8136868 0.0000000

C 0.3553527 -0.0486129 1.2918868

C 0.3553527 -0.0486129 -1.2918868

F 1.6615837 0.2409685 1.3403382

F 1.6615837 0.2409685 -1.3403382

O -1.6753694 0.1444764 0.0000000

C -0.3138445 0.4562734 0.0000000

F -0.2324313 0.5252562 2.3512505

F -0.2324313 0.5252562 -2.3512505

H -0.2256093 1.5425194 0.0000000

SCF energy GEOOPT = -789.8666264350 H

ZPE = 162.6 kJ/mol

FREEH energy = 186.60 kJ/mol

FREEH entropy = 0.40139 kJ/mol/K

$vibrational spectrum

# mode symmetry wave number IR intensity selection rules

# cm**(-1) km/mol IR RAMAN

1 -0.00 0.00000 - -

2 -0.00 0.00000 - -

3 -0.00 0.00000 - -

4 -0.00 0.00000 - -

5 0.00 0.00000 - -

6 0.00 0.00000 - -

7 a" 27.28 0.11147 YES YES

8 a' 90.01 0.30922 YES YES

9 a' 166.57 0.86336 YES YES

10 a' 227.74 6.75016 YES YES

11 a" 247.10 1.57910 YES YES

12 a" 287.48 13.02808 YES YES

13 a' 322.01 0.41388 YES YES

14 a" 339.38 15.75599 YES YES

15 a" 379.69 94.44921 YES YES

16 a' 455.78 8.51696 YES YES

17 a' 510.36 6.24578 YES YES

18 a" 528.05 2.94163 YES YES

19 a" 547.72 3.40427 YES YES

20 a' 603.71 2.16204 YES YES

21 a" 685.03 53.06137 YES YES

22 a' 734.12 12.34966 YES YES

23 a' 827.65 37.24656 YES YES

24 a" 892.68 46.30611 YES YES

25 a" 1097.49 148.35459 YES YES

26 a' 1112.29 120.57996 YES YES

27 a" 1162.37 19.53940 YES YES

28 a' 1191.77 380.61565 YES YES

29 a' 1220.24 302.67889 YES YES

30 a" 1246.36 146.56828 YES YES

31 a' 1270.83 132.88567 YES YES

32 a' 1307.73 129.09798 YES YES

33 a" 1378.35 87.97052 YES YES

34 a' 1426.47 11.73967 YES YES

35 a' 3102.75 3.49699 YES YES

36 a' 3790.14 60.92739 YES YES

$end

Total COSMO energy + OC correction = -789.8734887128 H

**[H–MOEA]^+^**


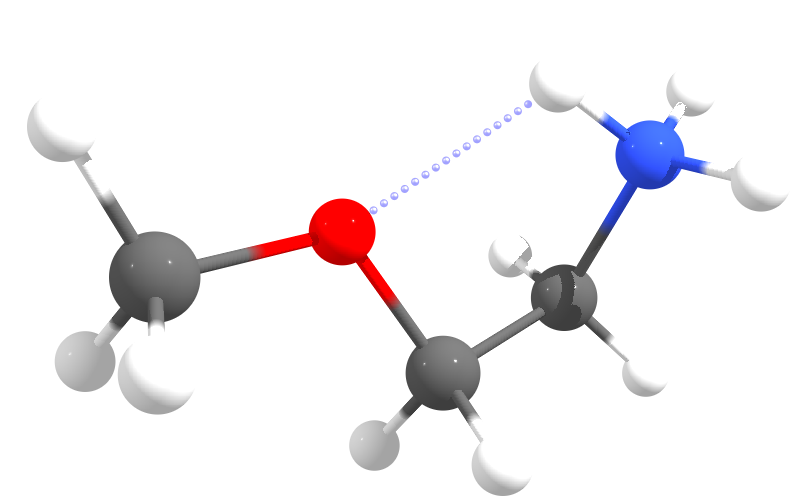


Method: (RI-)B3LYP(D3BJ)/def2-TZVPP

Symmetry: c1

Cartesian coordinates in Ångström:

C -2.5150209 0.7135518 -0.0797191

O -1.1191763 0.9784091 -0.2698837

H -3.0463448 1.6135319 -0.3731444

H -2.7231402 0.4853543 0.9680309

H -2.8301018 -0.1206496 -0.7087870

C -0.2692721 -0.0889841 0.0866739

H -0.3614130 -0.3252988 1.1547317

C 1.1321267 0.3838114 -0.2586815

H -0.4888394 -0.9980814 -0.4808605

H 1.2619870 0.4616576 -1.3351822

H 1.9137350 -0.2395515 0.1662926

N 1.2707388 1.7908267 0.2863655

H 0.3684624 2.2514881 0.0896435

H 2.0323309 2.3103607 -0.1490169

H 1.4140977 1.7974837 1.2971671

SCF energy GEOOPT = -250.0320973860 H

ZPE = 370.0 kJ/mol

FREEH energy = 387.49 kJ/mol

FREEH entropy = 0.33066 kJ/mol/K

$vibrational spectrum

# mode symmetry wave number IR intensity selection rules

# cm**(-1) km/mol IR RAMAN

1 -0.00 0.00000 - -

2 0.00 0.00000 - -

3 0.00 0.00000 - -

4 0.00 0.00000 - -

5 0.00 0.00000 - -

6 0.00 0.00000 - -

7 a 96.33 10.77039 YES YES

8 a 159.00 14.00838 YES YES

9 a 209.18 17.19917 YES YES

10 a 240.38 2.78599 YES YES

11 a 278.69 4.08932 YES YES

12 a 372.20 25.75187 YES YES

13 a 528.91 6.37732 YES YES

14 a 804.29 17.39551 YES YES

15 a 859.81 11.89846 YES YES

16 a 946.16 14.05136 YES YES

17 a 969.70 9.40264 YES YES

18 a 1011.74 24.32826 YES YES

19 a 1099.28 117.89894 YES YES

20 a 1132.43 118.94330 YES YES

21 a 1165.30 2.66893 YES YES

22 a 1192.68 2.79108 YES YES

23 a 1228.43 12.75558 YES YES

24 a 1276.83 5.11818 YES YES

25 a 1340.65 15.38595 YES YES

26 a 1394.92 6.19099 YES YES

27 a 1426.64 3.42309 YES YES

28 a 1472.35 104.26216 YES YES

29 a 1489.09 25.00802 YES YES

30 a 1493.12 37.20615 YES YES

31 a 1497.79 14.07031 YES YES

32 a 1504.02 24.97574 YES YES

33 a 1525.37 13.57105 YES YES

34 a 1644.75 31.64745 YES YES

35 a 1672.52 34.31706 YES YES

36 a 2987.69 25.35696 YES YES

37 a 3024.46 26.90384 YES YES

38 a 3048.37 10.01101 YES YES

39 a 3091.34 18.51071 YES YES

40 a 3101.91 0.96281 YES YES

41 a 3156.61 4.77914 YES YES

42 a 3163.35 0.28883 YES YES

43 a 3293.89 134.15204 YES YES

44 a 3452.20 108.68011 YES YES

45 a 3505.00 100.47359 YES YES

$end

Total COSMO energy + OC correction = -250.1140116845 H

**[OH]^−^**


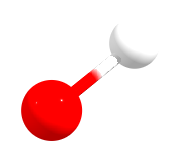


Method: (RI-)B3LYP(D3BJ)/def2-TZVPP

Symmetry: c6v

Cartesian coordinates in Ångström:

O 0.0000000 0.0000000 0.4840151

H 0.0000000 0.0000000 -0.4840151

SCF energy GEOOPT = -75.776424081 H

ZPE = 21.92 kJ/mol

FREEH energy = 28.11 kJ/mol

FREEH entropy = 0.17254 kJ/mol/K

$vibrational spectrum

# mode symmetry wave number IR intensity selection rules

# cm**(-1) km/mol IR RAMAN

1 0.00 0.00000 - -

2 0.00 0.00000 - -

3 0.00 0.00000 - -

4 0.00 0.00000 - -

5 0.00 0.00000 - -

6 a1 3664.18 205.87256 YES YES

$end

Total COSMO energy + OC correction = -75.9053013571 H

**[Al(Ohfip)_4_OH]^2−^**


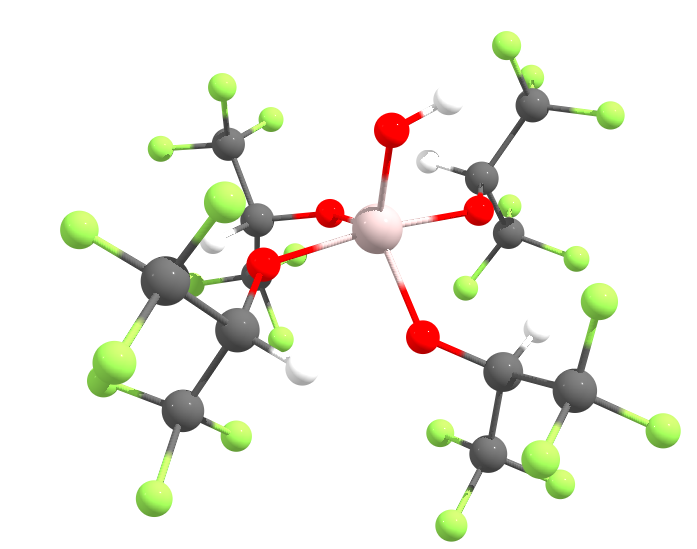


Method: (RI-)B3LYP(D3BJ)/def2-TZVPP

Symmetry: c1

Cartesian coordinates in Ångström:

Al -0.0659310 -0.0851315 0.6921038

O 0.8549462 1.5499250 0.4669459

O 1.3891427 -0.9612802 -0.0121414

O -1.4385965 0.6345199 -0.2963205

O -0.9785559 -1.7924327 0.6135324

C 2.5817320 -0.4613872 -0.4041586

H 2.6429461 0.6334045 -0.3294335

C -2.5829991 0.0705306 -0.7367590

F -2.8018062 1.5499296 -2.6444598

C -0.4370434 -3.0189389 0.5151938

C 0.3233461 2.7683705 0.2681038

F -3.7110331 0.3938605 1.3221527

C -3.7944442 0.6643094 0.0104184

F -4.9869688 0.1528069 -0.4113239

F -3.8981516 2.0063157 -0.1084108

C -2.7098933 0.2564680 -2.2623618

F -3.8049608 -0.3705740 -2.7809625

F -1.6447119 -0.2637369 -2.8855405

H -2.6349077 -1.0132265 -0.5613451

F 4.9427504 -0.5458624 0.1460794

C 3.7072684 -1.0036661 0.4987305

F 3.7940177 -2.3536417 0.5014680

F 3.5108873 -0.6235390 1.7692359

F 2.9033453 -2.1234703 -2.1381743

C 2.8314529 -0.7962989 -1.8883516

F 1.8448185 -0.3058356 -2.6489688

F 3.9910633 -0.2579242 -2.3638259

F -0.5315473 -3.0028303 -1.8546678

C -0.9014261 -3.7165996 -0.7837385

F -0.3716862 -4.9632833 -0.9474915

F -2.2456428 -3.8682347 -0.8588662

F 0.2240500 4.5453167 -1.3915135

C 0.8146026 3.3572721 -1.0758586

F 2.1516736 3.5841548 -1.1045826

F 0.5432437 2.5202508 -2.0842394

H -0.7749364 2.7757363 0.1900681

F 0.0943058 3.2737009 2.5771065

C 0.6600556 3.7110246 1.4456497

F 1.9850475 3.8258933 1.6810427

F 0.1996132 4.9868220 1.2578127

F -0.2153766 -5.1071529 1.7446779

H 0.6627672 -3.0217870 0.4569902

C -0.7863281 -3.8689438 1.7569527

F -2.1120762 -4.0682863 1.9272331

F -0.3402672 -3.2648954 2.8751455

O -0.1930518 0.0507513 2.4500667

H -0.6379140 -0.7168340 2.8094259

SCF energy GEOOPT = -3475.676896643 H

ZPE = 570.6 kJ/mol

FREEH energy = 683.29 kJ/mol

FREEH entropy = 1.16393 kJ/mol/K

$vibrational spectrum

# mode symmetry wave number IR intensity selection rules

# cm**(-1) km/mol IR RAMAN

1 -0.00 0.00000 - -

2 -0.00 0.00000 - -

3 -0.00 0.00000 - -

4 -0.00 0.00000 - -

5 -0.00 0.00000 - -

6 0.00 0.00000 - -

7 a 9.63 0.11152 YES YES

8 a 11.85 0.25530 YES YES

9 a 13.75 0.05513 YES YES

10 a 15.78 0.07893 YES YES

11 a 17.12 0.05417 YES YES

12 a 21.59 0.22958 YES YES

13 a 24.17 0.12719 YES YES

14 a 26.09 0.02952 YES YES

15 a 28.46 0.04379 YES YES

16 a 30.42 0.09827 YES YES

17 a 33.19 0.44762 YES YES

18 a 34.32 0.26660 YES YES

19 a 35.80 0.26662 YES YES

20 a 36.31 0.23326 YES YES

21 a 53.97 0.04687 YES YES

22 a 55.32 0.24774 YES YES

23 a 60.66 0.03732 YES YES

24 a 68.94 0.10676 YES YES

25 a 70.41 0.03555 YES YES

26 a 79.30 0.18974 YES YES

27 a 90.99 0.19073 YES YES

28 a 100.44 0.03876 YES YES

29 a 126.86 0.77108 YES YES

30 a 151.53 0.21808 YES YES

31 a 160.10 1.10227 YES YES

32 a 161.89 0.11354 YES YES

33 a 178.55 0.31247 YES YES

34 a 187.39 0.53458 YES YES

35 a 190.82 1.48115 YES YES

36 a 201.06 0.03730 YES YES

37 a 206.15 2.92060 YES YES

38 a 214.73 0.09424 YES YES

39 a 229.30 0.41525 YES YES

40 a 262.81 0.15064 YES YES

41 a 263.82 0.22050 YES YES

42 a 274.73 0.28162 YES YES

43 a 285.67 3.74093 YES YES

44 a 287.17 0.04504 YES YES

45 a 288.33 0.75124 YES YES

46 a 288.51 0.10976 YES YES

47 a 295.36 10.39876 YES YES

48 a 301.19 20.11083 YES YES

49 a 309.42 6.03057 YES YES

50 a 322.20 0.07401 YES YES

51 a 324.95 0.05153 YES YES

52 a 328.72 0.24884 YES YES

53 a 335.23 0.79134 YES YES

54 a 349.13 3.93157 YES YES

55 a 354.16 0.21999 YES YES

56 a 355.62 7.33344 YES YES

57 a 367.72 14.30833 YES YES

58 a 381.35 10.76824 YES YES

59 a 401.16 17.10811 YES YES

60 a 412.01 17.34679 YES YES

61 a 432.82 147.78939 YES YES

62 a 456.08 120.79406 YES YES

63 a 493.00 0.38796 YES YES

64 a 514.45 1.25062 YES YES

65 a 516.01 0.45433 YES YES

66 a 516.83 13.29402 YES YES

67 a 517.84 10.61621 YES YES

68 a 520.39 3.15669 YES YES

69 a 522.06 6.62272 YES YES

70 a 522.42 1.75788 YES YES

71 a 523.78 1.68327 YES YES

72 a 524.01 6.86517 YES YES

73 a 525.05 73.13817 YES YES

74 a 540.38 0.65300 YES YES

75 a 540.88 0.57390 YES YES

76 a 541.64 0.37753 YES YES

77 a 541.99 0.39094 YES YES

78 a 550.91 90.55659 YES YES

79 a 626.44 2.29743 YES YES

80 a 648.30 6.51427 YES YES

81 a 656.67 12.46195 YES YES

82 a 676.66 12.64293 YES YES

83 a 676.77 10.12608 YES YES

84 a 678.28 14.26963 YES YES

85 a 679.44 88.11573 YES YES

86 a 685.94 10.58876 YES YES

87 a 722.29 103.08637 YES YES

88 a 734.13 13.29461 YES YES

89 a 736.50 23.42519 YES YES

90 a 744.22 46.52305 YES YES

91 a 744.90 49.73608 YES YES

92 a 815.99 144.39263 YES YES

93 a 844.20 1.09874 YES YES

94 a 845.53 229.37270 YES YES

95 a 848.82 16.60440 YES YES

96 a 853.13 286.42375 YES YES

97 a 865.47 17.38511 YES YES

98 a 865.99 46.71752 YES YES

99 a 873.51 17.00592 YES YES

100 a 874.30 66.60207 YES YES

101 a 1054.61 42.54181 YES YES

102 a 1059.33 60.14324 YES YES

103 a 1063.56 18.28412 YES YES

104 a 1063.90 23.68560 YES YES

105 a 1090.14 18.20356 YES YES

106 a 1093.97 23.98121 YES YES

107 a 1098.67 23.29181 YES YES

108 a 1109.07 324.50154 YES YES

109 a 1125.70 30.91532 YES YES

110 a 1127.26 856.49478 YES YES

111 a 1130.79 925.94940 YES YES

112 a 1131.66 64.02077 YES YES

113 a 1190.49 345.49199 YES YES

114 a 1197.43 403.03760 YES YES

115 a 1200.61 512.34663 YES YES

116 a 1206.47 168.16602 YES YES

117 a 1212.61 36.80743 YES YES

118 a 1219.00 517.60713 YES YES

119 a 1223.91 504.31794 YES YES

120 a 1238.50 64.97948 YES YES

121 a 1239.63 168.24728 YES YES

122 a 1244.84 75.35201 YES YES

123 a 1247.05 412.33103 YES YES

124 a 1249.60 36.43772 YES YES

125 a 1272.12 24.52569 YES YES

126 a 1275.84 119.27712 YES YES

127 a 1281.91 123.69517 YES YES

128 a 1283.45 2.38765 YES YES

129 a 1329.62 9.15873 YES YES

130 a 1343.34 1.18412 YES YES

131 a 1347.54 0.07549 YES YES

132 a 1358.39 23.71273 YES YES

133 a 1385.09 19.28060 YES YES

134 a 1386.67 36.15012 YES YES

135 a 1397.24 13.51446 YES YES

136 a 1397.83 56.91918 YES YES

137 a 2963.95 48.20391 YES YES

138 a 2966.53 36.09129 YES YES

139 a 2990.63 74.39997 YES YES

140 a 2991.30 14.48192 YES YES

141 a 3896.88 6.13050 YES YES

$end

Total COSMO energy + OC correction = -3475.8429620786 H

**[Ohfip]^−^**


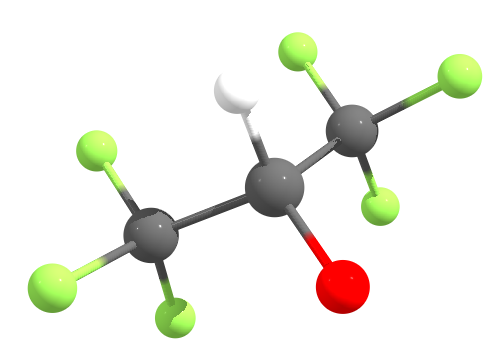


Method: (RI-)B3LYP(D3BJ)/def2-TZVPP

Symmetry: cs

Cartesian coordinates in Ångström:

F 0.0967278 -1.4718214 1.4313886

F 0.0967278 -1.4718214 -1.4313886

C 0.1615594 -0.1304857 1.2778167

C 0.1615594 -0.1304857 -1.2778167

F 1.4927043 0.1907166 1.3569983

F 1.4927043 0.1907166 -1.3569983

O -1.8482578 0.1152877 0.0000000

C -0.5769492 0.3980635 0.0000000

F -0.3998017 0.4093422 2.3859884

F -0.3998017 0.4093422 -2.3859884

H -0.2771726 1.4911452 0.0000000

SCF energy GEOOPT = -789.3013746030 H

ZPE = 123.4 kJ/mol

FREEH energy = 146.92 kJ/mol

FREEH entropy = 0.39928 kJ/mol/K

$vibrational spectrum

# mode symmetry wave number IR intensity selection rules

# cm**(-1) km/mol IR RAMAN

1 -0.00 0.00000 - -

2 -0.00 0.00000 - -

3 -0.00 0.00000 - -

4 -0.00 0.00000 - -

5 -0.00 0.00000 - -

6 -0.00 0.00000 - -

7 a" 27.68 0.00011 YES YES

8 a' 86.60 3.10160 YES YES

9 a' 161.82 0.73325 YES YES

10 a' 232.54 6.21726 YES YES

11 a" 244.11 9.10177 YES YES

12 a" 273.44 0.11651 YES YES

13 a' 305.87 0.82984 YES YES

14 a" 334.07 2.49081 YES YES

15 a' 449.94 0.74299 YES YES

16 a' 506.23 4.97791 YES YES

17 a" 511.81 5.17226 YES YES

18 a" 531.27 0.42257 YES YES

19 a' 583.63 3.83232 YES YES

20 a" 672.36 38.68570 YES YES

21 a' 719.65 8.20770 YES YES

22 a' 814.01 61.22463 YES YES

23 a" 833.74 50.04651 YES YES

24 a" 1033.33 5.71850 YES YES

25 a" 1058.56 193.67384 YES YES

26 a' 1097.70 432.50605 YES YES

27 a' 1156.73 265.76538 YES YES

28 a" 1213.36 199.94938 YES YES

29 a' 1233.61 121.04388 YES YES

30 a' 1272.27 221.93660 YES YES

31 a" 1328.51 3.97823 YES YES

32 a' 1358.18 77.44442 YES YES

33 a' 2588.72 315.11200 YES YES

$end

Total COSMO energy + OC correction = -789.3785506665 H

**[(Ohfip)_3_Al(OH)_2_Al(Ohfip)_3_]^2−^**


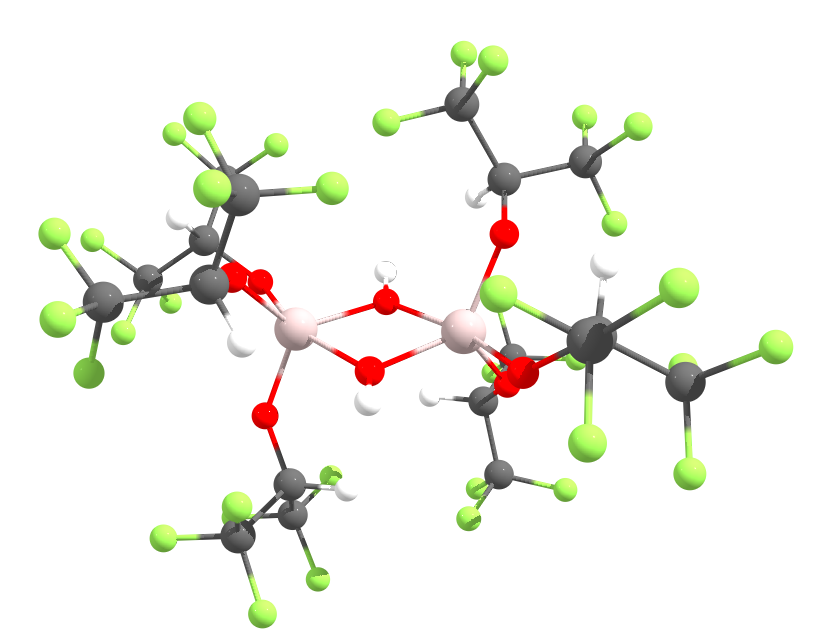


Method: (RI-)B3LYP(D3BJ)/def2-TZVPP

Symmetry: c1

Cartesian coordinates in Ångström:

Al 0.4654640 0.1123935 0.1563624

O 0.4825832 1.9352147 0.0664437

O 1.9274483 0.1645606 1.2703638

O -1.2515343 0.0642845 0.6713819

O 0.7399055 -0.1868757 -1.5804121

C -2.1390096 -0.9424028 0.8320693

F -4.0295850 0.4177360 0.1959724

C 1.2678122 -1.2494747 -2.2305589

C -0.5529976 2.8026318 0.1249797

F -1.4722099 -1.3516569 3.0590367

C -2.5455825 -1.0621634 2.3168971

F -3.4525104 -2.0509240 2.5258658

F -3.0848324 0.0711176 2.8041792

C -3.3552247 -0.7133652 -0.0909275

F -4.2523767 -1.7303838 -0.0328603

F -2.9534500 -0.6246404 -1.3664222

H -1.7628071 -1.9397350 0.5610822

F 3.2358626 -0.0297841 -2.7035427

C 2.2643894 -0.7476053 -3.2953689

F 2.8746520 -1.7579169 -3.9603453

F 1.6958211 0.0424321 -4.2248362

F -1.9213872 4.2434953 -1.2560738

C -0.8994201 3.3424114 -1.2793106

F 0.1351770 3.9529099 -1.8879172

F -1.2941766 2.3369013 -2.0721065

H -1.4858201 2.3563417 0.4993614

F 0.0843128 3.4268933 2.3164987

C -0.2185165 3.9394359 1.1111498

F 0.8342006 4.6923040 0.7282034

F -1.2562326 4.7937821 1.3041440

F 0.6352169 -3.1984194 -3.5143297

H 1.8423065 -1.9385339 -1.5950232

C 0.1533348 -2.1195666 -2.8500758

F -0.6367011 -1.4519506 -3.7045580

F -0.6419960 -2.6032262 -1.8697236

H 2.2300505 1.0286272 1.5401865

F 1.3931348 1.6335434 5.7014334

F 0.4898741 -0.3379379 5.8132278

C 1.1145157 0.5154266 4.9817929

F 0.2414848 0.8703213 4.0328042

F 3.8984472 0.6944895 6.0348901

F 3.0741110 -1.2966694 6.3053940

C 3.4735796 -0.4105050 5.3710901

C 2.3757044 -0.0935991 4.3321704

H 2.7975520 0.7155164 3.7180330

F 4.8611485 0.7173913 2.1122942

O 2.0546182 -1.2174773 3.6526460

F 4.5460478 -0.9319564 4.7607607

C 5.5501503 -0.0868103 1.2717416

F 6.4238736 -0.7794382 2.0182711

F 6.2737922 0.7329069 0.4710664

O 0.8351620 -1.7338253 0.6728499

Al 2.1850497 -1.6603427 1.9186311

H 0.4021394 -2.5752182 0.5492086

F -0.4119607 -4.2240380 3.4142400

O 3.8631631 -1.7724777 1.3246584

H 1.9222416 -3.4300316 4.3145482

C 4.5665665 -0.9789407 0.4843847

H 3.9574273 -0.2766534 -0.1027315

C 1.9503802 -4.0933123 3.4378564

O 1.9349852 -3.4367161 2.2557414

C 0.6922461 -4.9771151 3.5538231

F 0.5896408 -5.5951976 4.7586617

C 5.2953814 -1.8580830 -0.5525148

F 4.3101172 -4.0991076 3.5916813

C 3.2476234 -4.9152470 3.5924832

F 3.2909716 -5.6092301 4.7641175

F 6.1648518 -2.7227543 0.0018780

F 5.9900797 -1.1320257 -1.4607568

F 0.6282946 -5.9466666 2.6168172

F 4.3969811 -2.5848831 -1.2414478

F 3.4308268 -5.8157536 2.6075089

SCF energy GEOOPT = -5372.786721338 H

ZPE = 889.0 kJ/mol

FREEH energy = 1062.31 kJ/mol

FREEH entropy = 1.66116 kJ/mol/K

$vibrational spectrum

# mode symmetry wave number IR intensity selection rules

# cm**(-1) km/mol IR RAMAN

1 -0.00 0.00000 - -

2 0.00 0.00000 - -

3 0.00 0.00000 - -

4 0.00 0.00000 - -

5 0.00 0.00000 - -

6 0.00 0.00000 - -

7 a 4.62 0.16562 YES YES

8 a 7.19 0.07248 YES YES

9 a 8.82 0.05003 YES YES

10 a 12.45 0.00487 YES YES

11 a 14.81 0.02092 YES YES

12 a 17.50 0.04090 YES YES

13 a 19.48 0.06234 YES YES

14 a 20.67 0.05368 YES YES

15 a 22.55 0.00478 YES YES

16 a 23.51 0.07639 YES YES

17 a 25.26 0.07042 YES YES

18 a 26.03 0.02191 YES YES

19 a 26.67 0.01928 YES YES

20 a 27.83 0.01323 YES YES

21 a 29.78 0.10645 YES YES

22 a 30.39 0.08832 YES YES

23 a 32.39 0.14149 YES YES

24 a 35.15 0.04681 YES YES

25 a 37.67 0.04732 YES YES

26 a 38.48 0.37359 YES YES

27 a 40.63 0.03822 YES YES

28 a 42.04 0.12923 YES YES

29 a 45.61 0.15436 YES YES

30 a 49.60 0.14230 YES YES

31 a 53.42 0.04667 YES YES

32 a 59.81 0.19963 YES YES

33 a 62.83 0.00746 YES YES

34 a 67.29 0.10364 YES YES

35 a 69.81 0.02215 YES YES

36 a 72.46 0.06399 YES YES

37 a 73.22 0.01472 YES YES

38 a 78.22 0.01783 YES YES

39 a 80.28 0.09145 YES YES

40 a 84.84 0.06970 YES YES

41 a 100.14 0.42219 YES YES

42 a 101.89 0.07258 YES YES

43 a 134.27 0.03433 YES YES

44 a 141.72 4.32142 YES YES

45 a 142.87 1.58677 YES YES

46 a 151.89 1.12834 YES YES

47 a 154.36 0.39687 YES YES

48 a 171.29 0.52783 YES YES

49 a 173.91 2.11063 YES YES

50 a 181.03 0.22887 YES YES

51 a 185.82 0.08739 YES YES

52 a 191.04 1.43562 YES YES

53 a 192.01 0.18771 YES YES

54 a 200.84 0.87589 YES YES

55 a 203.94 0.10199 YES YES

56 a 210.58 2.78532 YES YES

57 a 216.53 2.32993 YES YES

58 a 250.07 3.01937 YES YES

59 a 254.76 1.62308 YES YES

60 a 260.35 3.31394 YES YES

61 a 265.22 0.21754 YES YES

62 a 268.47 1.02264 YES YES

63 a 269.09 0.81690 YES YES

64 a 281.74 20.73094 YES YES

65 a 289.26 0.03371 YES YES

66 a 289.27 0.26148 YES YES

67 a 289.35 0.18041 YES YES

68 a 289.53 0.10138 YES YES

69 a 290.85 0.19462 YES YES

70 a 291.18 0.24347 YES YES

71 a 299.49 0.99972 YES YES

72 a 309.36 2.62305 YES YES

73 a 319.08 2.80535 YES YES

74 a 322.27 9.17425 YES YES

75 a 324.06 1.72412 YES YES

76 a 325.25 1.54073 YES YES

77 a 326.63 0.07395 YES YES

78 a 328.54 0.13026 YES YES

79 a 330.30 1.05422 YES YES

80 a 337.16 1.12828 YES YES

81 a 341.46 0.95358 YES YES

82 a 346.15 8.05493 YES YES

83 a 346.82 2.44647 YES YES

84 a 365.80 1.04195 YES YES

85 a 368.85 8.91419 YES YES

86 a 370.10 0.62472 YES YES

87 a 373.10 6.64196 YES YES

88 a 382.92 7.67119 YES YES

89 a 402.28 0.97044 YES YES

90 a 414.89 13.98310 YES YES

91 a 420.08 117.74472 YES YES

92 a 437.95 3.08781 YES YES

93 a 442.90 44.88358 YES YES

94 a 457.89 195.94925 YES YES

95 a 467.65 101.10417 YES YES

96 a 487.62 48.01687 YES YES

97 a 515.80 16.15994 YES YES

98 a 516.70 0.85486 YES YES

99 a 516.80 16.06334 YES YES

100 a 517.08 12.02013 YES YES

101 a 517.33 8.12500 YES YES

102 a 517.52 1.87032 YES YES

103 a 525.03 1.56868 YES YES

104 a 525.11 4.21028 YES YES

105 a 525.67 4.83077 YES YES

106 a 526.07 2.77122 YES YES

107 a 527.49 2.45510 YES YES

108 a 527.70 1.55088 YES YES

109 a 530.78 2.32274 YES YES

110 a 537.81 97.02850 YES YES

111 a 540.79 0.18133 YES YES

112 a 541.34 1.30736 YES YES

113 a 542.38 0.64404 YES YES

114 a 542.50 0.46893 YES YES

115 a 542.60 0.40024 YES YES

116 a 543.53 3.73210 YES YES

117 a 543.75 0.90891 YES YES

118 a 555.36 0.68419 YES YES

119 a 556.88 0.53452 YES YES

120 a 561.05 103.74041 YES YES

121 a 621.59 136.83631 YES YES

122 a 645.87 0.09708 YES YES

123 a 677.22 13.83570 YES YES

124 a 678.16 6.67349 YES YES

125 a 680.13 47.62168 YES YES

126 a 681.64 45.55937 YES YES

127 a 681.81 35.71257 YES YES

128 a 683.05 1.17882 YES YES

129 a 683.76 71.46552 YES YES

130 a 684.95 12.49685 YES YES

131 a 699.90 0.99301 YES YES

132 a 707.56 1.24461 YES YES

133 a 708.75 66.67115 YES YES

134 a 714.00 16.98492 YES YES

135 a 745.99 5.11053 YES YES

136 a 746.74 70.75983 YES YES

137 a 753.50 67.83827 YES YES

138 a 757.98 0.14959 YES YES

139 a 764.59 10.46499 YES YES

140 a 768.04 118.53861 YES YES

141 a 842.92 15.16968 YES YES

142 a 849.79 225.34700 YES YES

143 a 851.35 32.04028 YES YES

144 a 852.63 41.56316 YES YES

145 a 853.50 258.67216 YES YES

146 a 856.41 68.53891 YES YES

147 a 871.49 132.49189 YES YES

148 a 877.22 46.01895 YES YES

149 a 877.81 32.38002 YES YES

150 a 880.02 179.36866 YES YES

151 a 881.23 33.59113 YES YES

152 a 882.09 9.70620 YES YES

153 a 885.40 23.91817 YES YES

154 a 892.04 449.68522 YES YES

155 a 1075.44 38.40291 YES YES

156 a 1075.81 32.48084 YES YES

157 a 1076.07 141.11872 YES YES

158 a 1080.23 70.10214 YES YES

159 a 1082.10 31.89132 YES YES

160 a 1082.76 106.42609 YES YES

161 a 1099.51 24.27582 YES YES

162 a 1101.33 19.75812 YES YES

163 a 1101.84 35.14776 YES YES

164 a 1102.64 48.06084 YES YES

165 a 1111.99 192.33019 YES YES

166 a 1121.31 175.82266 YES YES

167 a 1144.40 574.51050 YES YES

168 a 1144.84 440.40210 YES YES

169 a 1151.35 594.40370 YES YES

170 a 1151.44 27.54516 YES YES

171 a 1157.22 341.49431 YES YES

172 a 1161.23 814.75726 YES YES

173 a 1186.49 311.23670 YES YES

174 a 1187.42 157.83864 YES YES

175 a 1199.78 96.55894 YES YES

176 a 1202.70 433.59696 YES YES

177 a 1210.45 307.66164 YES YES

178 a 1214.27 7.17084 YES YES

179 a 1216.02 651.88683 YES YES

180 a 1216.97 209.50087 YES YES

181 a 1218.78 340.83694 YES YES

182 a 1220.27 929.86432 YES YES

183 a 1240.90 171.37663 YES YES

184 a 1247.01 210.72902 YES YES

185 a 1248.75 67.92854 YES YES

186 a 1249.24 262.64689 YES YES

187 a 1250.31 126.94453 YES YES

188 a 1253.08 150.07880 YES YES

189 a 1254.38 250.62416 YES YES

190 a 1254.58 18.06391 YES YES

191 a 1281.60 7.90079 YES YES

192 a 1282.06 163.95685 YES YES

193 a 1284.44 302.72689 YES YES

194 a 1286.68 36.44490 YES YES

195 a 1287.00 8.17194 YES YES

196 a 1287.25 129.03820 YES YES

197 a 1363.37 3.87026 YES YES

198 a 1364.16 0.84539 YES YES

199 a 1373.22 59.45946 YES YES

200 a 1375.02 24.23560 YES YES

201 a 1375.87 72.19321 YES YES

202 a 1376.22 3.88113 YES YES

203 a 1386.42 18.03188 YES YES

204 a 1388.43 14.80726 YES YES

205 a 1391.04 38.97899 YES YES

206 a 1391.27 32.48705 YES YES

207 a 1394.62 15.74280 YES YES

208 a 1399.09 27.10661 YES YES

209 a 2976.41 0.54029 YES YES

210 a 2976.68 71.34143 YES YES

211 a 2982.88 34.91942 YES YES

212 a 2984.06 34.26570 YES YES

213 a 2986.60 20.60008 YES YES

214 a 2988.07 20.21368 YES YES

215 a 3942.12 5.68474 YES YES

216 a 3945.68 82.10241 YES YES

$end

Total COSMO energy + OC correction = -5372.9292750812 H

**[Mg(MOEA)_3_]^2+^**


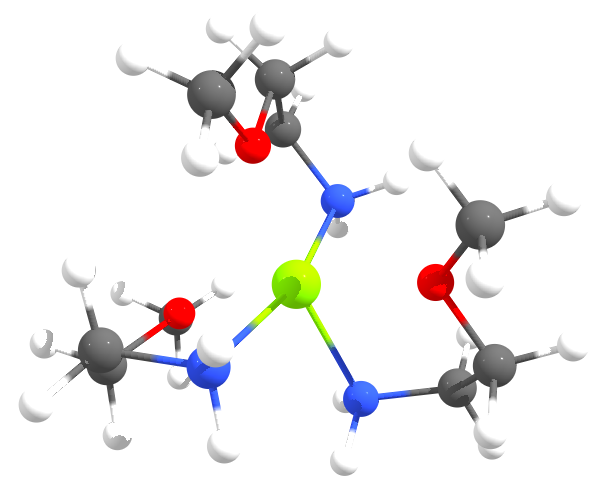


Method: (RI-)B3LYP(D3BJ)/def2-TZVPP

Symmetry: c1

Cartesian coordinates in Ångström:

C 0.3195855 2.2889266 -1.9683605

O 0.4457639 1.7137685 -0.6551137

H 1.2945722 2.6215697 -2.3235904

H -0.3676071 3.1352428 -1.9467288

H -0.0743091 1.5288595 -2.6350577

C 0.7962828 2.7118664 0.3292572

H 1.5874699 3.3497520 -0.0655569

C 1.2705161 2.0088392 1.5789314

H -0.0839951 3.3318336 0.5223884

H 2.2283470 1.5257707 1.3923323

N 0.2905173 0.9528039 1.9473830

H 1.4108735 2.7421276 2.3738087

Mg -0.0814732 -0.2143219 0.1242464

H -0.5810097 1.3931539 2.2339325

H 0.6212956 0.4650906 2.7739402

H -3.2942169 0.6078141 -1.5666459

C -2.8979541 1.1204131 -0.6888783

H -3.7176316 1.5607741 -0.1226009

H -3.2791905 -1.5262045 -0.1618050

H -2.2072083 1.8992493 -0.9918825

H -1.0081251 -2.7940167 0.5983823

C -3.0018355 -0.8592353 0.6604126

O -2.1685321 0.1991839 0.1440559

N -0.8930441 -1.9811369 1.1982915

H -3.9147555 -0.4325747 1.0771998

C -2.2241568 -1.5854650 1.7334380

H -2.8010233 -2.4401537 2.0881571

H -0.3113513 -2.3104946 1.9628491

H -2.0598961 -0.9235799 2.5829667

H 1.7395172 -0.1350599 -2.4680270

H -0.5510932 -0.6385520 -2.6063620

C 1.4168693 -1.1563050 -2.2754815

H 1.5832571 -1.7383279 -3.1823080

N -0.0200899 -1.1161828 -1.8847524

H -0.3868583 -2.0647846 -1.8888743

C 2.2272150 -1.7445058 -1.1442303

H 3.2935652 -1.6309291 -1.3431780

O 1.8845403 -1.0438294 0.0683093

C 2.7196280 -1.4343623 1.1718823

H 3.7574195 -1.1761856 0.9637366

H 2.0148481 -2.8094890 -1.0067485

H 2.6389334 -2.5079919 1.3488348

H 2.3843400 -0.8933506 2.0514465

SCF energy GEOOPT = -948.7543943335 H

ZPE = 1025. kJ/mol

FREEH energy = 1083.13 kJ/mol

FREEH entropy = 0.64291 kJ/mol/K

$vibrational spectrum

# mode symmetry wave number IR intensity selection rules

# cm**(-1) km/mol IR RAMAN

1 -0.00 0.00000 - -

2 0.00 0.00000 - -

3 0.00 0.00000 - -

4 0.00 0.00000 - -

5 0.00 0.00000 - -

6 0.00 0.00000 - -

7 a 40.82 0.06638 YES YES

8 a 48.23 0.00506 YES YES

9 a 60.03 0.34153 YES YES

10 a 68.78 0.13236 YES YES

11 a 84.04 0.43029 YES YES

12 a 94.74 0.18658 YES YES

13 a 112.60 1.34228 YES YES

14 a 120.46 2.59866 YES YES

15 a 127.51 0.33955 YES YES

16 a 146.94 0.39522 YES YES

17 a 148.71 3.92828 YES YES

18 a 150.99 1.72163 YES YES

19 a 160.73 0.76569 YES YES

20 a 170.38 1.69317 YES YES

21 a 180.71 0.31928 YES YES

22 a 208.70 3.18961 YES YES

23 a 214.90 0.83176 YES YES

24 a 225.90 3.97943 YES YES

25 a 251.89 4.84284 YES YES

26 a 261.28 10.58791 YES YES

27 a 282.53 3.99892 YES YES

28 a 292.38 7.58930 YES YES

29 a 306.55 4.31673 YES YES

30 a 308.36 3.26427 YES YES

31 a 321.17 31.88076 YES YES

32 a 337.13 17.01241 YES YES

33 a 371.84 15.46330 YES YES

34 a 400.65 0.13078 YES YES

35 a 407.37 33.41735 YES YES

36 a 425.74 25.69722 YES YES

37 a 481.86 24.07520 YES YES

38 a 500.37 31.42014 YES YES

39 a 513.14 35.53693 YES YES

40 a 574.34 6.16549 YES YES

41 a 596.70 24.69076 YES YES

42 a 615.04 63.80602 YES YES

43 a 830.06 18.70224 YES YES

44 a 833.93 10.90397 YES YES

45 a 836.49 0.38917 YES YES

46 a 896.64 53.73380 YES YES

47 a 900.62 27.19346 YES YES

48 a 903.15 16.15413 YES YES

49 a 985.51 95.84136 YES YES

50 a 986.63 149.58373 YES YES

51 a 994.46 2.13804 YES YES

52 a 1015.48 15.80920 YES YES

53 a 1024.53 25.49718 YES YES

54 a 1025.72 3.77593 YES YES

55 a 1043.29 29.39745 YES YES

56 a 1047.60 36.49035 YES YES

57 a 1048.56 173.19843 YES YES

58 a 1094.51 116.58924 YES YES

59 a 1095.72 57.33838 YES YES

60 a 1096.94 193.59806 YES YES

61 a 1124.54 67.05666 YES YES

62 a 1126.25 31.27382 YES YES

63 a 1127.96 67.75268 YES YES

64 a 1172.64 1.02604 YES YES

65 a 1174.21 2.17335 YES YES

66 a 1174.37 0.00664 YES YES

67 a 1215.71 2.29964 YES YES

68 a 1217.07 2.32566 YES YES

69 a 1219.31 5.04889 YES YES

70 a 1221.05 4.73196 YES YES

71 a 1222.00 3.29345 YES YES

72 a 1223.53 7.19413 YES YES

73 a 1310.18 7.58590 YES YES

74 a 1312.00 11.37165 YES YES

75 a 1316.90 8.05495 YES YES

76 a 1334.24 14.40137 YES YES

77 a 1337.91 10.52018 YES YES

78 a 1341.32 12.55604 YES YES

79 a 1411.32 6.52537 YES YES

80 a 1413.45 3.29909 YES YES

81 a 1416.57 8.03043 YES YES

82 a 1443.11 0.92969 YES YES

83 a 1446.45 1.26900 YES YES

84 a 1450.73 0.90423 YES YES

85 a 1483.35 16.80457 YES YES

86 a 1484.84 0.42097 YES YES

87 a 1487.08 2.42759 YES YES

88 a 1489.11 11.19396 YES YES

89 a 1490.61 4.06746 YES YES

90 a 1497.94 2.26820 YES YES

91 a 1505.47 10.08140 YES YES

92 a 1507.26 8.33240 YES YES

93 a 1507.89 23.43280 YES YES

94 a 1508.71 2.09168 YES YES

95 a 1510.53 6.44539 YES YES

96 a 1511.49 4.43761 YES YES

97 a 1521.96 17.05759 YES YES

98 a 1522.44 18.99139 YES YES

99 a 1523.39 2.17781 YES YES

100 a 1657.82 48.67290 YES YES

101 a 1658.58 43.51533 YES YES

102 a 1660.09 43.68279 YES YES

103 a 3020.83 14.78634 YES YES

104 a 3022.81 15.12780 YES YES

105 a 3028.48 12.97343 YES YES

106 a 3039.73 15.63040 YES YES

107 a 3042.27 13.61821 YES YES

108 a 3043.76 13.74961 YES YES

109 a 3063.17 5.50878 YES YES

110 a 3064.70 5.33946 YES YES

111 a 3069.05 4.09161 YES YES

112 a 3088.94 6.31480 YES YES

113 a 3090.87 5.94355 YES YES

114 a 3092.79 5.23233 YES YES

115 a 3109.59 9.90306 YES YES

116 a 3112.21 7.30005 YES YES

117 a 3113.50 12.38095 YES YES

118 a 3114.32 4.46804 YES YES

119 a 3115.56 4.04121 YES YES

120 a 3122.26 6.38586 YES YES

121 a 3154.78 16.22335 YES YES

122 a 3161.16 18.09470 YES YES

123 a 3175.64 6.46535 YES YES

124 a 3465.73 12.25912 YES YES

125 a 3470.04 8.75312 YES YES

126 a 3472.37 13.12477 YES YES

127 a 3529.14 14.98634 YES YES

128 a 3530.33 16.79073 YES YES

129 a 3530.99 20.15746 YES YES

$end

Total COSMO energy + OC correction = -948.9540772748 H

SP energy of [Mg(MOEA)_3_]^+^ with the optimized structure for [Mg(MOEA)_3_]^2+^ = -‍948.9537953267 H (No negative HOMO/LUMO gap is found for the radical cation).

**[(Ohfip)_6_Al_2_(OH)_2_Mg(MOEA)]**


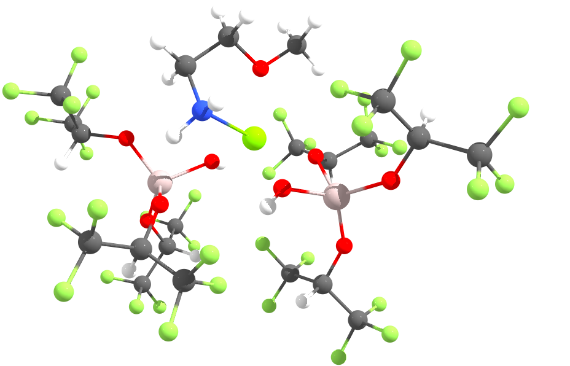


Method: (RI-)B3LYP(D3BJ)/def2-TZVPP

Symmetry: c1

Cartesian coordinates in Ångström:

Al 3.4454696 10.6777967 5.8964397

O 4.6844943 11.3558422 6.9028723

O 4.3403387 9.8184838 4.6102725

O 2.6433186 9.3702020 6.7571815

O 2.2839630 11.8229681 5.3502522

C 1.3613675 9.2931073 7.2404521

F 2.1590207 8.3315817 9.2835244

C 1.5384752 12.0427006 4.2241495

C 4.6452235 12.1769681 8.0007920

F 0.6043776 8.1526564 5.3244870

C 0.6853058 8.0375894 6.6654899

F -0.5473818 7.8358158 7.1305914

F 1.4049050 6.9111026 6.9125279

C 1.3940043 9.3151561 8.7797959

F 0.1748046 9.2134010 9.3245708

F 1.9222020 10.4869728 9.1835981

H 0.7255234 10.1338435 6.9388132

F 3.5067473 12.3756575 2.9774945

C 2.3088087 12.9568118 3.2521381

F 1.6691605 13.1053124 2.0833382

F 2.5612372 14.1670699 3.7522584

F 5.2161731 14.4009012 8.6777643

C 5.2040862 13.5641496 7.6250847

F 6.4513544 13.4982662 7.1359944

F 4.4242347 14.1098809 6.6789128

H 3.6361604 12.3591332 8.3895151

F 4.9160050 10.2815582 9.3837790

C 5.4176391 11.5156396 9.1585456

F 6.7271249 11.3594481 8.8891452

F 5.3117759 12.2052633 10.3012102

F -0.6147234 12.8528272 3.5748202

H 1.3162500 11.1328879 3.6548045

C 0.1698993 12.6129746 4.6362238

F 0.2675892 13.7387409 5.3484729

F -0.4647150 11.6989946 5.4066815

H 4.4697013 10.3146499 3.7984846

F 5.9168756 9.4156431 0.1540393

F 6.4875646 9.2130099 2.2388978

C 5.4773364 9.0560038 1.3589990

F 4.5122508 9.9250110 1.7195134

F 5.6993654 6.9018157 -0.7507145

F 7.0926982 6.6908589 0.8999818

C 5.7899147 6.6483435 0.5567854

C 4.9202326 7.6221259 1.3811013

H 3.9609657 7.6648511 0.8566356

F 1.9428690 8.9517547 2.7743712

O 4.7650939 7.1756983 2.6936616

F 5.3635864 5.3907617 0.7625707

C 1.0956747 8.4542327 1.8386283

F 1.6543941 8.6978027 0.6426576

F -0.0393845 9.1558519 1.9222586

O 3.4028215 6.6633803 4.7429787

Al 3.4297626 5.9954942 3.1178172

H 2.7949836 6.4856420 5.4598494

F 5.9871786 5.0266502 4.7640237

O 2.1022871 6.3045910 2.0802319

H 5.7208721 3.6056100 2.6325658

C 0.8959864 6.9510519 2.1095013

H 0.3891995 6.8940406 3.0796589

C 4.8897913 3.4914956 3.3406533

O 3.8557988 4.3398270 3.0884651

C 5.4989131 3.7490479 4.7335244

F 6.5296917 2.9446360 5.0142396

C -0.0531981 6.2775030 1.0993507

F 3.9122271 1.8771469 1.9347414

C 4.3961164 2.0388670 3.1724952

F 5.4000097 1.1570769 3.3369943

F 0.4353457 6.2912899 -0.1471373

F -1.2577586 6.8743776 1.0819754

F 4.6101398 3.6588456 5.7280782

F -0.2374381 4.9976508 1.4571406

F 3.4259458 1.7270704 4.0421964

H 4.4006793 8.3954415 7.3279200

H 5.1498155 6.9511888 7.2465522

N 5.1647600 7.8962433 6.8761501

H 6.7711459 8.4874242 8.1807141

C 6.4656702 8.5402070 7.1347221

H 6.3692933 9.5918407 6.8730232

Mg 4.9210318 7.9423939 4.7201954

C 7.5101504 7.8720379 6.2711290

H 7.7289007 6.8561713 6.6159635

O 7.0045240 7.7833925 4.9261518

H 8.4371023 8.4482793 6.2690382

C 7.9748474 7.2324255 4.0260525

H 7.4840865 7.0564524 3.0805035

H 8.3515597 6.2899333 4.4246068

H 8.7952886 7.9386236 3.8951382

SCF energy GEOOPT = -5822.553849920 H

ZPE = 1245. kJ/mol

FREEH energy = 1440.90 kJ/mol

FREEH entropy = 1.79888 kJ/mol/K

$vibrational spectrum

# mode symmetry wave number IR intensity selection rules

# cm**(-1) km/mol IR RAMAN

1 -0.00 0.00000 - -

2 -0.00 0.00000 - -

3 0.00 0.00000 - -

4 0.00 0.00000 - -

5 0.00 0.00000 - -

6 0.00 0.00000 - -

7 a 5.63 0.04476 YES YES

8 a 9.42 0.29971 YES YES

9 a 11.10 0.18814 YES YES

10 a 13.34 0.15784 YES YES

11 a 16.38 0.14081 YES YES

12 a 17.39 0.07984 YES YES

13 a 17.72 0.14812 YES YES

14 a 21.52 0.27065 YES YES

15 a 22.34 0.73928 YES YES

16 a 24.69 0.61114 YES YES

17 a 25.71 1.12096 YES YES

18 a 29.60 0.37032 YES YES

19 a 30.52 0.51372 YES YES

20 a 32.06 0.18985 YES YES

21 a 34.17 0.36294 YES YES

22 a 35.36 0.16208 YES YES

23 a 37.63 0.03669 YES YES

24 a 38.24 0.13437 YES YES

25 a 39.30 0.09028 YES YES

26 a 41.83 0.55948 YES YES

27 a 43.92 0.26570 YES YES

28 a 44.93 0.47100 YES YES

29 a 48.40 0.22921 YES YES

30 a 52.95 0.28132 YES YES

31 a 55.94 1.29503 YES YES

32 a 57.51 1.20746 YES YES

33 a 60.63 0.42816 YES YES

34 a 61.34 0.05777 YES YES

35 a 66.27 1.09481 YES YES

36 a 68.57 0.27458 YES YES

37 a 72.56 0.18423 YES YES

38 a 74.79 0.41590 YES YES

39 a 78.24 1.23631 YES YES

40 a 85.01 0.15831 YES YES

41 a 86.07 0.32392 YES YES

42 a 91.84 1.73357 YES YES

43 a 95.41 2.40744 YES YES

44 a 97.08 0.69281 YES YES

45 a 107.34 5.72344 YES YES

46 a 108.45 2.03225 YES YES

47 a 113.40 1.82248 YES YES

48 a 120.91 3.99497 YES YES

49 a 128.58 4.43666 YES YES

50 a 133.87 1.43026 YES YES

51 a 143.25 4.02299 YES YES

52 a 147.74 4.26101 YES YES

53 a 154.62 4.43477 YES YES

54 a 157.15 0.12253 YES YES

55 a 162.29 1.75413 YES YES

56 a 163.83 0.83375 YES YES

57 a 165.72 2.27616 YES YES

58 a 175.82 0.87181 YES YES

59 a 177.56 2.69024 YES YES

60 a 181.50 0.48295 YES YES

61 a 183.32 1.40734 YES YES

62 a 190.36 4.23941 YES YES

63 a 201.60 2.57937 YES YES

64 a 203.70 1.10693 YES YES

65 a 227.88 8.72093 YES YES

66 a 234.95 2.04883 YES YES

67 a 245.67 7.48867 YES YES

68 a 250.48 2.47118 YES YES

69 a 252.92 0.29921 YES YES

70 a 255.79 4.92352 YES YES

71 a 258.42 3.77313 YES YES

72 a 270.69 8.22607 YES YES

73 a 275.36 3.46515 YES YES

74 a 284.36 7.86849 YES YES

75 a 287.60 0.93290 YES YES

76 a 290.25 3.82880 YES YES

77 a 291.60 0.76677 YES YES

78 a 292.08 3.00148 YES YES

79 a 293.85 0.12048 YES YES

80 a 294.72 0.29228 YES YES

81 a 295.06 4.34039 YES YES

82 a 295.81 0.78801 YES YES

83 a 296.79 8.08849 YES YES

84 a 306.02 5.22103 YES YES

85 a 309.98 5.56578 YES YES

86 a 315.28 18.14938 YES YES

87 a 322.49 3.89971 YES YES

88 a 323.67 3.07636 YES YES

89 a 324.56 1.60057 YES YES

90 a 325.85 1.87551 YES YES

91 a 328.47 1.68721 YES YES

92 a 328.79 4.50810 YES YES

93 a 331.44 15.89975 YES YES

94 a 348.66 3.47238 YES YES

95 a 351.50 9.56812 YES YES

96 a 355.08 33.65026 YES YES

97 a 363.09 66.39301 YES YES

98 a 370.78 15.91123 YES YES

99 a 376.88 26.46183 YES YES

100 a 383.90 68.65780 YES YES

101 a 386.28 40.95246 YES YES

102 a 393.84 3.84475 YES YES

103 a 419.95 39.04195 YES YES

104 a 424.05 2.44095 YES YES

105 a 426.50 69.83156 YES YES

106 a 431.09 14.28738 YES YES

107 a 443.55 67.20432 YES YES

108 a 463.49 38.52357 YES YES

109 a 487.12 51.05365 YES YES

110 a 509.47 35.48244 YES YES

111 a 512.74 4.46575 YES YES

112 a 513.15 7.48922 YES YES

113 a 514.29 6.81015 YES YES

114 a 515.32 19.18705 YES YES

115 a 516.49 7.68527 YES YES

116 a 520.79 15.82506 YES YES

117 a 529.22 2.71087 YES YES

118 a 529.77 2.02941 YES YES

119 a 530.09 2.76112 YES YES

120 a 530.56 3.83257 YES YES

121 a 530.66 2.60172 YES YES

122 a 533.02 4.44630 YES YES

123 a 535.72 2.43005 YES YES

124 a 537.79 3.15136 YES YES

125 a 543.11 0.96834 YES YES

126 a 544.65 0.31212 YES YES

127 a 545.80 6.67765 YES YES

128 a 546.12 1.75214 YES YES

129 a 546.36 0.09752 YES YES

130 a 547.61 10.15555 YES YES

131 a 550.56 11.59265 YES YES

132 a 563.45 4.31518 YES YES

133 a 567.94 16.56752 YES YES

134 a 569.70 34.95993 YES YES

135 a 570.60 81.87663 YES YES

136 a 651.43 46.62042 YES YES

137 a 676.63 59.38429 YES YES

138 a 680.40 43.70732 YES YES

139 a 682.95 86.98672 YES YES

140 a 683.62 13.82514 YES YES

141 a 684.28 74.81892 YES YES

142 a 684.50 7.96762 YES YES

143 a 684.74 56.86217 YES YES

144 a 693.96 41.58825 YES YES

145 a 697.37 37.40784 YES YES

146 a 717.76 14.88040 YES YES

147 a 721.60 31.75107 YES YES

148 a 724.50 24.24785 YES YES

149 a 726.23 18.57307 YES YES

150 a 735.34 43.46186 YES YES

151 a 754.43 22.29032 YES YES

152 a 775.20 36.05282 YES YES

153 a 800.24 71.71811 YES YES

154 a 808.15 57.73257 YES YES

155 a 827.33 25.22058 YES YES

156 a 834.36 48.28204 YES YES

157 a 837.48 131.99565 YES YES

158 a 848.79 29.33030 YES YES

159 a 849.89 30.76128 YES YES

160 a 851.94 81.93697 YES YES

161 a 856.61 82.35018 YES YES

162 a 857.34 16.22693 YES YES

163 a 858.46 52.55203 YES YES

164 a 859.67 207.02646 YES YES

165 a 887.01 52.62740 YES YES

166 a 888.30 53.57455 YES YES

167 a 890.49 44.91985 YES YES

168 a 892.60 44.06403 YES YES

169 a 894.32 104.70896 YES YES

170 a 898.85 111.93919 YES YES

171 a 918.37 9.27707 YES YES

172 a 965.63 358.30261 YES YES

173 a 995.98 122.70940 YES YES

174 a 1023.21 17.21338 YES YES

175 a 1061.54 163.73560 YES YES

176 a 1064.47 77.91024 YES YES

177 a 1071.50 244.89617 YES YES

178 a 1073.80 100.65131 YES YES

179 a 1078.15 14.43266 YES YES

180 a 1082.10 129.63261 YES YES

181 a 1089.17 312.01371 YES YES

182 a 1104.37 70.98665 YES YES

183 a 1106.95 96.61016 YES YES

184 a 1141.52 50.72603 YES YES

185 a 1144.47 82.75577 YES YES

186 a 1146.35 27.11178 YES YES

187 a 1148.19 24.28773 YES YES

188 a 1148.84 0.11432 YES YES

189 a 1160.87 29.80150 YES YES

190 a 1167.34 128.46097 YES YES

191 a 1170.00 46.45654 YES YES

192 a 1173.19 148.75002 YES YES

193 a 1182.80 40.02897 YES YES

194 a 1182.91 164.31758 YES YES

195 a 1184.74 64.38187 YES YES

196 a 1190.68 224.66006 YES YES

197 a 1191.78 235.34923 YES YES

198 a 1193.66 27.84929 YES YES

199 a 1195.33 147.85671 YES YES

200 a 1199.23 274.14833 YES YES

201 a 1201.27 545.13350 YES YES

202 a 1205.09 353.45971 YES YES

203 a 1214.58 667.36125 YES YES

204 a 1220.01 174.33870 YES YES

205 a 1222.70 1033.08530 YES YES

206 a 1224.39 41.77433 YES YES

207 a 1231.04 11.18212 YES YES

208 a 1231.38 1074.61247 YES YES

209 a 1245.28 883.51069 YES YES

210 a 1247.92 9.74917 YES YES

211 a 1249.96 87.97469 YES YES

212 a 1251.03 264.75006 YES YES

213 a 1254.72 209.29457 YES YES

214 a 1256.84 271.95794 YES YES

215 a 1261.63 43.78934 YES YES

216 a 1266.27 143.57807 YES YES

217 a 1284.52 77.90786 YES YES

218 a 1288.54 79.39199 YES YES

219 a 1291.26 129.66389 YES YES

220 a 1302.13 121.83750 YES YES

221 a 1307.64 59.56540 YES YES

222 a 1308.07 116.47050 YES YES

223 a 1312.94 180.72462 YES YES

224 a 1335.68 8.51561 YES YES

225 a 1378.79 56.87238 YES YES

226 a 1379.68 34.55760 YES YES

227 a 1382.91 96.53466 YES YES

228 a 1388.33 53.86114 YES YES

229 a 1389.34 10.14808 YES YES

230 a 1392.34 7.97284 YES YES

231 a 1395.08 25.34881 YES YES

232 a 1397.30 29.11661 YES YES

233 a 1400.35 8.11898 YES YES

234 a 1402.63 10.38092 YES YES

235 a 1403.36 14.85601 YES YES

236 a 1403.45 15.26373 YES YES

237 a 1414.87 6.63630 YES YES

238 a 1450.19 6.35384 YES YES

239 a 1490.31 0.55660 YES YES

240 a 1500.82 10.12838 YES YES

241 a 1503.16 10.54810 YES YES

242 a 1520.48 9.70943 YES YES

243 a 1525.63 4.92930 YES YES

244 a 1677.69 28.72403 YES YES

245 a 3003.93 23.74426 YES YES

246 a 3012.58 25.99979 YES YES

247 a 3017.19 16.01052 YES YES

248 a 3024.96 15.55223 YES YES

249 a 3028.41 13.18250 YES YES

250 a 3030.69 11.29965 YES YES

251 a 3041.45 32.85735 YES YES

252 a 3052.73 11.49754 YES YES

253 a 3058.60 15.28642 YES YES

254 a 3073.49 25.34275 YES YES

255 a 3103.53 17.48126 YES YES

256 a 3122.69 5.43064 YES YES

257 a 3231.48 0.59980 YES YES

258 a 3441.97 138.16637 YES YES

259 a 3524.47 52.63191 YES YES

260 a 3862.94 147.20780 YES YES

261 a 3907.85 239.92031 YES YES

$end

Total COSMO energy + OC correction = -5822.5735146300 H

**[MOEA(H_2_O)]**


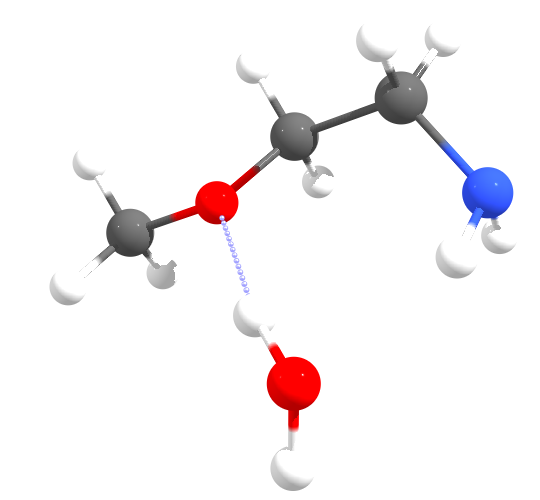


Method: (RI-)B3LYP(D3BJ)/def2-TZVPP

Symmetry: c1

Cartesian coordinates in Ångström:

C -2.1793264 0.6686869 -0.1539338

O -0.7988469 0.8812605 -0.3737814

H -2.7163581 1.4787683 -0.6436386

H -2.4162385 0.6720471 0.9161235

H -2.5028036 -0.2859403 -0.5822344

C 0.0268828 -0.1176940 0.2142139

H -0.1514063 -0.1471805 1.2983374

C 1.4937313 0.1753638 -0.0725498

H -0.2474776 -1.0979038 -0.1985772

H 1.6240253 0.2634485 -1.1533822

H 2.0631245 -0.7053914 0.2390401

N 2.0702638 1.3545200 0.5526405

H 1.6602193 2.2075215 0.1890004

O 0.1258623 3.5106127 -0.7914527

H -0.2734473 2.6317238 -0.6782606

H -0.2785479 4.0641252 -0.1192554

H 1.9395432 1.3427516 1.5563804

SCF energy GEOOPT = -326.1039230736 H

ZPE = 394.8 kJ/mol

FREEH energy = 420.53 kJ/mol

FREEH entropy = 0.40259 kJ/mol/K

$vibrational spectrum

# mode symmetry wave number IR intensity selection rules

# cm**(-1) km/mol IR RAMAN

1 -0.00 0.00000 - -

2 -0.00 0.00000 - -

3 -0.00 0.00000 - -

4 0.00 0.00000 - -

5 0.00 0.00000 - -

6 0.00 0.00000 - -

7 a 37.42 4.79008 YES YES

8 a 87.05 4.81931 YES YES

9 a 96.04 9.06235 YES YES

10 a 157.32 4.70168 YES YES

11 a 167.98 2.22874 YES YES

12 a 188.80 82.86364 YES YES

13 a 226.83 2.62201 YES YES

14 a 278.80 5.78882 YES YES

15 a 328.62 55.54128 YES YES

16 a 358.18 48.89530 YES YES

17 a 370.32 19.37251 YES YES

18 a 525.48 4.80088 YES YES

19 a 664.81 142.33766 YES YES

20 a 812.79 32.76563 YES YES

21 a 855.24 136.71131 YES YES

22 a 906.40 23.33902 YES YES

23 a 998.93 16.45336 YES YES

24 a 1053.74 59.12413 YES YES

25 a 1127.33 36.53249 YES YES

26 a 1141.42 105.90852 YES YES

27 a 1178.05 3.36144 YES YES

28 a 1197.20 8.61600 YES YES

29 a 1241.40 16.38154 YES YES

30 a 1293.63 6.13083 YES YES

31 a 1388.75 7.00900 YES YES

32 a 1397.99 6.16571 YES YES

33 a 1412.98 8.09739 YES YES

34 a 1475.83 1.73863 YES YES

35 a 1479.46 1.85714 YES YES

36 a 1487.91 7.35041 YES YES

37 a 1500.77 10.51234 YES YES

38 a 1516.57 2.58500 YES YES

39 a 1648.17 39.31056 YES YES

40 a 1680.89 20.13372 YES YES

41 a 2956.41 43.20519 YES YES

42 a 2985.96 68.29714 YES YES

43 a 2989.19 50.00020 YES YES

44 a 3022.30 63.55738 YES YES

45 a 3033.40 43.79171 YES YES

46 a 3066.50 27.49221 YES YES

47 a 3114.53 21.16366 YES YES

48 a 3481.93 31.57046 YES YES

49 a 3566.52 17.32682 YES YES

50 a 3627.47 447.10878 YES YES

51 a 3874.91 72.64294 YES YES

$end

Total COSMO energy + OC correction = -326.1171417577 H

**[(H_2_O)∙∙∙H-MOEA]^+^**


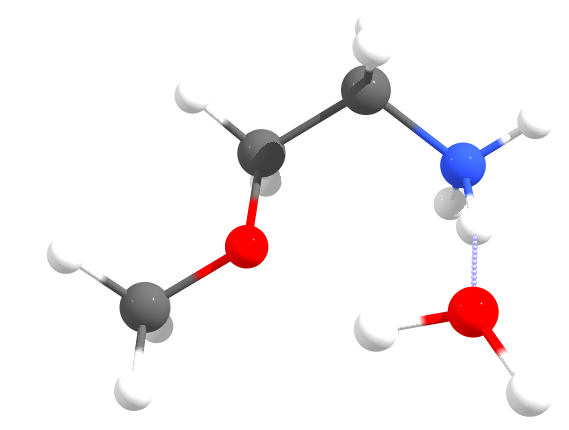


Method: (RI-)B3LYP(D3BJ)/def2-TZVPP

Symmetry: c1

Cartesian coordinates in Ångström:

C -2.2186802 0.5623956 0.0309057

O -0.8377867 0.8868561 -0.1629422

H -2.7949442 1.4423996 -0.2401924

H -2.4123931 0.3086483 1.0759783

H -2.5093480 -0.2738429 -0.6085845

C 0.0256443 -0.1780304 0.1663224

H -0.0906883 -0.4538007 1.2240728

C 1.4532829 0.2296154 -0.1230711

H -0.1969731 -1.0705258 -0.4290551

H 1.5855945 0.4890550 -1.1710968

H 2.1364244 -0.5750352 0.1395194

N 1.8380176 1.4553197 0.6684597

H 1.3306704 2.2981156 0.2795611

H 2.8392195 1.6348235 0.6010523

O 0.5015947 3.3083973 -0.6933843

H -0.3421976 2.8606572 -0.8353504

H 0.3660721 4.2565102 -0.7782331

H 1.6049307 1.3499613 1.6557483

SCF energy GEOOPT = -326.4901206580 H

ZPE = 433.3 kJ/mol

FREEH energy = 458.72 kJ/mol

FREEH entropy = 0.39675 kJ/mol/K

$vibrational spectrum

# mode symmetry wave number IR intensity selection rules

# cm**(-1) km/mol IR RAMAN

1 -0.00 0.00000 - -

2 -0.00 0.00000 - -

3 -0.00 0.00000 - -

4 -0.00 0.00000 - -

5 -0.00 0.00000 - -

6 0.00 0.00000 - -

7 a 50.43 1.68890 YES YES

8 a 93.61 13.41646 YES YES

9 a 101.48 12.90488 YES YES

10 a 132.65 6.22017 YES YES

11 a 197.97 145.41460 YES YES

12 a 210.02 4.13008 YES YES

13 a 277.21 1.34482 YES YES

14 a 320.71 23.04354 YES YES

15 a 333.74 86.77851 YES YES

16 a 354.46 9.07419 YES YES

17 a 384.72 109.37720 YES YES

18 a 470.83 60.77514 YES YES

19 a 530.44 14.39602 YES YES

20 a 824.21 7.01468 YES YES

21 a 862.35 2.24931 YES YES

22 a 976.75 2.12728 YES YES

23 a 988.31 37.84978 YES YES

24 a 1030.93 42.78243 YES YES

25 a 1127.49 174.64431 YES YES

26 a 1146.64 1.37353 YES YES

27 a 1168.92 4.44860 YES YES

28 a 1193.48 3.39669 YES YES

29 a 1235.67 13.82965 YES YES

30 a 1294.96 4.83154 YES YES

31 a 1351.59 11.11133 YES YES

32 a 1407.72 10.27377 YES YES

33 a 1440.96 4.52704 YES YES

34 a 1487.05 2.11408 YES YES

35 a 1490.21 12.36430 YES YES

36 a 1494.79 14.26576 YES YES

37 a 1503.05 10.58483 YES YES

38 a 1519.95 5.32502 YES YES

39 a 1573.02 103.86712 YES YES

40 a 1588.24 75.19031 YES YES

41 a 1662.64 3.32434 YES YES

42 a 1697.73 51.21741 YES YES

43 a 2831.45 1102.23715 YES YES

44 a 2971.17 38.37175 YES YES

45 a 3018.30 28.75472 YES YES

46 a 3026.39 15.14351 YES YES

47 a 3081.90 20.77840 YES YES

48 a 3092.46 0.78510 YES YES

49 a 3145.22 9.80824 YES YES

50 a 3147.88 0.21167 YES YES

51 a 3449.10 60.08468 YES YES

52 a 3507.66 72.98721 YES YES

53 a 3760.29 71.14123 YES YES

54 a 3882.01 207.29656 YES YES

$end

Total COSMO energy + OC correction = -326.5661702379 H

**[Mg(MOEA)_2_(TfO)_2_]**


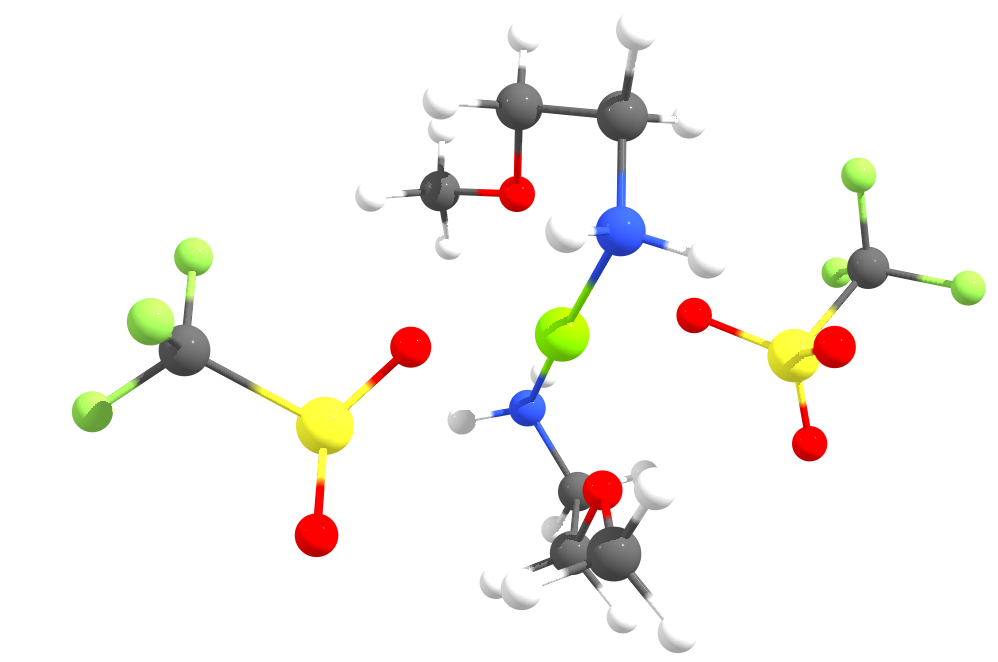


Method: (RI-)B3LYP(D3BJ)/def2-TZVPP

Symmetry: c1

Cartesian coordinates in Ångström:

C -5.5991215 -0.3784371 -0.2211645

O -6.3970722 0.5038872 0.5695006

H -4.6358709 0.0869880 -0.4377426

H -6.1131815 -0.6331468 -1.1482376

H -5.4403306 -1.2820359 0.3597935

C -6.6626671 1.7493461 -0.0863564

H -5.7202412 2.2752126 -0.2589786

C -7.5739448 2.5660766 0.8062857

H -7.1348864 1.5451122 -1.0532673

H -7.0788543 2.7504165 1.7580204

N -8.8095675 1.8112410 1.0873624

H -7.7659866 3.5316854 0.3321897

Mg -8.1569235 -0.2055401 1.5681656

H -9.4498597 1.8510720 0.3032980

H -9.2689843 2.1839092 1.9164963

H -11.6167714 -1.0821290 1.2916586

C -11.2425091 -0.5608050 2.1730679

H -11.8784331 -0.7547354 3.0377772

H -10.1198669 -2.9807118 2.0075206

H -11.2106327 0.5102485 1.9975432

H -7.4925250 -2.8090198 1.3704313

C -9.8036655 -2.3536360 2.8456408

O -9.9062863 -0.9774038 2.4605187

N -7.4498537 -2.1752715 2.1676779

H -10.4623501 -2.5361862 3.6973228

C -8.3661380 -2.6186047 3.2442744

H -8.2542456 -3.6814481 3.4701631

H -6.4999084 -2.1716996 2.5187717

H -8.1342444 -2.0454025 4.1394676

F -6.7340797 3.1223751 4.4528923

F -6.1326049 1.5063309 5.7727930

C -7.1852062 2.1866960 5.3076805

F -7.7809649 2.8039328 6.3287799

S -8.3786324 1.0529347 4.4475579

O -7.5155757 0.5141581 3.3662130

O -9.4135894 1.9365317 3.9433245

O -8.7582422 0.0749308 5.4308194

F -9.8427313 -0.8382469 -3.0414485

F -9.3453236 -2.9321974 -3.3258228

C -9.0338832 -1.8287827 -2.6480935

F -7.7717813 -1.4821729 -2.9563601

S -9.1983512 -2.1193196 -0.8212739

O -10.5861014 -2.4341323 -0.6049536

O -8.7994187 -0.7939274 -0.2887451

O -8.2283221 -3.1537728 -0.5207940

SCF energy GEOOPT = -2622.677886838 H

ZPE = 838.2 kJ/mol

FREEH energy = 921.38 kJ/mol

FREEH entropy = 0.86673 kJ/mol/K

$vibrational spectrum

# mode symmetry wave number IR intensity selection rules

# cm**(-1) km/mol IR RAMAN

1 -0.00 0.00000 - -

2 -0.00 0.00000 - -

3 -0.00 0.00000 - -

4 0.00 0.00000 - -

5 0.00 0.00000 - -

6 0.00 0.00000 - -

7 a 13.93 0.40660 YES YES

8 a 25.25 0.57673 YES YES

9 a 25.60 1.87812 YES YES

10 a 40.10 1.20052 YES YES

11 a 44.54 0.84812 YES YES

12 a 47.06 0.84830 YES YES

13 a 57.38 1.66395 YES YES

14 a 59.21 2.65054 YES YES

15 a 77.06 1.78173 YES YES

16 a 84.80 1.93503 YES YES

17 a 91.25 0.38747 YES YES

18 a 93.63 6.36224 YES YES

19 a 98.49 2.58292 YES YES

20 a 111.35 7.30694 YES YES

21 a 120.81 1.98145 YES YES

22 a 137.41 1.79937 YES YES

23 a 145.20 4.57458 YES YES

24 a 150.81 0.97773 YES YES

25 a 157.45 2.03464 YES YES

26 a 166.92 3.82975 YES YES

27 a 172.09 12.72573 YES YES

28 a 178.81 3.22137 YES YES

29 a 202.96 0.48305 YES YES

30 a 206.45 1.05894 YES YES

31 a 214.68 4.34707 YES YES

32 a 225.40 4.95054 YES YES

33 a 241.96 10.58468 YES YES

34 a 246.31 14.13653 YES YES

35 a 268.16 11.22168 YES YES

36 a 290.26 10.19160 YES YES

37 a 294.29 14.06885 YES YES

38 a 300.52 3.37790 YES YES

39 a 313.02 0.48151 YES YES

40 a 320.37 4.41847 YES YES

41 a 322.82 9.63703 YES YES

42 a 333.96 30.79319 YES YES

43 a 346.50 5.95963 YES YES

44 a 346.82 3.63484 YES YES

45 a 378.09 3.41957 YES YES

46 a 380.68 20.84505 YES YES

47 a 408.41 3.12585 YES YES

48 a 423.86 55.98861 YES YES

49 a 455.43 33.92548 YES YES

50 a 506.63 11.25424 YES YES

51 a 513.41 39.79551 YES YES

52 a 513.97 10.08597 YES YES

53 a 518.07 23.50662 YES YES

54 a 528.08 9.34895 YES YES

55 a 533.92 56.54494 YES YES

56 a 568.10 8.64146 YES YES

57 a 569.35 17.40950 YES YES

58 a 577.46 2.88133 YES YES

59 a 583.55 8.98331 YES YES

60 a 618.44 102.01758 YES YES

61 a 630.98 341.23439 YES YES

62 a 653.52 93.54186 YES YES

63 a 657.21 14.34962 YES YES

64 a 756.94 3.51247 YES YES

65 a 758.50 5.30001 YES YES

66 a 835.56 7.02652 YES YES

67 a 847.77 7.47371 YES YES

68 a 903.47 19.64993 YES YES

69 a 905.90 25.84870 YES YES

70 a 983.35 153.23972 YES YES

71 a 998.45 87.82800 YES YES

72 a 1016.82 487.23338 YES YES

73 a 1026.28 116.46062 YES YES

74 a 1034.70 15.40899 YES YES

75 a 1047.71 12.42584 YES YES

76 a 1068.54 17.46864 YES YES

77 a 1071.97 29.65025 YES YES

78 a 1095.09 82.65097 YES YES

79 a 1105.21 180.94426 YES YES

80 a 1112.49 37.73087 YES YES

81 a 1117.23 50.29109 YES YES

82 a 1154.67 232.64423 YES YES

83 a 1155.07 177.95185 YES YES

84 a 1175.46 35.52868 YES YES

85 a 1179.54 32.25953 YES YES

86 a 1180.78 10.42946 YES YES

87 a 1186.83 2.37692 YES YES

88 a 1198.75 248.55951 YES YES

89 a 1199.37 250.32693 YES YES

90 a 1219.33 7.72462 YES YES

91 a 1229.68 328.86704 YES YES

92 a 1230.52 30.57333 YES YES

93 a 1233.96 126.67451 YES YES

94 a 1236.18 19.38549 YES YES

95 a 1240.35 90.21274 YES YES

96 a 1309.26 15.20829 YES YES

97 a 1313.86 43.18497 YES YES

98 a 1326.59 283.01941 YES YES

99 a 1329.80 289.36967 YES YES

100 a 1344.56 7.41867 YES YES

101 a 1346.43 15.83019 YES YES

102 a 1411.46 5.45383 YES YES

103 a 1415.08 11.75663 YES YES

104 a 1442.14 2.50143 YES YES

105 a 1449.26 2.87677 YES YES

106 a 1485.30 0.30862 YES YES

107 a 1486.27 0.35622 YES YES

108 a 1490.81 12.87917 YES YES

109 a 1492.91 8.86612 YES YES

110 a 1501.08 7.06638 YES YES

111 a 1502.59 4.08958 YES YES

112 a 1506.28 6.36525 YES YES

113 a 1512.24 2.86399 YES YES

114 a 1517.43 7.52251 YES YES

115 a 1526.25 8.36671 YES YES

116 a 1657.14 39.96490 YES YES

117 a 1659.31 13.97934 YES YES

118 a 3008.11 20.09612 YES YES

119 a 3026.03 8.41463 YES YES

120 a 3033.16 38.55067 YES YES

121 a 3035.83 34.81609 YES YES

122 a 3037.22 38.62731 YES YES

123 a 3041.67 45.96128 YES YES

124 a 3057.40 38.18861 YES YES

125 a 3072.67 17.26784 YES YES

126 a 3103.57 14.09518 YES YES

127 a 3106.60 9.08621 YES YES

128 a 3114.06 3.37700 YES YES

129 a 3119.77 0.80933 YES YES

130 a 3148.97 13.26188 YES YES

131 a 3150.77 13.08519 YES YES

132 a 3425.18 101.87459 YES YES

133 a 3431.87 133.90926 YES YES

134 a 3551.13 36.18043 YES YES

135 a 3552.30 34.71341 YES YES

$end

Total COSMO energy + OC correction = -2622.7026948607 H

**[Mg(MOEA)_2_(Ohfip)_2_]**


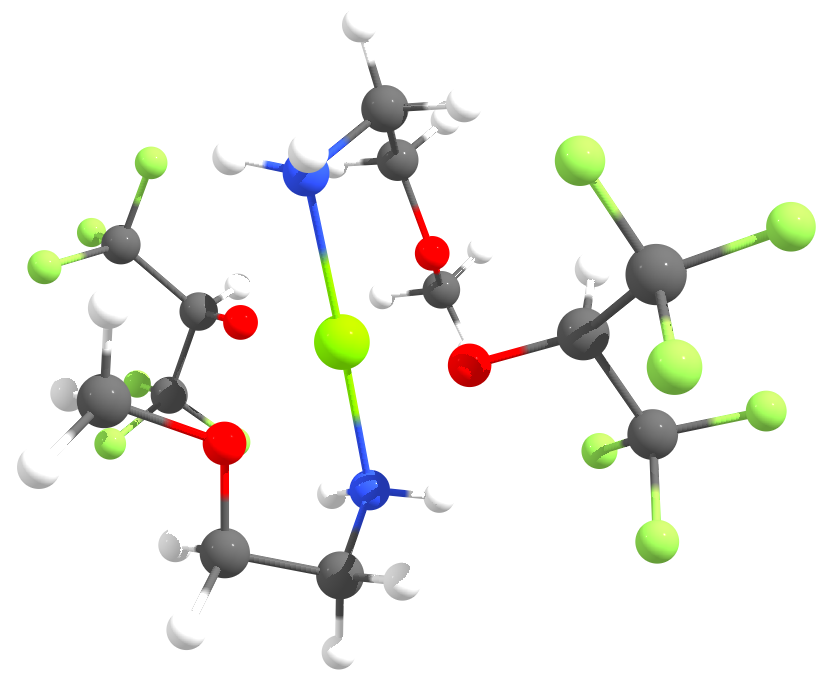


Method: (RI-)B3LYP(D3BJ)/def2-TZVPP

Symmetry: c1

Cartesian coordinates in Ångström:

F -1.4132835 -1.6100315 -2.4979913

F 0.3653533 -1.4143288 -0.3030244

C -2.2280329 -2.1766066 -1.5881464

C -0.6467173 -1.9387182 0.4117560

F -2.0521966 -3.5069598 -1.6589178

F -0.4214692 -3.2591728 0.5271617

O -2.2476405 -0.2790209 -0.1549251

C -2.0417180 -1.5937824 -0.1663233

F -3.4992415 -1.9284778 -2.0001232

F -0.5503621 -1.4153871 1.6616054

H -2.7395071 -2.1920594 0.4562339

Mg -2.9530378 1.5293029 -0.0202746

F -2.9247816 4.9899274 2.1899247

F -3.6595170 6.1447907 0.5062494

C -4.0295167 5.2694076 1.4507170

F -4.8894997 5.9009993 2.2775612

O -3.6693620 3.3273586 0.1323436

C -4.5912854 3.9489716 0.8770963

H -4.9375734 3.3897738 1.7689000

C -5.8873353 4.1801720 0.0658504

F -5.6855492 4.8529401 -1.0771441

F -6.8585176 4.8137362 0.7473454

F -6.4019255 2.9641960 -0.2878915

H -5.8440527 -0.3021846 2.2904858

H -4.0060818 0.4243410 3.6750980

H -1.7891996 1.4521750 3.8260778

H -1.1743713 2.7534575 1.7146006

C -3.3998502 0.5331310 2.7707620

C -2.4023781 1.6600717 2.9446833

C -5.2356829 -0.1227141 1.4008145

N -1.5992071 1.8345343 1.7211427

O -4.2460360 0.8612419 1.6684323

H -5.8695025 0.2685403 0.6094995

H -0.8706847 1.1319130 1.6679917

H -2.9381373 2.5901907 3.1235762

H -2.8899670 -0.4131477 2.5690550

H -4.7749293 -1.0580072 1.0749416

H -1.9526235 0.8574298 -3.1746018

H -4.0930634 2.0025006 -3.8035896

H -4.2685879 0.2828122 -2.1174437

H -0.0570348 0.7641926 -1.6844878

C -3.6585701 2.1366793 -2.8087384

C -2.1671668 1.8931385 -2.8912638

N -4.2547275 1.2443066 -1.7970090

O -1.5973777 2.1537917 -1.6074972

C -0.2119506 1.8344920 -1.5461540

H -1.7139511 2.5595123 -3.6309768

H -3.8388718 3.1594037 -2.4822738

H -5.2041947 1.5364359 -1.6023270

H 0.1441683 2.1268944 -0.5617731

H 0.3363088 2.3987471 -2.3044181

SCF energy GEOOPT = -2278.148782472 H

ZPE = 946.0 kJ/mol

FREEH energy = 1039.85 kJ/mol

FREEH entropy = 0.96871 kJ/mol/K

$vibrational spectrum

# mode symmetry wave number IR intensity selection rules

# cm**(-1) km/mol IR RAMAN

1 -0.00 0.00000 - -

2 0.00 0.00000 - -

3 0.00 0.00000 - -

4 0.00 0.00000 - -

5 0.00 0.00000 - -

6 0.00 0.00000 - -

7 a 6.28 0.08916 YES YES

8 a 17.72 0.88192 YES YES

9 a 22.75 0.05584 YES YES

10 a 26.39 0.01463 YES YES

11 a 33.89 0.12374 YES YES

12 a 37.90 1.98952 YES YES

13 a 41.77 0.92429 YES YES

14 a 42.74 0.03143 YES YES

15 a 55.22 2.97891 YES YES

16 a 62.61 2.23073 YES YES

17 a 68.16 0.53338 YES YES

18 a 73.53 1.39773 YES YES

19 a 78.23 0.64361 YES YES

20 a 84.28 5.19052 YES YES

21 a 93.44 1.05448 YES YES

22 a 109.63 10.96241 YES YES

23 a 115.55 6.31933 YES YES

24 a 117.81 5.23698 YES YES

25 a 133.27 4.29657 YES YES

26 a 147.50 3.59591 YES YES

27 a 149.80 4.60621 YES YES

28 a 159.16 0.75182 YES YES

29 a 166.94 1.19859 YES YES

30 a 168.95 0.37266 YES YES

31 a 173.30 3.12849 YES YES

32 a 185.24 1.32303 YES YES

33 a 208.51 1.62048 YES YES

34 a 227.55 4.96936 YES YES

35 a 244.18 5.70649 YES YES

36 a 256.59 1.00219 YES YES

37 a 261.95 2.34607 YES YES

38 a 270.76 6.22153 YES YES

39 a 273.95 11.47519 YES YES

40 a 286.47 7.24365 YES YES

41 a 290.49 0.17939 YES YES

42 a 292.53 0.36953 YES YES

43 a 299.30 4.29770 YES YES

44 a 303.04 1.52126 YES YES

45 a 310.79 54.23818 YES YES

46 a 320.87 7.04212 YES YES

47 a 323.01 11.66545 YES YES

48 a 344.57 6.11215 YES YES

49 a 348.58 2.77740 YES YES

50 a 361.66 27.54575 YES YES

51 a 374.27 8.73462 YES YES

52 a 393.11 31.49321 YES YES

53 a 411.56 42.51609 YES YES

54 a 490.81 20.34420 YES YES

55 a 496.21 2.49097 YES YES

56 a 501.78 23.56530 YES YES

57 a 510.23 6.52272 YES YES

58 a 511.51 8.77388 YES YES

59 a 524.23 2.47911 YES YES

60 a 525.22 2.31231 YES YES

61 a 533.53 41.00326 YES YES

62 a 540.25 0.39475 YES YES

63 a 540.67 0.70946 YES YES

64 a 583.62 39.04067 YES YES

65 a 599.28 98.14330 YES YES

66 a 635.98 2.40433 YES YES

67 a 661.39 20.73654 YES YES

68 a 678.91 31.07894 YES YES

69 a 680.68 37.89753 YES YES

70 a 735.16 11.42736 YES YES

71 a 738.05 43.91854 YES YES

72 a 833.85 15.99897 YES YES

73 a 839.95 36.23556 YES YES

74 a 841.56 9.45325 YES YES

75 a 849.84 61.54267 YES YES

76 a 869.00 38.90329 YES YES

77 a 875.98 62.16715 YES YES

78 a 900.10 51.66168 YES YES

79 a 901.64 27.21850 YES YES

80 a 961.98 195.70589 YES YES

81 a 971.22 71.60560 YES YES

82 a 1027.76 19.55872 YES YES

83 a 1030.27 6.91909 YES YES

84 a 1048.23 82.66369 YES YES

85 a 1054.33 182.73790 YES YES

86 a 1063.36 9.93622 YES YES

87 a 1075.38 13.79176 YES YES

88 a 1083.77 95.42158 YES YES

89 a 1085.14 18.82659 YES YES

90 a 1120.67 59.82995 YES YES

91 a 1121.94 17.63335 YES YES

92 a 1126.54 108.73102 YES YES

93 a 1132.60 30.72505 YES YES

94 a 1149.59 273.90558 YES YES

95 a 1156.18 187.97273 YES YES

96 a 1176.47 5.52063 YES YES

97 a 1177.39 153.45658 YES YES

98 a 1179.40 271.78848 YES YES

99 a 1181.76 437.88193 YES YES

100 a 1216.94 8.06138 YES YES

101 a 1219.91 7.26557 YES YES

102 a 1233.29 19.06478 YES YES

103 a 1235.85 19.26315 YES YES

104 a 1241.38 127.13055 YES YES

105 a 1243.33 569.71441 YES YES

106 a 1252.50 143.20667 YES YES

107 a 1256.95 172.49120 YES YES

108 a 1283.27 148.12150 YES YES

109 a 1297.53 238.69771 YES YES

110 a 1310.07 7.29277 YES YES

111 a 1313.69 9.35922 YES YES

112 a 1336.98 4.65523 YES YES

113 a 1345.45 7.01473 YES YES

114 a 1364.85 23.22359 YES YES

115 a 1370.57 12.08360 YES YES

116 a 1380.62 14.12171 YES YES

117 a 1385.35 15.34676 YES YES

118 a 1410.43 5.10964 YES YES

119 a 1413.86 11.25944 YES YES

120 a 1440.80 4.22169 YES YES

121 a 1448.89 2.58166 YES YES

122 a 1479.26 0.01830 YES YES

123 a 1483.30 0.20859 YES YES

124 a 1492.47 11.22153 YES YES

125 a 1493.99 8.62622 YES YES

126 a 1494.92 4.30762 YES YES

127 a 1497.22 7.62496 YES YES

128 a 1500.51 1.39970 YES YES

129 a 1501.35 6.92095 YES YES

130 a 1512.59 8.94287 YES YES

131 a 1518.02 4.99947 YES YES

132 a 1645.15 33.80788 YES YES

133 a 1652.40 23.73132 YES YES

134 a 2850.42 87.84459 YES YES

135 a 2878.49 70.76217 YES YES

136 a 3007.98 8.55706 YES YES

137 a 3011.27 7.28422 YES YES

138 a 3018.22 49.13873 YES YES

139 a 3022.21 50.76384 YES YES

140 a 3025.26 80.32835 YES YES

141 a 3028.58 58.79379 YES YES

142 a 3049.46 31.92585 YES YES

143 a 3056.34 26.42849 YES YES

144 a 3076.23 28.50385 YES YES

145 a 3095.91 16.75174 YES YES

146 a 3112.10 2.45974 YES YES

147 a 3114.03 8.63253 YES YES

148 a 3136.12 19.08362 YES YES

149 a 3137.78 13.82330 YES YES

150 a 3496.98 4.94559 YES YES

151 a 3500.65 6.97269 YES YES

152 a 3581.75 17.60303 YES YES

153 a 3582.49 41.40971 YES YES

$end

Total COSMO energy + OC correction = -2278.1637881143 H

**[Mg(TfO)_2_]**


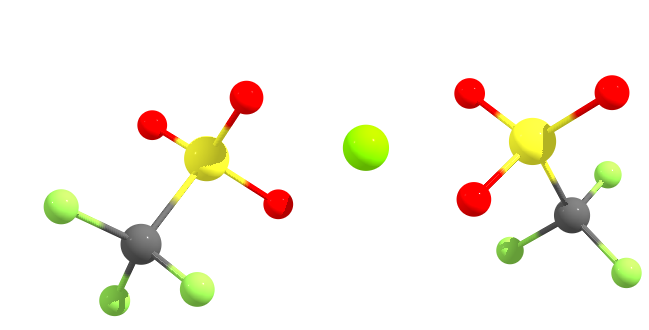


Method: (RI-)B3LYP(D3BJ)/def2-TZVPP

Symmetry: c2

Cartesian coordinates in Ångström:

O 1.4782719 -1.1015693 1.4544686

F 2.6501751 -1.1592010 -1.2378028

O -0.0174756 -1.8291938 -0.2080205

F -3.6878356 2.6139786 -0.0010670

S 1.1534243 -2.2848508 0.6011077

C 2.5501523 -2.3464743 -0.6290212

Mg 0.0000000 0.0000000 0.6392655

F 3.6878356 -2.6139786 -0.0010670

F 2.3061265 -3.2841658 -1.5356308

C -2.5501523 2.3464743 -0.6290212

F -2.3061265 3.2841658 -1.5356308

O 1.0802852 -3.5586542 1.2246507

F -2.6501751 1.1592010 -1.2378028

O -1.0802852 3.5586542 1.2246507

S -1.1534243 2.2848508 0.6011077

O -1.4782719 1.1015693 1.4544686

O 0.0174756 1.8291938 -0.2080205

SCF energy GEOOPT = -2123.212660231 H

ZPE = 155.4 kJ/mol

FREEH energy = 199.99 kJ/mol

FREEH entropy = 0.58261 kJ/mol/K

$vibrational spectrum

# mode symmetry wave number IR intensity selection rules

# cm**(-1) km/mol IR RAMAN

1 -0.00 0.00000 - -

2 -0.00 0.00000 - -

3 -0.00 0.00000 - -

4 -0.00 0.00000 - -

5 -0.00 0.00000 - -

6 0.00 0.00000 - -

7 a 18.91 0.01243 YES YES

8 a 25.50 1.37169 YES YES

9 b 27.05 1.66270 YES YES

10 a 47.96 0.02188 YES YES

11 b 48.44 0.10930 YES YES

12 a 98.28 10.30268 YES YES

13 b 102.21 10.70495 YES YES

14 a 166.06 0.38929 YES YES

15 b 180.87 0.28430 YES YES

16 a 184.38 0.05471 YES YES

17 b 217.89 8.78658 YES YES

18 a 221.09 0.37483 YES YES

19 b 301.20 0.54064 YES YES

20 a 304.94 0.04716 YES YES

21 b 313.18 6.45513 YES YES

22 a 314.93 6.56698 YES YES

23 b 328.36 1.10654 YES YES

24 a 328.93 0.98178 YES YES

25 b 338.48 9.79082 YES YES

26 a 373.79 0.96074 YES YES

27 b 511.72 11.05212 YES YES

28 a 512.00 14.23527 YES YES

29 b 516.81 63.78931 YES YES

30 a 546.77 1.18664 YES YES

31 b 565.76 84.45348 YES YES

32 a 571.31 4.02232 YES YES

33 b 571.41 8.39269 YES YES

34 a 608.45 71.34689 YES YES

35 b 631.15 141.12515 YES YES

36 b 662.18 309.58151 YES YES

37 a 672.04 47.51971 YES YES

38 b 766.76 0.79430 YES YES

39 a 766.79 0.64287 YES YES

40 b 981.00 383.60465 YES YES

41 a 990.42 115.32166 YES YES

42 a 1097.20 182.35209 YES YES

43 b 1097.23 186.93934 YES YES

44 a 1179.38 101.39049 YES YES

45 b 1179.78 269.39911 YES YES

46 b 1199.93 266.46319 YES YES

47 a 1200.90 19.23676 YES YES

48 b 1220.05 305.68575 YES YES

49 a 1220.39 297.97847 YES YES

50 b 1384.46 577.37722 YES YES

51 a 1391.85 45.13758 YES YES

$end

Total COSMO energy + OC correction = -2123.2295960988 H

**[Mg(MOEA)(TfO)_2_]**


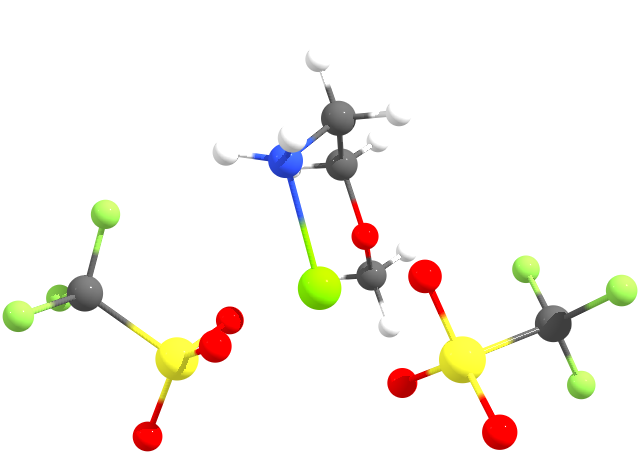


Method: (RI-)B3LYP(D3BJ)/def2-TZVPP

Symmetry: c1

Cartesian coordinates in Ångström:

Mg -7.9905944 -0.1315580 1.5278496

H -11.4375161 -0.1046880 1.3638400

C -10.9597052 0.1454008 2.3120126

H -11.6442774 -0.0272586 3.1433218

H -10.4455762 -2.3859369 1.6027662

H -10.6422585 1.1831805 2.3027020

H -7.8493366 -2.7572787 0.8967717

C -10.0077763 -2.0505065 2.5496537

O -9.7781225 -0.6441070 2.4919008

N -7.7058463 -2.2642935 1.7711305

H -10.7063973 -2.2774712 3.3583292

C -8.6656246 -2.7078633 2.8037449

H -8.7862559 -3.7927946 2.8323179

H -6.7524259 -2.4475830 2.0625323

H -8.2850921 -2.3797669 3.7700707

F -9.0128557 0.8725524 4.9273186

F -8.4844252 2.9736505 5.0467957

C -7.9933970 1.7447383 4.8945984

F -7.1733336 1.4719922 5.9090909

S -7.0716838 1.6215553 3.2821074

O -8.1109749 1.8396865 2.2492425

O -6.7072381 0.1882115 3.1841492

O -6.0162025 2.5733452 3.3805068

F -8.7487866 -1.5477084 -2.9469578

F -7.7011130 -2.5102034 -1.3079943

C -7.7168621 -1.4243509 -2.1160887

F -6.5868442 -1.4139951 -2.8144681

S -7.8785817 0.1340255 -1.1116205

O -9.0937477 -0.0979248 -0.2960408

O -7.9082256 1.1880449 -2.0694988

O -6.7434230 0.0766353 -0.1664942

SCF energy GEOOPT = -2372.953587972 H

ZPE = 496.0 kJ/mol

FREEH energy = 560.10 kJ/mol

FREEH entropy = 0.72554 kJ/mol/K

$vibrational spectrum

# mode symmetry wave number IR intensity selection rules

# cm**(-1) km/mol IR RAMAN

1 -0.00 0.00000 - -

2 -0.00 0.00000 - -

3 -0.00 0.00000 - -

4 -0.00 0.00000 - -

5 -0.00 0.00000 - -

6 0.00 0.00000 - -

7 a 25.38 0.32462 YES YES

8 a 29.97 0.33658 YES YES

9 a 35.25 0.52717 YES YES

10 a 37.52 1.51539 YES YES

11 a 40.80 0.95270 YES YES

12 a 50.87 1.60814 YES YES

13 a 64.45 0.43881 YES YES

14 a 73.55 0.67707 YES YES

15 a 79.32 0.56249 YES YES

16 a 110.56 4.48688 YES YES

17 a 122.66 3.08465 YES YES

18 a 151.13 4.55616 YES YES

19 a 157.88 0.60775 YES YES

20 a 167.09 0.14252 YES YES

21 a 176.86 2.52582 YES YES

22 a 206.25 2.47196 YES YES

23 a 211.92 5.33491 YES YES

24 a 215.47 2.03513 YES YES

25 a 220.67 2.82665 YES YES

26 a 224.86 0.52930 YES YES

27 a 268.03 3.75863 YES YES

28 a 286.69 5.53710 YES YES

29 a 294.64 8.94179 YES YES

30 a 308.45 1.10052 YES YES

31 a 321.37 18.35165 YES YES

32 a 332.07 5.65552 YES YES

33 a 333.04 6.62005 YES YES

34 a 334.79 15.46120 YES YES

35 a 359.35 6.82401 YES YES

36 a 373.66 4.18680 YES YES

37 a 416.36 80.30593 YES YES

38 a 441.96 60.59484 YES YES

39 a 505.31 11.73432 YES YES

40 a 508.83 17.95047 YES YES

41 a 515.30 77.91610 YES YES

42 a 539.76 20.54983 YES YES

43 a 541.28 13.26774 YES YES

44 a 566.66 3.65565 YES YES

45 a 569.50 5.16244 YES YES

46 a 592.96 33.04655 YES YES

47 a 597.17 59.37583 YES YES

48 a 605.76 65.59538 YES YES

49 a 647.91 284.89106 YES YES

50 a 658.14 10.05914 YES YES

51 a 758.11 4.40013 YES YES

52 a 761.13 2.95549 YES YES

53 a 844.51 12.85108 YES YES

54 a 907.76 16.54776 YES YES

55 a 988.92 132.81241 YES YES

56 a 1004.29 405.33499 YES YES

57 a 1010.77 45.64484 YES YES

58 a 1021.48 13.49699 YES YES

59 a 1069.00 25.98342 YES YES

60 a 1087.51 127.10245 YES YES

61 a 1115.65 58.72112 YES YES

62 a 1136.14 147.83874 YES YES

63 a 1142.40 142.04026 YES YES

64 a 1156.89 118.10715 YES YES

65 a 1168.52 269.17768 YES YES

66 a 1181.80 0.50816 YES YES

67 a 1199.28 308.91339 YES YES

68 a 1201.61 96.48966 YES YES

69 a 1205.92 221.91573 YES YES

70 a 1212.29 264.75382 YES YES

71 a 1220.51 7.27594 YES YES

72 a 1233.79 6.10771 YES YES

73 a 1303.78 7.19835 YES YES

74 a 1336.89 6.82192 YES YES

75 a 1370.38 325.71226 YES YES

76 a 1378.00 388.68129 YES YES

77 a 1407.20 8.39734 YES YES

78 a 1436.59 2.01948 YES YES

79 a 1485.17 0.63192 YES YES

80 a 1496.69 10.01878 YES YES

81 a 1499.92 11.64489 YES YES

82 a 1502.78 4.20564 YES YES

83 a 1519.84 8.62767 YES YES

84 a 1654.51 38.71952 YES YES

85 a 3005.08 24.03874 YES YES

86 a 3033.25 33.44808 YES YES

87 a 3043.67 22.79443 YES YES

88 a 3061.78 34.52053 YES YES

89 a 3101.90 21.85572 YES YES

90 a 3105.84 8.20180 YES YES

91 a 3162.19 1.69986 YES YES

92 a 3500.98 22.94859 YES YES

93 a 3570.36 35.76710 YES YES

$end

Total COSMO energy + OC correction = -2372.9755897416 H

**[Mg(MOEA)_3_(TfO)_2_]**


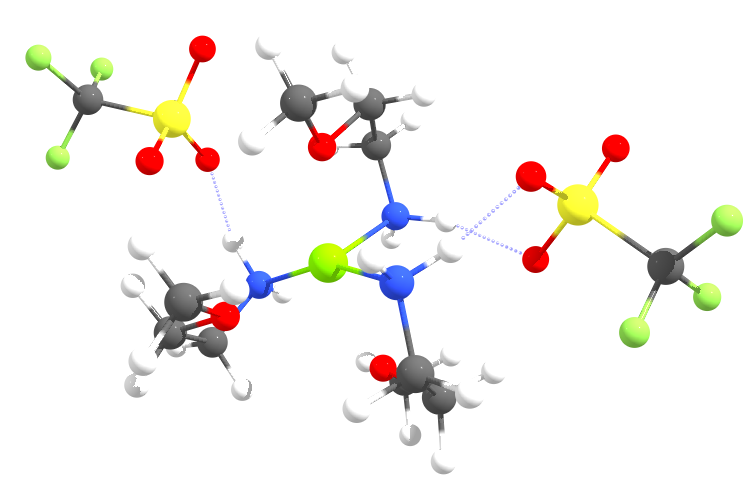


Method: (RI-)B3LYP(D3BJ)/def2-TZVPP

Symmetry: c1

Cartesian coordinates in Ångström:

C -6.9246544 2.4040503 -0.1683104

O -6.4355352 1.7119717 0.9817455

H -6.1378056 3.0402081 -0.5793368

H -7.7925421 3.0059858 0.1010581

H -7.2008423 1.6642491 -0.9132534

C -5.9905644 2.6471548 1.9830883

H -5.1874519 3.2588303 1.5644281

C -5.4952546 1.8611291 3.1734159

H -6.8358638 3.2793490 2.2558292

H -4.5797552 1.3271375 2.9143515

N -6.5226363 0.8761877 3.5455624

H -5.2661539 2.5606623 3.9814463

Mg -7.1521861 -0.2263290 1.7813458

H -7.3777966 1.3429830 3.8890969

H -6.1946540 0.3118120 4.3195902

H -10.1485682 -0.3317264 -0.3075134

C -9.9684231 0.4394874 0.4454627

H -10.9075637 0.8568875 0.8029634

H -10.1412689 -1.9813613 1.5240887

H -9.3704100 1.2475488 0.0411821

H -7.7551745 -2.7241862 2.5785935

C -10.0117122 -1.1446072 2.2146142

O -9.2491296 -0.1201498 1.5476680

N -7.8412546 -1.8386028 3.0862265

H -10.9793981 -0.7274804 2.4929275

C -9.2472479 -1.5520381 3.4511530

H -9.7410124 -2.4135855 3.9054324

H -7.2999350 -1.9648189 3.9327854

H -9.2572114 -0.7265766 4.1618424

F -9.1428392 4.7373089 4.5179033

F -10.9514282 3.8518776 5.3301537

C -10.2637672 4.0627497 4.2029720

F -11.0001572 4.8380693 3.3976766

S -9.8386159 2.4583467 3.3663981

O -9.0296984 1.7720852 4.3796918

O -9.0681991 2.8823162 2.2034640

O -11.1152620 1.8415583 3.0890126

F -6.2863387 -4.4824830 -1.3669460

F -8.0272339 -5.6999960 -1.8198118

C -7.2126961 -5.3165533 -0.8352778

F -6.5705618 -6.3904475 -0.3682347

S -8.1493710 -4.4477749 0.5164373

O -8.7018964 -3.2780495 -0.1715464

O -7.0819796 -4.0847900 1.4554828

O -9.1079916 -5.4048939 0.9932505

H -5.2335366 -0.1579903 -0.8288760

H -7.5682422 -0.4066428 -0.9320910

C -5.6721927 -1.1444623 -0.6738776

H -5.5784968 -1.7128909 -1.6019109

N -7.0727391 -0.9715381 -0.2534769

H -7.5719090 -1.8734988 -0.2552043

C -4.9206663 -1.8758072 0.4138028

H -3.8474407 -1.8796027 0.2079217

O -5.1596254 -1.1911189 1.6589902

C -4.5709560 -1.8877901 2.7544361

H -3.4900966 -1.9604569 2.6164621

H -5.2791576 -2.9008318 0.5109158

H -4.9965197 -2.8893029 2.8414050

H -4.7717791 -1.3169622 3.6571233

SCF energy GEOOPT = -2872.344076904 H

ZPE = 1180. kJ/mol

FREEH energy = 1282.06 kJ/mol

FREEH entropy = 1.00596 kJ/mol/K

$vibrational spectrum

# mode symmetry wave number IR intensity selection rules

# cm**(-1) km/mol IR RAMAN

1 -0.00 0.00000 - -

2 -0.00 0.00000 - -

3 -0.00 0.00000 - -

4 -0.00 0.00000 - -

5 0.00 0.00000 - -

6 0.00 0.00000 - -

7 a 17.18 2.32145 YES YES

8 a 19.72 1.18492 YES YES

9 a 29.73 1.17135 YES YES

10 a 30.88 0.50219 YES YES

11 a 34.38 1.22111 YES YES

12 a 38.57 0.26295 YES YES

13 a 42.39 3.10300 YES YES

14 a 43.19 2.11062 YES YES

15 a 51.11 3.58528 YES YES

16 a 56.79 2.26761 YES YES

17 a 73.18 2.28327 YES YES

18 a 79.78 6.54095 YES YES

19 a 84.67 1.21156 YES YES

20 a 88.46 6.97937 YES YES

21 a 104.00 5.02474 YES YES

22 a 109.38 1.61040 YES YES

23 a 118.60 7.78194 YES YES

24 a 120.97 2.92009 YES YES

25 a 129.00 6.47726 YES YES

26 a 136.81 3.28546 YES YES

27 a 143.73 1.72653 YES YES

28 a 151.79 1.62172 YES YES

29 a 168.59 3.40553 YES YES

30 a 174.36 8.85226 YES YES

31 a 175.31 6.46986 YES YES

32 a 182.17 6.96264 YES YES

33 a 193.87 3.02167 YES YES

34 a 196.63 1.29810 YES YES

35 a 199.16 3.48333 YES YES

36 a 205.08 1.87823 YES YES

37 a 214.05 4.13998 YES YES

38 a 217.43 1.61410 YES YES

39 a 222.85 2.57616 YES YES

40 a 237.85 3.05116 YES YES

41 a 248.44 8.40866 YES YES

42 a 260.46 5.93642 YES YES

43 a 269.23 21.57495 YES YES

44 a 284.43 26.76608 YES YES

45 a 300.76 8.51747 YES YES

46 a 304.23 7.14287 YES YES

47 a 309.45 15.24177 YES YES

48 a 310.17 22.34901 YES YES

49 a 316.06 1.81617 YES YES

50 a 323.79 34.47677 YES YES

51 a 328.64 0.68003 YES YES

52 a 342.47 2.77869 YES YES

53 a 342.97 0.40270 YES YES

54 a 346.44 10.96854 YES YES

55 a 357.49 8.48653 YES YES

56 a 361.93 4.92912 YES YES

57 a 390.68 21.12650 YES YES

58 a 413.20 1.06684 YES YES

59 a 428.96 20.57941 YES YES

60 a 445.15 23.75150 YES YES

61 a 510.32 27.78154 YES YES

62 a 512.41 11.97721 YES YES

63 a 514.95 17.69039 YES YES

64 a 516.21 46.07818 YES YES

65 a 530.69 13.83288 YES YES

66 a 543.61 8.61337 YES YES

67 a 544.53 50.71785 YES YES

68 a 568.06 7.72052 YES YES

69 a 569.72 5.94925 YES YES

70 a 573.54 6.03429 YES YES

71 a 574.35 19.87660 YES YES

72 a 603.92 59.60926 YES YES

73 a 632.89 344.33330 YES YES

74 a 635.47 60.96762 YES YES

75 a 645.03 33.94653 YES YES

76 a 663.27 19.50570 YES YES

77 a 751.36 4.62710 YES YES

78 a 753.75 5.17555 YES YES

79 a 846.88 30.63561 YES YES

80 a 851.45 8.24634 YES YES

81 a 852.00 5.69360 YES YES

82 a 915.90 28.37490 YES YES

83 a 918.17 11.31601 YES YES

84 a 922.36 11.74709 YES YES

85 a 999.78 60.55847 YES YES

86 a 1001.39 94.89688 YES YES

87 a 1007.23 4.31487 YES YES

88 a 1018.41 341.03962 YES YES

89 a 1023.77 196.56728 YES YES

90 a 1057.28 5.37122 YES YES

91 a 1065.37 22.03962 YES YES

92 a 1068.79 5.40131 YES YES

93 a 1072.38 90.89596 YES YES

94 a 1079.57 38.45683 YES YES

95 a 1080.39 23.71579 YES YES

96 a 1109.14 69.10756 YES YES

97 a 1112.26 157.20642 YES YES

98 a 1114.12 31.90055 YES YES

99 a 1127.57 207.90049 YES YES

100 a 1134.16 49.56456 YES YES

101 a 1147.24 27.95112 YES YES

102 a 1150.80 82.14804 YES YES

103 a 1152.75 227.24119 YES YES

104 a 1168.09 85.08251 YES YES

105 a 1174.59 31.43332 YES YES

106 a 1182.07 1.08822 YES YES

107 a 1183.49 5.81756 YES YES

108 a 1183.72 0.16845 YES YES

109 a 1208.34 104.79697 YES YES

110 a 1210.34 78.99932 YES YES

111 a 1215.07 264.33587 YES YES

112 a 1222.08 8.13999 YES YES

113 a 1224.55 119.67570 YES YES

114 a 1229.29 353.13475 YES YES

115 a 1233.15 14.05653 YES YES

116 a 1240.09 16.37087 YES YES

117 a 1244.19 9.49542 YES YES

118 a 1245.71 34.03325 YES YES

119 a 1296.99 357.27437 YES YES

120 a 1317.95 21.25378 YES YES

121 a 1318.83 6.12218 YES YES

122 a 1324.73 245.23849 YES YES

123 a 1326.74 137.29718 YES YES

124 a 1348.27 64.06235 YES YES

125 a 1354.51 5.06941 YES YES

126 a 1360.74 10.60357 YES YES

127 a 1419.23 8.13566 YES YES

128 a 1420.31 3.31102 YES YES

129 a 1421.81 8.97814 YES YES

130 a 1449.39 5.61450 YES YES

131 a 1451.31 0.62347 YES YES

132 a 1454.00 2.50827 YES YES

133 a 1480.31 7.59741 YES YES

134 a 1484.39 2.95056 YES YES

135 a 1486.94 6.35546 YES YES

136 a 1487.30 1.60233 YES YES

137 a 1493.34 6.32830 YES YES

138 a 1494.80 2.83570 YES YES

139 a 1504.56 5.59350 YES YES

140 a 1508.60 5.39379 YES YES

141 a 1510.44 2.90272 YES YES

142 a 1511.18 2.86925 YES YES

143 a 1512.15 5.68854 YES YES

144 a 1516.19 5.80616 YES YES

145 a 1518.67 5.29726 YES YES

146 a 1521.57 14.60509 YES YES

147 a 1523.11 9.28034 YES YES

148 a 1682.56 27.30465 YES YES

149 a 1692.31 40.33287 YES YES

150 a 1694.12 39.70281 YES YES

151 a 3022.88 27.87241 YES YES

152 a 3025.13 1.98115 YES YES

153 a 3026.77 30.91793 YES YES

154 a 3028.33 47.26757 YES YES

155 a 3031.81 18.63917 YES YES

156 a 3035.41 93.05712 YES YES

157 a 3039.15 7.13201 YES YES

158 a 3040.80 75.49217 YES YES

159 a 3043.96 28.98700 YES YES

160 a 3069.81 33.39180 YES YES

161 a 3077.68 24.34618 YES YES

162 a 3086.37 18.17763 YES YES

163 a 3096.86 3.62359 YES YES

164 a 3098.81 13.64958 YES YES

165 a 3100.79 6.55838 YES YES

166 a 3103.60 5.50101 YES YES

167 a 3109.19 3.54794 YES YES

168 a 3116.98 5.83052 YES YES

169 a 3135.51 26.80911 YES YES

170 a 3149.43 26.78851 YES YES

171 a 3178.20 5.49787 YES YES

172 a 3195.44 637.08523 YES YES

173 a 3233.99 551.04947 YES YES

174 a 3342.97 473.17288 YES YES

175 a 3537.96 13.66626 YES YES

176 a 3540.63 11.71221 YES YES

177 a 3542.72 11.88367 YES YES

$end

Total COSMO energy + OC correction = -2872.3810899659 H

**[TfO]^−^**


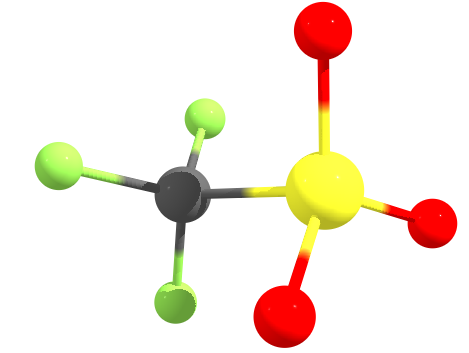


Method: (RI-)B3LYP(D3BJ)/def2-TZVPP

Symmetry: c3v

Cartesian coordinates in Ångström:

F -0.6262086 1.0846250 -1.3566066

F -0.6262086 -1.0846250 -1.3566066

C -0.0000000 0.0000000 -0.8483611

F 1.2524171 0.0000000 -1.3566066

S 0.0000000 0.0000000 1.0217783

O -1.4192266 0.0000000 1.3310388

O 0.7096133 1.2290863 1.3310388

O 0.7096133 -1.2290863 1.3310388

SCF energy GEOOPT = -961.5921348483 H

ZPE = 71.63 kJ/mol

FREEH energy = 90.31 kJ/mol

FREEH entropy = 0.34711 kJ/mol/K

$vibrational spectrum

# mode symmetry wave number IR intensity selection rules

# cm**(-1) km/mol IR RAMAN

1 -0.00 0.00000 - -

2 -0.00 0.00000 - -

3 -0.00 0.00000 - -

4 -0.00 0.00000 - -

5 0.00 0.00000 - -

6 0.00 0.00000 - -

7 a2 63.00 0.00000 NO NO

8 e 195.44 1.00415 YES YES

9 e 195.44 1.00415 YES YES

10 a1 288.39 0.05773 YES YES

11 e 338.02 0.03593 YES YES

12 e 338.02 0.03593 YES YES

13 e 511.24 10.99928 YES YES

14 e 511.24 10.99928 YES YES

15 e 564.89 12.07499 YES YES

16 e 564.89 12.07499 YES YES

17 a1 635.39 170.11608 YES YES

18 a1 735.20 8.95304 YES YES

19 a1 1031.41 173.80236 YES YES

20 e 1113.81 188.61759 YES YES

21 e 1113.81 188.61759 YES YES

22 a1 1199.73 64.65076 YES YES

23 e 1287.83 372.26413 YES YES

24 e 1287.83 372.26413 YES YES

$end

Total COSMO energy + OC correction = -961.6674198200 H

**G2**


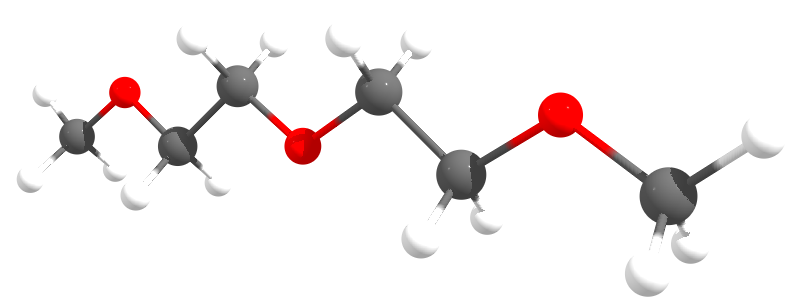


Method: (RI-)B3LYP(D3BJ)/def2-TZVPP

Symmetry: c2v

Cartesian coordinates in Ångström:

C 0.0000000 -4.7101375 -0.2783660

O 0.0000000 -3.5380682 0.5055604

H 0.8900173 -4.7639520 -0.9174622

H -0.8900173 -4.7639520 -0.9174622

H 0.0000000 -5.5583196 0.4033726

C 0.0000000 -2.3589868 -0.2709400

C 0.0000000 -1.1791908 0.6788087

H -0.8864977 -2.3169615 -0.9170251

H 0.8864977 -2.3169615 -0.9170251

O 0.0000000 0.0000000 -0.0987491

H 0.8863792 -1.2201458 1.3239831

H -0.8863792 -1.2201458 1.3239831

C 0.0000000 1.1791908 0.6788087

C 0.0000000 2.3589868 -0.2709400

H 0.8863792 1.2201458 1.3239831

H -0.8863792 1.2201458 1.3239831

H -0.8864977 2.3169615 -0.9170251

H 0.8864977 2.3169615 -0.9170251

O 0.0000000 3.5380682 0.5055604

C 0.0000000 4.7101375 -0.2783660

H 0.0000000 5.5583196 0.4033726

H -0.8900173 4.7639520 -0.9174622

H 0.8900173 4.7639520 -0.9174622

SCF energy GEOOPT = -462.6415740778 H

ZPE = 530.5 kJ/mol

FREEH energy = 561.72 kJ/mol

FREEH entropy = 0.45354 kJ/mol/K

$vibrational spectrum

# mode symmetry wave number IR intensity selection rules

# cm**(-1) km/mol IR RAMAN

1 -0.00 0.00000 - -

2 -0.00 0.00000 - -

3 0.00 0.00000 - -

4 0.00 0.00000 - -

5 0.00 0.00000 - -

6 0.00 0.00000 - -

7 b1 36.73 0.05108 YES YES

8 a2 56.99 0.00000 NO YES

9 a1 69.06 0.09718 YES YES

10 a2 88.54 0.00000 NO YES

11 b1 105.28 9.05023 YES YES

12 a2 137.15 0.00000 NO YES

13 b1 138.49 5.71777 YES YES

14 b2 180.18 5.48128 YES YES

15 a2 229.05 0.00000 NO YES

16 b1 230.09 8.49348 YES YES

17 a1 266.95 0.00002 YES YES

18 a1 314.42 0.66427 YES YES

19 b2 435.91 0.32792 YES YES

20 b2 500.48 0.00003 YES YES

21 a1 534.11 5.06591 YES YES

22 a2 834.71 0.00000 NO YES

23 b1 844.76 0.51897 YES YES

24 a1 966.01 89.59051 YES YES

25 b2 991.20 0.29048 YES YES

26 a1 1043.02 1.01927 YES YES

27 b2 1068.81 1.50998 YES YES

28 a1 1116.71 5.83096 YES YES

29 a2 1149.64 0.00000 NO YES

30 b2 1153.99 103.97383 YES YES

31 b1 1163.54 1.05063 YES YES

32 a1 1165.60 2.59338 YES YES

33 b2 1165.82 489.23671 YES YES

34 a2 1182.97 0.00000 NO YES

35 b1 1190.29 3.34741 YES YES

36 b2 1225.49 84.99511 YES YES

37 a1 1231.46 2.56965 YES YES

38 a2 1231.52 0.00000 NO YES

39 b1 1242.42 19.96030 YES YES

40 a2 1299.15 0.00000 NO YES

41 b1 1299.44 0.04840 YES YES

42 b2 1348.94 34.93052 YES YES

43 a1 1379.69 9.44414 YES YES

44 b2 1432.98 1.95554 YES YES

45 a1 1461.94 0.36043 YES YES

46 b2 1480.16 1.31455 YES YES

47 a1 1482.91 0.00269 YES YES

48 a2 1487.35 0.00000 NO YES

49 b1 1487.36 13.82713 YES YES

50 b2 1504.16 15.60393 YES YES

51 a1 1504.59 1.59846 YES YES

52 b2 1516.35 7.30279 YES YES

53 a1 1526.70 0.72492 YES YES

54 b2 1531.15 4.76531 YES YES

55 a1 1536.33 1.99266 YES YES

56 b2 2965.91 47.27637 YES YES

57 a1 2966.41 0.00350 YES YES

58 b2 2972.87 53.40256 YES YES

59 a1 2976.60 82.32644 YES YES

60 b2 2977.58 1.35166 YES YES

61 a1 2983.72 138.86146 YES YES

62 b1 2990.71 7.63966 YES YES

63 a2 2991.42 0.00000 NO YES

64 a2 3013.29 0.00000 NO YES

65 b1 3013.34 174.95225 YES YES

66 b1 3019.33 133.24711 YES YES

67 a2 3021.00 0.00000 NO YES

68 b2 3112.99 38.74545 YES YES

69 a1 3113.06 20.35561 YES YES

$end

Total COSMO energy + OC correction = -462.6496655211 H

**[Mg(G2)_2_]^2+^**


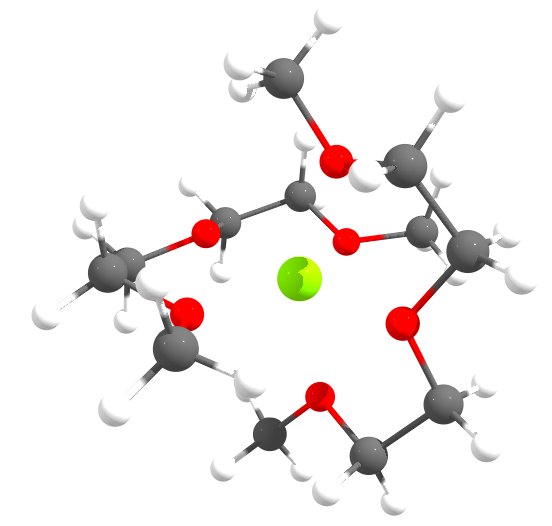


Method: (RI-)B3LYP(D3BJ)/def2-TZVPP

Symmetry: c2

Cartesian coordinates in Ångström:

H 2.9998186 -1.4418697 -0.1498989

H 1.1971347 1.1571890 3.2159179

H -2.9890962 -1.2078965 -1.6631868

H 1.7037310 -1.2250549 2.6256229

H -1.3546412 -2.9955985 -0.7157116

O -0.9048405 -1.1619558 -1.5748676

O 1.5338819 0.3688999 1.3136185

C 0.7742487 -2.7431935 -1.0235274

C 2.3029900 -1.7750023 0.6221921

C -2.1395397 -0.8486982 -2.2450135

C 1.5971594 1.4649481 2.2490345

H -2.1831814 0.2316341 -2.3473523

H 0.9964649 2.2707524 1.8401377

C 2.2394769 -0.8005528 1.7736834

C -0.6641440 -2.5756842 -1.4509073

O 0.9746222 -1.8254284 0.0686191

H 2.6022949 -2.7632790 0.9720491

H 2.6300529 1.7902081 2.3641175

H -2.1468496 -1.3015784 -3.2351927

H 3.2465470 -0.5264626 2.0901980

H -0.8297012 -3.0664400 -2.4106004

H 1.4626321 -2.4927548 -1.8334762

H 0.9657295 -3.7646335 -0.6937780

Mg 0.0000000 0.0000000 -0.0758697

H -2.6300529 -1.7902081 2.3641175

H -0.9964649 -2.2707524 1.8401377

C -1.5971594 -1.4649481 2.2490345

H -2.9998186 1.4418697 -0.1498989

H 2.1831814 -0.2316341 -2.3473523

O -1.5338819 -0.3688999 1.3136185

H -1.4626321 2.4927548 -1.8334762

H 2.1468496 1.3015784 -3.2351927

O 0.9048405 1.1619558 -1.5748676

C 2.1395397 0.8486982 -2.2450135

H -3.2465470 0.5264626 2.0901980

C -2.2394769 0.8005528 1.7736834

C -2.3029900 1.7750023 0.6221921

C -0.7742487 2.7431935 -1.0235274

H 0.8297012 3.0664400 -2.4106004

C 0.6641440 2.5756842 -1.4509073

O -0.9746222 1.8254284 0.0686191

H -1.1971347 -1.1571890 3.2159179

H -2.6022949 2.7632790 0.9720491

H 2.9890962 1.2078965 -1.6631868

H -0.9657295 3.7646335 -0.6937780

H -1.7037310 1.2250549 2.6256229

H 1.3546412 2.9955985 -0.7157116

SCF energy GEOOPT = -1125.037062688 H

ZPE = 1088. kJ/mol

FREEH energy = 1151.99 kJ/mol

FREEH entropy = 0.68456 kJ/mol/K

$vibrational spectrum

# mode symmetry wave number IR intensity selection rules

# cm**(-1) km/mol IR RAMAN

1 -0.00 0.00000 - -

2 -0.00 0.00000 - -

3 -0.00 0.00000 - -

4 0.00 0.00000 - -

5 0.00 0.00000 - -

6 0.00 0.00000 - -

7 a 41.10 0.21948 YES YES

8 b 43.41 0.03717 YES YES

9 a 52.53 0.21106 YES YES

10 a 65.57 0.21357 YES YES

11 b 78.72 0.15901 YES YES

12 b 84.04 0.50801 YES YES

13 a 92.48 0.43801 YES YES

14 a 113.67 0.06900 YES YES

15 b 115.91 3.64690 YES YES

16 a 130.30 3.01451 YES YES

17 b 131.59 0.27620 YES YES

18 a 152.48 1.29898 YES YES

19 b 156.02 3.35981 YES YES

20 a 165.39 1.43443 YES YES

21 b 173.07 0.17958 YES YES

22 a 175.36 0.17038 YES YES

23 b 194.66 0.02743 YES YES

24 a 197.28 0.87035 YES YES

25 b 208.85 6.48842 YES YES

26 a 211.89 9.44357 YES YES

27 b 223.06 1.94918 YES YES

28 a 245.37 0.00138 YES YES

29 b 265.92 1.02075 YES YES

30 a 269.18 0.72082 YES YES

31 b 292.94 1.14156 YES YES

32 a 293.15 1.13238 YES YES

33 b 306.26 13.65279 YES YES

34 a 323.83 55.90228 YES YES

35 b 325.20 51.16604 YES YES

36 b 352.80 3.69289 YES YES

37 a 359.03 0.08236 YES YES

38 a 373.55 0.25519 YES YES

39 b 426.99 33.85016 YES YES

40 a 427.46 32.35908 YES YES

41 b 453.98 58.82091 YES YES

42 a 556.22 0.07927 YES YES

43 b 558.50 3.39087 YES YES

44 a 572.67 1.04408 YES YES

45 b 572.84 0.99477 YES YES

46 b 830.14 8.52186 YES YES

47 a 830.61 8.14478 YES YES

48 b 842.14 22.43925 YES YES

49 a 842.70 20.07339 YES YES

50 b 878.39 107.72677 YES YES

51 a 894.62 0.99572 YES YES

52 a 963.22 16.62555 YES YES

53 b 963.23 20.42939 YES YES

54 b 1015.02 30.90254 YES YES

55 a 1015.21 31.88234 YES YES

56 b 1028.10 5.77166 YES YES

57 a 1033.41 3.67162 YES YES

58 a 1053.66 229.82802 YES YES

59 b 1054.25 251.63065 YES YES

60 a 1069.66 42.51634 YES YES

61 b 1071.26 13.49693 YES YES

62 a 1089.12 0.00020 YES YES

63 b 1095.63 503.33286 YES YES

64 b 1117.05 44.43252 YES YES

65 a 1117.15 26.20342 YES YES

66 a 1122.24 4.31210 YES YES

67 b 1122.37 7.70114 YES YES

68 b 1143.53 7.61980 YES YES

69 a 1143.74 0.11243 YES YES

70 b 1179.06 0.18405 YES YES

71 a 1179.07 1.41347 YES YES

72 b 1182.09 3.50464 YES YES

73 a 1182.75 1.02953 YES YES

74 b 1217.89 6.86579 YES YES

75 a 1218.06 3.27709 YES YES

76 b 1225.16 13.72870 YES YES

77 a 1225.73 2.45119 YES YES

78 b 1260.64 0.15272 YES YES

79 a 1260.78 3.21921 YES YES

80 a 1271.81 20.56469 YES YES

81 b 1272.46 24.08202 YES YES

82 b 1291.77 11.17883 YES YES

83 a 1292.23 7.48213 YES YES

84 b 1305.68 22.25572 YES YES

85 a 1305.96 0.01956 YES YES

86 b 1385.61 13.30640 YES YES

87 a 1385.65 12.76049 YES YES

88 a 1413.94 0.11836 YES YES

89 b 1416.82 9.69020 YES YES

90 a 1428.02 0.71851 YES YES

91 b 1428.06 0.51447 YES YES

92 b 1457.80 0.49684 YES YES

93 a 1458.40 0.01123 YES YES

94 a 1484.37 0.73696 YES YES

95 b 1484.75 0.89456 YES YES

96 a 1484.97 0.53795 YES YES

97 b 1486.66 1.93812 YES YES

98 a 1488.29 11.30388 YES YES

99 b 1488.57 4.04544 YES YES

100 b 1494.24 20.21312 YES YES

101 a 1494.37 12.36018 YES YES

102 a 1501.97 2.59641 YES YES

103 b 1502.40 24.53983 YES YES

104 a 1503.46 1.94596 YES YES

105 b 1505.53 12.42065 YES YES

106 b 1509.07 13.30145 YES YES

107 a 1510.77 9.17795 YES YES

108 b 1516.41 0.16961 YES YES

109 a 1518.59 0.32595 YES YES

110 b 1519.09 14.30249 YES YES

111 a 1521.36 26.51658 YES YES

112 b 1524.69 22.70204 YES YES

113 a 1525.83 0.29449 YES YES

114 a 3039.75 0.02514 YES YES

115 b 3039.76 10.95009 YES YES

116 a 3040.52 0.08160 YES YES

117 b 3041.50 23.09011 YES YES

118 b 3042.91 5.59610 YES YES

119 a 3042.91 9.83502 YES YES

120 b 3043.09 16.06423 YES YES

121 a 3044.46 13.30192 YES YES

122 b 3045.67 0.83809 YES YES

123 a 3045.94 0.52394 YES YES

124 a 3047.77 29.73894 YES YES

125 b 3047.88 33.01605 YES YES

126 b 3095.47 9.27971 YES YES

127 a 3095.52 0.44848 YES YES

128 b 3096.27 2.94921 YES YES

129 a 3096.33 2.29701 YES YES

130 b 3105.53 20.00948 YES YES

131 a 3105.59 19.94641 YES YES

132 b 3107.80 0.03505 YES YES

133 a 3107.94 0.48037 YES YES

134 b 3117.84 9.26118 YES YES

135 a 3118.23 2.33566 YES YES

136 b 3121.04 2.46271 YES YES

137 a 3121.23 8.31562 YES YES

138 a 3153.72 0.09954 YES YES

139 b 3153.79 19.94684 YES YES

140 a 3165.62 2.05242 YES YES

141 b 3165.74 8.71432 YES YES

$end

Total COSMO energy + OC correction = -1125.2248579199 H

SP energy of [Mg(G2)_2_]^+^ with the optimized structure for [Mg(G2)_2_]^2+^ = -‍1125.19776684326 H (A very small negative HOMO/LUMO gap of 0.01 eV is found in the radical cation).

**[Mg(G2)(TfO)_2_]**


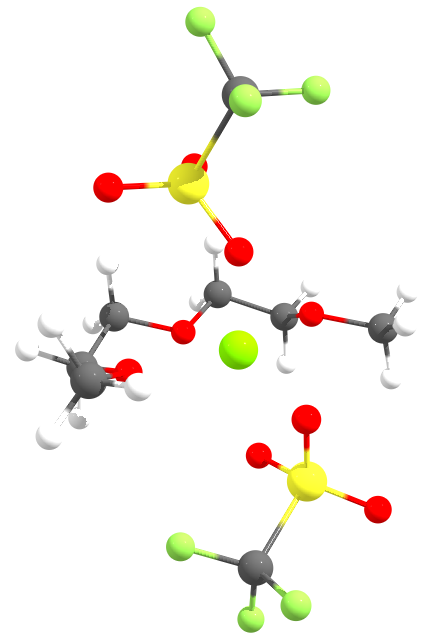


Method: (RI-)B3LYP(D3BJ)/def2-TZVPP

Symmetry: c1

Cartesian coordinates in Ångström:

Mg -7.7883360 -0.1985047 2.2213359

F -9.0783459 2.7215503 4.1667636

F -7.4686681 4.1706414 4.0233407

C -7.8032687 2.9724337 4.4981243

F -7.7056477 2.9851404 5.8271293

S -6.6836938 1.6655086 3.7941672

O -6.9592474 1.6986914 2.3361965

O -7.2370923 0.3931777 4.2883368

O -5.3646462 2.0258195 4.2104190

F -8.8098869 -1.6964509 -3.1350919

F -6.8714143 -1.1612544 -2.3171238

C -8.1849165 -0.9170809 -2.2489532

F -8.3975911 0.3605462 -2.5771180

S -8.8240198 -1.2681264 -0.5420328

O -8.4950678 -2.6525931 -0.2852893

O -10.2245325 -0.9250397 -0.5705259

O -8.0057871 -0.3251767 0.2640566

H -4.4386237 -0.5951861 3.2024519

H -6.0750858 -2.3295342 4.0687449

H -10.6136855 -0.7024782 4.1648411

H -11.2066207 1.6773089 2.5939380

C -4.7396372 -0.8979554 2.1981973

C -6.2275582 -2.5952632 3.0189869

C -10.5776658 -0.8989441 3.0913276

C -10.5774977 1.1885772 1.8496867

O -8.5006774 -2.0050669 3.0248560

O -6.0704288 -1.4261875 2.2115230

H -4.7506738 -0.0305138 1.5453060

H -10.2676016 -2.9981759 3.4720795

O -9.8189254 0.1400861 2.4673682

H -7.8531725 -3.9245816 3.4807692

C -9.8976691 -2.2227977 2.7970101

C -7.6267184 -3.1154859 2.7827386

H -11.5909850 -0.9141813 2.6898953

H -5.4948961 -3.3496093 2.7232036

H -9.8591726 1.9011162 1.4557556

H -4.0529259 -1.6488149 1.8054279

H -11.1675236 0.7771251 1.0327004

H -10.0532544 -2.5318437 1.7630090

H -7.7548590 -3.4525962 1.7536079

SCF energy GEOOPT = -2585.948413051 H

ZPE = 698.1 kJ/mol

FREEH energy = 775.40 kJ/mol

FREEH entropy = 0.83070 kJ/mol/K

$vibrational spectrum

# mode symmetry wave number IR intensity selection rules

# cm**(-1) km/mol IR RAMAN

1 -0.00 0.00000 - -

2 0.00 0.00000 - -

3 0.00 0.00000 - -

4 0.00 0.00000 - -

5 0.00 0.00000 - -

6 0.00 0.00000 - -

7 a 10.22 0.89404 YES YES

8 a 25.57 0.21836 YES YES

9 a 28.94 0.71076 YES YES

10 a 33.35 1.19730 YES YES

11 a 46.40 0.67046 YES YES

12 a 47.89 0.45948 YES YES

13 a 54.83 1.35921 YES YES

14 a 64.18 1.63367 YES YES

15 a 78.91 2.14962 YES YES

16 a 85.54 2.36367 YES YES

17 a 90.89 4.59473 YES YES

18 a 108.97 3.29414 YES YES

19 a 113.18 8.76911 YES YES

20 a 117.56 4.65843 YES YES

21 a 132.42 0.43313 YES YES

22 a 140.16 1.72424 YES YES

23 a 151.54 1.38173 YES YES

24 a 159.84 1.76194 YES YES

25 a 175.10 0.18831 YES YES

26 a 194.87 2.39447 YES YES

27 a 204.20 2.01744 YES YES

28 a 206.63 0.59700 YES YES

29 a 213.06 1.31823 YES YES

30 a 214.81 6.57248 YES YES

31 a 231.69 5.65567 YES YES

32 a 246.29 34.15056 YES YES

33 a 266.00 2.11980 YES YES

34 a 285.52 10.86946 YES YES

35 a 297.14 10.20577 YES YES

36 a 307.38 3.50278 YES YES

37 a 321.04 13.35742 YES YES

38 a 321.83 23.29237 YES YES

39 a 331.90 35.35824 YES YES

40 a 334.91 3.03412 YES YES

41 a 344.61 2.57310 YES YES

42 a 363.36 4.53879 YES YES

43 a 374.48 7.66217 YES YES

44 a 416.52 36.54746 YES YES

45 a 433.29 27.24831 YES YES

46 a 453.40 124.02889 YES YES

47 a 509.04 9.39048 YES YES

48 a 510.31 21.03600 YES YES

49 a 519.11 3.04639 YES YES

50 a 540.06 15.86911 YES YES

51 a 549.69 2.61280 YES YES

52 a 566.02 0.65301 YES YES

53 a 567.83 5.32200 YES YES

54 a 569.79 6.35655 YES YES

55 a 587.95 2.93293 YES YES

56 a 596.83 45.96781 YES YES

57 a 643.02 194.89700 YES YES

58 a 653.02 155.36973 YES YES

59 a 761.33 2.86402 YES YES

60 a 762.66 3.73890 YES YES

61 a 831.58 5.25604 YES YES

62 a 852.98 20.67972 YES YES

63 a 890.42 35.61704 YES YES

64 a 960.78 10.04576 YES YES

65 a 1009.98 336.12391 YES YES

66 a 1022.36 116.47045 YES YES

67 a 1024.97 143.94016 YES YES

68 a 1035.11 63.32491 YES YES

69 a 1078.08 183.29828 YES YES

70 a 1084.04 12.60940 YES YES

71 a 1105.97 179.44697 YES YES

72 a 1127.25 36.85335 YES YES

73 a 1140.09 17.07086 YES YES

74 a 1156.54 62.87998 YES YES

75 a 1160.16 17.35576 YES YES

76 a 1170.37 228.24241 YES YES

77 a 1173.28 44.21962 YES YES

78 a 1176.54 163.14180 YES YES

79 a 1186.36 4.25391 YES YES

80 a 1190.54 2.84688 YES YES

81 a 1199.98 196.88436 YES YES

82 a 1203.03 292.20545 YES YES

83 a 1205.43 257.18521 YES YES

84 a 1226.32 4.08987 YES YES

85 a 1233.61 181.51698 YES YES

86 a 1237.52 118.09364 YES YES

87 a 1264.77 0.26942 YES YES

88 a 1276.97 19.24106 YES YES

89 a 1299.83 2.56051 YES YES

90 a 1312.00 40.06633 YES YES

91 a 1324.67 295.97948 YES YES

92 a 1355.71 325.57566 YES YES

93 a 1387.62 21.45378 YES YES

94 a 1413.46 7.04614 YES YES

95 a 1423.53 1.89390 YES YES

96 a 1460.42 0.66493 YES YES

97 a 1485.05 2.28923 YES YES

98 a 1486.43 0.57308 YES YES

99 a 1494.04 15.81934 YES YES

100 a 1494.33 7.33481 YES YES

101 a 1499.56 9.62720 YES YES

102 a 1500.82 17.04553 YES YES

103 a 1506.87 7.00363 YES YES

104 a 1508.66 0.76192 YES YES

105 a 1515.21 10.16036 YES YES

106 a 1530.68 7.59404 YES YES

107 a 3023.81 10.98058 YES YES

108 a 3031.60 33.03985 YES YES

109 a 3035.77 10.90932 YES YES

110 a 3039.06 3.00685 YES YES

111 a 3040.83 79.81610 YES YES

112 a 3044.37 52.23137 YES YES

113 a 3071.69 20.89410 YES YES

114 a 3090.78 5.15251 YES YES

115 a 3099.64 19.84607 YES YES

116 a 3103.66 2.14364 YES YES

117 a 3105.64 10.62284 YES YES

118 a 3122.70 8.46665 YES YES

119 a 3155.57 3.93559 YES YES

120 a 3157.12 5.23871 YES YES

$end

Total COSMO energy + OC correction = -2585.9701649150 H

**[Mg(MOEA)_3_(H_2_O)]^2+^**


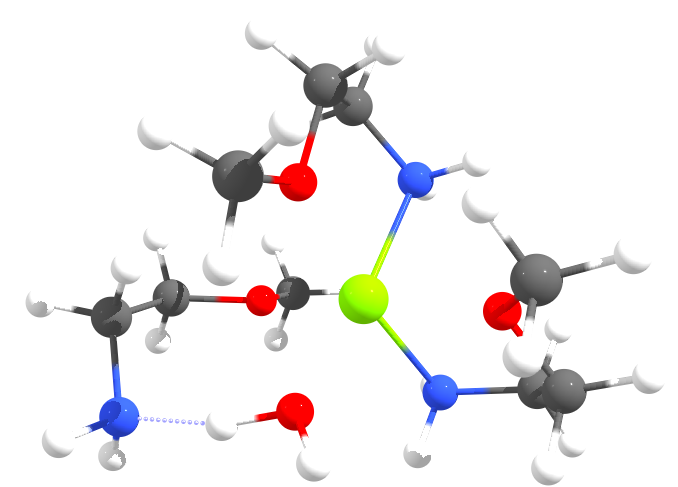


Method: (RI-)B3LYP(D3BJ)/def2-TZVPP

Symmetry: c1

Cartesian coordinates in Ångström:

C 0.5529437 2.0755926 -1.8593788

O 0.6132496 1.5370261 -0.5259871

H 1.5405990 2.4230015 -2.1630706

H -0.1479968 2.9097067 -1.9010879

H 0.2164261 1.2791196 -2.5140202

C 0.9529984 2.5357101 0.4562425

H 1.8189869 3.1046174 0.1157029

C 1.2636504 1.8329283 1.7578258

H 0.1096906 3.2248802 0.5621249

H 2.1926402 1.2722501 1.6652857

N 0.1801461 0.8638236 2.0620322

H 1.3950297 2.5750322 2.5462069

Mg -0.1687688 -0.2992205 0.2208120

H -0.6776359 1.3699539 2.2694603

H 0.4091404 0.3686025 2.9174469

H -3.1055906 1.0797056 -1.5369234

C -2.7742360 1.4558608 -0.5675714

H -3.6178353 1.9029281 -0.0428432

H -3.4449208 -1.1704002 -0.3611213

H -1.9984067 2.2005376 -0.7068254

H -1.3674857 -2.6645203 0.3621023

C -3.1817750 -0.6223358 0.5493081

O -2.2136396 0.3936345 0.2230022

N -1.2447062 -2.0059846 1.1279341

H -4.0860049 -0.1526526 0.9381896

C -2.5749211 -1.5329591 1.5909180

H -3.2611003 -2.3561459 1.7942459

H -0.8130635 -2.5466989 1.8697018

H -2.4297717 -0.9850806 2.5216069

H 2.4244381 0.0836251 -1.8635818

H 1.9917124 -1.6152629 -3.5665058

C 2.8252925 -0.9275426 -1.8147973

H 3.8281608 -0.8973859 -2.2480321

N 1.9272092 -1.8208823 -2.5740317

H 2.2103390 -2.7910609 -2.4708570

C 3.0027036 -1.3558347 -0.3784528

H 3.7639514 -0.7260280 0.0872316

O 1.7960346 -1.2715867 0.4225139

C 2.0596783 -1.8471880 1.7163073

H 2.8993446 -1.3392322 2.1931433

H 3.3540299 -2.3905841 -0.3357812

H 2.2892723 -2.9082259 1.6166578

H 1.1735691 -1.7271559 2.3296545

O -0.3560438 -1.1054391 -1.6177587

H 0.4975050 -1.4947216 -2.0798205

H -1.0935791 -1.1372871 -2.2304889

SCF energy GEOOPT = -1025.210958360 H

ZPE = 1089. kJ/mol

FREEH energy = 1153.06 kJ/mol

FREEH entropy = 0.68536 kJ/mol/K

$vibrational spectrum

# mode symmetry wave number IR intensity selection rules

# cm**(-1) km/mol IR RAMAN

1 -0.00 0.00000 - -

2 0.00 0.00000 - -

3 0.00 0.00000 - -

4 0.00 0.00000 - -

5 0.00 0.00000 - -

6 0.00 0.00000 - -

7 a 37.70 0.29923 YES YES

8 a 46.07 0.15887 YES YES

9 a 55.91 0.49779 YES YES

10 a 59.54 0.26991 YES YES

11 a 71.09 0.03647 YES YES

12 a 86.66 0.80591 YES YES

13 a 100.21 0.41067 YES YES

14 a 103.16 0.90129 YES YES

15 a 125.57 2.71347 YES YES

16 a 138.59 2.97737 YES YES

17 a 147.71 5.22862 YES YES

18 a 153.94 2.99880 YES YES

19 a 158.45 3.32753 YES YES

20 a 161.43 11.40789 YES YES

21 a 169.22 1.05894 YES YES

22 a 182.08 3.10499 YES YES

23 a 193.70 1.58194 YES YES

24 a 212.60 3.63627 YES YES

25 a 220.18 5.30254 YES YES

26 a 240.10 1.47883 YES YES

27 a 249.40 11.22661 YES YES

28 a 266.48 19.66964 YES YES

29 a 279.60 35.50903 YES YES

30 a 282.76 55.34629 YES YES

31 a 293.07 1.85452 YES YES

32 a 301.25 6.58044 YES YES

33 a 309.98 23.06678 YES YES

34 a 313.66 5.15257 YES YES

35 a 343.53 44.84980 YES YES

36 a 352.94 31.40089 YES YES

37 a 373.32 21.31303 YES YES

38 a 400.62 18.35360 YES YES

39 a 413.89 32.24915 YES YES

40 a 418.27 11.04132 YES YES

41 a 472.85 8.94630 YES YES

42 a 501.71 26.88927 YES YES

43 a 513.68 40.77510 YES YES

44 a 529.18 3.20695 YES YES

45 a 581.64 11.79470 YES YES

46 a 614.16 58.57234 YES YES

47 a 774.18 99.62242 YES YES

48 a 821.50 3.70412 YES YES

49 a 835.23 11.12694 YES YES

50 a 836.93 8.68750 YES YES

51 a 895.20 18.94886 YES YES

52 a 902.03 51.25385 YES YES

53 a 903.77 15.55936 YES YES

54 a 981.63 107.99099 YES YES

55 a 987.43 142.59208 YES YES

56 a 992.86 21.13621 YES YES

57 a 999.01 7.53256 YES YES

58 a 1020.73 14.53235 YES YES

59 a 1022.63 22.15458 YES YES

60 a 1038.86 78.99935 YES YES

61 a 1048.87 72.82808 YES YES

62 a 1053.53 140.68094 YES YES

63 a 1098.34 99.44721 YES YES

64 a 1100.45 127.89966 YES YES

65 a 1111.89 10.89859 YES YES

66 a 1115.40 66.56944 YES YES

67 a 1126.58 53.69687 YES YES

68 a 1127.72 73.64226 YES YES

69 a 1169.32 32.77533 YES YES

70 a 1174.82 1.86488 YES YES

71 a 1177.93 1.87343 YES YES

72 a 1184.45 22.70336 YES YES

73 a 1216.52 12.01129 YES YES

74 a 1219.26 9.82747 YES YES

75 a 1220.57 4.55141 YES YES

76 a 1221.81 1.91224 YES YES

77 a 1224.31 1.45496 YES YES

78 a 1235.13 17.03282 YES YES

79 a 1311.92 10.63659 YES YES

80 a 1313.77 9.36599 YES YES

81 a 1326.74 11.55804 YES YES

82 a 1332.41 17.07052 YES YES

83 a 1335.49 8.09685 YES YES

84 a 1339.05 4.23935 YES YES

85 a 1412.85 5.47074 YES YES

86 a 1414.63 6.84047 YES YES

87 a 1423.64 12.33621 YES YES

88 a 1446.68 1.75987 YES YES

89 a 1448.64 1.48761 YES YES

90 a 1453.59 6.45707 YES YES

91 a 1482.61 1.47714 YES YES

92 a 1485.54 0.99905 YES YES

93 a 1488.10 16.08901 YES YES

94 a 1489.13 1.35309 YES YES

95 a 1499.81 4.06275 YES YES

96 a 1501.47 11.47250 YES YES

97 a 1504.52 1.67648 YES YES

98 a 1505.28 15.49822 YES YES

99 a 1507.81 15.01491 YES YES

100 a 1509.34 10.74178 YES YES

101 a 1509.99 4.55230 YES YES

102 a 1511.32 8.07211 YES YES

103 a 1517.80 16.15561 YES YES

104 a 1522.63 11.20620 YES YES

105 a 1525.34 15.92347 YES YES

106 a 1655.05 69.07222 YES YES

107 a 1658.84 34.34831 YES YES

108 a 1660.23 29.38308 YES YES

109 a 1706.96 131.51965 YES YES

110 a 2301.75 2341.49009 YES YES

111 a 3018.86 15.97646 YES YES

112 a 3024.28 15.12655 YES YES

113 a 3025.50 11.17974 YES YES

114 a 3039.18 7.86325 YES YES

115 a 3039.32 20.45362 YES YES

116 a 3041.90 18.06391 YES YES

117 a 3045.00 5.27373 YES YES

118 a 3058.79 6.35077 YES YES

119 a 3062.57 7.04093 YES YES

120 a 3071.05 10.42990 YES YES

121 a 3087.63 9.63596 YES YES

122 a 3087.80 5.99846 YES YES

123 a 3105.03 8.56728 YES YES

124 a 3105.50 11.63176 YES YES

125 a 3108.38 16.26286 YES YES

126 a 3110.58 5.34637 YES YES

127 a 3111.51 19.00512 YES YES

128 a 3111.95 1.02953 YES YES

129 a 3164.26 22.48763 YES YES

130 a 3169.43 5.03864 YES YES

131 a 3171.73 9.96728 YES YES

132 a 3469.95 16.02293 YES YES

133 a 3470.34 8.72541 YES YES

134 a 3478.05 20.43854 YES YES

135 a 3537.99 13.03150 YES YES

136 a 3539.36 23.12959 YES YES

137 a 3541.91 25.38249 YES YES

138 a 3877.94 88.36973 YES YES

$end

Total COSMO energy + OC correction = -1025.4046146171 H

**[Mg(MOEA)_3_(OH)]^+^**


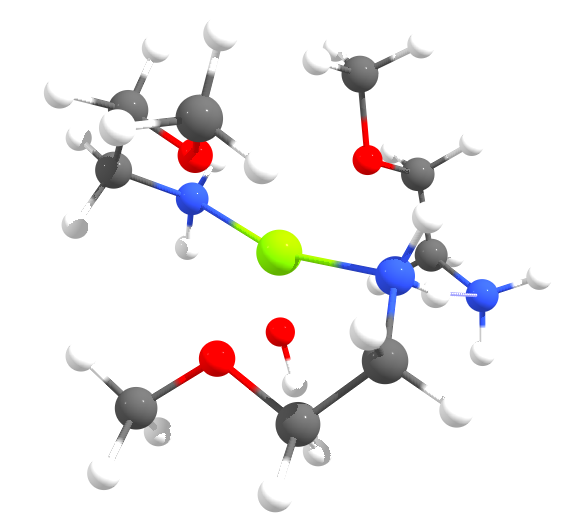


Method: (RI-)B3LYP(D3BJ)/def2-TZVPP

Symmetry: c1

Cartesian coordinates in Ångström:

C 1.1134893 1.8738041 -2.0728910

O 0.7261215 1.7143181 -0.7093605

H 2.1313356 2.2611988 -2.1349420

H 0.4323908 2.5528437 -2.5912019

H 1.0778399 0.8928707 -2.5344170

C 0.7614341 2.9300828 0.0513308

H 1.6178108 3.5319086 -0.2579215

C 0.8737876 2.5506215 1.5169270

H -0.1501672 3.5034465 -0.1472813

H 1.8680814 2.1484110 1.7081515

N -0.1019865 1.4945003 1.8024059

H 0.7559549 3.4417325 2.1377783

Mg 0.0098513 -0.1211174 0.2908657

H -1.0228430 1.8711686 1.9897148

H 0.1539171 0.9013639 2.5866545

H -3.2926968 1.1891375 -1.5550748

C -2.6021816 1.4214394 -0.7410981

H -3.0963093 2.1044757 -0.0443773

H -4.0467665 -0.6198746 -0.3652313

H -1.7141780 1.8965561 -1.1469042

H -3.2519478 -3.0873381 -0.5964466

C -3.3194608 -0.4995052 0.4452399

O -2.1915041 0.2292377 -0.0788376

N -2.4654084 -2.7700687 -0.0400408

H -3.7872499 0.1018230 1.2306006

C -2.9102282 -1.8393385 1.0105425

H -3.7676429 -2.2186504 1.5777277

H -2.0694279 -3.5973979 0.3903181

H -2.0760306 -1.6950938 1.6984977

H 1.4044542 -1.4561435 -2.7559679

H -0.7361663 -0.9205131 -2.2781591

C 0.9677712 -2.0255181 -1.9355016

H 0.7741986 -3.0350538 -2.3046280

N -0.2625931 -1.3429271 -1.4891393

H -0.9498673 -1.9972121 -1.0835124

C 1.9667901 -2.0880160 -0.7978941

H 2.9274892 -2.4686640 -1.1532672

O 2.1248194 -0.7580155 -0.3004158

C 3.1232022 -0.6599045 0.7154459

H 4.0759122 -1.0451056 0.3477173

H 1.6093803 -2.7341058 0.0098834

H 2.8107138 -1.2026936 1.6086267

H 3.2350975 0.3956589 0.9513687

O 0.0667121 -1.2381286 1.8072344

H 0.3188505 -2.1199436 2.0658001

SCF energy GEOOPT = -1024.888400285 H

ZPE = 1051. kJ/mol

FREEH energy = 1116.84 kJ/mol

FREEH entropy = 0.70067 kJ/mol/K

$vibrational spectrum

# mode symmetry wave number IR intensity selection rules

# cm**(-1) km/mol IR RAMAN

1 -0.00 0.00000 - -

2 0.00 0.00000 - -

3 0.00 0.00000 - -

4 0.00 0.00000 - -

5 0.00 0.00000 - -

6 0.00 0.00000 - -

7 a 32.11 0.05208 YES YES

8 a 45.20 0.17291 YES YES

9 a 50.20 0.86239 YES YES

10 a 65.36 0.28707 YES YES

11 a 78.62 1.03427 YES YES

12 a 79.14 0.29664 YES YES

13 a 83.09 0.19590 YES YES

14 a 103.59 0.92832 YES YES

15 a 112.83 2.56773 YES YES

16 a 124.40 2.12461 YES YES

17 a 128.47 0.88951 YES YES

18 a 142.09 12.74094 YES YES

19 a 144.69 12.84722 YES YES

20 a 157.84 4.43282 YES YES

21 a 164.38 9.93182 YES YES

22 a 169.58 16.27173 YES YES

23 a 184.68 13.13747 YES YES

24 a 196.06 4.77793 YES YES

25 a 200.88 4.49001 YES YES

26 a 220.60 19.95186 YES YES

27 a 229.07 56.12917 YES YES

28 a 241.99 17.85064 YES YES

29 a 252.17 19.49725 YES YES

30 a 258.31 9.09098 YES YES

31 a 287.17 33.80548 YES YES

32 a 299.38 42.37083 YES YES

33 a 306.68 42.05664 YES YES

34 a 311.50 2.31411 YES YES

35 a 328.21 23.21514 YES YES

36 a 344.02 0.39215 YES YES

37 a 362.28 17.95186 YES YES

38 a 389.10 13.13278 YES YES

39 a 400.51 22.59411 YES YES

40 a 410.63 61.15434 YES YES

41 a 439.47 85.05021 YES YES

42 a 497.32 12.83752 YES YES

43 a 535.78 3.36793 YES YES

44 a 557.58 11.79355 YES YES

45 a 578.95 12.50399 YES YES

46 a 590.34 6.73841 YES YES

47 a 631.73 114.22968 YES YES

48 a 824.97 9.27949 YES YES

49 a 830.41 5.59050 YES YES

50 a 841.65 7.66251 YES YES

51 a 883.41 59.78369 YES YES

52 a 911.63 16.15197 YES YES

53 a 919.91 34.17577 YES YES

54 a 943.11 205.59458 YES YES

55 a 951.58 121.25837 YES YES

56 a 1003.82 5.51212 YES YES

57 a 1008.81 54.76621 YES YES

58 a 1018.36 0.82662 YES YES

59 a 1041.50 29.74239 YES YES

60 a 1044.31 8.10653 YES YES

61 a 1063.27 4.55041 YES YES

62 a 1074.92 43.97237 YES YES

63 a 1076.91 68.33763 YES YES

64 a 1080.59 103.42030 YES YES

65 a 1114.38 43.83314 YES YES

66 a 1116.14 78.92538 YES YES

67 a 1120.61 171.83940 YES YES

68 a 1153.34 71.30764 YES YES

69 a 1175.78 1.40327 YES YES

70 a 1177.94 0.59541 YES YES

71 a 1178.59 1.44100 YES YES

72 a 1212.36 4.82304 YES YES

73 a 1215.32 3.87596 YES YES

74 a 1217.30 7.31102 YES YES

75 a 1221.37 9.04964 YES YES

76 a 1237.07 17.47265 YES YES

77 a 1245.71 9.16899 YES YES

78 a 1301.61 5.61807 YES YES

79 a 1305.07 6.76330 YES YES

80 a 1326.26 2.75777 YES YES

81 a 1337.56 4.29231 YES YES

82 a 1344.42 5.47762 YES YES

83 a 1362.78 10.21451 YES YES

84 a 1405.58 11.32362 YES YES

85 a 1406.20 7.45431 YES YES

86 a 1421.10 10.94529 YES YES

87 a 1426.91 1.08755 YES YES

88 a 1432.34 7.62848 YES YES

89 a 1469.13 5.50997 YES YES

90 a 1477.30 0.44284 YES YES

91 a 1480.89 0.89915 YES YES

92 a 1485.00 1.40791 YES YES

93 a 1487.78 3.02443 YES YES

94 a 1490.71 6.46349 YES YES

95 a 1496.03 4.57346 YES YES

96 a 1498.21 10.08121 YES YES

97 a 1501.17 6.03560 YES YES

98 a 1503.42 4.59202 YES YES

99 a 1503.61 9.37920 YES YES

100 a 1505.82 16.59235 YES YES

101 a 1508.28 3.52947 YES YES

102 a 1513.42 12.04416 YES YES

103 a 1518.89 7.59401 YES YES

104 a 1523.27 8.12658 YES YES

105 a 1654.50 36.13147 YES YES

106 a 1668.92 25.01843 YES YES

107 a 1689.15 49.95711 YES YES

108 a 2994.41 15.33904 YES YES

109 a 3001.38 78.77984 YES YES

110 a 3009.81 14.05657 YES YES

111 a 3013.08 60.13758 YES YES

112 a 3015.50 18.68290 YES YES

113 a 3025.01 38.08790 YES YES

114 a 3027.89 33.05968 YES YES

115 a 3040.56 36.59311 YES YES

116 a 3041.22 25.69697 YES YES

117 a 3041.84 25.19295 YES YES

118 a 3060.81 28.60502 YES YES

119 a 3068.08 18.75090 YES YES

120 a 3071.33 18.64610 YES YES

121 a 3072.61 13.39571 YES YES

122 a 3088.53 17.45548 YES YES

123 a 3089.34 37.95716 YES YES

124 a 3096.98 17.11178 YES YES

125 a 3098.15 13.40539 YES YES

126 a 3130.70 20.09285 YES YES

127 a 3150.62 9.22511 YES YES

128 a 3163.06 11.84524 YES YES

129 a 3207.63 507.21703 YES YES

130 a 3478.66 41.47969 YES YES

131 a 3488.08 1.75486 YES YES

132 a 3542.06 10.83337 YES YES

133 a 3561.69 6.79521 YES YES

134 a 3565.16 23.60453 YES YES

135 a 3954.67 14.50001 YES YES

$end

Total COSMO energy + OC correction = -1024.9451574422 H

**[Mg(MOEA)_2_(OH)]^+^**


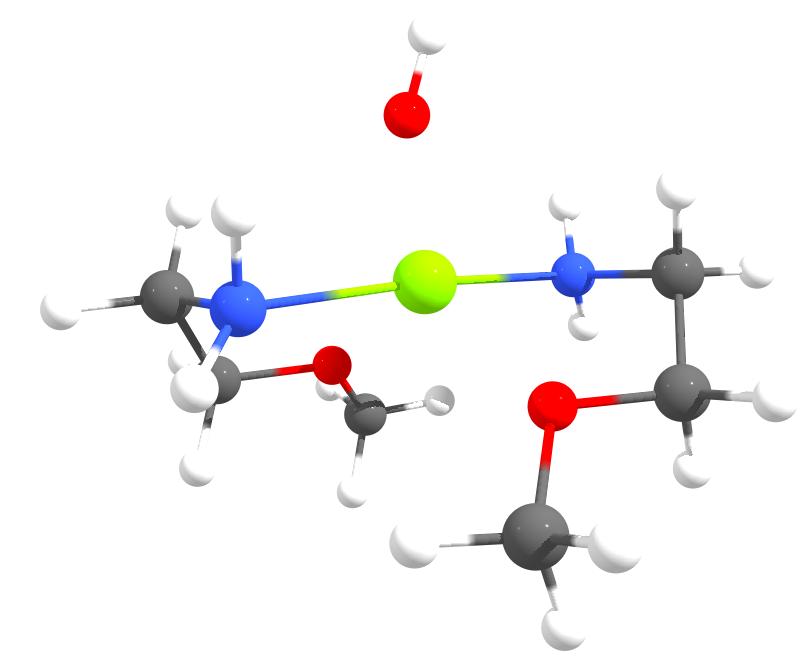


Method: (RI-)B3LYP(D3BJ)/def2-TZVPP

Symmetry: c1

Cartesian coordinates in Ångström:

Mg -0.3022895 0.8061409 0.1263875

O -1.1263738 2.4320004 0.2917767

H -2.3619643 -2.2021271 1.5948445

H -1.0577402 -1.1396927 3.3315104

H 0.7246177 0.5493926 3.8924407

H 1.2743527 1.9824130 1.8878616

C -0.2753650 -0.8577434 2.6248537

C 0.2269489 0.5398057 2.9213565

C -1.4939753 -2.0677073 0.9488677

N 1.1063616 0.9844112 1.8143436

O -0.8106466 -0.8570053 1.2900467

H -1.8173457 -1.9778808 -0.0830075

H 2.0097427 0.5276034 1.8793614

H -0.6147040 1.2308521 2.9606915

H 0.5279122 -1.5994483 2.6785650

H -0.8214070 -2.9210886 1.0492541

H 0.3846860 -0.7732479 -2.9900209

H -1.3263548 0.9067236 -3.6493953

H -2.1594177 -0.3702899 -1.8521865

H 2.4516026 -1.1816423 -1.6523266

C -0.8400091 0.9965765 -2.6765787

C 0.4935708 0.2798051 -2.7128223

N -1.6579444 0.4616545 -1.5666972

O 1.0662430 0.3460687 -1.3907299

C 2.4108165 -0.1385212 -1.3339299

H 1.1698765 0.7605687 -3.4216393

H -0.6805736 2.0549391 -2.4734989

H -2.3343078 1.1621291 -1.2731300

H 2.7453211 -0.0585123 -0.3043462

H 3.0547308 0.4671733 -1.9721450

H -1.1566745 3.3126792 0.6488526

SCF energy GEOOPT = -775.1911908956 H

ZPE = 714.2 kJ/mol

FREEH energy = 760.23 kJ/mol

FREEH entropy = 0.56396 kJ/mol/K

$vibrational spectrum

# mode symmetry wave number IR intensity selection rules

# cm**(-1) km/mol IR RAMAN

1 -0.00 0.00000 - -

2 -0.00 0.00000 - -

3 0.00 0.00000 - -

4 0.00 0.00000 - -

5 0.00 0.00000 - -

6 0.00 0.00000 - -

7 a 24.74 0.15607 YES YES

8 a 47.98 0.17496 YES YES

9 a 57.48 0.61384 YES YES

10 a 72.54 5.55774 YES YES

11 a 97.07 5.44099 YES YES

12 a 108.64 4.99512 YES YES

13 a 130.62 4.72356 YES YES

14 a 131.85 9.23762 YES YES

15 a 139.76 42.89597 YES YES

16 a 171.43 2.45531 YES YES

17 a 189.10 7.42029 YES YES

18 a 192.11 2.14605 YES YES

19 a 210.51 0.47600 YES YES

20 a 244.09 23.31221 YES YES

21 a 268.16 42.36118 YES YES

22 a 275.80 53.08312 YES YES

23 a 284.87 15.27023 YES YES

24 a 311.22 4.32000 YES YES

25 a 325.95 33.73964 YES YES

26 a 345.17 52.32619 YES YES

27 a 376.20 68.03676 YES YES

28 a 401.70 7.67423 YES YES

29 a 424.90 69.79799 YES YES

30 a 492.75 8.74385 YES YES

31 a 503.03 12.32409 YES YES

32 a 591.81 3.92769 YES YES

33 a 601.22 6.16926 YES YES

34 a 687.91 128.85270 YES YES

35 a 829.73 3.93331 YES YES

36 a 838.89 4.20983 YES YES

37 a 901.82 61.06901 YES YES

38 a 905.39 18.08780 YES YES

39 a 978.02 262.99866 YES YES

40 a 987.48 20.56737 YES YES

41 a 1015.29 6.43811 YES YES

42 a 1025.78 5.65028 YES YES

43 a 1056.87 15.52241 YES YES

44 a 1059.22 57.28910 YES YES

45 a 1089.43 163.82765 YES YES

46 a 1094.59 96.58393 YES YES

47 a 1106.97 40.24949 YES YES

48 a 1109.66 105.83944 YES YES

49 a 1178.15 0.61469 YES YES

50 a 1178.43 0.87522 YES YES

51 a 1216.24 2.15915 YES YES

52 a 1217.85 7.43416 YES YES

53 a 1227.51 7.13224 YES YES

54 a 1230.86 8.77812 YES YES

55 a 1306.12 8.17507 YES YES

56 a 1307.43 4.20862 YES YES

57 a 1335.64 7.27286 YES YES

58 a 1344.91 3.56526 YES YES

59 a 1408.04 2.95262 YES YES

60 a 1411.94 10.35369 YES YES

61 a 1436.94 2.27079 YES YES

62 a 1442.82 1.59799 YES YES

63 a 1484.62 0.05737 YES YES

64 a 1485.31 0.25937 YES YES

65 a 1488.81 8.69060 YES YES

66 a 1491.22 8.77198 YES YES

67 a 1500.12 8.70282 YES YES

68 a 1503.60 17.82435 YES YES

69 a 1504.53 1.97992 YES YES

70 a 1505.56 4.26431 YES YES

71 a 1520.54 7.10596 YES YES

72 a 1521.70 7.67892 YES YES

73 a 1631.19 50.62024 YES YES

74 a 1645.32 32.44364 YES YES

75 a 3016.93 21.52309 YES YES

76 a 3019.00 21.01060 YES YES

77 a 3034.28 26.59044 YES YES

78 a 3035.77 28.96215 YES YES

79 a 3052.62 23.56156 YES YES

80 a 3052.73 4.13198 YES YES

81 a 3078.47 18.46175 YES YES

82 a 3079.03 16.05443 YES YES

83 a 3101.33 12.56766 YES YES

84 a 3103.05 12.81984 YES YES

85 a 3106.36 2.74085 YES YES

86 a 3109.11 2.92435 YES YES

87 a 3153.68 16.85402 YES YES

88 a 3159.77 13.61274 YES YES

89 a 3469.52 38.05156 YES YES

90 a 3485.55 12.28791 YES YES

91 a 3555.65 18.96339 YES YES

92 a 3561.60 21.77825 YES YES

93 a 3994.08 50.24634 YES YES

$end

Total COSMO energy + OC correction = -775.2571513894 H

**[Mg(MOEA)_3_(OH)(H_2_O)]^+^**


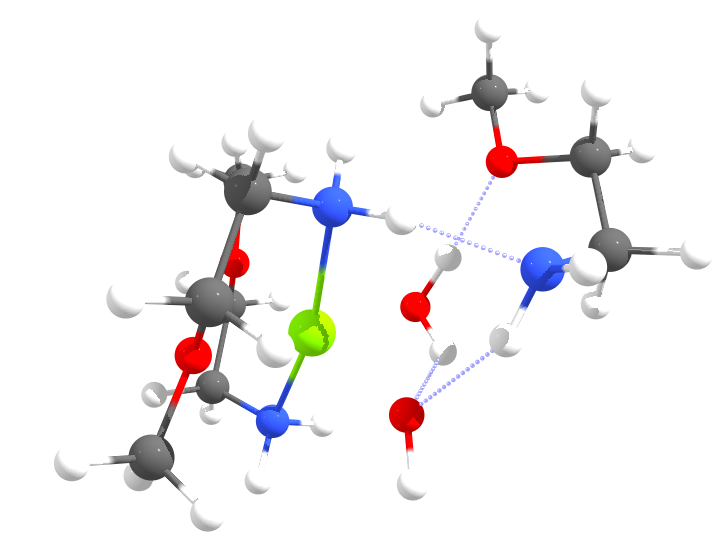


Method: (RI-)B3LYP(D3BJ)/def2-TZVPP

Symmetry: c1

Cartesian coordinates in Ångström:

C -0.0998145 1.9855569 -1.8994382

O 0.5846231 1.6944345 -0.6823709

H 0.3602867 2.8422849 -2.3937504

H -1.1540908 2.1992663 -1.7074336

H -0.0077396 1.1176273 -2.5440750

C 0.7066938 2.8457564 0.1624644

H 1.1424047 3.6709880 -0.4044808

C 1.6000905 2.4710088 1.3226169

H -0.2884831 3.1439755 0.5086650

H 2.5965079 2.2351295 0.9490600

N 1.0540458 1.2624535 1.9660618

H 1.6871059 3.3200333 2.0041905

Mg 0.2707757 -0.1776976 0.4799932

H 0.2144462 1.4896111 2.4914424

H 1.7069318 0.8764037 2.6372473

H -4.0391880 -0.4750994 -2.8404110

C -4.0442264 0.0542884 -1.8830251

H -5.0781403 0.2802485 -1.6083317

H -4.0067980 -2.5702289 -1.5550918

H -3.4921771 0.9870237 -1.9837829

H -1.9741090 -4.1496993 0.0926658

C -4.0520694 -1.9701283 -0.6396489

O -3.4034178 -0.7155842 -0.8757304

N -2.0182813 -3.1462596 0.2011171

H -5.1050227 -1.7873233 -0.3983812

C -3.3829309 -2.7023855 0.5080972

H -4.0311956 -3.5410769 0.7751546

H -1.3726033 -2.8603614 0.9411522

H -3.3647746 -2.0445550 1.3795859

H 1.2806140 -1.0617354 -2.6341540

H -0.9328920 -0.7992935 -1.9742994

C 0.8595105 -1.8174197 -1.9697519

H 0.6350288 -2.7017068 -2.5716557

N -0.3323189 -1.2634396 -1.3050872

H -0.9213403 -2.0184457 -0.9096965

C 1.8924077 -2.1855613 -0.9267785

H 2.8177847 -2.5206368 -1.3992419

O 2.1604335 -1.0114034 -0.1403371

C 3.1888799 -1.2260583 0.8266573

H 4.0941990 -1.5877039 0.3375443

H 1.5217163 -2.9740453 -0.2651254

H 2.8620766 -1.9455802 1.5801874

H 3.4008816 -0.2685916 1.2950343

O -0.2245905 -1.5274537 1.7881557

H 0.1518634 -1.8439886 2.6065340

O -1.7153101 0.4321365 0.9803032

H -2.4186085 0.1264278 0.3729775

H -1.6010653 -0.3003915 1.6289914

SCF energy GEOOPT = -1101.354844533 H

ZPE = 1122. kJ/mol

FREEH energy = 1191.55 kJ/mol

FREEH entropy = 0.73782 kJ/mol/K

$vibrational spectrum

# mode symmetry wave number IR intensity selection rules

# cm**(-1) km/mol IR RAMAN

1 -0.00 0.00000 - -

2 -0.00 0.00000 - -

3 -0.00 0.00000 - -

4 -0.00 0.00000 - -

5 0.00 0.00000 - -

6 0.00 0.00000 - -

7 a 28.35 0.24153 YES YES

8 a 40.49 0.07364 YES YES

9 a 48.98 0.36376 YES YES

10 a 56.02 0.78433 YES YES

11 a 58.86 1.45714 YES YES

12 a 68.46 0.38291 YES YES

13 a 76.46 1.46400 YES YES

14 a 92.75 0.82616 YES YES

15 a 105.82 8.33623 YES YES

16 a 110.45 1.00492 YES YES

17 a 116.11 1.09631 YES YES

18 a 122.01 1.94509 YES YES

19 a 127.04 5.37081 YES YES

20 a 153.27 0.96931 YES YES

21 a 175.01 7.07190 YES YES

22 a 179.88 10.80917 YES YES

23 a 182.38 11.83941 YES YES

24 a 184.93 3.96212 YES YES

25 a 199.87 3.49732 YES YES

26 a 210.47 3.12412 YES YES

27 a 218.48 5.71412 YES YES

28 a 225.89 8.63105 YES YES

29 a 236.23 11.29363 YES YES

30 a 250.20 45.12125 YES YES

31 a 266.48 6.69993 YES YES

32 a 269.28 2.57166 YES YES

33 a 277.53 1.85541 YES YES

34 a 298.93 12.32689 YES YES

35 a 308.78 2.43609 YES YES

36 a 322.52 13.79272 YES YES

37 a 337.51 24.84612 YES YES

38 a 342.64 12.46645 YES YES

39 a 359.90 32.64836 YES YES

40 a 378.79 21.16194 YES YES

41 a 405.24 4.08871 YES YES

42 a 425.73 61.58243 YES YES

43 a 444.42 5.07010 YES YES

44 a 494.36 46.83640 YES YES

45 a 516.31 93.31298 YES YES

46 a 536.16 18.09507 YES YES

47 a 541.15 103.96285 YES YES

48 a 556.81 9.68195 YES YES

49 a 591.34 42.90595 YES YES

50 a 651.47 54.12836 YES YES

51 a 666.15 25.55228 YES YES

52 a 793.83 81.17987 YES YES

53 a 807.47 13.07083 YES YES

54 a 839.65 77.51244 YES YES

55 a 841.94 19.89645 YES YES

56 a 853.02 140.66210 YES YES

57 a 901.08 51.88465 YES YES

58 a 914.97 12.00068 YES YES

59 a 918.37 27.05871 YES YES

60 a 968.42 213.98369 YES YES

61 a 987.64 126.99367 YES YES

62 a 1004.63 25.02235 YES YES

63 a 1018.87 17.50093 YES YES

64 a 1019.87 30.95868 YES YES

65 a 1056.02 0.57461 YES YES

66 a 1064.12 13.03537 YES YES

67 a 1071.90 19.32438 YES YES

68 a 1074.88 79.79143 YES YES

69 a 1089.64 68.54729 YES YES

70 a 1101.41 135.99566 YES YES

71 a 1111.22 89.48251 YES YES

72 a 1117.12 152.00505 YES YES

73 a 1132.27 34.01477 YES YES

74 a 1163.45 34.96845 YES YES

75 a 1177.22 0.81193 YES YES

76 a 1178.04 1.91707 YES YES

77 a 1179.20 1.82715 YES YES

78 a 1205.63 3.04790 YES YES

79 a 1216.64 4.08688 YES YES

80 a 1219.18 2.73829 YES YES

81 a 1224.15 5.09564 YES YES

82 a 1239.92 15.22156 YES YES

83 a 1250.45 12.99852 YES YES

84 a 1305.73 6.64985 YES YES

85 a 1309.45 5.82380 YES YES

86 a 1311.98 9.77295 YES YES

87 a 1334.43 7.77185 YES YES

88 a 1366.35 5.22021 YES YES

89 a 1389.57 9.12496 YES YES

90 a 1403.72 12.62971 YES YES

91 a 1410.49 3.63862 YES YES

92 a 1411.31 9.10317 YES YES

93 a 1423.73 3.88769 YES YES

94 a 1441.02 5.32408 YES YES

95 a 1441.91 4.72012 YES YES

96 a 1480.05 0.87368 YES YES

97 a 1481.15 0.53669 YES YES

98 a 1482.44 1.54378 YES YES

99 a 1485.17 7.07885 YES YES

100 a 1487.08 7.58196 YES YES

101 a 1494.18 9.42393 YES YES

102 a 1496.11 11.75764 YES YES

103 a 1500.49 3.63169 YES YES

104 a 1501.46 4.85861 YES YES

105 a 1503.17 8.16618 YES YES

106 a 1504.89 12.60707 YES YES

107 a 1507.57 3.89597 YES YES

108 a 1515.16 12.90690 YES YES

109 a 1517.20 3.77876 YES YES

110 a 1519.21 6.92689 YES YES

111 a 1656.09 37.53400 YES YES

112 a 1678.39 29.90194 YES YES

113 a 1691.00 27.27345 YES YES

114 a 1703.53 92.99480 YES YES

115 a 2993.18 26.97928 YES YES

116 a 3004.80 57.51587 YES YES

117 a 3013.19 15.17479 YES YES

118 a 3021.48 10.72302 YES YES

119 a 3023.17 17.98196 YES YES

120 a 3024.47 38.58787 YES YES

121 a 3028.48 29.22234 YES YES

122 a 3032.02 47.96561 YES YES

123 a 3037.06 64.18783 YES YES

124 a 3041.91 20.07349 YES YES

125 a 3060.81 24.17692 YES YES

126 a 3068.18 22.86551 YES YES

127 a 3069.15 24.29893 YES YES

128 a 3070.58 19.27452 YES YES

129 a 3084.19 66.19234 YES YES

130 a 3088.07 25.14059 YES YES

131 a 3094.85 10.23896 YES YES

132 a 3094.97 15.03478 YES YES

133 a 3114.18 12.17037 YES YES

134 a 3130.75 579.06649 YES YES

135 a 3137.25 18.03965 YES YES

136 a 3155.18 16.81270 YES YES

137 a 3340.03 294.95369 YES YES

138 a 3415.90 284.34161 YES YES

139 a 3484.43 10.43265 YES YES

140 a 3516.00 771.77516 YES YES

141 a 3553.00 7.92083 YES YES

142 a 3561.20 15.53807 YES YES

143 a 3571.87 28.34334 YES YES

144 a 3930.07 18.77744 YES YES

$end

Total COSMO energy + OC correction = -1101.4094995496 H

**[Mg(MOEA)_2_(OH)_2_]^+^**


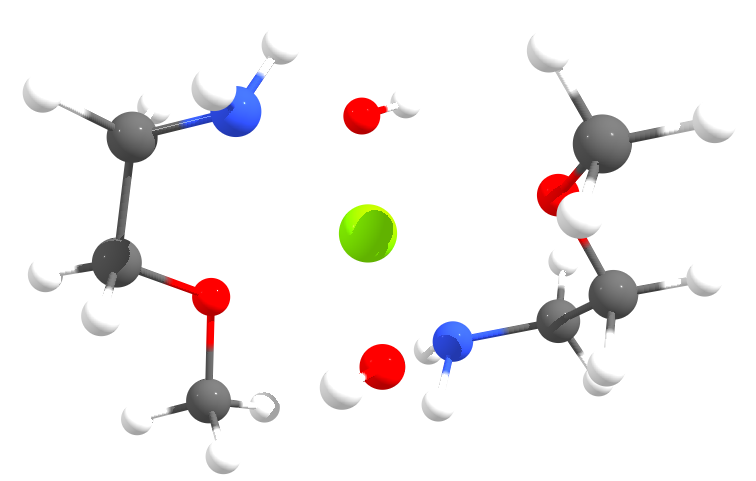


Method: (RI-)B3LYP(D3BJ)/def2-TZVPP

Symmetry: c1

Cartesian coordinates in Ångström:

O 0.6423734 -1.4717789 0.0200957

Mg -0.2337711 0.2727182 -0.0035371

O -1.3199080 1.8520578 -0.0454407

H -2.8225726 -1.9173442 2.4260574

H -0.9251749 -1.0883707 3.7907617

H 1.2255064 0.0538964 3.8015993

H 1.7922614 1.3722757 1.7759602

C -0.4327707 -0.8757698 2.8349397

C 0.5262704 0.2867539 2.9906750

C -2.2865556 -1.5713975 1.5364988

N 1.1995933 0.5560064 1.7125686

O -1.4044697 -0.5111321 1.8630637

H -3.0036569 -1.1758571 0.8198549

H 1.7480510 -0.2499910 1.4194570

H -0.0337167 1.1787805 3.2745084

H 0.1016860 -1.7663202 2.4907025

H -1.7318421 -2.4000904 1.0885193

H 0.9693528 -0.5777702 -2.9587704

H -1.0439119 0.6128740 -3.9670679

H -1.8090595 -0.8218281 -2.2992000

H 2.7187025 -0.4699248 -1.2365948

C -0.6920995 0.8060782 -2.9490450

C 0.7896435 0.5008026 -2.8726234

N -1.4318051 0.0411307 -1.9352680

O 1.2708625 0.9599361 -1.6132232

C 2.6287058 0.6076100 -1.3896580

H 1.3318014 1.0105956 -3.6772062

H -0.8388394 1.8626818 -2.7297866

H -2.1602377 0.6203756 -1.5269249

H 2.9538553 1.1292026 -0.4921110

H 3.2501199 0.9325834 -2.2293648

H -1.5263191 2.4254549 0.6891952

H 0.5479247 -2.1842391 -0.6086353

SCF energy GEOOPT = -851.2134264734 H

ZPE = 743.1 kJ/mol

FREEH energy = 794.43 kJ/mol

FREEH entropy = 0.59258 kJ/mol/K

$vibrational spectrum

# mode symmetry wave number IR intensity selection rules

# cm**(-1) km/mol IR RAMAN

1 -0.00 0.00000 - -

2 -0.00 0.00000 - -

3 0.00 0.00000 - -

4 0.00 0.00000 - -

5 0.00 0.00000 - -

6 0.00 0.00000 - -

7 a 34.79 3.79342 YES YES

8 a 43.66 0.95159 YES YES

9 a 51.90 0.85967 YES YES

10 a 94.97 0.88666 YES YES

11 a 103.86 0.56986 YES YES

12 a 114.96 1.80574 YES YES

13 a 127.15 15.46213 YES YES

14 a 138.26 8.24836 YES YES

15 a 150.75 14.64769 YES YES

16 a 168.94 6.34351 YES YES

17 a 187.53 17.61436 YES YES

18 a 194.45 2.38664 YES YES

19 a 194.97 1.69138 YES YES

20 a 218.65 45.26803 YES YES

21 a 226.83 31.16099 YES YES

22 a 233.79 1.52665 YES YES

23 a 256.93 115.06650 YES YES

24 a 268.99 9.06776 YES YES

25 a 283.43 26.49159 YES YES

26 a 296.96 24.53046 YES YES

27 a 297.91 11.97926 YES YES

28 a 316.18 94.65083 YES YES

29 a 320.15 53.20139 YES YES

30 a 370.05 7.08794 YES YES

31 a 384.99 1.86423 YES YES

32 a 429.94 5.55907 YES YES

33 a 475.63 77.90950 YES YES

34 a 484.00 34.91473 YES YES

35 a 521.13 14.56442 YES YES

36 a 534.30 228.74154 YES YES

37 a 573.52 6.72655 YES YES

38 a 580.46 16.46864 YES YES

39 a 643.70 104.89060 YES YES

40 a 815.15 14.84428 YES YES

41 a 845.31 12.47432 YES YES

42 a 896.19 181.48417 YES YES

43 a 899.92 5.08445 YES YES

44 a 930.20 129.85762 YES YES

45 a 953.25 110.38511 YES YES

46 a 1026.36 12.14106 YES YES

47 a 1031.23 20.14212 YES YES

48 a 1053.02 29.11194 YES YES

49 a 1073.27 19.46659 YES YES

50 a 1085.61 29.09707 YES YES

51 a 1091.64 42.57961 YES YES

52 a 1125.16 77.70062 YES YES

53 a 1136.62 136.20448 YES YES

54 a 1175.66 0.74613 YES YES

55 a 1177.81 2.30549 YES YES

56 a 1210.68 11.58895 YES YES

57 a 1216.26 4.68786 YES YES

58 a 1234.40 3.95061 YES YES

59 a 1243.36 8.13406 YES YES

60 a 1301.08 4.61221 YES YES

61 a 1307.33 6.34740 YES YES

62 a 1332.86 7.72277 YES YES

63 a 1357.32 2.43343 YES YES

64 a 1403.58 6.99358 YES YES

65 a 1411.97 8.71874 YES YES

66 a 1428.20 3.73875 YES YES

67 a 1440.98 4.31523 YES YES

68 a 1470.80 2.11243 YES YES

69 a 1472.33 1.14084 YES YES

70 a 1477.19 4.93565 YES YES

71 a 1489.74 8.05211 YES YES

72 a 1490.87 7.36847 YES YES

73 a 1495.90 3.93797 YES YES

74 a 1502.12 3.58405 YES YES

75 a 1505.32 6.94642 YES YES

76 a 1506.89 18.30109 YES YES

77 a 1508.57 3.90368 YES YES

78 a 1620.98 31.35921 YES YES

79 a 1645.45 24.92993 YES YES

80 a 2982.25 27.53606 YES YES

81 a 2988.38 2.13057 YES YES

82 a 2997.34 69.23854 YES YES

83 a 3004.15 148.24966 YES YES

84 a 3005.60 62.47124 YES YES

85 a 3013.24 37.98230 YES YES

86 a 3022.72 87.04674 YES YES

87 a 3041.94 11.27220 YES YES

88 a 3058.13 27.80348 YES YES

89 a 3072.32 19.92812 YES YES

90 a 3076.25 25.97537 YES YES

91 a 3103.56 4.61391 YES YES

92 a 3116.68 18.69498 YES YES

93 a 3119.66 28.47364 YES YES

94 a 3444.88 26.43203 YES YES

95 a 3457.15 61.98686 YES YES

96 a 3579.12 13.71368 YES YES

97 a 3591.30 17.96046 YES YES

98 a 3918.74 2.31665 YES YES

99 a 3926.92 2.81279 YES YES

$end

Total COSMO energy + OC correction = -851.2368824988 H

**[Mg(MOEA)_2_(OH)_2_(MOEA)]^+^**


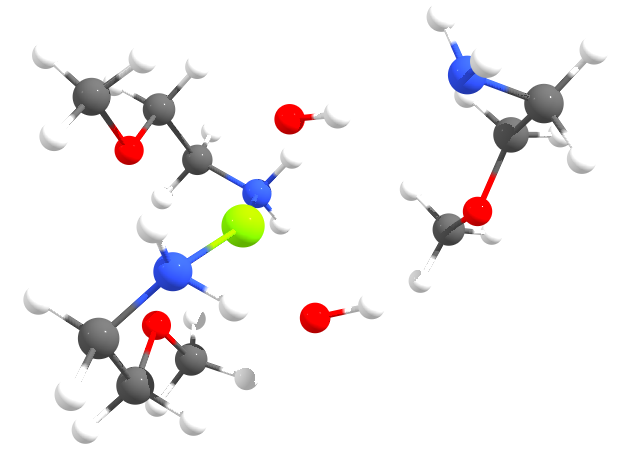


Method: (RI-)B3LYP(D3BJ)/def2-TZVPP

Symmetry: c1

Cartesian coordinates in Ångström:

C 0.4537088 2.6557430 -1.6874313

O 1.0528413 1.9918003 -0.5794135

H 1.0170833 3.5600421 -1.9371316

H -0.5845185 2.8979452 -1.4516393

H 0.4942214 1.9713172 -2.5310283

C 1.2028647 2.8665795 0.5364133

H 1.9372155 3.6435825 0.2969418

C 1.6699094 2.0533957 1.7253830

H 0.2358417 3.3293617 0.7531837

H 2.6228914 1.5765491 1.4877918

N 0.6826891 1.0094054 2.0119686

H 1.8377632 2.7368167 2.5655956

Mg -0.2725941 0.1956941 0.1622328

H -0.2319299 1.4225447 2.1956679

H 0.9504650 0.4187002 2.7867524

H -3.4139630 -0.8342647 -1.5635780

C -4.0738306 -0.1012577 -1.0837705

H -5.0469013 -0.1265912 -1.5926229

H -3.9562438 -2.4033408 0.0493243

H -3.6284679 0.8865090 -1.1779349

H -2.9118587 -3.0459916 2.2902338

C -4.6306343 -1.6932100 0.5486080

O -4.2081967 -0.3718876 0.2926121

N -3.2871982 -2.1469875 2.5668985

H -5.6415429 -1.8488308 0.1433821

C -4.6392216 -1.9561601 2.0430572

H -5.3049436 -2.8105439 2.2316322

H -3.2744088 -2.1100453 3.5771410

H -5.0803893 -1.0887628 2.5368790

H 1.3614210 -0.3461868 -2.8767212

H -1.0323773 -0.0274326 -2.6315263

C 0.7174266 -1.1373687 -2.4871435

H 0.5208272 -1.8414229 -3.3040081

N -0.4966184 -0.5302655 -1.9384446

H -1.0840104 -1.2380425 -1.4911948

C 1.4406567 -1.8807223 -1.3843471

H 2.3554813 -2.3414652 -1.7737592

O 1.7732195 -0.9540981 -0.3542322

C 2.2863343 -1.6169873 0.7948251

H 3.1226715 -2.2666334 0.5180745

H 0.7905596 -2.6547318 -0.9657138

H 1.4898744 -2.1939315 1.2702789

H 2.6492052 -0.8499377 1.4745464

O -0.8780457 -1.5951980 0.5816918

H -1.5942428 -1.6330841 1.2212835

O -1.5696906 1.6105832 0.4265375

H -2.4442739 1.3322129 0.7008341

SCF energy GEOOPT = -1100.885168008 H

ZPE = 1081. kJ/mol

FREEH energy = 1152.47 kJ/mol

FREEH entropy = 0.77270 kJ/mol/K

$vibrational spectrum

# mode symmetry wave number IR intensity selection rules

# cm**(-1) km/mol IR RAMAN

1 -0.00 0.00000 - -

2 0.00 0.00000 - -

3 0.00 0.00000 - -

4 0.00 0.00000 - -

5 0.00 0.00000 - -

6 0.00 0.00000 - -

7 a 16.58 2.33736 YES YES

8 a 22.91 3.01104 YES YES

9 a 32.17 0.32921 YES YES

10 a 38.65 2.37857 YES YES

11 a 45.27 0.22182 YES YES

12 a 59.36 2.13471 YES YES

13 a 78.54 3.83289 YES YES

14 a 81.20 3.54443 YES YES

15 a 85.53 0.67006 YES YES

16 a 90.52 1.50686 YES YES

17 a 109.83 1.77634 YES YES

18 a 121.91 1.02038 YES YES

19 a 129.58 3.32984 YES YES

20 a 134.21 5.38513 YES YES

21 a 139.13 4.65768 YES YES

22 a 147.90 2.08857 YES YES

23 a 155.72 11.01912 YES YES

24 a 182.06 85.69236 YES YES

25 a 187.60 57.81293 YES YES

26 a 198.97 0.53364 YES YES

27 a 200.86 8.96613 YES YES

28 a 215.13 4.16662 YES YES

29 a 225.45 10.89272 YES YES

30 a 230.17 6.73942 YES YES

31 a 238.33 4.54189 YES YES

32 a 261.17 9.77759 YES YES

33 a 274.46 13.68279 YES YES

34 a 288.51 8.42245 YES YES

35 a 292.50 3.49949 YES YES

36 a 301.42 2.41924 YES YES

37 a 306.57 10.13214 YES YES

38 a 343.39 36.03449 YES YES

39 a 366.30 0.22635 YES YES

40 a 391.85 5.26244 YES YES

41 a 396.79 9.31859 YES YES

42 a 483.44 6.31339 YES YES

43 a 492.36 22.74851 YES YES

44 a 496.93 116.40608 YES YES

45 a 516.69 40.76245 YES YES

46 a 522.32 11.21252 YES YES

47 a 555.40 60.17476 YES YES

48 a 572.96 19.13157 YES YES

49 a 583.21 3.36597 YES YES

50 a 617.32 172.47397 YES YES

51 a 620.54 183.39815 YES YES

52 a 756.83 119.32264 YES YES

53 a 819.09 51.67196 YES YES

54 a 843.25 12.23847 YES YES

55 a 849.95 8.11034 YES YES

56 a 867.06 78.07996 YES YES

57 a 906.80 91.70727 YES YES

58 a 910.30 28.13141 YES YES

59 a 929.08 23.08791 YES YES

60 a 951.98 221.66209 YES YES

61 a 958.51 26.94889 YES YES

62 a 1010.28 0.04259 YES YES

63 a 1029.60 52.02761 YES YES

64 a 1034.30 7.78933 YES YES

65 a 1055.76 20.96749 YES YES

66 a 1072.93 28.54262 YES YES

67 a 1077.47 14.78475 YES YES

68 a 1083.59 17.31258 YES YES

69 a 1087.24 29.17019 YES YES

70 a 1099.58 26.52490 YES YES

71 a 1122.71 43.11946 YES YES

72 a 1125.45 160.13822 YES YES

73 a 1153.97 120.92596 YES YES

74 a 1175.09 2.51999 YES YES

75 a 1180.16 4.60187 YES YES

76 a 1181.45 2.37034 YES YES

77 a 1202.97 6.23067 YES YES

78 a 1216.26 3.25663 YES YES

79 a 1216.98 9.54860 YES YES

80 a 1234.94 0.99826 YES YES

81 a 1235.15 3.85430 YES YES

82 a 1243.09 14.17194 YES YES

83 a 1302.67 6.31000 YES YES

84 a 1304.28 6.35125 YES YES

85 a 1306.94 3.29932 YES YES

86 a 1329.68 5.15477 YES YES

87 a 1331.48 5.28418 YES YES

88 a 1335.64 6.94175 YES YES

89 a 1409.40 11.57247 YES YES

90 a 1411.36 3.86709 YES YES

91 a 1412.39 7.94711 YES YES

92 a 1434.80 14.60120 YES YES

93 a 1438.62 1.39079 YES YES

94 a 1440.04 6.62517 YES YES

95 a 1470.59 4.65793 YES YES

96 a 1473.39 0.41757 YES YES

97 a 1478.22 5.00028 YES YES

98 a 1485.62 9.21204 YES YES

99 a 1487.12 5.46073 YES YES

100 a 1488.22 4.80527 YES YES

101 a 1494.11 3.41482 YES YES

102 a 1499.45 8.77645 YES YES

103 a 1501.33 4.86434 YES YES

104 a 1502.59 5.32374 YES YES

105 a 1505.61 3.21584 YES YES

106 a 1506.94 8.51357 YES YES

107 a 1511.52 7.14843 YES YES

108 a 1515.38 5.48586 YES YES

109 a 1517.40 3.32481 YES YES

110 a 1642.66 31.25848 YES YES

111 a 1650.93 41.14135 YES YES

112 a 1657.72 23.97188 YES YES

113 a 2938.14 4.19108 YES YES

114 a 2955.45 103.56803 YES YES

115 a 2965.16 156.73161 YES YES

116 a 2977.54 63.14621 YES YES

117 a 2986.91 3.88376 YES YES

118 a 2989.66 4.27904 YES YES

119 a 2996.82 68.31343 YES YES

120 a 2999.83 75.17160 YES YES

121 a 3003.11 187.37031 YES YES

122 a 3005.37 75.83050 YES YES

123 a 3006.54 96.43385 YES YES

124 a 3040.47 5.20924 YES YES

125 a 3044.04 5.29972 YES YES

126 a 3062.65 38.03136 YES YES

127 a 3066.11 36.41630 YES YES

128 a 3067.90 23.85749 YES YES

129 a 3068.38 13.89693 YES YES

130 a 3074.56 12.84860 YES YES

131 a 3120.78 7.82613 YES YES

132 a 3125.65 24.88057 YES YES

133 a 3128.58 29.20949 YES YES

134 a 3370.84 91.83445 YES YES

135 a 3412.92 56.24193 YES YES

136 a 3503.21 0.14121 YES YES

137 a 3578.81 9.30689 YES YES

138 a 3580.30 9.26261 YES YES

139 a 3585.70 2.02881 YES YES

140 a 3819.70 181.50988 YES YES

141 a 3883.58 24.54202 YES YES

$end

Total COSMO energy + OC correction = -1100.9109631997 H

**[Mg(MOEA)_2_(H_2_O)_2_(TfO)_2_]**


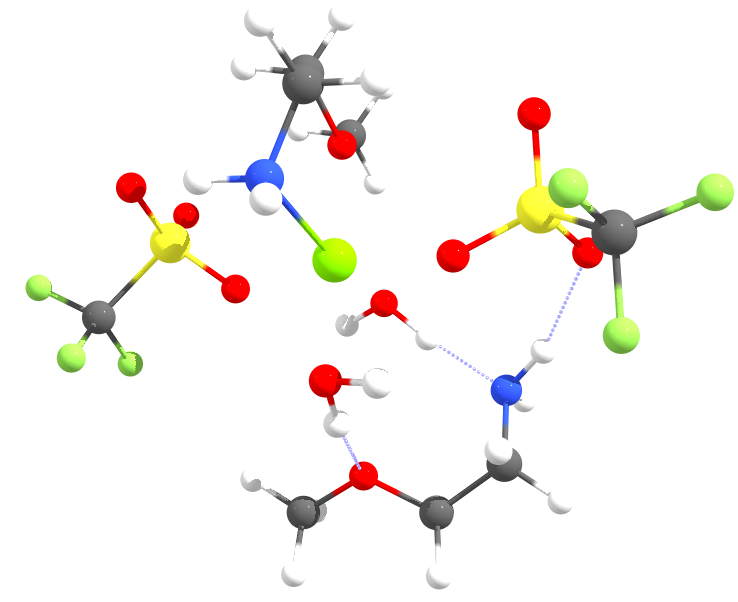


Method: (RI-)B3LYP(D3BJ)/def2-TZVPP

Symmetry: c1

Cartesian coordinates in Ångström:

C -6.8118726 2.2938816 -2.2810594

O -7.0146999 2.5624343 -0.8993786

H -5.8078226 2.5930730 -2.5962952

H -7.5513260 2.8248229 -2.8870834

H -6.9338969 1.2221509 -2.4199265

C -6.9138797 3.9385330 -0.5515722

H -5.8948912 4.2954593 -0.7413764

C -7.2626667 4.1335809 0.9123963

H -7.5955185 4.5188312 -1.1859471

H -6.6549615 3.4676152 1.5257193

N -8.6706513 3.8417474 1.1945662

H -6.9793487 5.1553046 1.1882742

Mg -8.3226279 -0.1496848 1.4584162

H -9.2704108 4.5846622 0.8558746

H -8.8256786 3.7499762 2.1990096

H -11.6707796 -1.6373887 1.1552362

C -11.3952269 -0.8601584 1.8696344

H -12.0578240 -0.8821752 2.7365601

H -10.0149540 -3.1035210 2.3261460

H -11.4435992 0.1124897 1.3925356

H -7.4992581 -2.6555353 1.7000501

C -9.8326420 -2.2674507 3.0068251

O -10.0434337 -1.0423928 2.2986906

N -7.4927667 -1.9236225 2.4097399

H -10.5294870 -2.3234124 3.8459285

C -8.4057323 -2.2718942 3.5169799

H -8.1903577 -3.2522551 3.9495513

H -6.5449336 -1.8234468 2.7500892

H -8.3004918 -1.5181882 4.2950474

F -6.1379002 2.7696824 4.6093586

F -6.3498744 0.8386250 5.5794367

C -6.9903168 1.9815927 5.2912064

F -7.3057570 2.5916908 6.4322514

S -8.5080464 1.6414493 4.2760444

O -7.8818418 1.0203121 3.0848478

O -9.0544341 2.9523144 3.9990464

O -9.2981552 0.7340022 5.0621218

F -8.4085161 -1.5976044 -3.2415342

F -8.4481109 -3.7496366 -2.9514675

C -8.1134533 -2.5891886 -2.3858330

F -6.7890108 -2.5799392 -2.1824204

S -9.0247179 -2.3564282 -0.7843024

O -10.4204224 -2.3740200 -1.1299357

O -8.5277595 -1.0189254 -0.3799533

O -8.5375001 -3.4162979 0.0765987

O -6.4215764 0.5884455 0.8683608

H -6.0990148 1.0336010 1.6593353

H -6.5279929 1.2914972 0.1925867

O -9.4518004 1.3431301 0.6099586

H -9.1830619 2.3070746 0.7425039

H -9.5279071 1.1767668 -0.3338326

SCF energy GEOOPT = -2775.575319210 H

ZPE = 968.6 kJ/mol

FREEH energy = 1065.80 kJ/mol

FREEH entropy = 0.99799 kJ/mol/K

$vibrational spectrum

# mode symmetry wave number IR intensity selection rules

# cm**(-1) km/mol IR RAMAN

1 -0.00 0.00000 - -

2 0.00 0.00000 - -

3 0.00 0.00000 - -

4 0.00 0.00000 - -

5 0.00 0.00000 - -

6 0.00 0.00000 - -

7 a 6.60 0.50846 YES YES

8 a 9.95 0.85907 YES YES

9 a 16.59 0.25457 YES YES

10 a 17.53 1.20524 YES YES

11 a 32.08 1.83852 YES YES

12 a 38.77 0.28588 YES YES

13 a 45.22 1.16089 YES YES

14 a 49.44 2.55322 YES YES

15 a 57.95 0.81615 YES YES

16 a 62.79 2.01353 YES YES

17 a 69.32 2.02817 YES YES

18 a 78.35 0.72029 YES YES

19 a 83.24 2.53754 YES YES

20 a 85.23 1.44563 YES YES

21 a 88.63 1.12146 YES YES

22 a 101.30 0.48808 YES YES

23 a 108.06 6.82279 YES YES

24 a 115.42 8.48808 YES YES

25 a 130.36 8.37625 YES YES

26 a 140.50 2.36013 YES YES

27 a 145.92 0.69110 YES YES

28 a 153.30 7.62666 YES YES

29 a 158.18 6.81952 YES YES

30 a 169.51 10.99654 YES YES

31 a 174.88 4.50947 YES YES

32 a 196.10 0.31386 YES YES

33 a 202.18 1.33153 YES YES

34 a 203.72 2.05801 YES YES

35 a 211.24 14.26304 YES YES

36 a 215.07 2.90312 YES YES

37 a 231.87 8.24905 YES YES

38 a 235.43 3.52850 YES YES

39 a 240.25 10.57598 YES YES

40 a 246.91 4.13116 YES YES

41 a 272.42 37.17044 YES YES

42 a 287.26 17.82290 YES YES

43 a 295.12 7.51290 YES YES

44 a 302.19 9.34753 YES YES

45 a 311.09 0.35344 YES YES

46 a 317.79 5.46840 YES YES

47 a 332.65 8.46880 YES YES

48 a 343.46 0.51884 YES YES

49 a 344.37 0.66036 YES YES

50 a 354.29 27.90239 YES YES

51 a 366.62 16.87342 YES YES

52 a 380.40 3.91557 YES YES

53 a 397.04 14.69397 YES YES

54 a 415.17 38.55529 YES YES

55 a 429.11 86.62999 YES YES

56 a 441.71 56.10613 YES YES

57 a 464.09 68.10815 YES YES

58 a 492.26 22.74320 YES YES

59 a 508.64 44.02495 YES YES

60 a 512.70 32.81680 YES YES

61 a 516.09 18.22662 YES YES

62 a 518.74 13.50541 YES YES

63 a 527.41 14.93840 YES YES

64 a 557.65 47.35368 YES YES

65 a 567.10 2.73909 YES YES

66 a 569.12 12.90457 YES YES

67 a 580.71 14.60613 YES YES

68 a 583.35 29.81057 YES YES

69 a 596.96 46.07255 YES YES

70 a 633.80 348.53768 YES YES

71 a 638.83 146.88287 YES YES

72 a 653.62 31.24375 YES YES

73 a 721.27 220.29232 YES YES

74 a 754.72 3.85644 YES YES

75 a 757.33 4.61345 YES YES

76 a 799.51 64.60480 YES YES

77 a 828.37 19.44417 YES YES

78 a 853.89 8.10169 YES YES

79 a 894.65 20.50218 YES YES

80 a 910.97 18.60541 YES YES

81 a 959.43 80.07276 YES YES

82 a 994.59 87.92147 YES YES

83 a 1023.96 305.64551 YES YES

84 a 1027.09 163.01234 YES YES

85 a 1032.39 111.13231 YES YES

86 a 1051.00 21.94945 YES YES

87 a 1062.61 18.09427 YES YES

88 a 1070.61 24.68365 YES YES

89 a 1095.06 67.85274 YES YES

90 a 1103.47 81.60529 YES YES

91 a 1110.13 97.65886 YES YES

92 a 1118.31 82.63552 YES YES

93 a 1132.34 112.93440 YES YES

94 a 1146.87 196.09836 YES YES

95 a 1157.13 151.19848 YES YES

96 a 1176.25 98.90339 YES YES

97 a 1177.83 85.18923 YES YES

98 a 1179.50 0.58508 YES YES

99 a 1190.05 0.30159 YES YES

100 a 1199.14 230.26249 YES YES

101 a 1201.36 198.14269 YES YES

102 a 1210.35 6.11686 YES YES

103 a 1230.60 77.63639 YES YES

104 a 1234.31 279.90496 YES YES

105 a 1238.45 107.17924 YES YES

106 a 1240.95 196.49988 YES YES

107 a 1241.77 37.38280 YES YES

108 a 1307.22 9.48489 YES YES

109 a 1313.21 32.44855 YES YES

110 a 1330.75 280.77622 YES YES

111 a 1336.36 265.60939 YES YES

112 a 1342.90 80.91072 YES YES

113 a 1346.00 25.65191 YES YES

114 a 1411.03 10.74254 YES YES

115 a 1415.96 7.24475 YES YES

116 a 1436.50 6.24252 YES YES

117 a 1448.69 2.57686 YES YES

118 a 1482.56 1.17489 YES YES

119 a 1484.35 0.64690 YES YES

120 a 1493.04 8.36076 YES YES

121 a 1497.59 7.49672 YES YES

122 a 1499.46 5.04074 YES YES

123 a 1502.25 9.72338 YES YES

124 a 1506.28 9.55546 YES YES

125 a 1516.07 5.57203 YES YES

126 a 1518.17 2.52773 YES YES

127 a 1520.39 8.12670 YES YES

128 a 1650.80 32.78673 YES YES

129 a 1669.67 24.10387 YES YES

130 a 1684.59 78.14525 YES YES

131 a 1689.74 47.07721 YES YES

132 a 2891.35 1752.03863 YES YES

133 a 2980.90 43.85371 YES YES

134 a 3002.18 54.34696 YES YES

135 a 3005.39 20.82514 YES YES

136 a 3018.71 47.36336 YES YES

137 a 3022.80 2.97500 YES YES

138 a 3029.67 37.93679 YES YES

139 a 3034.91 62.20741 YES YES

140 a 3058.05 30.53259 YES YES

141 a 3070.15 20.21581 YES YES

142 a 3083.59 23.87194 YES YES

143 a 3096.36 14.67021 YES YES

144 a 3114.13 3.20200 YES YES

145 a 3131.11 7.53500 YES YES

146 a 3166.49 2.97748 YES YES

147 a 3407.62 205.30660 YES YES

148 a 3428.22 108.93921 YES YES

149 a 3457.40 899.03140 YES YES

150 a 3542.62 38.09536 YES YES

151 a 3561.05 25.52712 YES YES

152 a 3826.88 90.93723 YES YES

153 a 3852.87 65.47619 YES YES

$end

Total COSMO energy + OC correction = -2775.6021394595 H

**[Mg(MOEA)(H_2_O)(OH)(TfO)_2_]^−^**


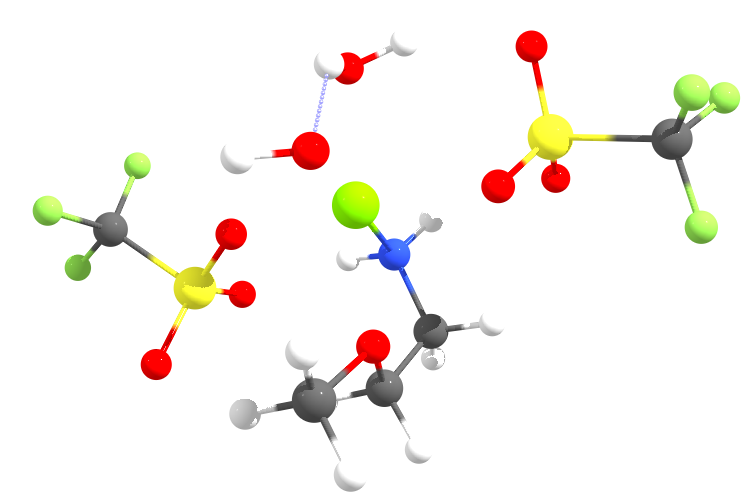


Method: (RI-)B3LYP(D3BJ)/def2-TZVPP

Symmetry: c1

Cartesian coordinates in Ångström:

Mg -8.2539327 -0.0020231 1.5830002

H -11.5517319 -1.0154203 1.2026543

C -11.2654584 -0.4487154 2.0908376

H -11.9979772 -0.6018264 2.8890738

H -10.1455217 -2.8284868 1.9712125

H -11.1819049 0.6076451 1.8492585

H -7.4806563 -2.6217749 1.4125047

C -9.8575973 -2.2370972 2.8448483

O -9.9732621 -0.8503138 2.5402072

N -7.4948885 -1.9994496 2.2165782

H -10.5318450 -2.4880537 3.6710663

C -8.4185117 -2.5020859 3.2468200

H -8.3011462 -3.5745931 3.4322686

H -6.5640600 -1.9360298 2.6131424

H -8.1944155 -1.9714147 4.1699285

F -7.2558028 0.8026375 6.4642228

F -6.7849405 2.6887481 5.5002715

C -6.3657769 1.4299897 5.6776532

F -5.1949718 1.4711034 6.3340190

S -6.1735240 0.5439233 4.0556860

O -7.5374534 0.5984194 3.5180761

O -5.7380347 -0.7868647 4.4302721

O -5.2148272 1.3471654 3.3208753

F -9.4614225 -1.1332025 -3.1191394

F -9.1396907 -3.2784549 -3.1822059

C -8.8141630 -2.1496155 -2.5297563

F -7.4985838 -1.9495631 -2.6839572

S -9.2708598 -2.2822709 -0.7330387

O -10.7010895 -2.4892770 -0.7412522

O -8.8518718 -0.9746197 -0.2180981

O -8.4666265 -3.3904531 -0.2469796

O -6.4988172 0.9469010 0.7426860

H -5.9036773 1.2646239 1.4385469

H -7.2258300 1.6242602 0.6594146

O -8.9516795 1.7225718 1.0376257

H -9.4667377 1.7675473 0.2317870

SCF energy GEOOPT = -2525.354231270 H

ZPE = 596.8 kJ/mol

FREEH energy = 672.38 kJ/mol

FREEH entropy = 0.80920 kJ/mol/K

$vibrational spectrum

# mode symmetry wave number IR intensity selection rules

# cm**(-1) km/mol IR RAMAN

1 -0.00 0.00000 - -

2 -0.00 0.00000 - -

3 0.00 0.00000 - -

4 0.00 0.00000 - -

5 0.00 0.00000 - -

6 0.00 0.00000 - -

7 a 18.48 0.46326 YES YES

8 a 19.36 0.65948 YES YES

9 a 27.89 1.34400 YES YES

10 a 40.94 0.35309 YES YES

11 a 44.20 0.17727 YES YES

12 a 46.90 0.55575 YES YES

13 a 68.47 0.58168 YES YES

14 a 74.47 2.56223 YES YES

15 a 89.58 1.63876 YES YES

16 a 91.26 2.69456 YES YES

17 a 96.14 1.63364 YES YES

18 a 102.53 2.45691 YES YES

19 a 118.45 0.74542 YES YES

20 a 138.53 1.00740 YES YES

21 a 146.52 5.88503 YES YES

22 a 158.12 4.24122 YES YES

23 a 164.43 10.54726 YES YES

24 a 184.20 8.26660 YES YES

25 a 194.68 16.19639 YES YES

26 a 206.75 1.23565 YES YES

27 a 208.88 2.88691 YES YES

28 a 211.94 18.69422 YES YES

29 a 228.62 19.42400 YES YES

30 a 234.55 8.72958 YES YES

31 a 247.35 8.24652 YES YES

32 a 257.80 19.73905 YES YES

33 a 263.94 28.42744 YES YES

34 a 296.34 1.44755 YES YES

35 a 306.41 16.88791 YES YES

36 a 310.79 5.61064 YES YES

37 a 318.88 10.42883 YES YES

38 a 334.98 37.18773 YES YES

39 a 344.04 0.43453 YES YES

40 a 348.66 12.71049 YES YES

41 a 361.31 2.49601 YES YES

42 a 377.04 28.45303 YES YES

43 a 400.81 107.48382 YES YES

44 a 404.54 16.54519 YES YES

45 a 507.67 30.94255 YES YES

46 a 511.70 34.32559 YES YES

47 a 513.43 4.90884 YES YES

48 a 514.58 18.56090 YES YES

49 a 533.26 128.44373 YES YES

50 a 540.45 117.70442 YES YES

51 a 564.79 6.78291 YES YES

52 a 566.05 9.17988 YES YES

53 a 570.81 32.38262 YES YES

54 a 573.92 2.28716 YES YES

55 a 578.53 0.66147 YES YES

56 a 620.56 93.92676 YES YES

57 a 628.75 468.27815 YES YES

58 a 639.18 32.57442 YES YES

59 a 683.49 87.58209 YES YES

60 a 748.92 47.77974 YES YES

61 a 753.40 8.17396 YES YES

62 a 776.77 131.52096 YES YES

63 a 847.49 11.42921 YES YES

64 a 902.99 14.21886 YES YES

65 a 978.03 155.62723 YES YES

66 a 1004.71 96.43811 YES YES

67 a 1030.74 453.77459 YES YES

68 a 1036.25 116.10135 YES YES

69 a 1047.16 11.52028 YES YES

70 a 1079.55 16.06643 YES YES

71 a 1111.58 127.83434 YES YES

72 a 1129.37 42.31908 YES YES

73 a 1148.94 195.08720 YES YES

74 a 1149.14 79.77690 YES YES

75 a 1160.89 106.81715 YES YES

76 a 1160.94 210.68945 YES YES

77 a 1193.51 1.44743 YES YES

78 a 1212.02 58.14916 YES YES

79 a 1212.28 83.68045 YES YES

80 a 1228.99 16.26340 YES YES

81 a 1241.56 94.89463 YES YES

82 a 1245.44 378.73182 YES YES

83 a 1250.29 308.54286 YES YES

84 a 1293.89 276.69647 YES YES

85 a 1301.27 337.83447 YES YES

86 a 1311.26 93.80860 YES YES

87 a 1351.32 4.56882 YES YES

88 a 1410.96 7.30023 YES YES

89 a 1443.52 2.52932 YES YES

90 a 1474.70 3.25956 YES YES

91 a 1499.51 5.60983 YES YES

92 a 1501.01 2.39614 YES YES

93 a 1512.33 5.19147 YES YES

94 a 1532.32 6.06937 YES YES

95 a 1647.27 40.13593 YES YES

96 a 1647.83 31.91650 YES YES

97 a 2997.00 4.59837 YES YES

98 a 3003.98 90.55312 YES YES

99 a 3014.15 111.32001 YES YES

100 a 3049.03 14.59986 YES YES

101 a 3075.70 14.08021 YES YES

102 a 3111.78 10.51395 YES YES

103 a 3138.57 1.80777 YES YES

104 a 3195.30 527.66187 YES YES

105 a 3450.20 62.10423 YES YES

106 a 3543.43 120.00761 YES YES

107 a 3699.32 295.90051 YES YES

108 a 3888.98 2.64411 YES YES

$end

Total COSMO energy + OC correction = -2525.4219177792 H

**[Mg(MOEA)_2_(H_2_O)(OH)(TfO)]**


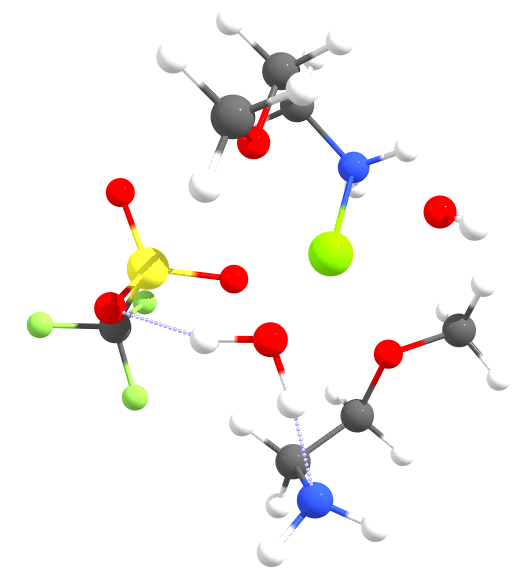


Method: (RI-)B3LYP(D3BJ)/def2-TZVPP

Symmetry: c1

Cartesian coordinates in Ångström:

C -5.9871910 -0.6011153 -0.5020086

O -6.6264723 0.4321332 0.2552146

H -5.1584002 -1.0335588 0.0674621

H -5.5995081 -0.1887631 -1.4370309

H -6.7595416 -1.3401892 -0.7059491

C -5.7645820 1.5410213 0.5028349

H -5.0539720 1.2908923 1.2965948

C -6.5299958 2.7859122 0.8965198

H -5.1946918 1.7336747 -0.4140471

H -7.0041686 2.6379874 1.8614520

N -7.5746450 3.1281463 -0.0830646

H -5.7850557 3.5799635 1.0182882

Mg -8.4006642 -0.3479457 1.3337097

H -7.2336192 3.0494171 -1.0340211

H -7.8835299 4.0835719 0.0476453

H -11.6206543 -1.4091039 1.3902042

C -11.3525868 -0.8089545 2.2630665

H -12.0113731 -1.0459111 3.1013611

H -10.0782586 -3.0622690 2.1825166

H -11.4349745 0.2443152 2.0178101

H -7.6705512 -2.7874916 1.3567876

C -9.7728615 -2.4116933 3.0092833

O -9.9983902 -1.0555320 2.6283704

N -7.4928016 -2.1926443 2.1614464

H -10.3746373 -2.6442552 3.8919776

C -8.3029310 -2.5939919 3.3231452

H -8.1416612 -3.6364642 3.6158724

H -6.5075143 -2.2165915 2.3867282

H -8.0344290 -1.9546890 4.1618937

F -6.5622834 3.1497805 4.1731486

F -5.9773966 1.4764009 5.4243986

C -7.0218254 2.2123441 5.0238483

F -7.5367870 2.8340912 6.0856816

S -8.3057818 1.1391902 4.2113574

O -7.5423606 0.5473382 3.0926904

O -9.3159484 2.0800942 3.7468483

O -8.6880747 0.2133086 5.2425447

O -8.8640186 -1.5112010 -0.1151697

H -9.4324972 -1.3105260 -0.8559926

O -9.4383267 1.4311637 1.0500092

H -8.9093680 2.0574177 0.4954437

H -9.5377498 1.8558859 1.9241182

SCF energy GEOOPT = -1813.397032632 H

ZPE = 859.0 kJ/mol

FREEH energy = 931.67 kJ/mol

FREEH entropy = 0.76677 kJ/mol/K

$vibrational spectrum

# mode symmetry wave number IR intensity selection rules

# cm**(-1) km/mol IR RAMAN

1 -0.00 0.00000 - -

2 -0.00 0.00000 - -

3 -0.00 0.00000 - -

4 0.00 0.00000 - -

5 0.00 0.00000 - -

6 0.00 0.00000 - -

7 a 14.87 0.42918 YES YES

8 a 35.22 0.31114 YES YES

9 a 44.36 0.83258 YES YES

10 a 50.40 2.10569 YES YES

11 a 64.30 5.11266 YES YES

12 a 70.31 2.08881 YES YES

13 a 73.54 1.21121 YES YES

14 a 77.37 1.62024 YES YES

15 a 91.48 2.04544 YES YES

16 a 101.42 3.20352 YES YES

17 a 111.78 4.61521 YES YES

18 a 122.49 3.22241 YES YES

19 a 137.02 1.26152 YES YES

20 a 148.64 5.82353 YES YES

21 a 166.51 3.60926 YES YES

22 a 175.65 21.80712 YES YES

23 a 182.37 0.66712 YES YES

24 a 195.85 6.11481 YES YES

25 a 201.23 1.23563 YES YES

26 a 204.64 16.31536 YES YES

27 a 219.46 1.86881 YES YES

28 a 226.73 2.91939 YES YES

29 a 238.49 1.89988 YES YES

30 a 254.07 6.61446 YES YES

31 a 264.96 15.17182 YES YES

32 a 270.18 4.10009 YES YES

33 a 287.34 67.72792 YES YES

34 a 294.78 10.68171 YES YES

35 a 300.30 10.57741 YES YES

36 a 309.89 2.62867 YES YES

37 a 317.36 5.44462 YES YES

38 a 334.01 24.92719 YES YES

39 a 341.72 51.10774 YES YES

40 a 348.61 9.03852 YES YES

41 a 368.95 9.88335 YES YES

42 a 385.62 14.31141 YES YES

43 a 398.69 20.08135 YES YES

44 a 424.15 32.95937 YES YES

45 a 500.60 6.47575 YES YES

46 a 510.41 18.22726 YES YES

47 a 517.22 54.45451 YES YES

48 a 526.84 187.79849 YES YES

49 a 533.35 10.06871 YES YES

50 a 566.55 17.92852 YES YES

51 a 569.35 25.65419 YES YES

52 a 580.39 6.63596 YES YES

53 a 597.23 30.15804 YES YES

54 a 636.83 206.78836 YES YES

55 a 692.69 46.60547 YES YES

56 a 754.14 6.26327 YES YES

57 a 817.58 9.73245 YES YES

58 a 840.50 45.01324 YES YES

59 a 848.68 89.14297 YES YES

60 a 877.51 76.78307 YES YES

61 a 904.47 53.41221 YES YES

62 a 927.05 15.59932 YES YES

63 a 957.40 171.27182 YES YES

64 a 982.71 199.53688 YES YES

65 a 995.44 31.27693 YES YES

66 a 1018.45 229.30825 YES YES

67 a 1024.13 20.41721 YES YES

68 a 1075.59 49.43690 YES YES

69 a 1079.40 47.13516 YES YES

70 a 1081.38 13.98200 YES YES

71 a 1099.18 27.12718 YES YES

72 a 1124.29 103.87627 YES YES

73 a 1124.87 93.34564 YES YES

74 a 1148.49 196.80846 YES YES

75 a 1172.11 44.24607 YES YES

76 a 1179.15 0.92453 YES YES

77 a 1184.65 4.93885 YES YES

78 a 1199.92 227.27597 YES YES

79 a 1215.56 52.23418 YES YES

80 a 1223.28 147.12187 YES YES

81 a 1224.43 113.72455 YES YES

82 a 1235.13 0.86865 YES YES

83 a 1235.25 6.11903 YES YES

84 a 1308.12 11.05368 YES YES

85 a 1313.81 38.54759 YES YES

86 a 1323.56 193.87955 YES YES

87 a 1326.53 2.88894 YES YES

88 a 1330.51 127.51411 YES YES

89 a 1414.11 6.42910 YES YES

90 a 1419.72 8.64393 YES YES

91 a 1446.58 4.45758 YES YES

92 a 1448.47 6.17833 YES YES

93 a 1464.42 7.25973 YES YES

94 a 1482.09 0.82098 YES YES

95 a 1491.10 8.50369 YES YES

96 a 1494.01 7.77031 YES YES

97 a 1500.29 4.82638 YES YES

98 a 1507.88 9.15236 YES YES

99 a 1512.90 0.39874 YES YES

100 a 1513.55 9.55381 YES YES

101 a 1523.08 10.98976 YES YES

102 a 1526.21 5.11354 YES YES

103 a 1648.86 27.99733 YES YES

104 a 1649.23 49.91450 YES YES

105 a 1700.58 54.80294 YES YES

106 a 2983.70 9.08361 YES YES

107 a 2993.55 81.68046 YES YES

108 a 3001.01 6.67388 YES YES

109 a 3005.99 84.59902 YES YES

110 a 3013.11 68.24361 YES YES

111 a 3018.33 69.12224 YES YES

112 a 3036.71 34.98113 YES YES

113 a 3051.31 35.29649 YES YES

114 a 3055.01 39.68188 YES YES

115 a 3076.88 25.66197 YES YES

116 a 3113.85 0.98209 YES YES

117 a 3114.14 14.11474 YES YES

118 a 3150.55 0.20405 YES YES

119 a 3163.36 5.77681 YES YES

120 a 3272.22 963.17768 YES YES

121 a 3470.78 15.50442 YES YES

122 a 3496.78 1.48190 YES YES

123 a 3545.35 441.16551 YES YES

124 a 3574.23 7.65611 YES YES

125 a 3581.25 12.24155 YES YES

126 a 3920.98 5.86140 YES YES

$end

Total COSMO energy + OC correction = -1813.4208861126 H

**[Mg(MOEA)(OH)_2_(TfO)]^−^**


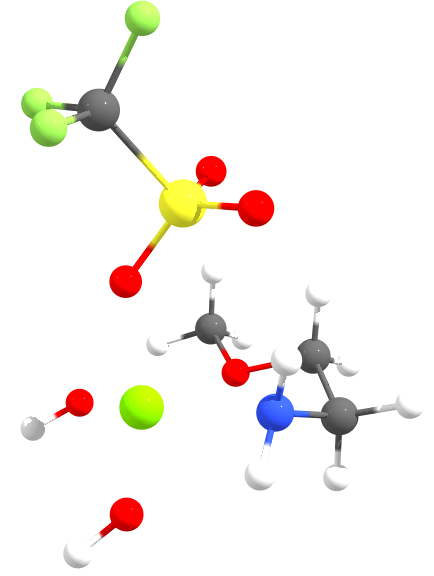


Method: (RI-)B3LYP(D3BJ)/def2-TZVPP

Symmetry: c1

Cartesian coordinates in Ångström:

Mg -7.9431260 -0.2057184 1.6323065

H -11.4155953 -0.9501732 1.6128236

C -10.9643740 -0.5193892 2.5094393

H -11.6151633 -0.6890391 3.3752614

H -10.1287774 -2.9753893 1.9750312

H -10.7659406 0.5398941 2.3620974

H -7.4639868 -2.9817535 1.1804333

C -9.7094261 -2.5234686 2.8801226

O -9.6866556 -1.1122089 2.7437687

N -7.4465656 -2.4274232 2.0270914

H -10.3381541 -2.8070421 3.7341879

C -8.2703080 -2.9664614 3.0952431

H -8.2400299 -4.0604601 3.1781663

H -6.5031477 -2.1662456 2.2913433

H -7.9156133 -2.5534660 4.0415175

F -9.8202956 -0.5535769 -2.9606055

F -9.7094304 -2.6829483 -3.3604560

C -9.1603206 -1.6795327 -2.6509288

F -7.8902670 -1.5387434 -3.0623238

S -9.2490308 -2.0427998 -0.8288460

O -10.6658536 -2.1457485 -0.5588019

O -8.6019636 -0.8557466 -0.2629938

O -8.4847842 -3.2690447 -0.6867203

O -6.0668913 -0.1501169 2.0674123

H -5.6235106 0.6853354 2.2088460

O -8.8679670 1.4534910 1.8344559

H -8.3908516 2.2773556 1.7509784

SCF energy GEOOPT = -1563.155770402 H

ZPE = 480.4 kJ/mol

FREEH energy = 535.05 kJ/mol

FREEH entropy = 0.64109 kJ/mol/K

$vibrational spectrum

# mode symmetry wave number IR intensity selection rules

# cm**(-1) km/mol IR RAMAN

1 -0.00 0.00000 - -

2 -0.00 0.00000 - -

3 0.00 0.00000 - -

4 0.00 0.00000 - -

5 0.00 0.00000 - -

6 0.00 0.00000 - -

7 a 17.00 2.08267 YES YES

8 a 30.94 0.63370 YES YES

9 a 54.99 0.45435 YES YES

10 a 56.44 5.30592 YES YES

11 a 65.82 4.28670 YES YES

12 a 79.88 0.63255 YES YES

13 a 94.55 0.23244 YES YES

14 a 103.83 43.54114 YES YES

15 a 128.52 0.97168 YES YES

16 a 143.70 1.71898 YES YES

17 a 159.67 3.39655 YES YES

18 a 174.40 2.84408 YES YES

19 a 202.12 1.99920 YES YES

20 a 205.83 3.44291 YES YES

21 a 213.51 27.29811 YES YES

22 a 218.78 31.72942 YES YES

23 a 235.86 26.86764 YES YES

24 a 248.31 16.32737 YES YES

25 a 262.97 11.78574 YES YES

26 a 285.31 0.12082 YES YES

27 a 308.68 6.19601 YES YES

28 a 319.54 13.60941 YES YES

29 a 330.53 142.75256 YES YES

30 a 343.18 0.11675 YES YES

31 a 371.41 22.74618 YES YES

32 a 396.82 12.09038 YES YES

33 a 489.78 169.09207 YES YES

34 a 505.30 17.48279 YES YES

35 a 511.20 123.68106 YES YES

36 a 518.47 86.42761 YES YES

37 a 534.65 24.74376 YES YES

38 a 565.15 3.98261 YES YES

39 a 573.60 21.73625 YES YES

40 a 577.81 37.60550 YES YES

41 a 611.62 70.56170 YES YES

42 a 626.81 186.19620 YES YES

43 a 658.43 100.33627 YES YES

44 a 750.80 9.85785 YES YES

45 a 836.70 53.16023 YES YES

46 a 874.23 192.31762 YES YES

47 a 921.39 2.78381 YES YES

48 a 1021.39 1.12703 YES YES

49 a 1034.87 241.19481 YES YES

50 a 1035.73 86.60031 YES YES

51 a 1099.10 38.88804 YES YES

52 a 1131.53 77.83196 YES YES

53 a 1141.71 141.77129 YES YES

54 a 1154.61 172.40409 YES YES

55 a 1191.28 0.34947 YES YES

56 a 1211.50 71.21046 YES YES

57 a 1217.83 3.94530 YES YES

58 a 1241.63 5.59037 YES YES

59 a 1252.54 399.31940 YES YES

60 a 1289.66 31.57274 YES YES

61 a 1301.98 281.29491 YES YES

62 a 1345.14 3.98487 YES YES

63 a 1400.67 11.01394 YES YES

64 a 1420.21 3.98876 YES YES

65 a 1467.03 9.13725 YES YES

66 a 1492.96 1.44520 YES YES

67 a 1495.51 5.56784 YES YES

68 a 1516.60 4.41960 YES YES

69 a 1527.42 4.27482 YES YES

70 a 1667.63 16.95155 YES YES

71 a 2967.03 7.24317 YES YES

72 a 2976.52 77.57379 YES YES

73 a 2983.95 231.98483 YES YES

74 a 3026.76 7.96453 YES YES

75 a 3061.99 28.73015 YES YES

76 a 3063.37 32.07053 YES YES

77 a 3126.35 12.73417 YES YES

78 a 3472.48 63.16999 YES YES

79 a 3571.30 121.63486 YES YES

80 a 3896.43 0.68522 YES YES

81 a 3906.94 0.82629 YES YES

$end

Total COSMO energy + OC correction = -1563.2353639080 H

**[Mg(MOEA)(H_2_O)(OH)(TfO)]**


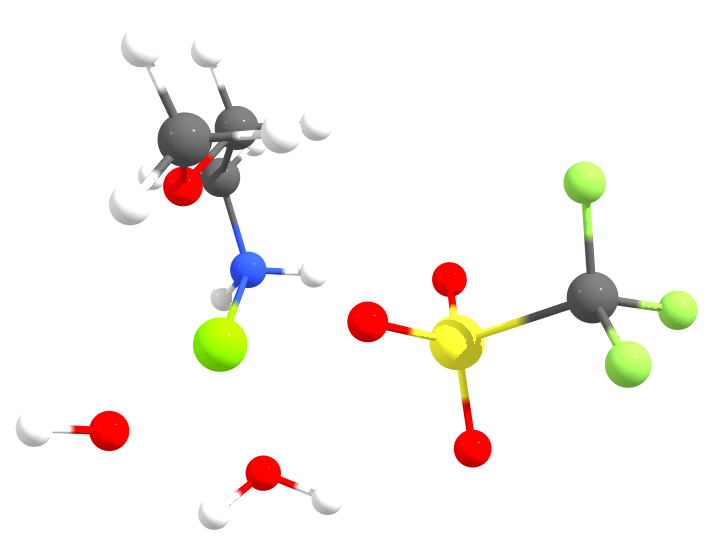


Method: (RI-)B3LYP(D3BJ)/def2-TZVPP

Symmetry: c1

Cartesian coordinates in Ångström:

Mg -8.2129808 -0.0882446 1.7048973

H -11.3516749 -1.0427364 1.1227950

C -11.2033788 -0.6261126 2.1204288

H -11.9871037 -0.9748469 2.7955556

H -9.8122046 -2.8658945 1.7409069

H -11.2142395 0.4593594 2.0701777

H -7.1626663 -2.4703020 1.6459694

C -9.7313886 -2.4140565 2.7340721

O -9.9206352 -1.0003094 2.6287279

N -7.3529735 -1.9310841 2.4917855

H -10.4996303 -2.8406655 3.3838554

C -8.3516113 -2.6330304 3.3221999

H -8.1568785 -3.7053035 3.3969933

H -6.4749856 -1.8252639 2.9847958

H -8.3190979 -2.2100739 4.3261496

F -10.2580395 -2.8537717 -1.7218574

F -8.6365752 -2.9577030 -3.1631366

C -9.2316810 -2.1792172 -2.2596454

F -9.7122745 -1.0951099 -2.8713463

S -8.0227744 -1.7003515 -0.9352134

O -7.6146895 -2.9498734 -0.3250809

O -8.8777711 -0.8872910 -0.0339994

O -6.9990974 -0.9255471 -1.5973972

O -6.3719575 0.5046716 0.8376790

H -6.5975110 1.4317491 1.0341576

H -6.3190719 0.3809873 -0.1219390

O -8.2570338 1.6939206 2.2006925

H -8.7595736 2.2836111 2.7535762

SCF energy GEOOPT = -1563.706239605 H

ZPE = 519.9 kJ/mol

FREEH energy = 573.73 kJ/mol

FREEH entropy = 0.62648 kJ/mol/K

$vibrational spectrum

# mode symmetry wave number IR intensity selection rules

# cm**(-1) km/mol IR RAMAN

1 -0.00 0.00000 - -

2 -0.00 0.00000 - -

3 -0.00 0.00000 - -

4 -0.00 0.00000 - -

5 0.00 0.00000 - -

6 0.00 0.00000 - -

7 a 26.87 1.26325 YES YES

8 a 38.35 0.71162 YES YES

9 a 54.29 0.89549 YES YES

10 a 64.11 0.97466 YES YES

11 a 75.49 6.35037 YES YES

12 a 85.19 1.45696 YES YES

13 a 107.72 2.73267 YES YES

14 a 114.11 5.01310 YES YES

15 a 137.42 7.14892 YES YES

16 a 155.13 12.03038 YES YES

17 a 160.77 33.34145 YES YES

18 a 182.13 1.06446 YES YES

19 a 196.63 3.92094 YES YES

20 a 200.83 10.46413 YES YES

21 a 213.10 4.41896 YES YES

22 a 241.66 22.97947 YES YES

23 a 250.91 83.45968 YES YES

24 a 255.35 17.37327 YES YES

25 a 269.48 8.80329 YES YES

26 a 300.68 15.50322 YES YES

27 a 316.92 11.46926 YES YES

28 a 325.49 18.67972 YES YES

29 a 346.87 2.46377 YES YES

30 a 367.87 54.48889 YES YES

31 a 407.04 3.20356 YES YES

32 a 416.82 12.44802 YES YES

33 a 453.20 146.95794 YES YES

34 a 480.97 34.42508 YES YES

35 a 506.39 53.82056 YES YES

36 a 513.62 34.84967 YES YES

37 a 527.93 26.74314 YES YES

38 a 565.03 22.41275 YES YES

39 a 576.51 16.87203 YES YES

40 a 617.12 28.54298 YES YES

41 a 632.95 400.54226 YES YES

42 a 668.95 116.02933 YES YES

43 a 675.21 111.44207 YES YES

44 a 753.19 44.99773 YES YES

45 a 768.21 25.72294 YES YES

46 a 846.09 7.81785 YES YES

47 a 909.27 11.99173 YES YES

48 a 996.71 74.21025 YES YES

49 a 1020.35 293.46156 YES YES

50 a 1046.35 5.74007 YES YES

51 a 1072.27 36.33294 YES YES

52 a 1108.43 183.73904 YES YES

53 a 1116.64 30.43719 YES YES

54 a 1169.47 167.29818 YES YES

55 a 1178.96 42.61883 YES YES

56 a 1182.25 2.29103 YES YES

57 a 1201.67 285.44132 YES YES

58 a 1221.01 3.24573 YES YES

59 a 1229.43 240.47154 YES YES

60 a 1235.55 66.86675 YES YES

61 a 1306.26 48.35921 YES YES

62 a 1313.37 314.89020 YES YES

63 a 1353.67 6.25333 YES YES

64 a 1407.73 5.89350 YES YES

65 a 1437.74 3.65564 YES YES

66 a 1481.12 0.28738 YES YES

67 a 1495.37 9.21393 YES YES

68 a 1501.43 8.41390 YES YES

69 a 1502.73 2.53400 YES YES

70 a 1520.51 8.92823 YES YES

71 a 1591.52 79.60955 YES YES

72 a 1658.89 27.21800 YES YES

73 a 3018.93 10.98662 YES YES

74 a 3026.31 42.10268 YES YES

75 a 3038.80 32.35261 YES YES

76 a 3063.26 28.26513 YES YES

77 a 3092.55 33.27627 YES YES

78 a 3093.98 9.12172 YES YES

79 a 3142.84 6.01986 YES YES

80 a 3407.70 140.89223 YES YES

81 a 3556.17 34.13943 YES YES

82 a 3619.94 84.91334 YES YES

83 a 3733.48 329.86521 YES YES

84 a 3975.78 22.94222 YES YES

$end

Total COSMO energy + OC correction = -1563.7291104511 H

All thermodynamic values calculated for this work are summarized in **Table S1**–**Table S4** (tables) and **Scheme S1**–**Scheme S 7** (reaction schemes).

**Table S1**: Calculated reaction enthalpies Δ_r_H° and free reaction enthalpies Δ_r_G° in the gas phase and in a G2 solution (RI-B3LYP(D3BJ)/def2-TZVPP; ε_r_ = 7.23).^[9]^

| **Reaction** | **Δ_r_*H*° (gas phase) [kJ mol^−1^]** | **Δ_r_*G*° (gas phase) [kJ mol^−1^]** | **Δ_r_*G*° (solution) [kJ mol^−1^]** |
| --- | --- | --- | --- |
| [Al(Ohfip)_4_]^−^ + MOEA 🡪 [Al(Ohfip)_3_(MOEA^−H^)]^−^ + HOhfip | +100 | +103 | +65 |
| [Al(Ohfip)_4_]^−^ + H_2_O 🡪 [Al(Ohfip)_3_(OH)]^−^ + HOhfip | +48 | +43 | −119 |
| MOEA + H_2_O 🡪 [MOEA–H]^+^ + [OH]^−^ | +745 | +750 | +120 |
| 2 H_2_O + MOEA 🡪 OH^−^ + [(H_2_O)∙∙∙H-MOEA]^+^ | +678 | +720 | +6 |
| [Mg(MOEA)_3_]^2+^ + 2 G2 🡪 [Mg(G2)_2_]^2+^ + 3 MOEA | +43 | +4 | +11 |
| [Mg(G2)_2_]^2+^ + e^−^ 🡪 [Mg(G2)_2_]^⋅+^ (single point calculation on structure of dication) | −422 | −422 | −72 |
| [Mg(MOEA)_3_]^2+^ + e^−^ 🡪 [Mg(MOEA)_3_]^⋅+^ (single point calculation on structure of dication) | −524 | −524 | −149 |
| [Mg(MOEA)_3_]^2+^ + 2 [Ohfip]^−^ 🡪 [Mg(MOEA)_2_(Ohfip)_2_] + MOEA | −1178 | −1136 | −269 |

**Table S2**: Calculated reaction enthalpies Δ_r_H° and free reaction enthalpies Δ_r_G° in the gas phase and in a G2 solution (RI-B3LYP(D3BJ)/def2-TZVPP; ε_r_ = 7.23).

| **Reaction** | **Δ_r_*H*° (gas phase) [kJ mol^−1^]** | **Δ_r_*G*° (gas phase) [kJ mol^−1^]** | **Δ_r_*G*° (solution) [kJ mol^−1^]** |
| --- | --- | --- | --- |
| [Al(Ohfip)_4_]^−^ + OH^−^ 🡪 [Al(Ohfip)_4_OH]^2−^ | −96 | −49 | −62 |
| [Al(Ohfip)_4_OH]^2−^ 🡪 [Al(Ohfip)_3_OH]^−^ + [Ohfip]^−^ | −94 | −149 | −46 |
| [Ohfip]^−^ + [(H_2_O)∙∙∙H-MOEA]^+^ 🡪 [(H_2_O)∙∙∙MOEA] + HOhfip | −469 | −471 | −121 |
| 2 [Al(Ohfip)_3_OH]^−^ 🡪 [(Ohfip)_3_Al(OH)_2_Al(Ohfip)_3_]^2−^ | +86 | +157 | +44 |
| [(Ohfip)_3_Al(OH)_2_Al(Ohfip)_3_]^2−^ + [Mg(MOEA)_3_]^2+^ 🡪 [(Ohfip)_6_Al_2_(OH)_2_Mg(MOEA)] + 2 MOEA | −881 | −929 | −127 |
|  |  |  |  |
| [Mg(MOEA)_2_(TfO)_2_] 🡪 [Mg(TfO)_2_] + 2 MOEA | +359 | +246 | +222 |
| [Mg(MOEA)_2_(TfO)_2_] 🡪 [Mg(MOEA)( TfO)_2_] + MOEA | +157 | +100 | +85 |
| [Mg(MOEA)_2_(TfO)_2_] + MOEA 🡪 [Mg(MOEA)_3_(TfO)_2_] | −5 | +52 | +43 |
| [Mg(MOEA)_2_(TfO)_2_] + MOEA 🡪 [Mg(MOEA)_3_]^2+^ + 2 [TfO]^−^ | +1046 | +1005 | +173 |
| [Mg(MOEA)_2_(TfO)_2_] + 2 G2 🡪 [Mg(G2)_2_]^2+^ + 2 MOEA + 2 [TfO]^−^ | +1089 | +1009 | +183 |
| [Mg(MOEA)_2_(TfO)_2_] + G2 🡪 [Mg(G2)( TfO)_2_] + 2 MOEA | +123 | +71 | +56 |

**Table S3**: Calculated reaction enthalpies Δ_r_H° and free reaction enthalpies Δ_r_G° in the gas phase and in a G2 solution (RI-B3LYP(D3BJ)/def2-TZVPP; ε_r_ = 7.23).

| **Reaction** | **Δ_r_*H*° (gas phase) [kJ mol^−1^]** | **Δ_r_*G*° (gas phase) [kJ mol^−1^]** | **Δ_r_*G*° (solution) [kJ mol^−1^]** |
| --- | --- | --- | --- |
| [MOEA(H_2_O)] + [Mg(MOEA)_3_]^2+^ 🡪 [HMOEA]^+^ + [Mg(MOEA)_3_(OH)]^+^ | −163 | −158 | +37 |
| [MOEA(H_2_O)] + [Mg(MOEA)_3_(OH)]^+^ 🡪 [HMOEA]^+^ + [Mg(MOEA)_3_(OH)_2_] | +193 | +200 | +109 |
| [MOEA(H_2_O)] + [Mg(MOEA)_3_]^2+^ 🡪 MOEA + [Mg(MOEA)_3_(H_2_O)]^2+^ | −37 | −28 | 0 |
| [Mg(MOEA)_3_(H_2_O)]^2+^ 🡪 [Mg(MOEA)_2_(OH)]^+^ + [HMOEA]^+^ | −35 | −98 | +23 |
| [Mg(MOEA)_2_(OH)]^+^ + [MOEA(H_2_O)] 🡪 [Mg(MOEA)_3_(OH)(H_2_O)]^+^ | −149 | −80 | −16 |
| [Mg(MOEA)_3_(OH)(H_2_O)]^+^ 🡪 [HMOEA]^+^ + [Mg(MOEA)_2_(OH)_2_] | +280 | +225 | +91 |
| [Mg(MOEA)_2_(OH)_2_] + MOEA 🡪 [Mg(MOEA)_2_(OH)_2_(MOEA)] | −23 | +23 | +39 |

**Table S4**: Calculated reaction enthalpies Δ_r_H° and free reaction enthalpies Δ_r_G° in the gas phase and in a G2 solution (RI-B3LYP(D3BJ)/def2-TZVPP; ε_r_ = 7.23).

| **Reaction** | **Δ_r_*H*° (gas phase) [kJ mol^−1^]** | **Δ_r_*G*° (gas phase) [kJ mol^−1^]** | **Δ_r_*G*° (solution) [kJ mol^−1^]** |
| --- | --- | --- | --- |
| [Mg(MOEA)_2_(TfO)_2_] + 2 [MOEA(H_2_O)] 🡪 2 MOEA + [Mg(MOEA)_2_(H_2_O)_2_(TfO)_2_] | −27 | −25 | −5 |
| [Mg(MOEA)_2_(H_2_O)_2_(TfO)_2_] 🡪 [HMOEA]^+^ + [Mg(MOEA)(H_2_O)(OH)( TfO)_2_]^−^ | +493 | +450 | +128 |
| [Mg(MOEA)(H_2_O)(OH)( TfO)_2_]^−^ + MOEA 🡪 [Mg(MOEA)_2_(H_2_O)(OH)( TfO)] + [TfO]^−^ | +68 | +76 | +16 |
| [Mg(MOEA)_2_(H_2_O)(OH)( TfO)] 🡪 [HMOEA]^+^ + [Mg(MOEA)(OH)_2_(TfO)]^−^ | +543 | +481 | +120 |
| [Mg(MOEA)(OH)_2_(TfO)]^−^ + MOEA 🡪 [TfO]^−^ + [Mg(MOEA)_2_(OH)_2_] | +29 | +39 | +11 |
| [Mg(MOEA)_2_(H_2_O)_2_(TfO)_2_] 🡪 [HMOEA]^+^ + [TfO]^−^ + [Mg(MOEA)(H_2_O)(OH)( TfO)] | +634 | +542 | +140 |

**Scheme S1**: Calculated reaction enthalpies Δ_r_H° and free reaction enthalpies Δ_r_G° in the gas phase and in a G2 solution for a reaction path describing the decomposition of the [Al(Ohfip)_4_]^−^ anion (RI-B3LYP(D3BJ)/def2-TZVPP; ε_r_ = 7.23). Note that a very small negative HOMO/LUMO gap is found for the [Mg(G2)_2_]^⋅+^ radical cation (0.01 eV).

**Scheme S2**: Calculated reaction enthalpies Δ_r_H° and free reaction enthalpies Δ_r_G° in the gas phase and in a G2 solution for a reaction path describing the decomposition of the [Al(Ohfip)_4_]^−^ anion (RI-B3LYP(D3BJ)/def2-TZVPP; ε_r_ = 7.23).

**Scheme S3**: Calculated reaction enthalpies Δ_r_H° and free reaction enthalpies Δ_r_G° in the gas phase and in a G2 solution. The reaction equations are designed to compare the stability of different complexes in a G2/MOEA solution of Mg[O_3_SCF_3_]_2_ (RI-B3LYP(D3BJ)/def2-TZVPP; ε_r_ = 7.23).

**Scheme S4**: Calculated reaction enthalpies Δ_r_H° and free reaction enthalpies Δ_r_G° in the gas phase and in a G2 solution. The reaction equations are designed to compare the stability of different complexes in a G2/MOEA solution of Mg[O_3_SCF_3_]_2_ (RI-B3LYP(D3BJ)/def2-TZVPP; ε_r_ = 7.23).

*2.2 Löwdin Spin Population Analysis for [Mg(G2)_2_]^⋅+^ and [Mg(MOEA)_3_]^⋅+^*

The Löwdin analysis for the [Mg(G2)_2_]^⋅+^ and [Mg(MOEA)_3_]^⋅+^ radical cations was also performed on the RI-B3LYP(D3BJ)/def2-TZVPP level of theory. The results are summarized in **Scheme S5** and **Table S5**.


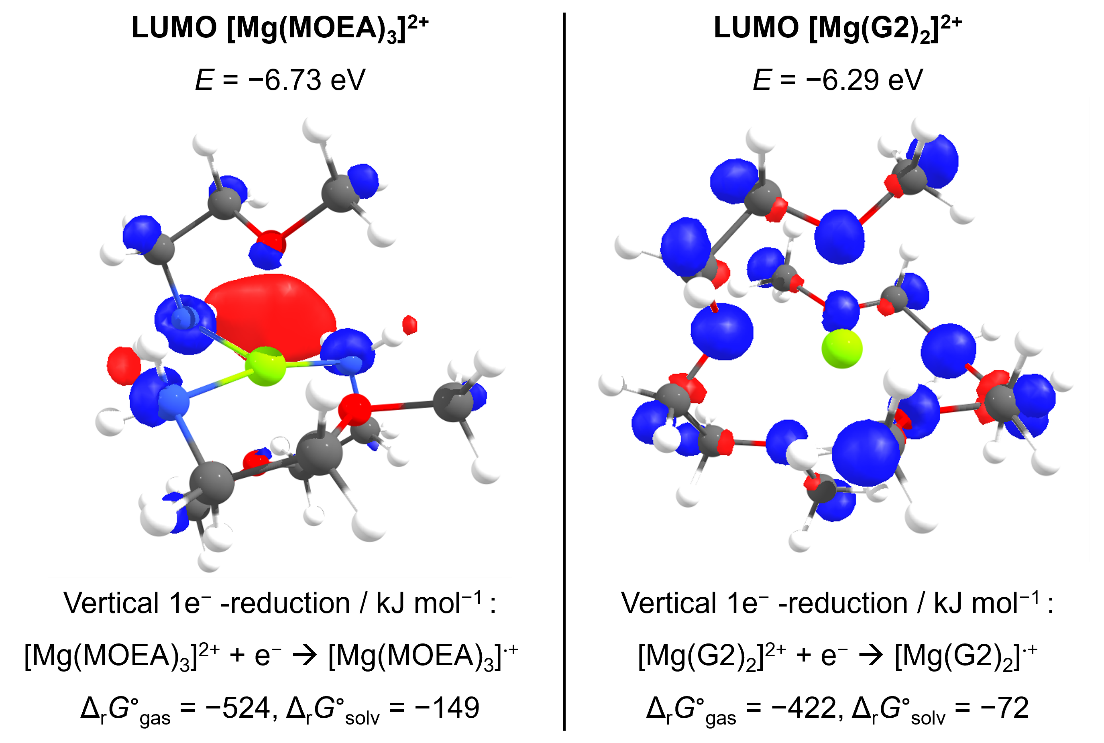


**Scheme S5**. Investigation of the LUMO orbitals of the [Mg(MOEA)_3_]^2+^ and [Mg(G2)_2_]^2+^ ions and their first vertical 1 e^–^ reduction by DFT calculation (RI−B3LYP(D3BJ)/def2-TZVPP; isosurface values: 0.045 a.u.).

**Table S5**: Summary of the Löwdin population analysis for the [Mg(G2)_2_]^⋅+^ and [Mg(MOEA)_3_]^⋅+^ radical cations (RI-B3LYP(D3BJ)/def2-TZVPP; single point calculations were performed on the optimized structures of the dications, thus investigating the complex obtained upon vertical one-electron reduction, without relaxation).

|  | **Spin Population on Atom Types** | | | | | |
| --- | --- | --- | --- | --- | --- | --- |
| **Complex** | Mg atom | C atoms | O atoms | C**H** atoms | N atoms | N**H** atoms |
| [Mg(G2)_2_]^⋅+^ | 0.097 | 0.028–0.034 | 0.015–0.017 | 0.010–0.020 | - | - |
| [Mg(MOEA)_3_]^⋅+^ | 0.129 | 0.010–0.018 | 0.005–0.007 | 0.003–0.023 | 0.018–0.027 | 0.045–0.093 |

*2.3 DFT Calculations to investigate the role of Mg-hydroxide complexes in the two electrolyte systems.*

The analysis of the hydroxylation of the [Al(Ohfip)_4_]^−^ anion revealed that hydroxide ions are present in the electrolyte system. In the following we analyze the potential importance of those in the two electrolyte systems and also with respect to the observed surface etching of the Mg interface in [Al(Ohfip)_4_]^−^ electrolyte, but its absence in the triflate electrolyte. This first involved the question, as to whether solvated magnesium-hydroxide complexes are favorable in solution in the two distinctly different electrolyte environments, that we address separately.

**Situation in the Mg[Al(Ohfip)_4_]_2_/G2-MOEA system:**


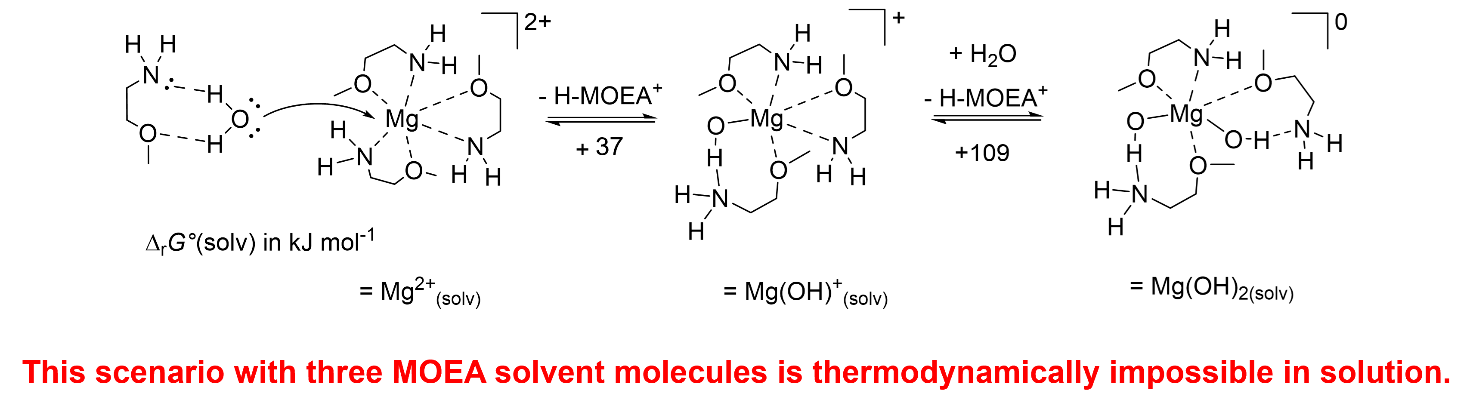


**Scheme S6**: Calculated free reaction enthalpies Δ_r_G° in a G2 solution for the formation of solvated [Mg(OH)]^+^ and [Mg(OH)_2_]^0^ species, in the absence of the [TfO]^−^ anion (RI-B3LYP(D3BJ)/def2-‍‍TZVPP; ε_r_ = 7.23).


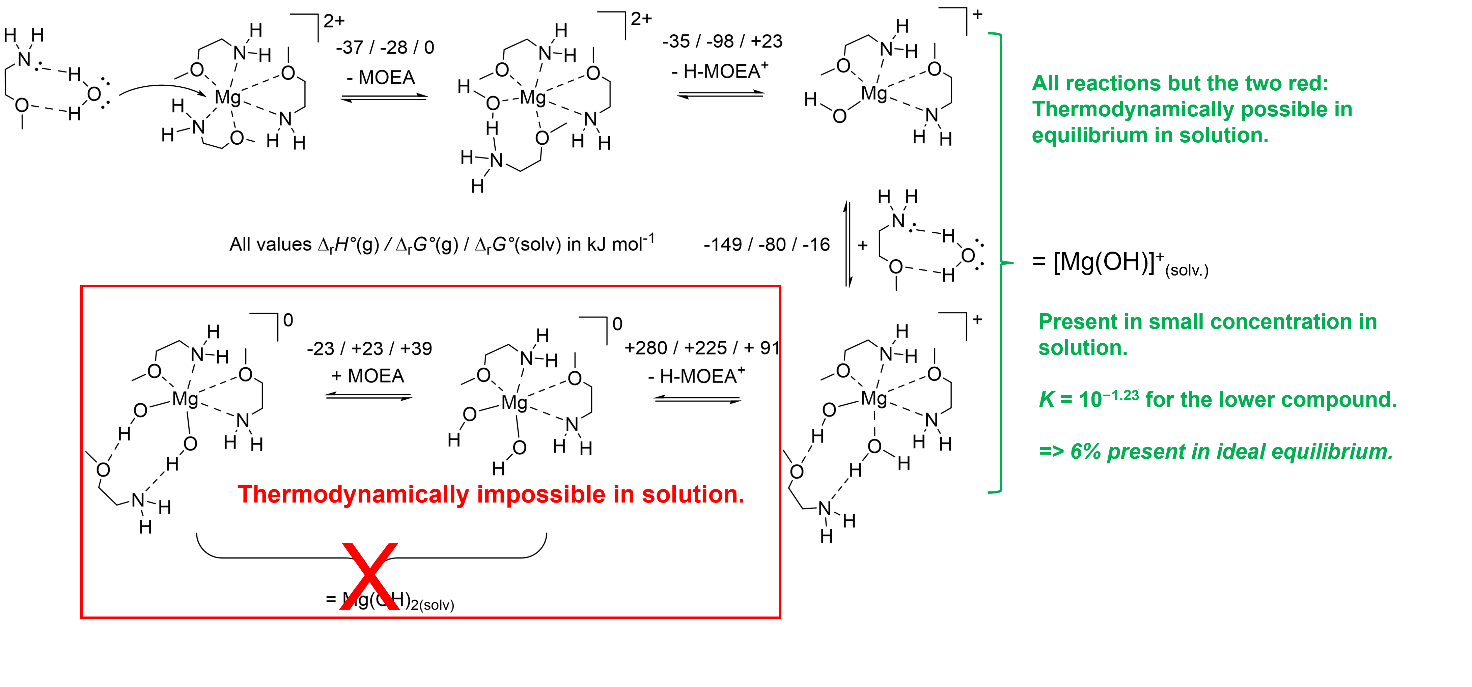


**Scheme S7**: Calculated reaction enthalpies Δ_r_H° and free reaction enthalpies Δ_r_G° in the gas phase and in a G2 solution, describing different plausible equilibria in a solution of Mg[Al(Ohfip)_4_]_2_ (RI-B3LYP(D3BJ)/def2-TZVPP; ε_r_ = 7.23).

**Situation in the Mg[TfO]_2_/G2-MOEA system:**


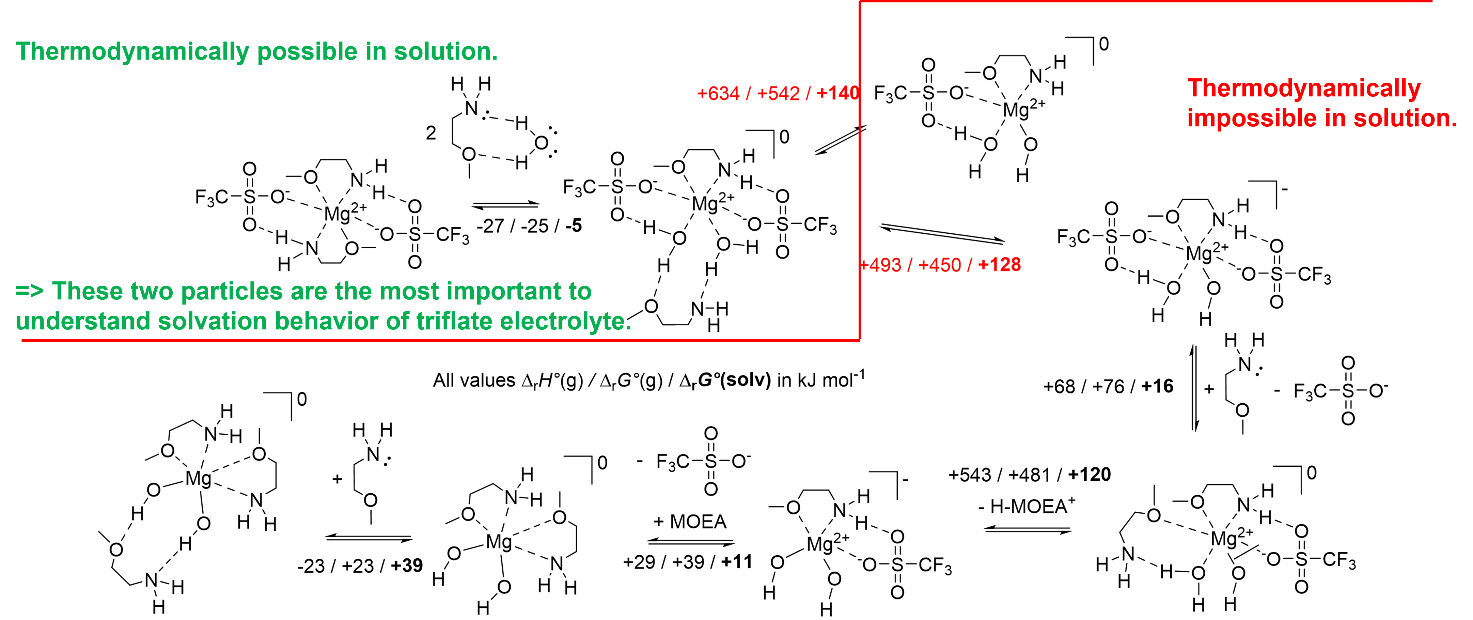


**Scheme S8**: Calculated reaction enthalpies Δ_r_H° and free reaction enthalpies Δ_r_G° in the gas phase and in a G2 solution, describing different plausible equilibria in a solution of Mg[TfO]_2_ (RI-B3LYP(D3BJ)/def2-TZVPP; ε_r_ = 7.23)

**§3 Solubility and basic electrochemistry**

- 1. *Salt solubility.*

Amines are recognized as strongly solvating solvents owing to their relatively high Gutmann donor numbers (*DNs*). The *DN* of a representative amine, triethylamine, is 61.0—approximately three times higher than those of conventional ethers, which typically range from 18 to 20.^[31,32]^ Despite the large *DNs* of amines, the solubility of Mg salts in alkylamines remains limited, as illustrated in Figures S1 and S2. Even Mg salts containing weakly-coordinated anions (WCAs) are insoluble in ternary amines but dissolve in secondary amines, indicating a significant contribution of hydrogen bonding for nitrogen-based solvation to the stabilization of ionic species. Mismatch against the Pearson’s hardness/softness concept also contributes to the rather low solubility of Mg salts especially in tertiary amine solvents, where bond energies between the alkylated nitrogen (soft base) and Mg^2+^ (hard acid) are relatively low and provide not enough driving force for solubilization. Moreover, the inherently low dielectric nature of amines contributes to the limited solubilization they offer for salts; the dielectric constants of typical alkylamines are below 4, approaching those of carbon disulfide and toluene.^[32]^ Hence, the low dielectric constant of soft alkylamines prevents effective stabilization of isolated ionic species. In contrast, glymes exhibit higher permittivities than amines.^[32]^ The hard base nature of ether oxygens, based on Pearson’s concept, also help to explain the marked stabilization of Mg^2+^ in solutions. Functionalization of amines with such ether oxygen atoms significantly enhances the solubility of Mg salts owing to the improved dielectric characteristics and additional chelating effects provided by polyether oxygen donors. Consequently, coupling multidentate glymes with strongly solvating alkoxyalkylamines possessing both, hydrogen substituted nitrogen-, as well as oxygen-donors, is an effective strategy to substantially increase the solubility of the highly associated Mg[TfO]_2_ salt by establishing a balanced solvation–dissociation equilibrium in solution.


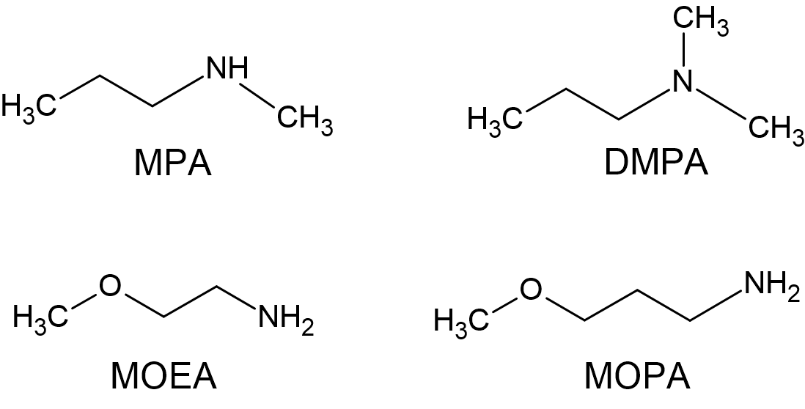


**Figure S1**. Chemical structures of the screened amine molecules.


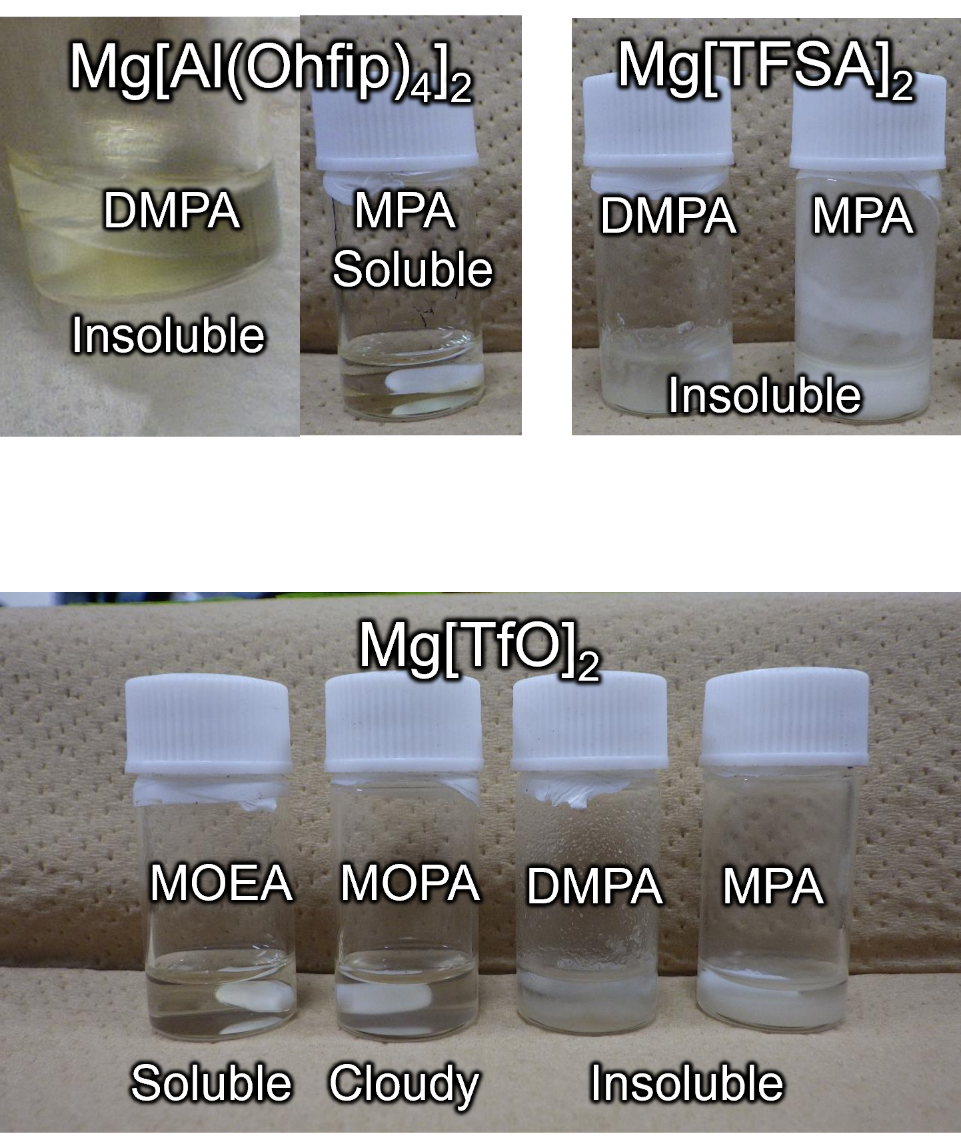


**Figure S2**. Digital photographs of the amine-Mg salt mixtures.

- 1. *Basic electrochemistry*

Figure S3 summarizes the CV profiles of Pt and Mg working electrodes recorded in single G2, single MOEA, and G2–MOEA (1:1 v/v) dual solvent mixtures using Mg[Al(Ohfip)_4_]_2_ and Mg[TfO]_2_ as conductive salts. All measurements were performed in a three-electrode configuration, employing magnesium metal and Ag^+^/Ag as the counter and reference electrodes, respectively. The electrode potential of the Ag^+^/Ag reference was calibrated using typical Grignard reagent–based (C_2_H_5_MgCl/THF) and Mg[Z(Ohfip)_4_]_2_/G2 (Z = B or Al) electrolytes.^[7,33]^

For electrolyte solutions containing the dissociative Mg[Al(Ohfip)_4_]_2_ salt, they exhibited favorable Mg plating/stirpping performance irrespective of the solvents. Incorporation of MOEA is found to be particularly effective in minimizing the overpotential during the Mg stripping process. In the case of Mg[TfO]_2_, their electrochemical Mg plating/stripping performance was markedly improved using G2-MOEA mixture. Although the current density is smaller than that of the corresponding Mg[Al(Ohfip)_4_]_2_-based system, possibly because of its lower ionic conductivity (Figure S12), the electrochemical response of the Mg electrodes exhibited a linear current–potential relationship throughout the scanning range. This behavior indicates that no significant activation barrier is required to initiate the Mg plating/stripping reactions and that Mg^2+^ ions diffuse smoothly under the present experimental conditions.

It should be mentioned that the Mg[TfO]_2_/G2-MOEA system displayed non-trivial behavior in which the potentials corresponding to Mg plating and stripping differed between the working electrodes. Subsequent visual and microscopic observations confirmed that the reversible responses at both electrodes can be attributed to genuine Mg plating/stripping reactions. Although the underlying origin of this phenomenon remains unclear, the unique interfacial characteristics of amines (vide infra) and electrode-dependent characteristics for hydrogen evolution reactions may contribute to such anomalous behavior. Elucidating the detailed mechanism responsible for the observed potential shift lies beyond the scope of the present study.


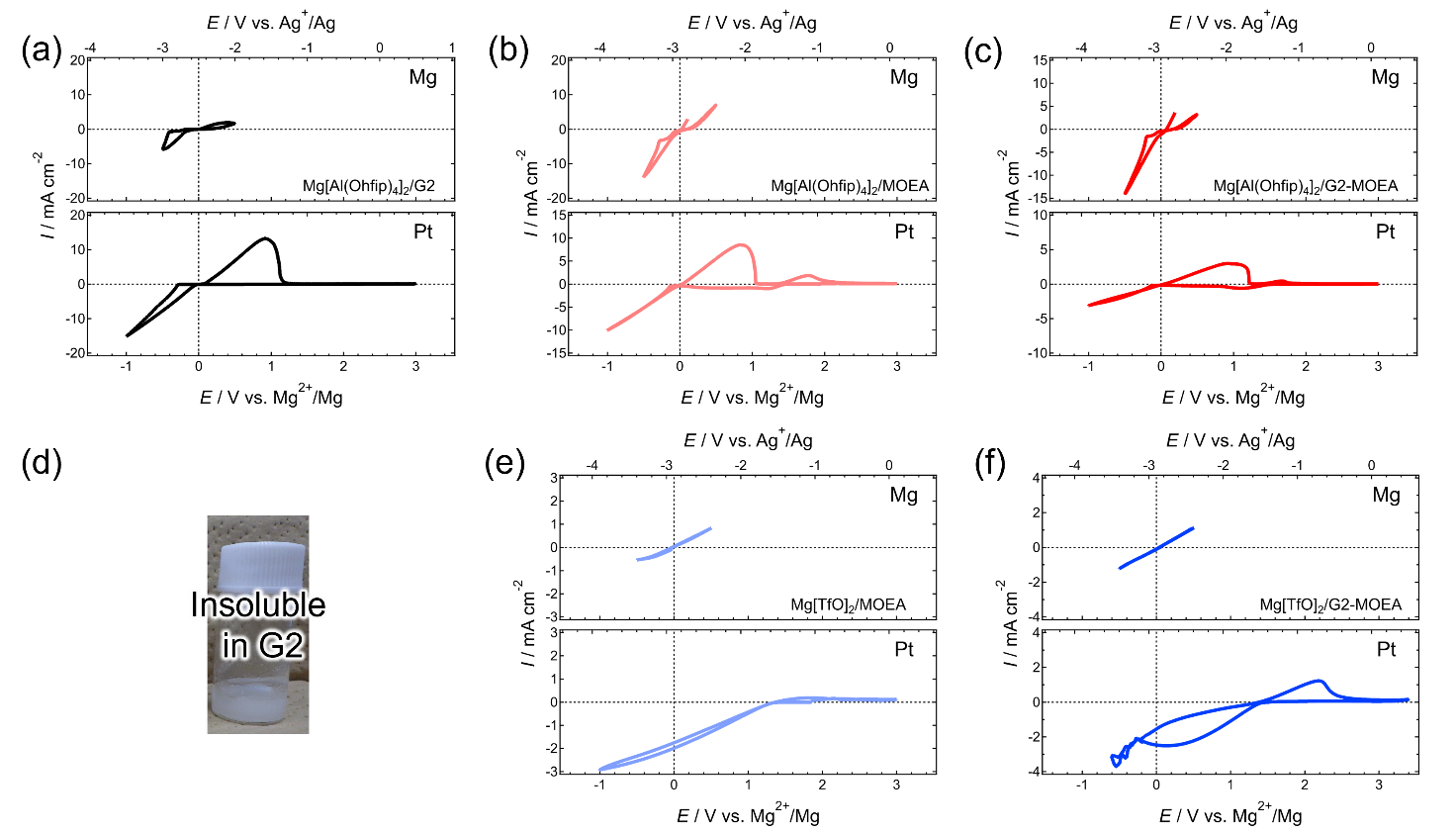


**Figure S3**. CV profiles of Pt and Mg electrodes recorded in (a, d) single G2-, (b, e) single MOEA-, and (c, f) dual G2–MOEA-based electrolyte solutions at a scan rate of 10 mV s^−1^, using Ag^+^/Ag as an external reference. The potentials vs. Ag^+^/Ag with only G2 are by about 400 mV less negative than those using the MOEA solvent. The conductive Mg salts employed were (a–c) Mg[Al(Ohfip)_4_]_2_ and (d–f) Mg[TfO]_2_. Due to the extremely low solubility of Mg[TfO]_2_ in G2, as shown in (d), the corresponding CV profile could not be obtained. The profiles of the initial cycles are presented to highlight the initial plating/stripping overpotential. Note that for the parallel CV measurements, various unidentified factors including the surface state of electrodes and spatial arrangement of each electrode can affect the observed current density.

In the electrolyte solutions, G2 plays the specific role not only offering good ionic conductivity but also constructing favorable interface. The galvanostatic cycling profiles using the 1:9 (v/v) G2:MOEA mixture with the Mg[TfO]_2_ salt are obviously inferior to those using the 1:1 (v/v) mixture (Figure S4). According to this brief compositional study, the 1:1 (v/v) mixture would be selected as the benchmarking solvent composition in this study.


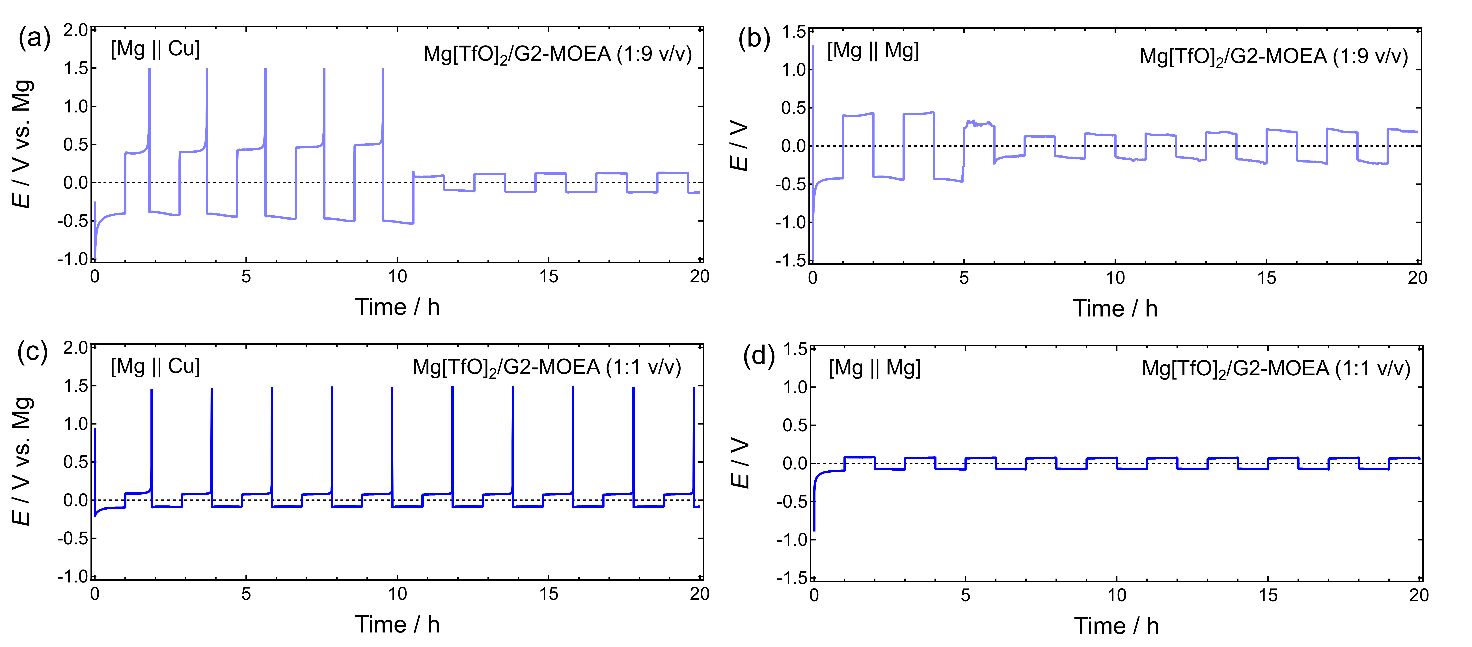


**Figure S4**. Galvanostatic cycling profiles of (a, c) [Mg || Cu] and (b, d) [Mg || Mg] cells using (a, b) Mg[TfO]_2_/G2-MOEA (1:9 v/v) and (c, d) Mg[TfO]_2_/G2-MOEA (1:1 v/v) measured at a current density of 1 mA cm^−2^ and 30 °C.

**§4 Supporting Figures**


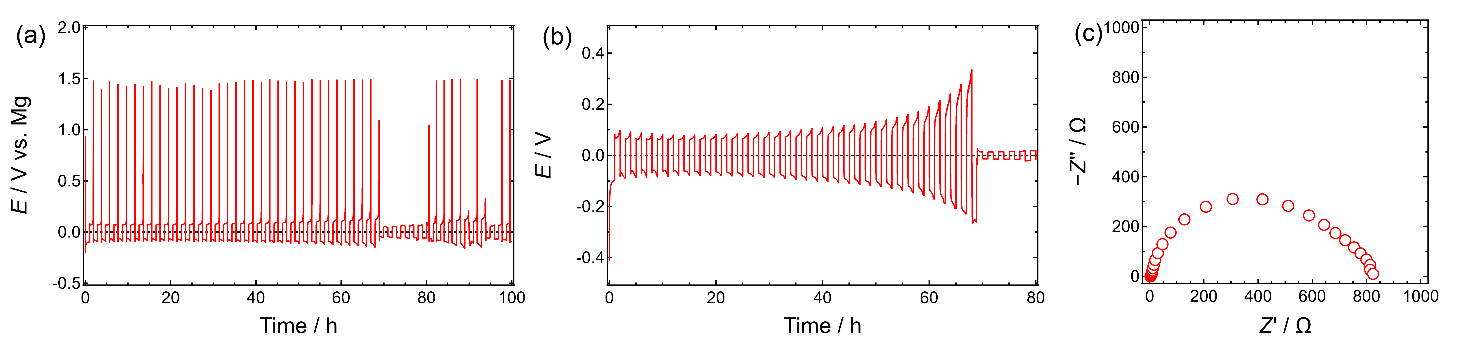


**Figure S5**. Galvanostatic Mg plating/stripping cycling profiles of (a) asymmetric [Mg || Cu] and (b) symmetric [Mg || Mg] cells recorded in Mg[B(Ohfip)_4_]_2_/G2-MOEA at a current density of 1 mA cm^−2^ at 30 °C. (c) Nyquist plot of the [Mg || Mg] cell before the cycling measurements.


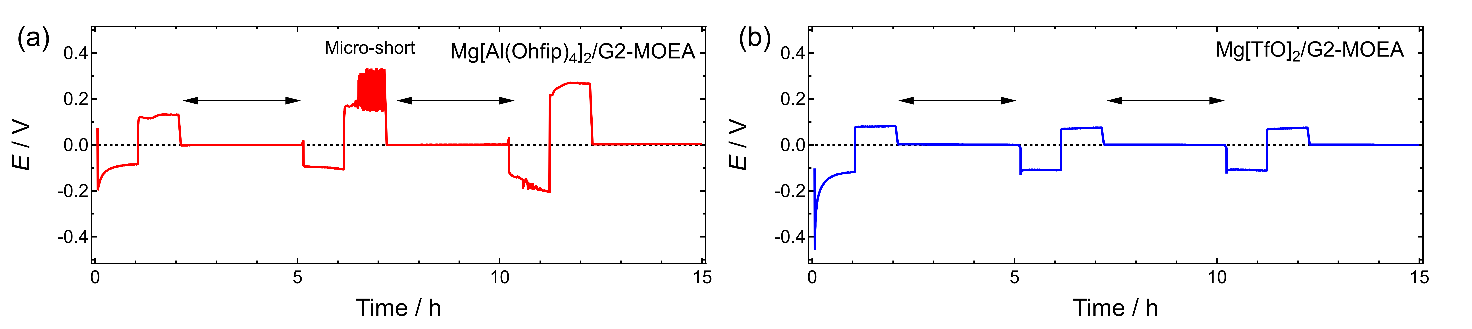


**Figure S6**. Galvanostatic cycling-hold measurements of symmetric [Mg || Mg] cells recorded in (a) Mg[Al(Ohfip)_4_]_2_/G2-MOEA and (b) Mg[TfO]_2_/G2-MOEA at a current density of 1 mA cm^−2^ at 30 °C. Arrows in the figures indicate the rest period (3 hour). The voltage fluctuations observed in (a) are indicative of occurrence of successive micro-short-circuiting events.


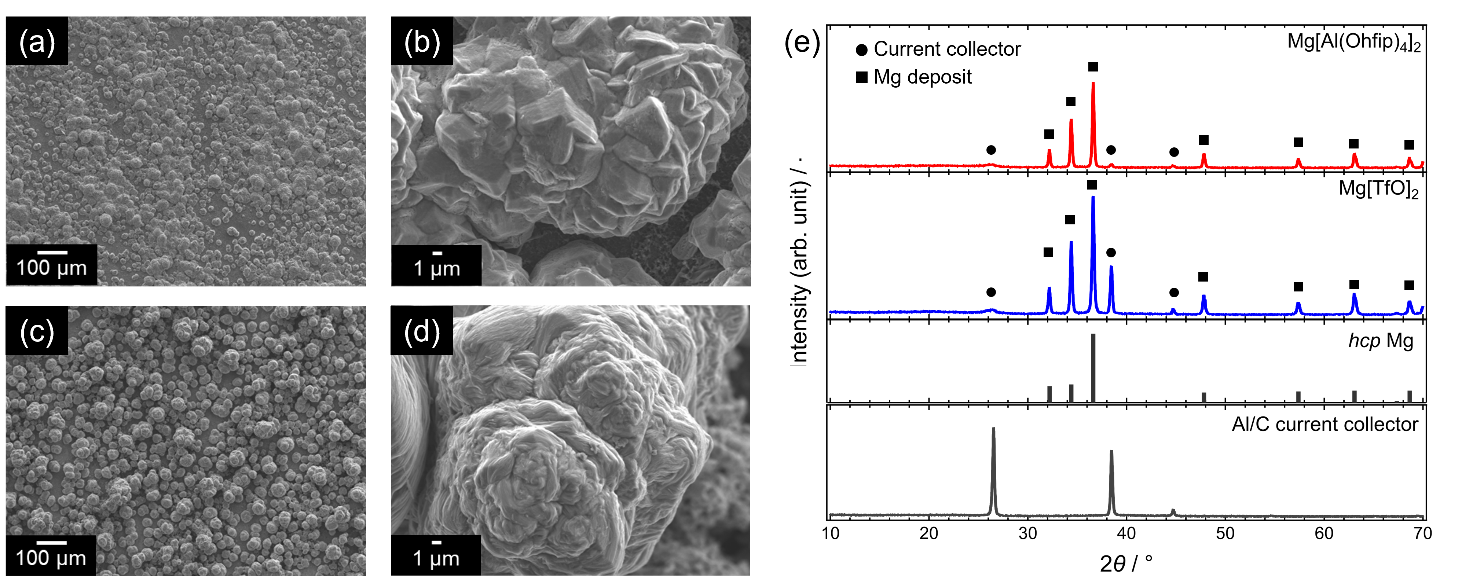


**Figure S7**. SEM images of Mg deposits obtained by galvanostatic deposition at 1 mA cm^−2^ for 2 h from (a, b) Mg[Al(Ohfip)_4_]_2_/G2-MOEA and (c, d) Mg[TfO]_2_/G2-MOEA electrolytes. Panels (a, c) show bird’s-eye views, and (b, d) present the corresponding magnified images. (e) pXRD profiles of the same deposits on the carbon-coated Al (Al/C) substrate. Simulated profiles of Mg as well as the experimental profile of Al/C are included as references.


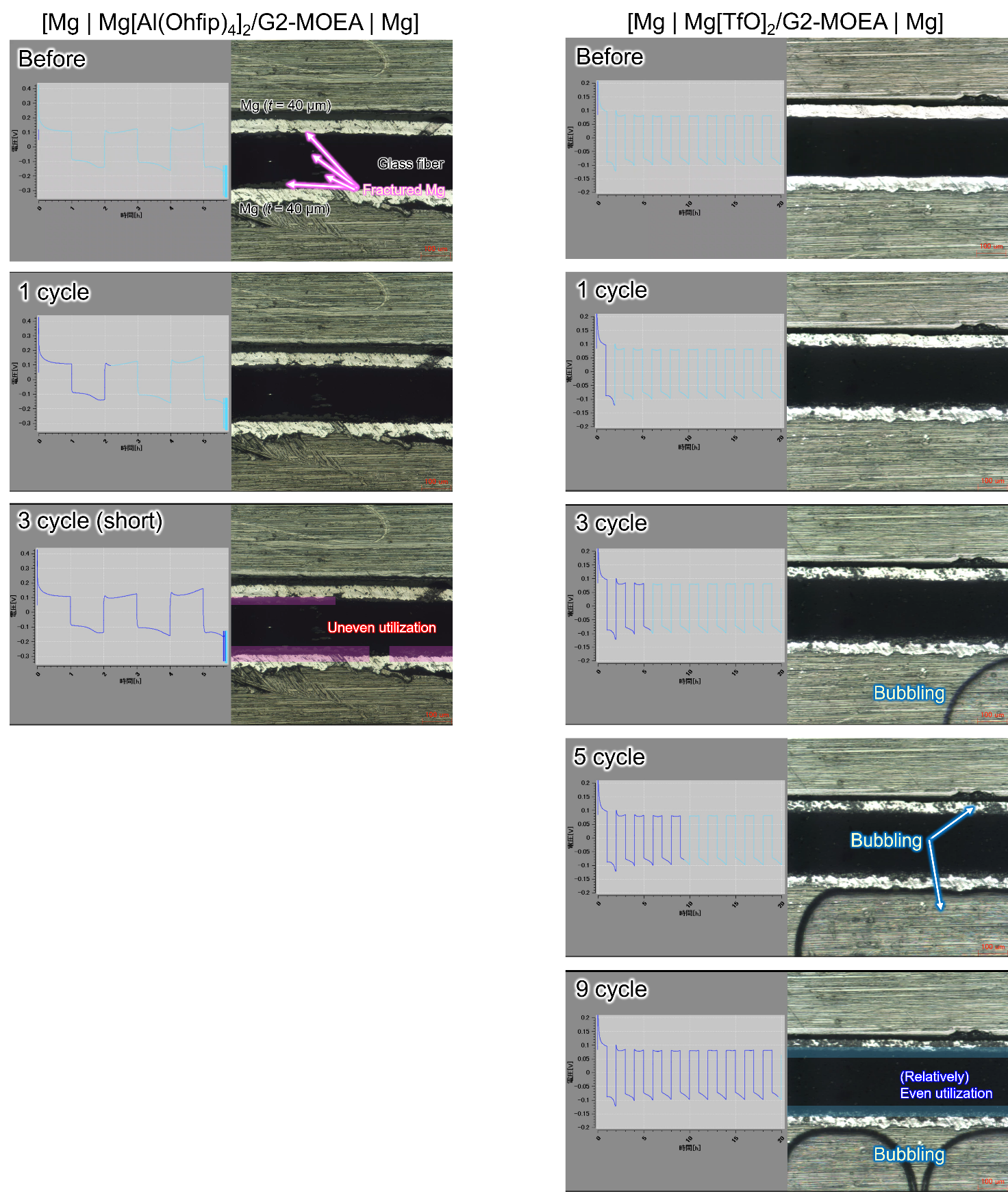


**Figure S8**. Snapshots of the corresponding ECCS measurements (Videos 1 and 2).


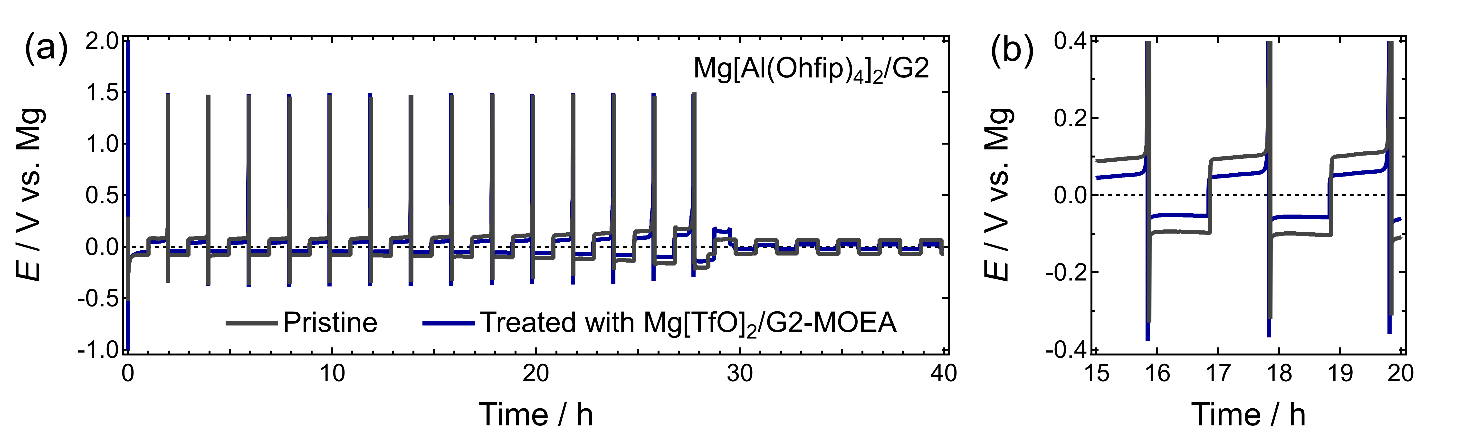


**Figure S9**. Galvanostatic Mg plating/stripping cycling profiles of asymmetric [Mg || Cu] cells using pristine Mg electrodes and Mg electrodes precycled in Mg[TfO]_2_/G2-MOEA. An ethereal electrolyte of Mg[Al(Ohfip)_4_]_2_/G2 was used for the measurements.


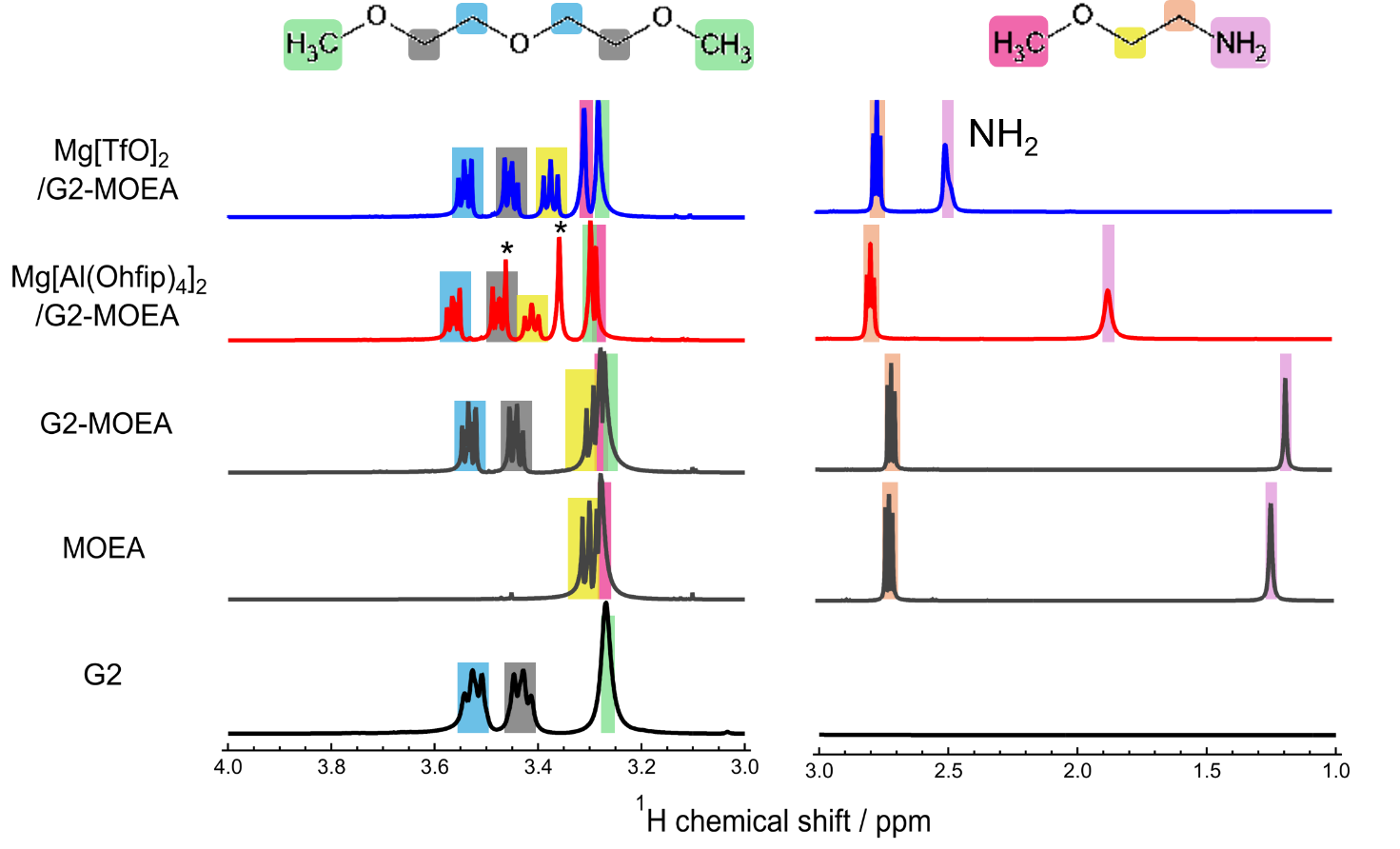


**Figure S10**. ^1^H NMR (400.17 or 300.18 MHz) spectra of Mg[Al(Ohfip)_4_]_2_/G2-MOEA and Mg[TfO]_2_/G2-MOEA electrolytes measured at 25 °C. The corresponding spectra of the individual solvents and the dual-solvent mixture are included as references. Asterisks (*) indicate the protons of G1, which acts as a crystal solvent in the Mg[Al(Ohfip)_4_]_2_ salt (three molar equivalents).


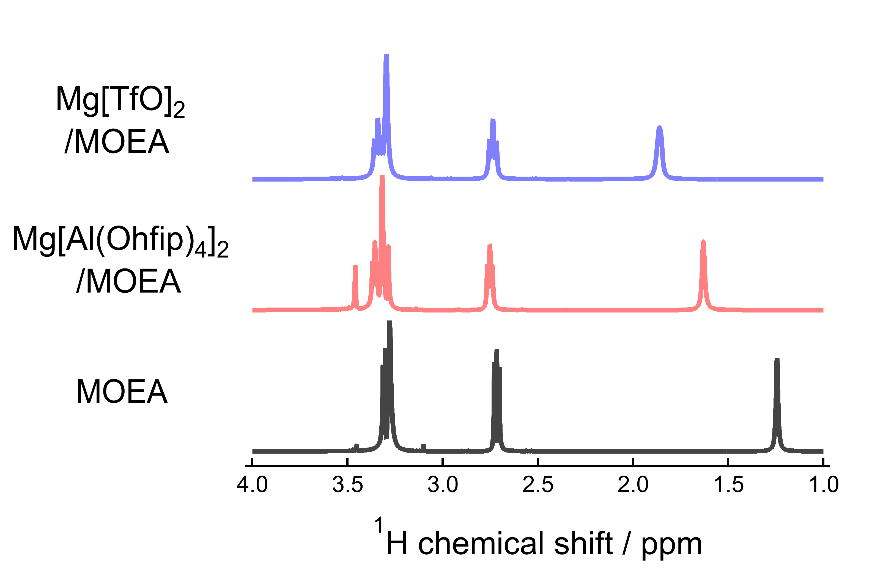


**Figure S11**. ^1^H NMR spectra (400.17 or 300.18 MHz) of MOEA, Mg[Al(Ohfip)_4_]_2_/MOEA, and Mg[TfO]_2_/MOEA at 298 K.


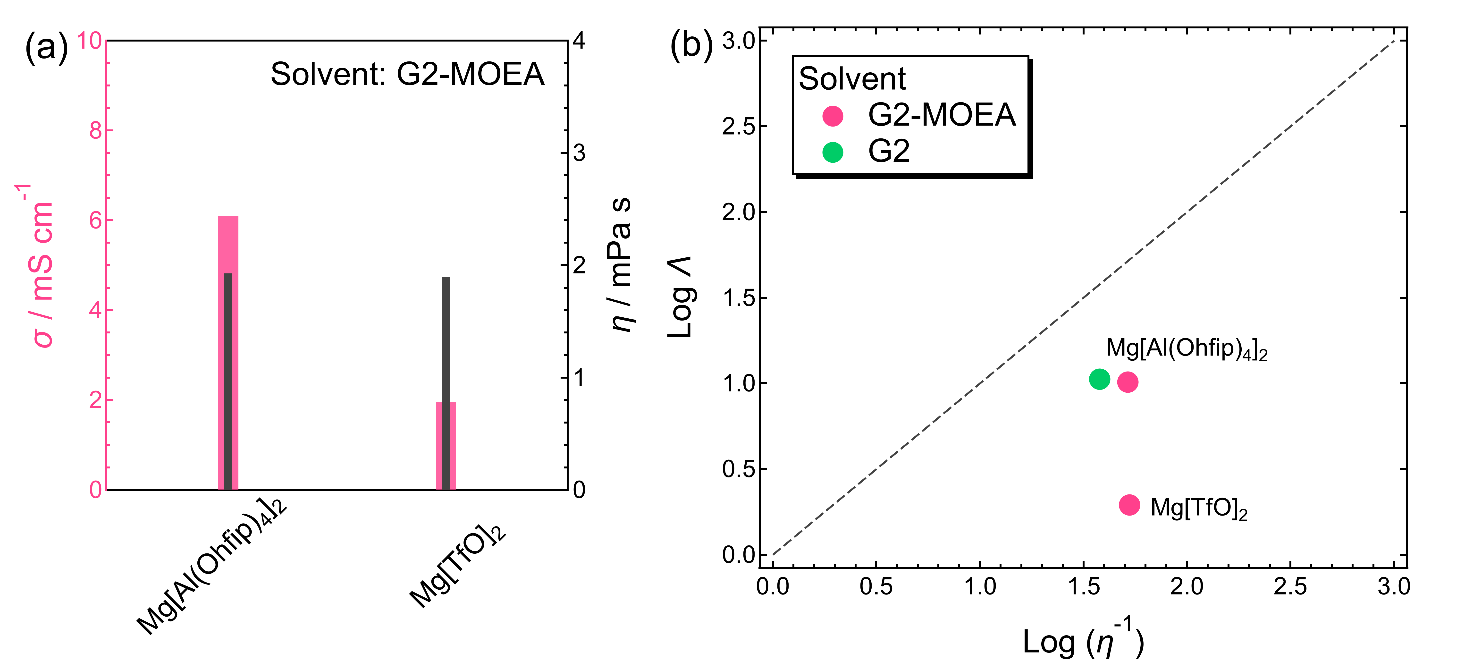


**Figure S12**. (a) Ionic conductivities and viscosities and (b) Walden plots of the G2 and G2-MOEA solutions of Mg[Al(Ohfip)_4_]_2_ and Mg[TfO]_2_ measured at 30 °C.


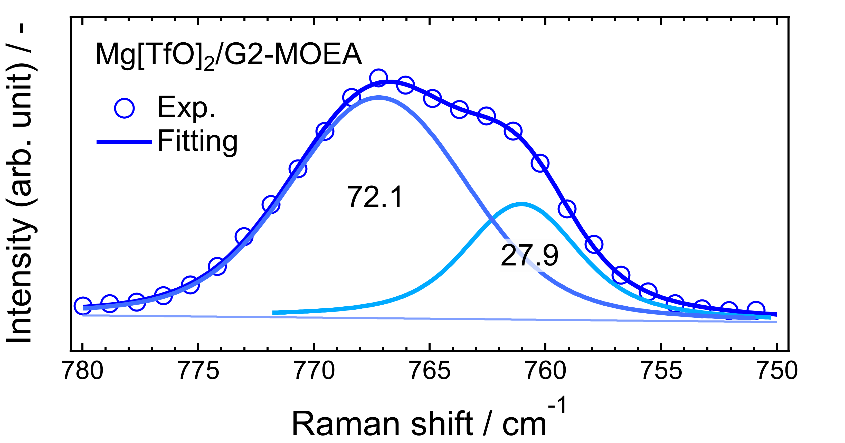


**Figure S13**. Raman spectrum showing the δ_s_(CF_3_) modes of [TfO]^−^ for Mg[TfO]_2_/G2-MOEA. A spectral deconvolution was performed using the Voigt function. Relative peak areas are included in the figure.


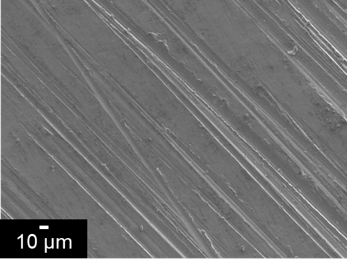


**Figure S14**. SEM images of Mg electrodes soaked in Mg[Al(Ohfip)_4_]_2_/G2 for 48 h at 30 °C.


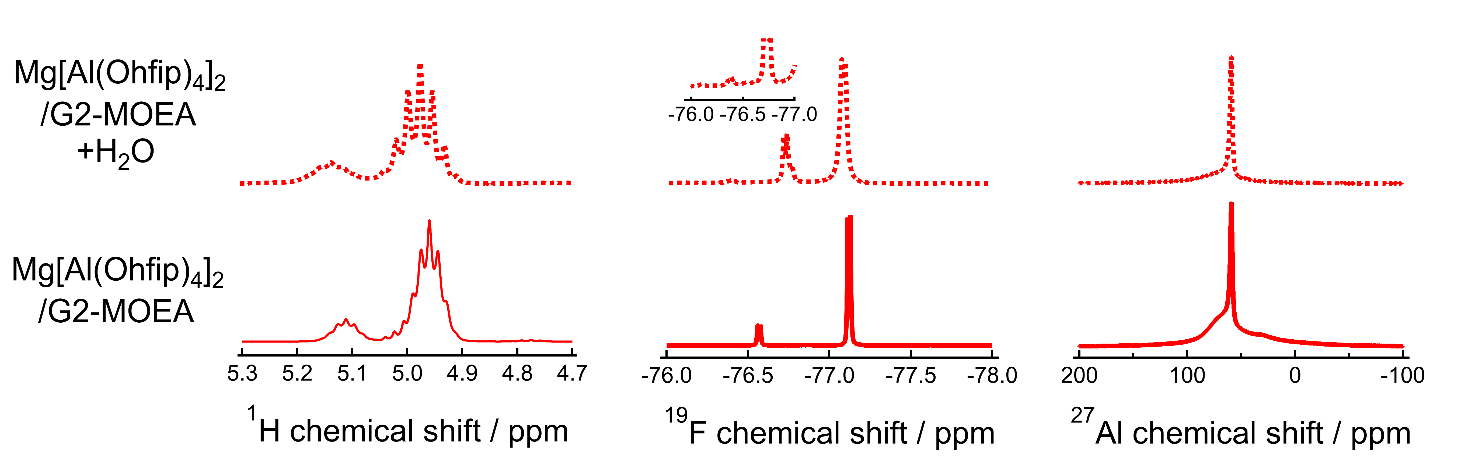


**Figure S15**. ^1^H, ^19^F, and ^27^Al NMR spectra (either 400.17, 376.4 and 104.27 MHz or 300.18, 282.45, and 78.22 MHz, respectively) of the as-prepared Mg[Al(Ohfip)_4_]_2_/G2-MOEA and that containing ca. 0.5 mol dm^−3^ of water at 298 K. The spectra shown here were not calibrated with TMS. An additional small peak at about −76.5 ppm in the ^19^F spectrum of the electrolyte containing excess water is assignable to the signal from the reaction products. The intensive broad resonance between 0 and 100 ppm in the ^27^Al-NMR spectrum is a background signal from the probehead, whereas the small broad shoulder at 30 ppm is a signal from the sample.


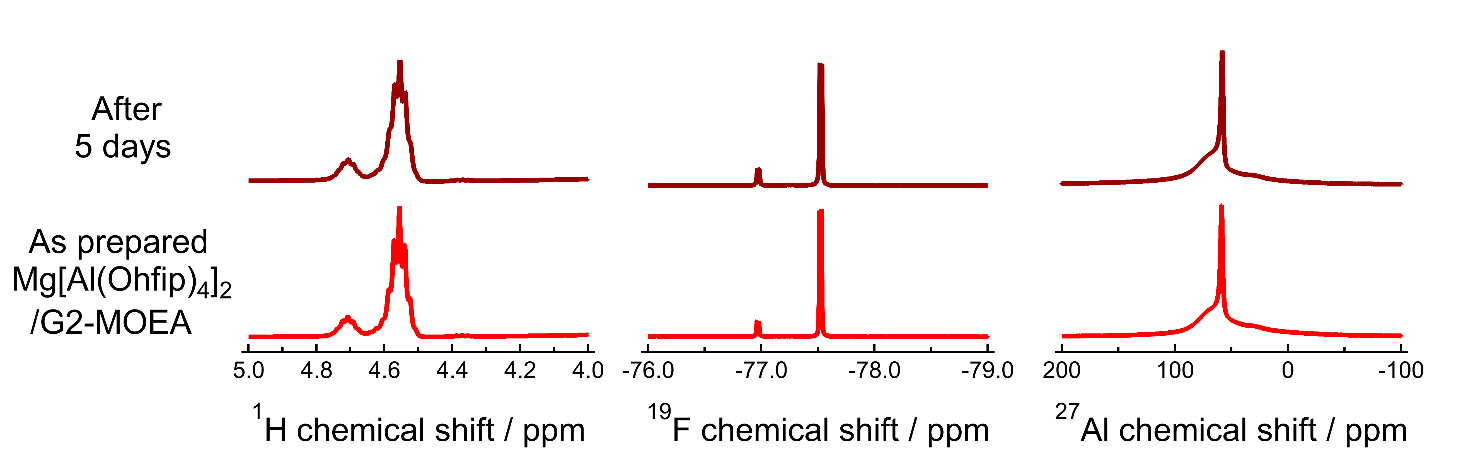


**Figure S16**. ^1^H, ^19^F, and ^27^Al NMR spectra (400.17, 376.4 and 104.27 MHz) of the as-prepared and aged Mg[Al(Ohfip)_4_]_2_/G2-MOEA at 298 K. Certain spectral ranges relevant for [Al(Ohfip)_4_]^−^ are shown. The intensive broad resonance between 0 and 100 ppm in the ^27^Al-NMR spectrum is a background signal from the probehead, whereas the small broad shoulder at 30 ppm is a signal from the sample.


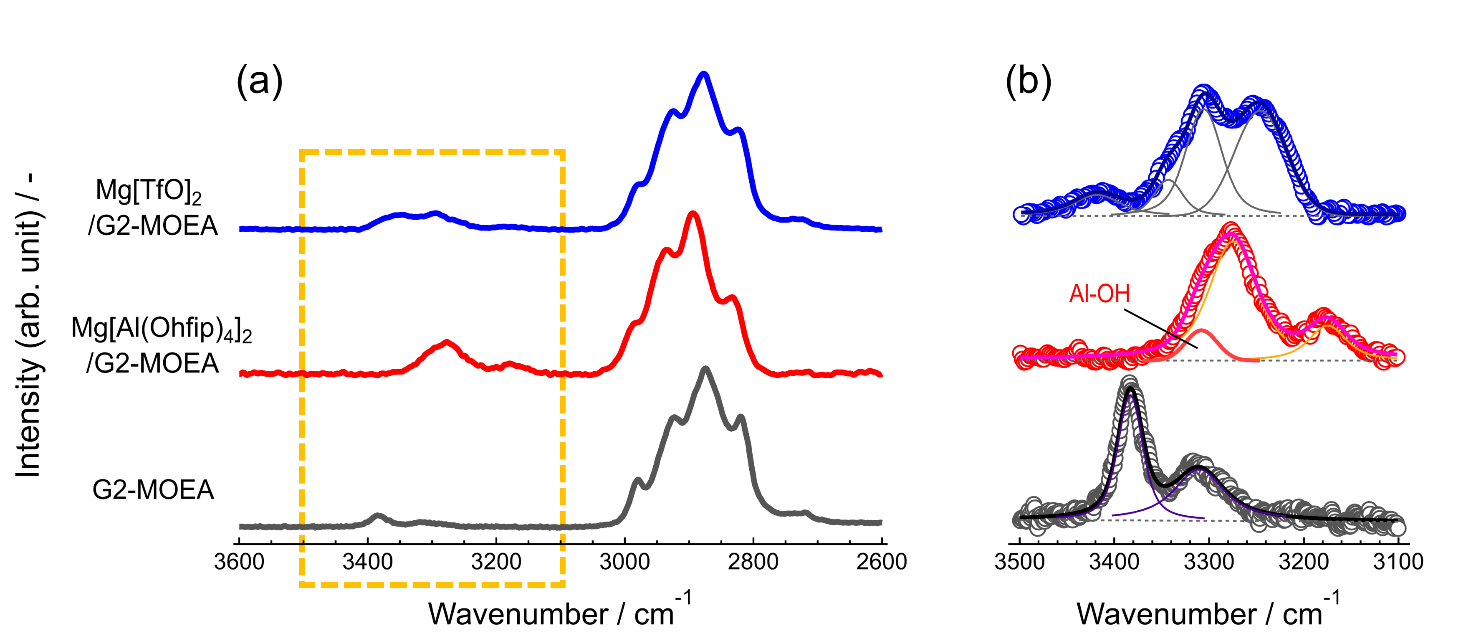


**Figure S17**. FT-ATR-IR spectra of Mg[Al(Ohfip)_4_]_2_/G2-MOEA and Mg[TfO]_2_/G2-MOEA showing the specific spectral range relevant to N-H, O-H, and C-H stretching vibration. (a) 3600–2600 cm^−1^ and (b) 3500–3100 cm^−1^ with spectral deconvolution by Voigt functions (magnified region marked in (a)). The spectra of G2-MOEA are also included as a reference. The redshift of N-H vibrational modes (at 3383 and 3310 cm^−1^ in the blank G2-MOEA solution) in the electrolytes suggests H-bonding. The shoulder peak at around 3308 cm^−1^ for Mg[Al(Ohfip)_4_]_2_/G2-MOEA is attributable to O-H stretching of the reaction products.


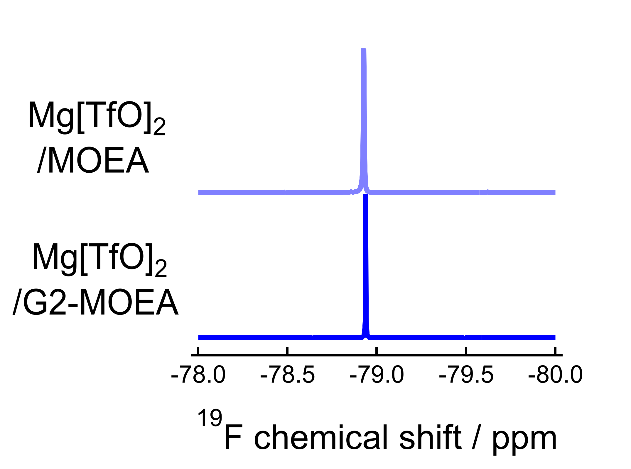


**Figure S18**. ^19^F NMR spectra (376.54 or 282.45 MHz) of Mg[TfO]_2_/G2-MOEA and Mg[TfO]_2_/MOEA at 298 K.


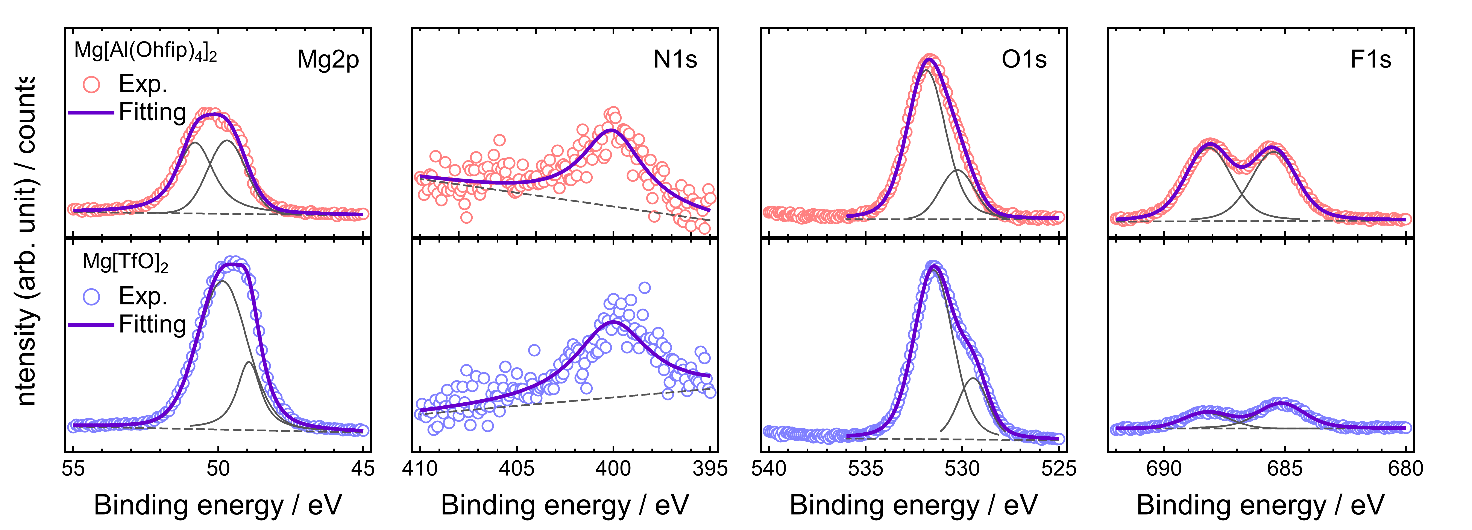


**Figure S19**. Mg 2p, N 1s, O 1s, and F 1s (left to right) XPS spectra of Mg electrodes cycled in (upper) Mg[Al(Ohfip)_4_]_2_/G2-MOEA and (lower) Mg[TfO]_2_/G2-MOEA. After electrochemical Mg plating/stripping cycling, the nitrogen and fluorine species can be detected on the Mg surface cycled in Mg[Al(Ohfip)_4_]_2_/G2-MOEA.


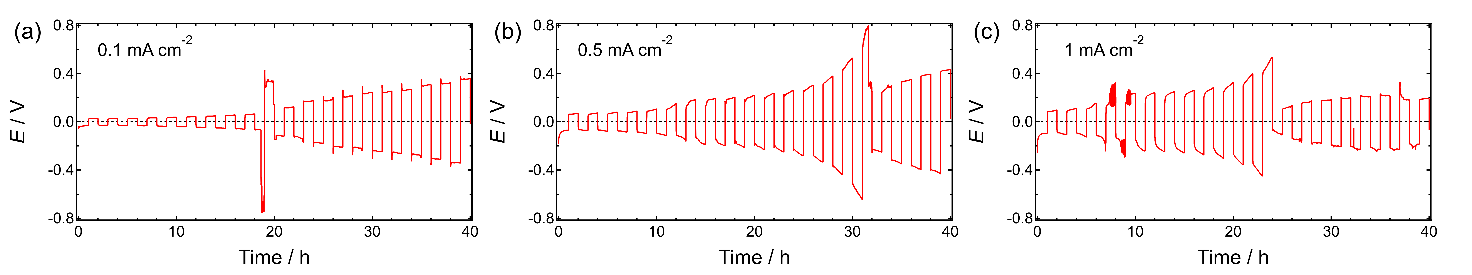


**Figure S20**. Galvanostatic Mg plating/stripping cycling profiles of [Mg || Mg] symmetric cells recorded in Mg[Al(Ohfip)_4_]_2_/G2-MOEA at a current density of (a) 0.1, (b) 0.5, and (c) 1 mA cm^−2^ at 30 °C.


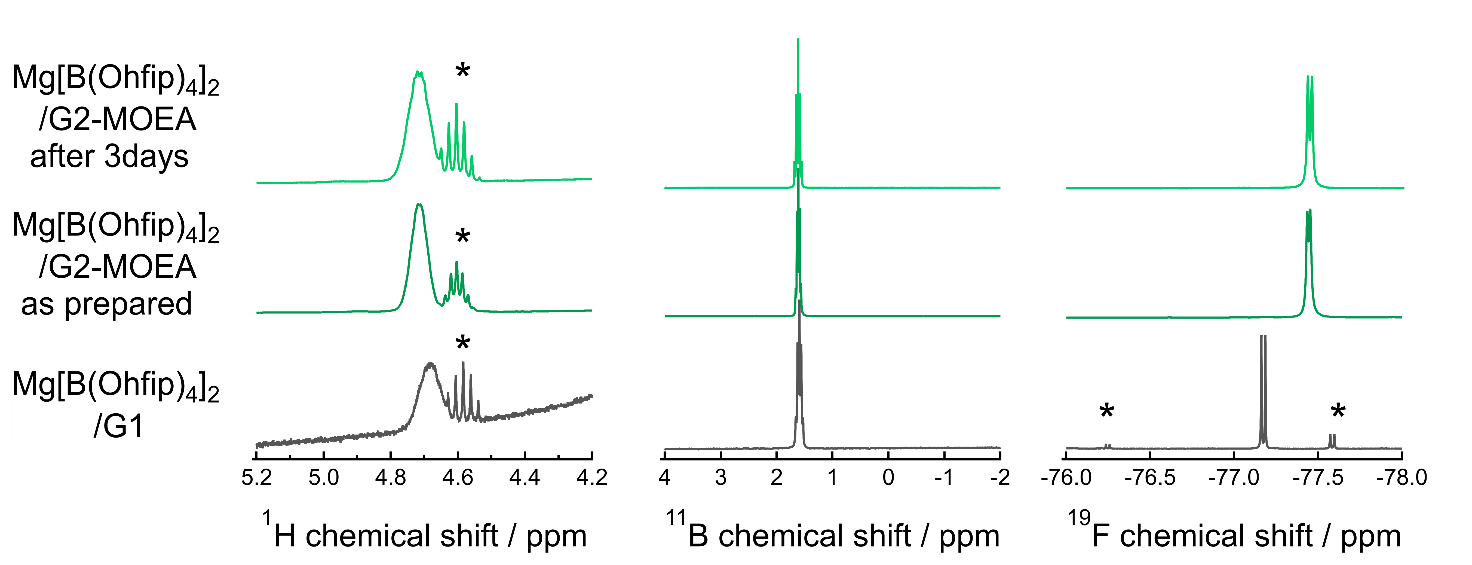


**Figure S21**. ^1^H, ^11^B, and ^19^F NMR spectra (either 400.17, 128.4, and 376.4 MHz, or 300.18, 96.31, and 282.45 MHz, respectively) of Mg[B(Ohfip)_4_]_2_/G2-MOEA as prepared and aged for 3days measured at 298 K. The spectra of Mg[B(Ohfip)_4_]_2_/G1 are also included as a reference. The asterisks shown in the spectra are assignable to impurities, such as Mg(Ohfip)_2_ and B(Ohfip)_3_, in the parent salt. The substantial upfield shift of the ^19^F signal of Mg[B(Ohfip)_4_]_2_/G2-MOEA from that of Mg[B(Ohfip)_4_]_2_/G1 is due to the change in the solution environment. Observation of no new peak evolution upon mixing Mg[B(Ohfip)_4_]_2_ salt with the G2-MOEA solvent indicates sufficient stability of [B(Ohfip)_4_]^−^ against hydroxide ions. Broadening of the peaks over time however suggests gradual decomposition (hydrolysis) of the anion.


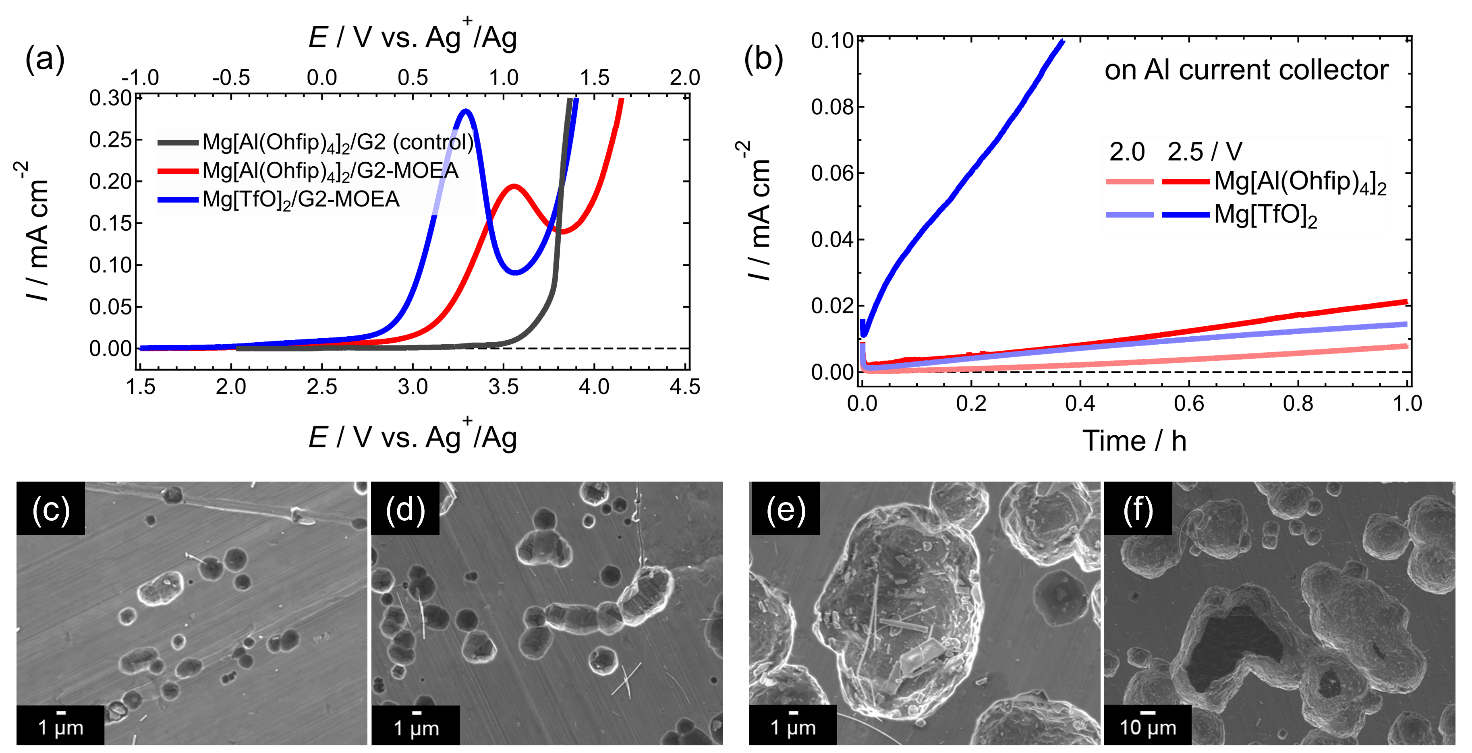


**Figure S22**. (a) LSV and (b) chronoamperometric profiles of Pt and Al working electrodes, respectively, recorded in Mg[Al(Ohfip)_4_]_2_/G2-MOEA and Mg[TfO]_2_/G2-MOEA electrolytes. An LSV profile of Pt working electrode recorded in the MOEA-free Mg[Al(Ohfip)4]2/G2 electrolyte was included as a reference in (a). (c)-(f) SEM images of Al current collector after corresponding chronoamperometry measurements polarized at (c,e) 2.0 V and (d,f) at 2.5 V in (c,d) Mg[Al(Ohfip)_4_]_2_/G2-MOEA and (e,f) Mg[TfO]_2_/G2-MOEA.

**References**

[1] T. Mandai, Y. Youn, Y. Tateyama, *Mater. Adv.* **2021**, *2*, 6283.

[2] T. Mandai, *Batteries & Supercaps* **2025**, *8*, e202500348.

[3] T. Mandai, *ACS Appl. Mater. Interfaces* **2020**, *12*, 39135.

[4] E. Levi, Y. Gofer, Y. Vestfreed, E. Lancry, D. Aurbach, *Chem. Mater.* **2002**, *14*, 2767.

[5] D. Setiawan, H. J. Kim, J. Lyoo, S.-T. Hong, M. S. Chae, *Chem. Eng. J.* **2023**, *474*, 145596.

[6] Hydranal^TM^ Laboratory Report L288, *Water determination in nitrogen bases*, http://lab.solstice.com/en/resources/hydranal-lab-reports/water-determination-in-nitrogen-bases. Accessed on 3^rd^ of June, 2026.

[7] T. Mandai, Y. Akita, S. Yagi, M. Egashira, H. Munakata, K. Kanamura, *J. Mater. Chem. A* **2017**, *5*, 3152.

[8] J. P. Perdew, *Phys. Rev. B* **1986**, *33*, 8822.

[9] J. P. Perdew, *Phys. Rev. B* **1986**, *34*, 7406.

[10] A. D. Becke, *Phys. Rev. A* **1988**, *38*, 3098.

[11] R. Ahlrichs, M. Bär, M. Häser, H. Horn, C. Kölmel, *Chem. Phys. Lett.* **1989**, *162*, 165.

[12] A. Schäfer, H. Horn, R. Ahlrichs, *J. Chem. Phys.* **1992**, *97*, 2571.

[13] K. Eichkorn, O. Treutler, H. Öhm, M. Häser, R. Ahlrichs, *Chem. Phys. Lett.* **1995**, *242*, 652.

[14] M. Von Arnim, R. Ahlrichs, *J. Comput. Chem.* **1998**, *19*, 1746.

[15] P. Deglmann, F. Furche, *J. Chem. Phys.* **2002**, *117*, 9535.

[16] O. Treutler, R. Ahlrichs, *J. Chem. Phys.* **1995**, *102*, 346.

[17] P. Deglmann, F. Furche, R. Ahlrichs, *Chem. Phys. Lett.* **2002**, *362*, 511.

[18] F. Neese, *WIREs Comput. Mol. Sci.* **2012**, *2*, 73.

[19] F. Neese, *WIREs Comput. Mol. Sci.* **2022**, *12*, e1606.

[20] M. Sierka, A. Hogekamp, R. Ahlrichs, *J. Chem. Phys.* **2003**, *118*, 9136.

[21] R. Ahlrichs, *Phys. Chem. Chem. Phys.* **2004**, *6*, 5119.

[22] F. Weigend, *Phys. Chem. Chem. Phys.* **2006**, *8*, 1057.

[23] S. Grimme, J. Antony, S. Ehrlich, H. Krieg, *J. Chem. Phys.* **2010**, *132*, 154104.

[24] S. Grimme, S. Ehrlich, L. Goerigk, *J. Comput. Chem.* **2011**, *32*, 1456.

[25] C. Lee, W. Yang, R. G. Parr, *Phys. Rev. B* **1988**, *37*, 785.

[26] A. D. Becke, *J. Chem. Phys.* **1993**, *98*, 1372.

[27] A. D. Becke, *J. Chem. Phys.* **1993**, *98*, 5648.

[28] F. Weigend, R. Ahlrichs, *Phys. Chem. Chem. Phys.* **2005**, *7*, 3297.

[29] A. Klamt, G. Schüürmann, *J. Chem. Soc., Perkin Trans. 2* **1993**, 799.

[30] K. Hoshi, T. Chiba, J. Sato, Y. Hayashi, Y. Takahashi, H. Ebe, S. Ohisa, J. Kido, *ACS Appl. Mater. Interfaces* **2018**, *10*, 24607.

[31] P. Zhou, Y. Xiang, K. Liu, *Energy Environ. Sci.* **2024**, *17*, 8057.

[32] Y. Marcus, *Chem. Soc. Rev.* **1993**, *22*, 409.

[33] T. Mandai, *ChemSusChem* **2025**, *18*, e202500418.
